# Structure of a photosystem I-ferredoxin complex from a marine cyanobacterium provides insights into far-red light photoacclimation

**DOI:** 10.1016/j.jbc.2021.101408

**Published:** 2021-11-15

**Authors:** Christopher J. Gisriel, David A. Flesher, Gaozhong Shen, Jimin Wang, Ming-Yang Ho, Gary W. Brudvig, Donald A. Bryant

**Affiliations:** 1 Department of Chemistry, Yale University, New Haven, Connecticut, USA; 2 Department of Molecular Biophysics and Biochemistry, Yale University, New Haven, Connecticut, USA; 3 Department of Biochemistry and Molecular Biology, The Pennsylvania State University, University Park, Pennsylvania, USA; 4 Department of Life Science, National Taiwan University, Taipei, Taiwan

**Keywords:** photosystem I, far-red light photoacclimation, chlorophyll *f*, ferredoxin, PsaF, PsaJ, photosynthesis, cyanobacteria, Chl, chlorophyll, CTF, contrast transfer function, β-DM, *n*-dodecyl-β-D-maltoside, ESP, electrostatic potential, FaRLiP, far-red light photoacclimation, Fd, ferredoxin, *Fischerella* 7521, *Fischerella thermalis* PCC 7521, FRL, far-red light, FRL-PSI, far-red light-acclimated PSI, PG, phosphatidylglycerol, PIB, photosystem isolation buffer, PDB, Protein Data Bank, PSI, photosystem I, *Synechococcus* 7335, *Synechococcus* sp. PCC 7335, WL, white light

## Abstract

Far-red light photoacclimation exhibited by some cyanobacteria allows these organisms to use the far-red region of the solar spectrum (700–800 nm) for photosynthesis. Part of this process includes the replacement of six photosystem I (PSI) subunits with isoforms that confer the binding of chlorophyll (Chl) *f* molecules that absorb far-red light (FRL). However, the exact sites at which Chl *f* molecules are bound are still challenging to determine. To aid in the identification of Chl *f*-binding sites, we solved the cryo-EM structure of PSI from far-red light-acclimated cells of the cyanobacterium *Synechococcus* sp. PCC 7335. We identified six sites that bind Chl *f* with high specificity and three additional sites that are likely to bind Chl *f* at lower specificity. All of these binding sites are in the core-antenna regions of PSI, and Chl *f* was not observed among the electron transfer cofactors. This structural analysis also reveals both conserved and nonconserved Chl *f*-binding sites, the latter of which exemplify the diversity in FRL-PSI among species. We found that the FRL–PSI structure also contains a bound soluble ferredoxin, PetF1, at low occupancy, which suggests that ferredoxin binds less transiently than expected according to the canonical view of ferredoxin-binding to facilitate electron transfer. We suggest that this may result from structural changes in FRL-PSI that occur specifically during FRL photoacclimation.

Photosystem I (PSI) is a plastocyanin:ferredoxin photooxidoreductase that is essential for the light reactions of oxygenic photosynthesis (1, 2). This multi-subunit, pigment–protein complex uses light energy to transfer electrons across the thylakoid membrane. In cyanobacteria, PSI is frequently trimeric with molecular mass ∼1.1 MDa and comprises 11 or 12 subunits per monomer (3). Each PSI monomer comprises a central core, where the electron transfer cofactors are found, surrounded by many antenna pigments, chlorophyll (Chl), and carotenoid molecules, which are involved in light absorption, energy transfer, and photoprotection. Upon light absorption, excitation energy is transferred through the antenna cofactors and to the electron transfer chain where charge separation then occurs. This initiates electron transfer through one of the two active electron transfer chain branches (4, 5). Specifically, from the lumenal to the stromal side of PSI, the electron transfer chain includes a pair of Chls called P_700_; two Chls per branch called A_−1_ and A_0_; a phylloquinone in each branch called A_1_; a [4Fe-4S] cluster called F_X_ where both branches converge; and two [4Fe-4S] clusters called F_A_ and F_B_. On the stromal side of PSI, a soluble [2Fe-2S] ferredoxin (Fd) or flavodoxin is reduced by the electron transfer chain, and on the lumenal side, photooxidized P_700_ is reduced by plastocyanin or cytochrome *c*_6_. In this way, Fd reduction by PSI provides reducing equivalents to fuel metabolism and carbon dioxide fixation by the Calvin–Benson–Bassham cycle. The binding of Fd to PSI in cyanobacteria has been studied extensively using biochemical approaches (6, 7, 8, 9), but the structural requirements for the reduction of Fd remain somewhat obscure because of the possibility of multiple binding conformations (10).

Far-red light photoacclimation (FaRLiP) is a physiological phenomenon that allows some terrestrial cyanobacteria to use the far-red region of the solar spectrum that is prevalent in shaded environments for photoexcitation (11, 12, 13). When cyanobacteria capable of FaRLiP are grown in far-red light (FRL), most of the core subunits of PSI are replaced with FRL-specific isoforms. Specifically, the core subunits PsaA, PsaB, PsaF, PsaI, PsaJ, and PsaL are replaced with FRL-specific isoforms PsaA2, PsaB2, PsaF2, PsaI2, PsaJ2, and PsaL2, respectively (11, 14, 15, 16). In addition, when grown in white light (WL), all Chls found in PSI are Chl *a*, but when grown in FRL, ∼10% of the Chls are replaced by Chl *f* in the PSI complexes of FaRLiP-capable cyanobacteria (11). Thus, it appears that the FRL-specific subunit isoforms may confer selectivity for Chl *f* binding thereby enabling their enhanced functionality. Understanding FaRLiP, especially the insertion and function of Chl *f* molecules into the photosystems, has become a major interest to understand better the diversity of acclimation mechanisms for photosynthesis in nature and to harness the understanding of these processes to derive design principles for tuning light absorption in crop plants (17, 18). To this end, various groups have used spectroscopic techniques and cryo-EM in attempts to identify the binding sites of Chl *f* in FRL-acclimated PSI (FRL-PSI) (14, 16, 19, 20). Cryo-EM structures of FRL-PSI from *Fischerella thermalis* PCC 7521 (*Fischerella* 7521) (14, 15) and *Halomicronema hongdechloris* (16) have been reported, but identifying the Chl *f* sites has been challenging because of the minor chemical difference (Chl *a* has a methyl moiety at the C2 position, whereas Chl *f* has a formyl moiety at the C2 position.), so ambiguity still exists in the exact sites (15, 21).

To provide further insight into structural and functional relationships of FRL-PSI, we used cryo-EM to solve the molecular structure of a third example from the marine, terrestrial, and FaRLiP-capable cyanobacterium, *Synechococcus* sp. PCC 7335 (*Synechococcus* 7335) (12). The *Synechococcus* 7335 FRL–PSI complex contains all 11 expected subunits and lacks the PsaX subunit of some PSI complexes, which influences the peripheral Chl arrangement. PsaF2, PsaJ2, and some nearby pigments bound in their vicinity are found at low occupancy, which helps to explain previous biochemical and biophysical observations of PSI complexes lacking analogous subunits. Based on a quantitative assessment of the cryo-EM map, consideration of sequence variation between WL- and FRL-subunit isoforms, and the identification of previously determined characteristics of Chl *f*-binding sites, we assigned six high specificity Chl *f*-binding sites and three low specificity Chl *f*-binding sites per *Synechococcus* 7335 FRL–PSI monomer. The Chl *f* analysis reveals the important aspects of Chl *f* binding in FRL-PSI including species-specific variation and specificity for binding Chl types in different sites. In addition, and surprisingly, Fd is bound at low occupancy to the stromal ridge of PSI formed by PsaC, PsaD, and PsaE, even though Fd was not added to the sample, suggesting that Fd-binding is less transient than in canonical models. This study of *Synechococcus* 7335 FRL-PSI reveals important new characteristics of FaRLiP, subunit variation, and Fd binding in cyanobacteria.

## Results

### Overall structure

**Figure 1:**
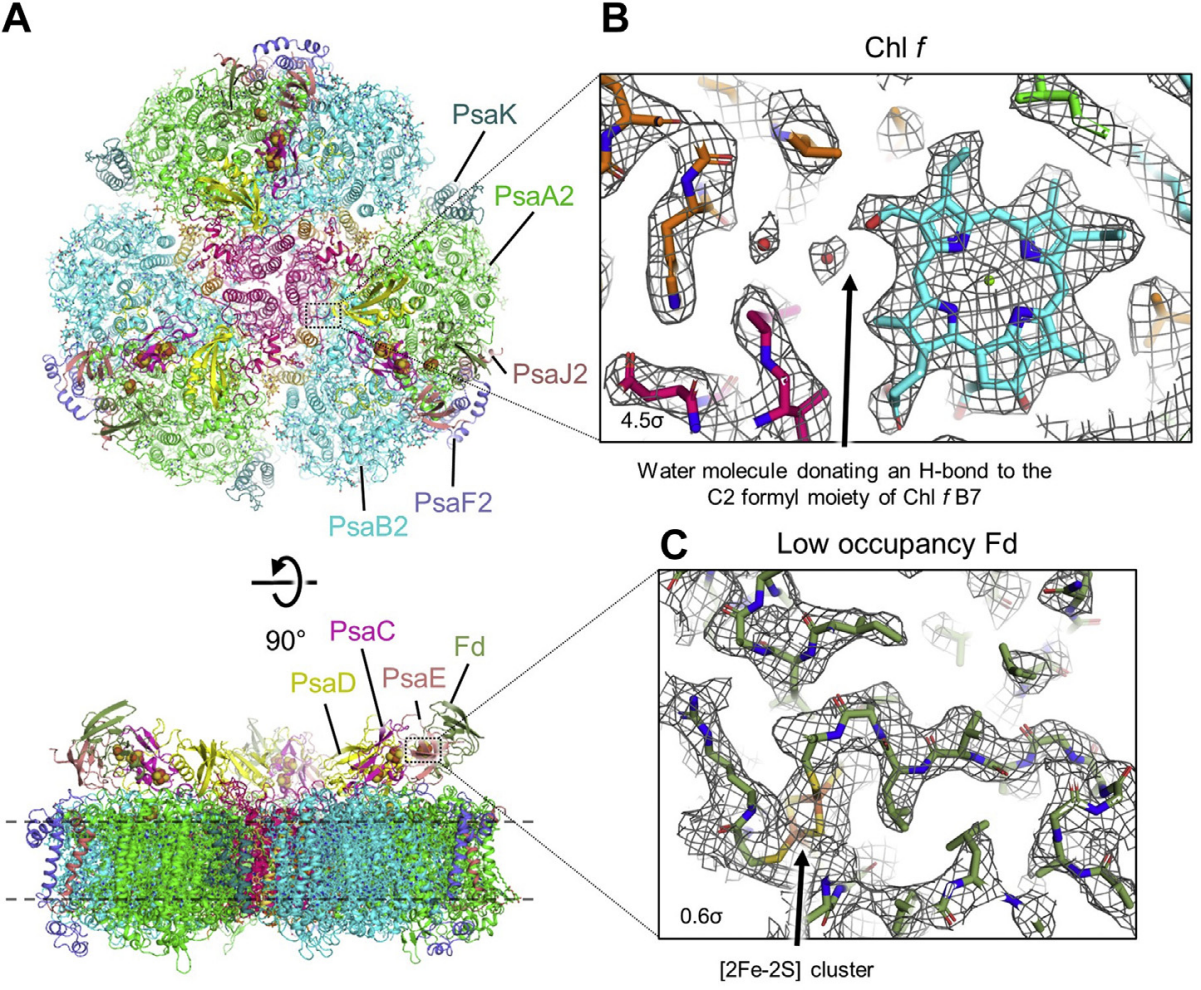
**Overview of the *Synechococcus* 7335 FRL-PSI structure and example cryo-EM map regions.***A*, stromal view (*top*) and membrane plane view (*bottom*) of the complete model are shown. In the *bottom* panel, the intermembrane region is denoted by *dashed lines*. The subunits are colored individually and notable subunits focused upon in the main text are labeled. The *dotted boxes* correspond generally to the regions shown in (*B*) and (*C*). *B*, view of the sharpened cryo-EM map and model corresponding to Chl B7 that is occupied by Chl *f*. Note that Chl and carotenoid site nomenclature used herein is based on those first introduced in Jordan *et al*. (3). The C2-formyl substituent is in a network of FRL-specific H-bonding. Some high occupancy water molecules can be observed in the map. *C*, view of the unsharpened cryo-EM map and model corresponding to low occupancy Fd. Despite its visualization at very low contour, the [2Fe-2S] cluster and many sidechains are visible in the cryo-EM map. Chl, chlorophyll; Fd, ferredoxin; FRL, far-red light; PSI, photosystem I.

FRL-PSI was isolated from FRL-acclimated *Synechococcus* 7335 cells and plunge-frozen for cryo-EM, as described in Experimental procedures. The cryo-EM data processing workflow is shown in Figure S1, and cryo-EM data statistics are presented in Table S1. The cryo-EM map, which is a distribution map of the electrostatic potential (ESP) of a molecule, exhibits a global resolution of 2.91 Å (Fig. S2) with local resolutions ranging from ∼2.8 to 3.2 Å (Fig. S3). An overview of the model built from the map and representative map regions are shown in Figure 1. Each monomer of the trimeric FRL-PSI model built from this cryo-EM-generated ESP map contained the following subunits: PsaA2, PsaB2, PsaC, PsaD, PsaE, PsaF2, PsaI2, PsaJ2, PsaK, PsaL2, and PsaM. Note that the ESP signals corresponding to subunits PsaF2 and PsaJ2 and a bound Fd, are observed at low occupancy as discussed below. Each monomer of the trimeric *Synechococcus* 7335 FRL-PSI coordinates 92 Chls, two phylloquinones, three [4Fe-4S] clusters, 19 β-carotenes, seven diacyl lipids, one Cl^−^ ion, one Ca^2+^ ion, and 13 *n*-dodecyl-β-D-maltosides (β-DM). The bound soluble Fd in addition coordinates one [2Fe-2S] cluster.

To model the Fd, we identified Fd sequences in *Synechococcus* 7335 with blastp (22) using the query sequence of Fd from *Thermosynechococcus elongatus* (National Center for Biotechnology Information code WP_011056851.1). Six Fd sequences were identified, and the one whose sequence corresponded to the cryo-EM map was used for modeling (Fig. S4). This sequence, corresponding to PetF1, was in addition confirmed by tryptic peptide fingerprinting of the purified FRL-PSI sample by tandem mass spectrometry (data not shown). Although it is present at low occupancy, nearly the entire main chain and the [2Fe-2S] cluster of Fd could be modeled, and only a few of the most peripheral sidechains were removed because of the absence of any corresponding ESP signal. The ESP corresponding to PsaF2 and PsaJ2 was also weak, and their locations overlap with that of the detergent micelle (Fig. S5). Despite this ESP convolution, the majority of PsaF2 and PsaJ2 could still be modeled with only some of the most peripheral sidechains being trimmed because of the absence of corresponding ESP. The low occupancy of PsaF2 and PsaJ2 observed in the cryo-EM map is consistent with SDS-PAGE results. PsaF2 exhibited much lower staining intensity than the similarly sized PsaD subunit (Fig. S6).

Determining the occupancy of PsaF2, PsaJ2, and Fd is challenging because the scaling in different regions of a cryo-EM map cannot easily account for both differences in occupancy and local resolution; however, ESP corresponding to protein secondary structure is clearly observed for peripheral regions of the core subunits PsaA2 and PsaB2, where B-factors are probably similar at ∼8.0 σ. The secondary structure for PsaF2 and PsaJ2 is observable at ∼2.5 σ and for Fd is clear at ∼0.8 σ. Thus, if the core subunits are assumed to be present in ∼100% occupancy, we estimate that the occupancies for PsaF2 and PsaJ2 are ∼30% and for Fd is ∼10%. In addition to these subunits, some nearby Chls associated with PsaA2 and PsaB2 appear to have low occupancy (Fig. S7), probably connected to the partial loss of PsaF2 and PsaJ2. The tetrapyrrole rings of most Chls in the structure are apparent at ∼8 σ, whereas the tetrapyrrole rings of four Chls near PsaF2 and PsaJ2 are not apparent until ∼4 σ, suggesting ∼50% occupancy of these sites. Specifically, these are Chl sites A1, A2, A39, and B30. The β-carotene in site B14 also appears to have low occupancy (Fig. S7*B*).

The FRL-PSI structure from *Synechococcus* 7335 allows for a more robust comparison of multiple FRL-PSI structures with PSI from both FaRLiP and non-FaRLiP strains. To achieve this, we created C_α_ superpositions of subunits from *Synechococcus* 7335 FRL-PSI, *H. hongdechloris* FRL-PSI (16), *Fischerella* 7521 FRL-PSI (14, 15), *H. hongdechloris* WL-PSI (16), *T. elongatus* PSI (3), *Synechocystis* 6803 PSI (23), and *Anabaena* sp. PCC 7120 PSI (24) (Fig. S8), and we calculated the sequence identity between their corresponding subunits (Fig. S9). Based on the superpositions (Fig. S8), the FRL-specific subunits are generally more similar to each other than they are with WL-PSI subunits or subunits of PSI from non-FaRLiP strains. This is consistent with higher sequence identity between FRL-specific subunits (Fig. S9). An exception to the high degree of FRL-PSI structural similarity is PsaK (Fig. S8), whose sequence comparison exhibits low sequence identity in all the organisms (Fig. S9). Unlike many PSI structures, nearly all of PsaK could be modeled in the *Synechococcus* 7335 FRL-PSI structure.

The *Synechococcus* 7335 FRL-PSI structure is more similar to the previously solved FRL-PSI structure from *H. hongdechloris* than other PSI structures (Fig. S8)*,* despite sharing similar sequence identity with FRL-PSI from both *H. hongdechloris* and *Fischerella* 7521. The similarity shared between FRL-PSI from *Synechococcus* 7335 and *H. hongdechloris* is consistent with the close phylogenetic relationship between these two strains, based on their 16S rRNA sequences that are closely related to many *Leptolyngbya* spp. (25, 26). PsaF2 and PsaJ2 are bound in the *Synechococcus* 7335 FRL-PSI structure similarly to those in *Fischerella* 7521 FRL-PSI, exhibiting RMSDs of 0.843 and 0.568 Å, respectively. PsaF2 and PsaJ2 were not modeled in the FRL-PSI structure from *H. hongdechloris* so their RMSDs could not be calculated.

Interestingly, the *H. hongdechloris* PSI (16) subunits that are not FRL-specific (*e.g.*, the stromal subunits PsaC, PsaD, and PsaE) exhibit different RMSDs with the analogous subunits from other PSI structures when they are within the FRL–PSI complex compared with the WL-PSI complex. For example, PsaE is not substituted during FaRLiP, and thus has the same sequence regardless of light conditions during cell growth, yet the RMSD of PsaE from the *H. hongdechloris* FRL–PSI complex compared with PsaE from the *Synechococcus* 7335 FRL-PSI complex is 0.289 Å and the RMSD of PsaE from the *H. hongdechloris* WL-PSI complex compared with PsaE from *Synechococcus* 7335 FRL-PSI complex is 0.555 Å. This suggests that even the PSI subunits that are not exchanged with different isoforms during FaRLiP may be slightly altered in their structure when found in FRL-PSI compared with WL-PSI.

We were also able to resolve a peripheral loop region of PsaB2, called loop D, and an associated Chl, both of which were missing in FRL-PSI from *H. hongdechloris* (16) (Fig. S10). This Chl site is called B40 and its axial ligand is the backbone carbonyl oxygen of PsaB2-Ile318 found in the newly modeled loop D. In *T. elongatus* (3) and *Fischerella* 7521 (14, 15) that have the *psaX* gene (which is lacking in *Leptolyngbya*-like strains such as *Synechococcus* 7335 and *H. hongdechloris*), the Chl B40 binding site corresponds to that for the stromal half of the single transmembrane helix of subunit PsaX found in those PSI structures. In those two cases, a phosphatidylglycerol (PG), called PG4, is bound and it is adjacent to the Chl dimer in sites B18 and B19 that is found in all PSI structures to date. PSI structures from the cyanobacteria *Synechocystis* sp. PCC 6803 (23) and *Synechococcus* sp. PCC 7942 (27) as well as plant (28, 29, 30) and algal (31, 32, 33, 34) PSI structures lack PsaX, but all bind PG4 in a similar location. However, in those complexes, the phosphate headgroup of PG4 provides the axial ligand to another Chl, also called Chl B40, that joins the B18/19 dimer to form a Chl trimer, B18/19/40. In FRL-PSI from *Synechococcus* 7335, Chl B40 is not in an orientation that would facilitate extension of the Chl B18/19 dimer to form a trimer; moreover, this Chl is coordinated differently, and thus it likely possesses different absorbance characteristics. In *T. elongatus* and *Synechocystis* sp. PCC 6803, IsiA-PSI cryo-EM structures have shown that the Chl B18/19 dimer or B18/19/40 trimer are among the closest Chls to the IsiA subunits and are very likely involved in energy transfer from IsiA to the Chls of the core (27, 35). Similarly, in plants and algae that also exhibit the B18/19/40 Chl trimer, the peripheral light-harvesting complex subunits are nearby, implicating the Chl trimer in energy transfer (28). The arrangement of B40 in FRL-PSI from *Synechococcus* 7335 is unique compared with other PSI structures, but this may reflect the diversity in antenna systems relative to the core, which is in agreement with low sequence similarity in this region of PsaB(2) (Fig. S10). It is also consistent with another unique orientation of B40 observed recently in a PSI structure from the high-light-tolerant cyanobacterium *Cyanobacterium aponinum* (36).

An alignment of PsaB sequences in the region of the Chl B40 binding site contains a conserved VEGP motif that appears to cooccur with PsaX (Fig. S10). It is unclear from the structures why these residues correlate with the presence of PsaX, but their proximity to PG4 suggest that these residues may be involved in binding PG4 and stabilizing the binding of PsaX. To test whether PsaX was commonly found when the VEGP motif is present in PsaB, we used the National Center for Biotechnology Information blastp (22) tool to identify other organisms that have annotated PsaX genes and analyzed their corresponding PsaB sequences (Table S2). Although the Val and Pro residues show some variation, the Glu and Gly residues are absolutely conserved in all these PsaB sequences. Thus, the presence of the conserved Glu-Gly motif in loop D of PsaB sequences may be used in future studies to predict PsaX-binding in organisms without accompanying PSI molecular structures or having incompletely annotated genomes.

### Identification of binding sites for Chl *f*

**Figure 2:**
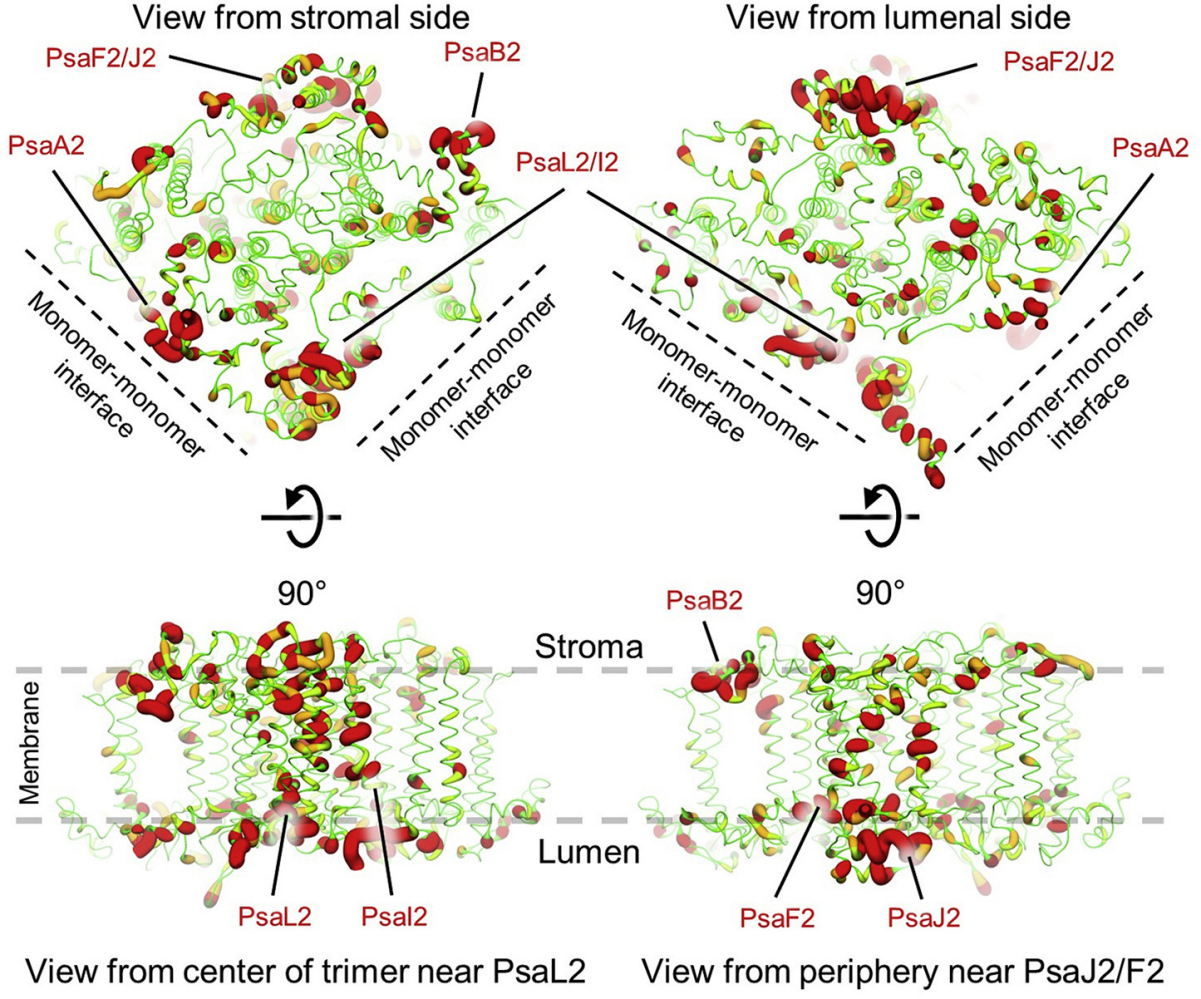
**Structure-based view of sequence similarity between FRL- and WL-specific sequences.** For each FRL-specific subunit, sequence alignments were created with its WL-specific sequence using Clustal Omega (39). The sequence alignments were used to calculate sequence conservation in UCSF Chimera (40) and coordinate files were colored such that *large red ribbons* correspond to low-sequence conservation and *thin green ribbons* correspond to high-sequence conservation. Only a single monomer of the trimeric complex is shown. Four views are shown as labeled and the regions of especially low similarity are labeled. FRL, far-red light; WL, white light.

Based on pigment analysis (37, 38) (see Experimental procedures) and deconvolution of low-temperature absorbance spectra (20), eight Chl *f* molecules are expected per *Synechococcus* 7335 FRL–PSI monomer. To gain insight into where Chl *f* might bind in the *Synechococcus* 7335 FRL-PSI structure, we mapped the sequence similarity between FRL and WL sequences to the FRL-specific subunits of the cryo-EM structure, which is shown in Figure 2. Because the incorporation of FRL-specific subunits is associated with the binding of Chl *f*, the regions of low sequence similarity are likely to be associated with nearby Chl *f* binding. The structure-based view of sequence similarity resembles that observed previously when the same procedure was carried out for *Fischerella* 7521 FRL-PSI (14). In both organisms, especially low sequence similarity is present: (A) in PsaA2, near the monomer-monomer interface, (B) near the center of the trimer where PsaL2 and PsaI2 are located, and (C) near the peripheral PsaF2 and PsaJ2 subunits, especially on the lumenal side. Unlike what was observed for *Fischerella* 7521, low similarity is also observed at the periphery of the complex on the stromal side of PsaB2 near the binding site for Chl B40 and where PsaX is found in some other cyanobacteria including *Fischerella* 7521. To improve the robustness of our sequence comparisons, we also prepared sequence alignments of FRL-specific sequences corresponding to the subunits in the three FRL-PSI structures from *Synechococcus* 7335, *H. hongdechloris*, and *Fischerella* 7521, their WL-specific sequences, and the corresponding subunit sequences from cyanobacterial strains that do not perform FaRLiP (Fig. S11). The most substantial differences are consistent with the low sequence similarity regions shown in Figure 2.

**Figure 3:**
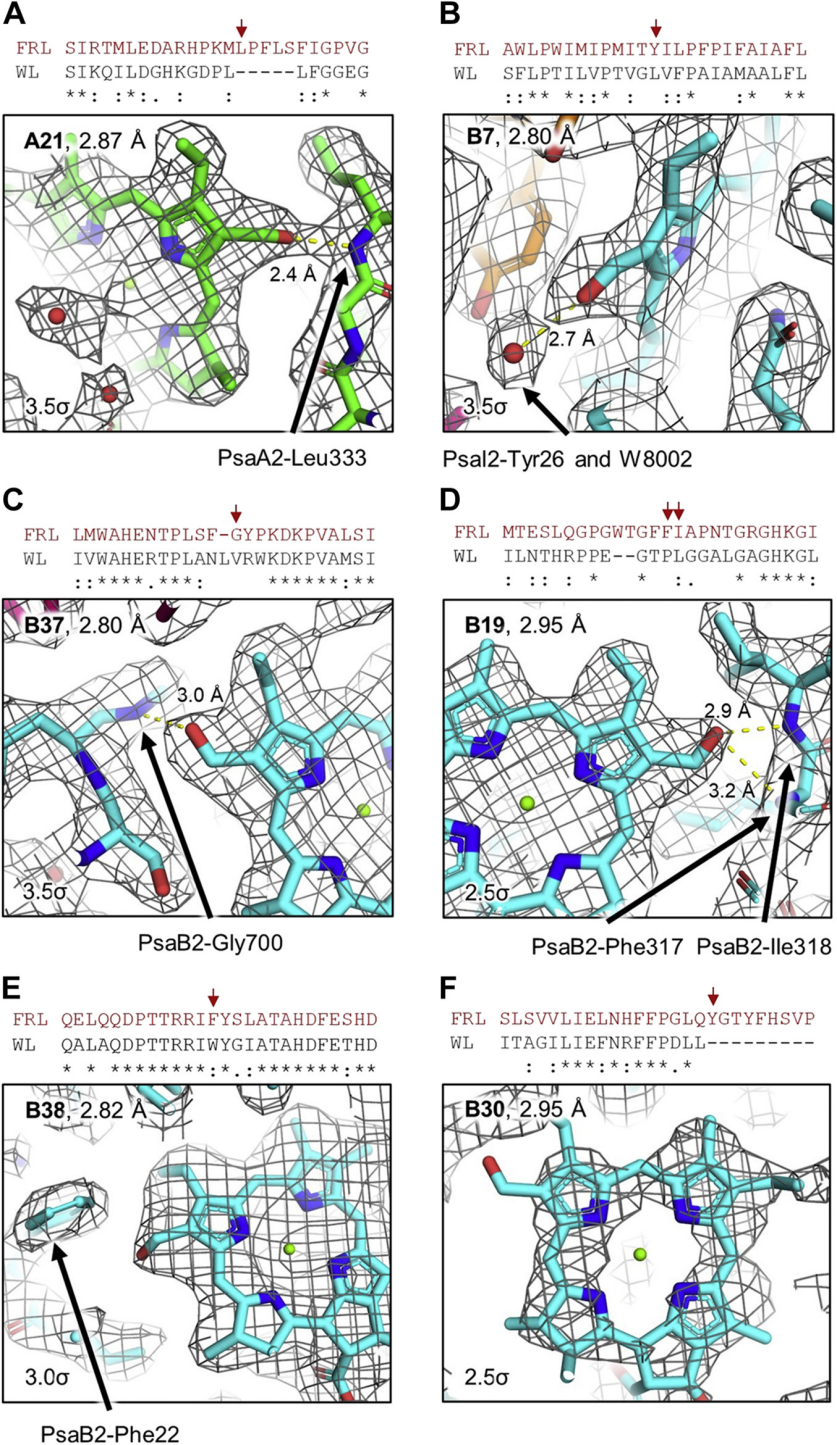
**Map and model for Chl *f* in sites A21, B7, B37, B19, B38, and B30.***A*–*F*, show the model within the ESP map for proposed Chl *f* sites A21, B7, B37, B19, B38, and B30, respectively. For each panel, a partial sequence alignment is shown of the region corresponding to directly observable or suggested H-bond donation to the formyl substituent, with *red arrows* denoting the amino acids involved. The local resolution for the central Mg of each Chl is indicated at the *top left* of each panel. For (*A*–*D*), the structural features involved in possible H-bond donation for the C2 formyl moiety are labeled. For panel (*E*), a PsaB2-Trp sidechain present in WL-PSI is replaced by the labeled Phe in FRL-PSI that is suggested to allow space for an H-bond from an unresolved water molecule to the formyl moiety of Chl *f* (20), as described in the text. In panel (*F*), low occupancy of the Chl in site B30 and PsaJ2 results in poorly defined map features. In *Fischerella* 7521, a PsaJ2-Tyr sidechain H-bonds to the formyl moiety of Chl *f* in site B30. The H-bonding Tyr is conserved in *Synechococcus* 7335; therefore, B30 is modeled as Chl *f* in *Synechococcus* 7335 based on homology. Chl, chlorophyll; ESP, electrostatic potential; FRL, far-red light; PSI, photosystem I; WL, white light.

To assess which Chl sites bind Chl *f* molecules, we visually inspected the cryo-EM map of each Chl site, searching the map for ESP that could correspond to a formyl substituent at the C2 position rather than a methyl substituent. The ESP maps of the Chls in sites A21, B19, B7, B37, and B38 all appear to have additional ESP that may correspond to a formyl substituent (Fig. 3). We also considered that Chl *f* binding sites typically include an H-bond donor to the C2 formyl moiety (14, 15, 21). In addition, ∼70% of Chl *a* molecules have a His sidechain as their axial ligand, whereas only one of the previously assigned Chl *f*-binding sites has a His axial ligand. This suggests that Chl *f* tends to disfavor axial ligation by His (15, 21). The C2 formyl H-bond donor and non-His axial ligation are often reflected as sequence differences between FRL and WL subunit isoforms (15, 21). We found that the Chls in sites A21, B19, B7, and B37 all have non-His axial ligands and obvious H-bond donors to the C2 formyl moiety, supporting the assignment of Chl *f* at these sites. The Chl in site B38 is also axially ligated by a non-His sidechain but does not have a clear H-bond donor to the formyl moiety. However, this site forms a Chl dimer with B37 that was proposed to explain spectroscopic evidence for a Chl *f* dimer (20). Furthermore, the structure and sequence alignment show that a WL-specific Trp residue is replaced with the smaller Phe residue in FRL, which may accommodate the insertion of a water molecule that H-bonds to the formyl substituent of Chl *f* at B38 and the backbone carbonyl O atom of PsaB2-Thr18, as described previously (20). Therefore, we think it likely that a water molecule H-bonds to the Chl *f* in site B38 but is unresolved because of resolution limitations. Together, this evidence suggests that Chl *f* binds at B38 despite limited resolution in our *Synechococcus* 7335 FRL-PSI map. Similarly, Chl B30 is also likely to bind Chl *f* but is not well resolved. In our *Synechococcus* 7335 FRL-PSI cryo-EM map, Chl B30 and the PsaJ2-Tyr sidechain that H-bonds to the C2 formyl moiety of B30 have low occupancy (Figs. S5 and S7), limiting our ability to directly distinguish the Chl type in that site. Although Chl B30 is axially coordinated by a His sidechain, it was confirmed to bind Chl *f* in *Fischerella* 7521 (15, 20). The same PsaJ2-Tyr sidechain that provides the H-bond to Chl *f* B30 in *Fischerella* 7521 is conserved in the PsaJ2 sequences from *Synechococcus* 7335 and *H. hongdechloris*, strongly suggesting the conservation of Chl *f* binding at B30 among FRL-acclimating species. We note that the sites B30 and B7 are related by the pseudo-C2 symmetry of the core, and the sites A21 and B19 are also symmetry related in this way. All four of those Chls are assigned as Chl *f,* as discussed further below.

We further investigated the Chl sites by quantitatively analyzing the ESP corresponding to the C2 substituent using the cone scan method developed previously (15) (Fig. S12 and Data S1). This method assists in distinguishing signal from noise in cryo-EM maps (21). In brief, a scan of the ESP for the C2 substituent is compared against a null distribution produced from comparable scans of methyl substituents at the C7 position. When the C2 substituent scan exceeds the null distribution, the null hypothesis is rejected with a corresponding *p*-value of <0.002. The alternative hypothesis is then accepted, signifying that the C2 substituent is dissimilar from the ESP corresponding to a methyl moiety, suggesting the presence of a formyl moiety. The Chls in sites A21, B7, and B37 all exhibit ESP cone scans for the C2 substituent that exceed the null distribution, which rejects the null hypothesis, consistent with the presence of Chl *f* in agreement with the initial visual inspection (Fig. 3). Based on the visual inspection of ESP corresponding to the C2 moieties, chemical environment features, sequence homology to other FRL-PSI structures, and cone scan analysis, we modeled Chl *f* molecules in six sites: A21, B19, B7, B30, B37, and B38.

### Ferredoxin-binding location

**Figure 4:**
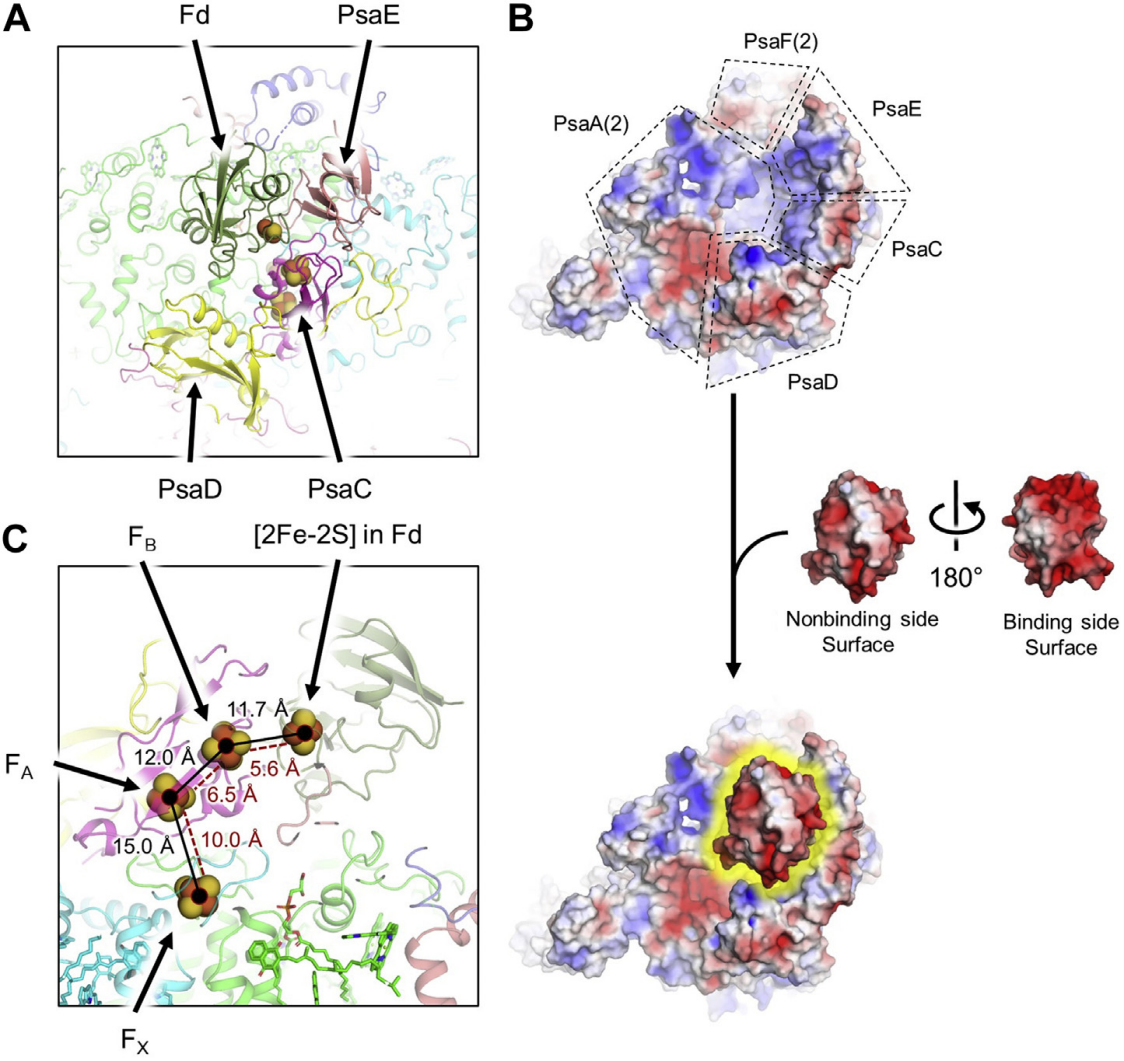
**Ferredoxin-binding and Fe-S cluster distances in *Synechococcus* 7335 FRL-PSI.***A*, stromal view of the stromal ridge where Fd is bound. *B*, surface electrostatics maps from the cryo-EM models showing the core without Fd (*top*), the Fd alone (*middle*), and the binding location of the Fd (*bottom*). *C*, side view of the stromal ridge with Fd bound. The cartoons are made slightly transparent, and the Fe-S clusters are shown in *sphere* representation. Both center-to-center distances (*solid black lines* and *font color*) and edge-to-edge distances (*dashed red lines* and *font color*) are shown. Edge-to-edge distances include the S atoms from coordinating Cys sidechains. Fd, ferredoxin; FRL, far-red light; PSI, photosystem I.

Although Fd was not added during any experimental procedure, Fd was identified in the cryo-EM map of the FRL–PSI complex. Similar to previous PSI structures with electron acceptors bound (27, 41, 42), Fd is located in a position surrounded by PsaA2 and the stromal ridge formed by PsaC, PsaD, and PsaE (Fig. 4*A*). The binding location and interactions are essentially identical to those observed in the X-ray crystal structure of *T. elongatus* PSI with Fd bound (41) (Protein Data Bank code [PDB] 5ZF0, Fig. S13), which is consistent with high sequence identity of the stromal ridge subunits and low RMSDs of those subunits and of the respective Fds (Table S3). The binding of Fd to PSI is thought to be substantially driven by electrostatic interactions (43, 44, 45); therefore, we calculated the electrostatic surfaces (46) for the structure without Fd bound and of the Fd alone. The electrostatic surface of the core clearly shows a primarily positively charged cavity, and the binding side surface of Fd is primarily negatively charged (Fig. 4*B*). The X-ray crystal structure of *T. elongatus* PSI with Fd bound was reported at relatively low resolution without trimeric symmetry, and it was determined that the three Fd-binding sites bound Fd asymmetrically (41). We attempted to reconstruct the cryo-EM map without C3 symmetry, but no differences in the Fd-binding sites were observed, and this reconstruction resulted in a decreased global resolution to 2.96 Å. Although this result suggests that Fd is not bound asymmetrically, it is possible that the ESP signal corresponding to Fd may be too low for the orientational alignment software to detect asymmetry between monomers. Therefore, asymmetric Fd binding cannot be ruled out. We measured the distances between the Fe-S clusters (Fig. 4*C*) and found that they most closely resemble the Fd from the *T. elongatus* PSI-Fd X-ray crystal structure that exhibited the shortest F_B_ to [2Fe-2S] distance (41) (Table S4).

## Discussion

The similarity between *Synechococcus* 7335 FRL-PSI and *H. hongdechloris* FRL-PSI (Figs. S8 and S9) was anticipated because both cyanobacteria are closely related to *Leptolynbgya*-like strains isolated from similar environments (25, 26). However, the FRL-PSI structural model from *H. hongdechloris* lacked PsaF2, PsaJ2, and Fd which are present in low occupancy in *Synechococcus* 7335 FRL-PSI. When the structure of *H. hongdechloris* FRL-PSI was initially described, the authors suggested that PsaF2 and PsaJ2 may have been lost during the plunge-freezing step for cryo-EM sample preparation (16). For FRL-PSI from *Synechococcus* 7335, SDS-PAGE and tryptic peptide fingerprinting demonstrated that these subunits, and in addition Fd, were already depleted before plunge freezing. It seems possible that PsaF2 is present at substoichiometric levels in the FRL-PSI preparations from *H. hongdechloris* based upon SDS-PAGE band intensities (16). This may suggest that PsaF2 and PsaJ2 are bound more loosely in FRL-PSI from *Leptolyngbya*-like strains than some other cyanobacteria. It is presently unclear whether this difference in PsaF2 and PsaJ2 binding holds any biological significance, but we note that the structure of WL-PSI from *H. hongdechloris* also lacked PsaF and PsaJ (16), so the loose binding does not seem to correlate with changes introduced during FaRLiP.

The *psaF* and *psaJ* genes are cotranscribed (47), and loss of PsaJ from PSI always accompanies removal of PsaF (48), which is consistent with our observation that they are present at similarly low occupancies. In previous work, the removal of PsaF and PsaJ from *Synechococcus* sp. PCC 7002 cells by deletion or interruption of the genes encoding these subunits was accompanied by photoaccumulation of A_0_^−^, which was explained by the formation of a water channel that allowed for protonation and subsequent double reduction, and thus inactivation, of the A_1_ semiquinone radical (48). In the absence of PsaF2, PsaJ2, the Chls in sites A1, A2, and A38, and β-carotene 14 (Fig. S7), the A_1A_ site is relatively open to solvent (Fig. S14). We cannot be certain how the environment of A_1A_ changes as a result of the loss of these structural features, but such substantial changes so close to A_1A_ could result in the formation of a water channel as proposed (48), especially near the stromal soluble domains of PsaF2 and PsaJ2. The absence of PsaF also resulted in a blue shift in the fluorescence emission maximum and decrease in the long wavelength fluorescence emission at 77 K (49). When present, the Chls in sites A1 and A2 appear to form a Chl dimer that could contribute to longer wavelength fluorescence emission. Upon their loss, which appears to be concomitant with the loss of PsaF(2) and PsaJ(2), this possible lower-energy Chl dimer is removed, which may cause an overall hypsochromic shift in the fluorescence emission spectrum. Furthermore, the Chl in site A39 is observed at low occupancy in the *Synechococcus* 7335 FRL-PSI structure which appears to form a Chl dimer with the Chl in site A38 (Fig. S7). Therefore, the removal of PsaF2 and PsaJ2 probably causes this Chl A38/39 dimer that could contribute to lower energy absorbance to monomerize, leaving only Chl A38 which may also shift its absorbance to higher energy.

Our analysis of the Chl sites in *Synechococcus* 7335 FRL-PSI suggest that at least six sites bind Chl *f*: A21, B19, B7, B30, B37, and B38. All of these sites except B19 have been suggested previously (14, 15). The fact that some Chl sites that bind Chl *f* with high specificity are conserved across species suggests that these Chl *f* sites are especially important to achieve uphill energy transfer which has previously been shown to occur (38). For example, it appears that the Chl *f* molecules at sites B7 and B30, which are related by pseudo-C2 symmetry to the core and somewhat close (<15 Å) to the electron transfer chain (Fig. S15), are conserved among species; these Chl *f* molecules could serve as energy-transfer intermediates between the core antenna and the electron transfer chain of the reaction center. On the other hand, our identification of Chl *f* binding at site B19, which is the symmetry mate to the Chl *f* at site A21, reveals some species variation in FRL-PSI. The peripheral location of Chl *f* at site B19 may have higher site energy among the Chl *f* molecules so that energy can be transferred through Chl *a* toward the electron transfer chain, and thus could correspond to one of the shorter-wavelength Chl *f* assignments in spectral deconvolution reported recently (20). This may also be the case for its symmetry mate, the Chl *f* at site A21. In WL, A21 is found in a Chl trimer, but in FRL, it is instead found in a dimeric configuration. This difference in arrangements was suggested previously to increase its site energy when Chl *f* is inserted, thus avoiding the creation of a low energy well (14).

The Chl *f* found in site B19 of *Synechococcus* 7335 FRL-PSI is close to where PsaX is found in *Fischerella* 7521 FRL-PSI, and the same loop D is involved in its binding, as discussed above (Fig. S10). This site also correlates to the region of low sequence similarity observed near the peripheral region of PsaB2 (Fig. 2). Although B19 was present in the FRL-PSI structure from *H. hongdechloris*, it was located in a poorly resolved region of the map, which is exemplified by the lack of the nearby Chl B40 and PsaB2 loop D in that structure (16). In the *Synechococcus* 7335 FRL-PSI structure, loop D is well resolved and clearly donates an H-bond to the C2 formyl substituent of Chl B19 (Fig. 3). The Phe and Ile residues whose backbone amide N atoms are within H-bonding distance of the B19 formyl moiety are conserved in PsaB2 from *H. hongdechloris* (Fig. S11), confidently suggesting that the *H. hongdechloris* FRL–PSI complex also binds Chl *f* at site B19. Chl B19 is also present in *Fischerella* 7521 FRL-PSI, but the coordinating loop D greatly differs, having interactions with the PsaX subunit not found in the other two strains. Furthermore, site B19 in *Fischerella* 7521 lacks any evidence for possible C2 H-bond donors to the formyl moiety if B19 binds Chl *f* (Fig. S16). This does not necessarily exclude Chl *f* from binding to site B19 in *Fischerella* 7521 where the axial ligand is unclear and thus, is probably a water molecule, a common Chl *f* axial ligand. If it did, Chl *f* would probably bind with much less specificity in the absence of an H-bond donor to the formyl moiety, which may result in a mixture of Chl *a* and Chl *f* at this position in those complexes.

Thus, it appears that B19 binds Chl *f* with high specificity in *Synechococcus* 7335 and *H. hongdechloris* but not *Fischerella* 7521, which is probably linked to the presence or absence of the PsaX subunit. A species-specific Chl *f*-binding site was noted previously for the Chl in site A23, where a PsaA2-Gln sidechain near the C2 position of site A23 confers specificity for Chl *f* binding in *H. hongdechloris* FRL-PSI but not in *Fischerella* 7521 FRL-PSI, in which the residue is not conserved and instead has a Met at the same position (21). Like *Fischerella* 7521 FRL-PSI, *Synechococcus* 7335 FRL-PSI conserves the PsaA2-Met near the C2 position of Chl site A23, conferring specificity for binding Chl *a* rather than Chl *f*. Not all FaRLiP-capable cyanobacteria contain genes for PsaX, so it appears that there are at least four different types of FRL-PSI: complexes with or without Chl *f* at site A23, and complexes in which site B19 binds Chl *f* with high or low specificity. These observations pave the way for future phylogenetic analyses of FRL-PSI that link sequence observations based on molecular structures to the evolutionary history of Chl *f* binding.

**Figure 5:**
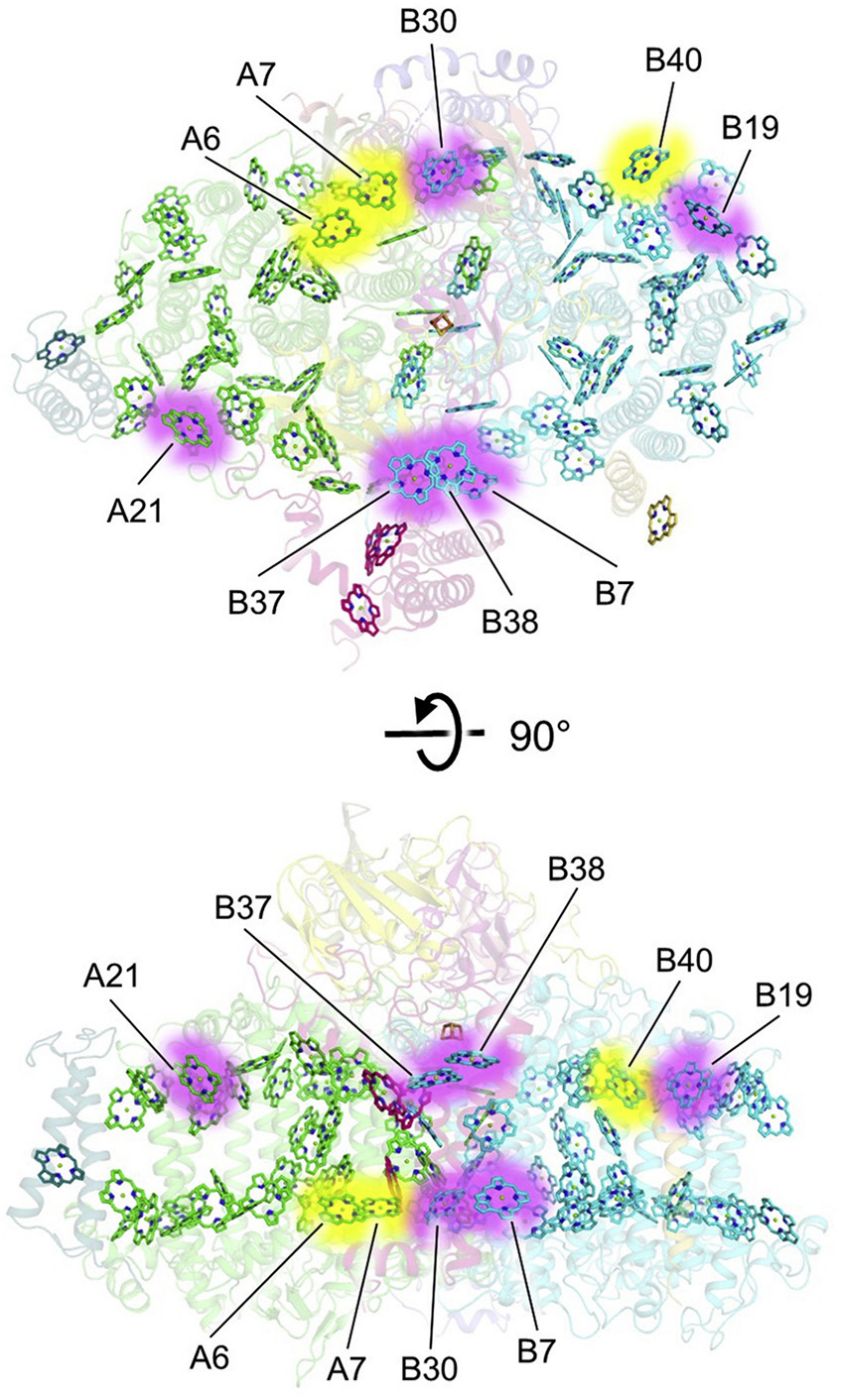
**Summary of Chl *f* sites.** A monomer of *Synechococcus* 7335 FRL-PSI is shown with transparent protein cartoons and the tetrapyrrole rings for all Chl sites. The Chl sites A21, B7, B19, B30, B37, and B38 are high-specificity Chl *f*-binding sites (*pink glow*). We suggest that Chl sites A6, A7, and B40 bind Chl *f* with lower specificity (*yellow glow*) or that H-bond donors to their C2 formyl moieties cannot be resolved because of their peripheral positions. Note that the Chl *f* molecules at sites B7 and B30 are related by pseudo-C2 symmetry, and the Chl *f* molecules at sites A21 and B19 are related by pseudo-C2 symmetry. Figure S15 also shows the Chl *f* positions relative to the electron transfer chain cofactors only. Chl, chlorophyll; FRL, far-red light; PSI, photosystem I.

A final note regarding Chl *f*-binding sites is that despite the expectation of ∼8 Chl *f* molecules per FRL-PSI monomer, direct evidence from the cryo-EM maps is only observed for fewer. Major contributing factors to this discrepancy are likely because of the limitations of cryo-EM as an experimental method for detecting Chl *f* and the nature of Chl *f* binding. Concerning the former, the partial negative charge of the formyl O atom of Chl *f* decreases its visibility in ESP maps produced by cryo-EM as a function of resolution, making resolution limitations a major factor in distinguishing Chl *f* molecules from Chl *a* (21). Concerning the latter, most of the unidentified Chl *f*-binding sites are probably bound near the periphery of the FRL–PSI complex, where the resolution is lower than for more stable central regions (Fig. S3). This latter point is exemplified by (A) the fact that the B19 site could not be observed in the *H. hongdechloris* FRL-PSI structure due to peripheral instability and (B) the C2 cone scan signal for Chl *f* at peripheral site B19 does not reject the null hypothesis in our cone scan analysis even though it is clearly Chl *f* by visual inspection of the map and contains nearby H-bond donors (Fig. 3). Furthermore, we think it is possible that some Chl *f*-binding sites may exhibit lower specificity, resulting in low occupancy in the ensemble cryo-EM map. Indeed, up to *ca.* four Chl *f* molecules can be bound per PSI monomer in *Synechococcus* sp. PCC 7002, a cyanobacterium incapable of FaRLiP (38, 50). Good candidates for sites that bind Chl *f* and Chl *a* promiscuously include A6, A7, and B40. None of these three Chls are coordinated by His sidechains; all lack obvious H-bond donors near the C2 position; none exhibit sterically hindering or repulsive interactions near the C2 position; and all are found in regions containing FRL-specific sequence differences compared to WL sequences. Figure 5 summarizes the Chl sites where we suggest that Chl *f* is bound with high specificity and in addition, shows other sites that are good candidates for Chl *f* binding with lower specificity. The positions of these sites relative to the electron transfer chain cofactors are in addition shown in Figure S15. The locations of these sites are consistent with the regions of FRL-specific sequence differences shown in Figure 2, which supports the hypothesis that FRL-subunit isoforms confer selectivity for the insertion of Chl *f* in FRL-PSI. All potential Chl *f*-binding sites occur at the periphery of the FRL-PSI core and are rather distant from the electron transfer chain cofactors. This is consistent with the observation that Chl *f* in FRL-PSI acts solely as an antenna pigment (51) and was added by evolution without disrupting the key structural elements required for efficient excitation energy transfer and electron transfer.

The presence of Fd, even at very low occupancy, was unexpected because we are unaware of any Fd-binding model that would predict retention of Fd bound to PSI complexes throughout sample preparation. This suggests that Fd binding is not always entirely transient during FaRLiP, but is at some point bound tightly to the stromal ridge, at least in this complex. This could correlate to the redox state of Fd, or perhaps another soluble Fd could be reduced by the one that we observe to be bound. Phycobilisomes, which notably undergo changes during FaRLiP (12), are also present on the stromal side of the membrane that could influence Fd-binding as well. *Synechococcus* 7335 cells grown in white, green, or red light produce hemidiscoidal phycobilisomes with tricylindrical cores with six peripheral rods, some of which bind ferredoxin:NADP^+^ reductase (52, 53, 54, 55). However, *Synechococcus* 7335 cells grown in FRL produce bicylindrical cores with no peripheral rods (54, 56). Therefore, ferredoxin:NADP^+^ reductase must be unbound in FRL and could diffuse to the Fd bound to PSI. It may also be that the compositional and structural changes induced by FaRLiP increase the binding affinity of PSI for Fd. Although the stromal ridge subunits are not substituted in FRL-PSI compared with WL-PSI, a piston mechanism involving PsaF and PsaJ, both of which are substituted by FRL-specific isoforms (PsaF2 and PsaJ2), was proposed previously to explain asymmetry in Fd-binding sites in cyanobacterial PSI (41). In addition, it has been shown in *Arabidopsis thaliana* that the loss of PsaF destabilizes the stromal ridge subunits indirectly *via* PsaA (57). Furthermore, as noted previously, loss of PsaF and PsaJ by mutation led to destabilization of PsaE binding (48). Thus, the key subunits that are known to influence Fd-binding are replaced by FRL-specific isoforms that could alter Fd binding. This is consistent with the observation that subunits that are not specific to FRL exhibit somewhat different structures in WL-PSI from *H. hongdechloris* compared with FRL-PSI from *H. hongdechloris* despite their identical sequence (Figs. S8 and S9). Therefore, future molecular structures of WL-PSI from *Synechococcus* 7335 and *Fischerella* 7521 are desirable in part to compare the non-FRL-specific subunits between WL- and FRL-complexes. If FaRLiP does influence the binding affinity of PSI for Fd, it could mean that shaded environments, where FaRLiP occurs, might favor tight Fd binding rather than fast reduction and release. This would be reasonable because fast reduction is important to avoid the production of reactive oxygen species such as superoxide, which is probably more prevalent in WL where irradiance levels are higher. Thus, future biochemical studies that aim to characterize Fd binding to FRL-PSI are desirable and could prove very informative. It would also be interesting to detect what fraction of PSI has Fd bound *in vivo* by biochemical or structural approaches.

## Experimental procedures

### Strain and growth conditions

*Synechococcus* 7335 was obtained from the Pasteur Culture Collection (www.pasteur.fr/en/pcc) (25). The cells were grown at room temperature (∼25 °C) in ASNIII medium and were sparged with 1% (v/v) CO_2_ in air (54, 58). Liquid maintenance cultures were grown in WL, provided by cool white fluorescent bulbs (∼45–50 μmol photons m^−2^ s^−1^). To grow the cells in FRL, the cultures were first acclimated to red light (∼35–40 μmol photons m^−2^ s^−1^), which was provided by using a red plastic filter, as previously described (59). The cultures grown in red light were diluted to about 0.2 OD_750_ to initiate the FRL cultures. FRL was provided by an LED panel with emission centered at 720 nm and/or by filtering halogen light with a combination of green and red plastic filters to provide FRL at ∼20 to 28 μmol photons m^−2^ s^−1^ (for details, see Gan *et al*. (11) and Shen *et al*. (59)). For complete acclimation to FRL, the cells were grown with stirring and sparging in FRL for 8 to 12 weeks. The cells were diluted and the medium was refreshed at 2-week intervals.

### Isolation of trimeric PSI complexes

PSI complexes were isolated from the cells grown in FRL, as previously described (14, 59). The cells were lysed by three passages through a chilled French pressure cell operated at 138 MPa. After the removal of cell debris, total membranes were prepared by ultracentrifugation (126,000*g*, 1 h); resuspended in photosystem isolation buffer (PIB), which is composed of 50 mM MES, pH = 6.5, 15 mM CaCl_2_, and 10 mM MgCl_2_; and solubilized at 4 °C for 1 h by the addition of β-DM to a final concentration of 1% (w/v). After the removal of large insoluble debris by centrifugation, the solubilized membranes were loaded onto 5 to 22% (w/v) sucrose gradients prepared with PIB containing 0.1% (w/v) β-DM. The gradients were centrifuged for ∼18 h at 108,000*g*. Green-colored, Chl-containing fractions containing trimeric FRL-PSI (38) were collected and dialyzed against PIB. After concentration using Millipore Centriprep 100 kDa Centrifugal Filtration Devices (EMD Millipore), the fractions containing trimeric FRL–PSI complexes were loaded onto 5 to 22% (w/v) sucrose gradients containing 0.03% β-DM and subjected to a second round of ultracentrifugation. The trimeric FRL–PSI complexes were collected from the sucrose gradients. Most of the sample was brought to 0.1% β-DM, concentrated to ∼3 mg Chl/ml, and prepared for cryo-EM, as described below. An aliquot of the purified trimeric FRL-PSI particles from the sucrose gradients was also retained for biochemical analyses which was dialyzed against PIB, concentrated, and resuspended in PIB containing 0.03% (w/v) β-DM and 5% (w/v) glycerol for storage at −80 °C until required.

### Analytical procedures

The purity of the isolated PSI complexes was assessed by SDS-PAGE and by tryptic peptide fingerprinting by mass spectrometry. SDS-PAGE was performed, as described previously (60). Samples for peptide analysis were prepared, as previously described (61). The methods for pigment analysis of the FRL–PSI complexes from *Synechococcus* 7335 were described previously (38, 59). Based on 92 total Chl molecules, the FRL–PSI complexes contained 8.05 Chl *f* and 84.0 Chl *a* molecules.

### Cryo-EM grid preparation

FRL-PSI was kept in the dark until just before it was plunge frozen at which point it was exposed to ∼30 s of low-fluorescent light. Using a Thermo Fisher Vitrobot system, 3 μl of FRL-PSI at ∼3 mg Chl ml^−1^ was applied to a holey-carbon C-flat 2/1 Cu 300-mesh electron microscopy grid (Electron Microscopy Sciences) that had been glow-discharged for 30 s at 25 mA. The grid was blotted immediately for 3 s, plunged into liquid ethane, and stored in liquid nitrogen for data collection. The Vitrobot system was set to 4 °C and 100% humidity for plunge freezing.

### Cryo-EM data collection

The grid was imaged on a Titan Krios G2 transmission electron microscope (Thermo Fisher/FEI) operated at 300 kV equipped with a Gatan K3 direct electron detector in super-resolution mode with a slit width of 20 eV. The defocus range was set to −1.5 to −2.5 μm, and the nominal magnification was 105,000×. The super-resolution pixel size was 0.413 Å. The dose rate was 12.4 e^−^ physical pixel^−1^ s^−1^. The total exposure time was 2.05 s per exposure with a total dose of 28.9 e^−^ (Å) ^−2^. SerialEM was used to collect 14,992 micrograph movies with 28 images per stack.

### Cryo-EM data processing

Data processing was performed using Relion 3.1 (62). The frames were corrected, aligned, and dose-weighted using MotionCor2 (63). Ctffind-4.1.13 (64) was used to estimate the contrast transfer function (CTF). An initial set of ∼2000 particles was selected manually, and their 2D classification was used as autopicking templates. Autopicking resulted in an initial selection of 2,504,601 particles. The Initial Model function was used to create an *ab initio* model used as a low-resolution reference for initial 3D classification. Further 2D and 3D classification resulted in a particle set containing 444,298 particles. CTF refinement, Bayesian Polishing, 3D classification, and selection of the particles based on the metadata CtfMaxResolution ≤5.0 led to a final particle set containing 286,672 particles. Reconstruction using C3 symmetry led to a global resolution of 2.91 Å based on the Gold-standard Fourier Shell Correlation (0.143) cutoff criterion (Fig. S2) (62, 65). A flowchart for data processing is shown in Figure S1.

### Model building

To generate an initial input model, homology models of all subunits were created using the template structure of FRL-PSI from *H*. *hongdechloris* (PDB 6KMX) (16) and its associated protein sequences for all subunits except PsaF2, PsaJ2, and ferredoxin which were not present in that structure. For PsaF2 and PsaJ2, the template model was the structure of FRL-PSI from *Fischerella* 7521 (PDB 7LX0) (14, 15) and for the ferredoxin, the template model was the structure of PSI-Fd from *T. elongatus* (PDB 5ZF0) (41). All homology modeling was created using SwissModel (66). Chl and carotenoid numbering are based on those reported in Jordan *et al*. (3). These components were fit into the ESP map using UCSF Chimera (40). Manual editing and automated water placement were performed in Coot (67), and automated refinement was performed using real_space_refine (68) in the Phenix software suite (69).

### Cone scans

Cone scans were performed, as described previously (15). Every Chl molecule was least-squares aligned onto an ideal reference Chl inside the cubic box using the first atom of the substituent plus three coplanar adjacent atoms where the substituent was located alongside the corresponding experimental map using the program suites CCP4 (70) and Rave (71). The map was inverted using Phenix (72). The rescaled experimental ESP values on each cone axis were extracted with an increment of 0.01 Å using direct Fourier summation of corresponding structure factors. The experimental values for the C2 and C7 axis were extracted every 5° at an expected bond length. These scans were binned by resolution of the central Mg with a cut-off of 2.91 Å. The methyl moiety null distribution for each bin was generated assuming a normal distribution at each sampled angle at the C7 position. With a significance level of 0.002, the null distribution is thus modeled by the equation *μ* + 3σ where *μ* is the distribution mean and σ is the distribution standard deviation.

## Data availability

Cryo-EM structures have been deposited in the Protein Data Bank and Electron Microscopy Data Bank with accession codes 7S3D and EMD-24821, respectively.

## Supporting information

This article contains supporting information (22, 27, 28, 29, 30, 31, 32, 33, 34, 39, 62).

## Conflict of interests

The authors declare that they have no conflicts of interest with the contents of this article.
