# Supplementary material for: Structure of a photosystem I-ferredoxin complex from a marine cyanobacterium provides insights into far-red light photoacclimation
Source: J Biol Chem. 2021 Nov 15;298(1):101408. doi: 10.1016/j.jbc.2021.101408 (PMC8689207; doi:10.1016/j.jbc.2021.101408)
Supplement: Figures S1–S16, Table S1–S4 and Data S1 [file mmc1.doc]

**Supporting Information for**

Structure of a photosystem I-ferredoxin complex from a marine cyanobacterium provides insights into far-red light photoacclimation

Christopher J. Gisriel^1,†^, David A. Flesher^2,†^, Gaozhong Shen^3^, Jimin Wang^2^, Ming-Yang Ho^4^, Gary W. Brudvig^1,2^,*, and Donald A. Bryant^3^,*

^1^Department of Chemistry, Yale University, New Haven, CT 06520, USA.

^2^Department of Molecular Biophysics and Biochemistry, Yale University, New Haven, CT 06520, USA.

^3^Department of Biochemistry and Molecular Biology, The Pennsylvania State University, University Park, PA 16802, USA.

^4^Department of Life Science, National Taiwan University, Taipei 10617, Taiwan.

^†^These authors contributed equally.

*To whom correspondence should be addressed: [dab14@psu.edu](mailto:dab14@psu.edu) and [gary.brudvig@yale.edu](mailto:gary.brudvig@yale.edu)

**Fig. S1.** Cryo-EM data processing workflow, example micrograph image, and example 2D class images.

**Fig. S2.** FSC curves for the *Synechococcus* 7335 FRL-PSI structure.

**Fig. S3.** Local resolution of the *Synechococcus* 7335 FRL-PSI cryo-EM map.

**Fig. S4.** Fd sequences identified in the blastp search of *S*. 7335.

**Fig. S5.** ESP for low-occupancy subunits PsaF2 and PsaJ2.

**Fig. S6.** SDS-PAGE of *Synechococcus* 7335 FRL-PSI.

**Fig. S7.** ESP for low occupancy pigment sites near PsaF2 and PsaJ2.

**Fig. S8.** Structural similarity of selected PSI subunits.

**Fig. S9.** Sequence identities of selected PSI subunits.

**Fig. S10.** Example comparison of Chl B40 region.

**Fig. S11.** Sequence alignment comparing FRL sequences to WL and non-FaRLiP sequences.

**Fig. S12.** Cone scans for Chls at sites A21, B19, B7, B30, B37, and B38.

**Fig. S13.** Conservation of Fd binding between *Synechococcus* 7335 and *T. elongatus* PSI.

**Fig. S14.** Proximity of the A_1A_ phylloquinone to the low occupancy structural elements.

**Fig. S15.** Chl *f* positions relative to the electron transfer chain cofactors.

**Fig. S16.** Structural features nearby the C2 moiety of Chl B19 in FRL-PSI from *Fischerella* 7521.

**Table S1.** Cryo-EM data collection, refinement, and validation statistics for FRL-PSI from *Synechococcus* 7335.

**Table S2.** Correlation between PsaB loop D sequence and the PsaX subunit.

**Table S3.** Comparison of stromal ridge subunits and Fd between *T. elongatus* PSI and *Synechococcus* 7335 FRL-PSI.

**Table S4.** Edge-to-edge distances of Fe-S clusters in PSI structures with Fd bound.

**Data S1.** Jupyter Notebook for cone scans (external file).

**Supporting Figures**


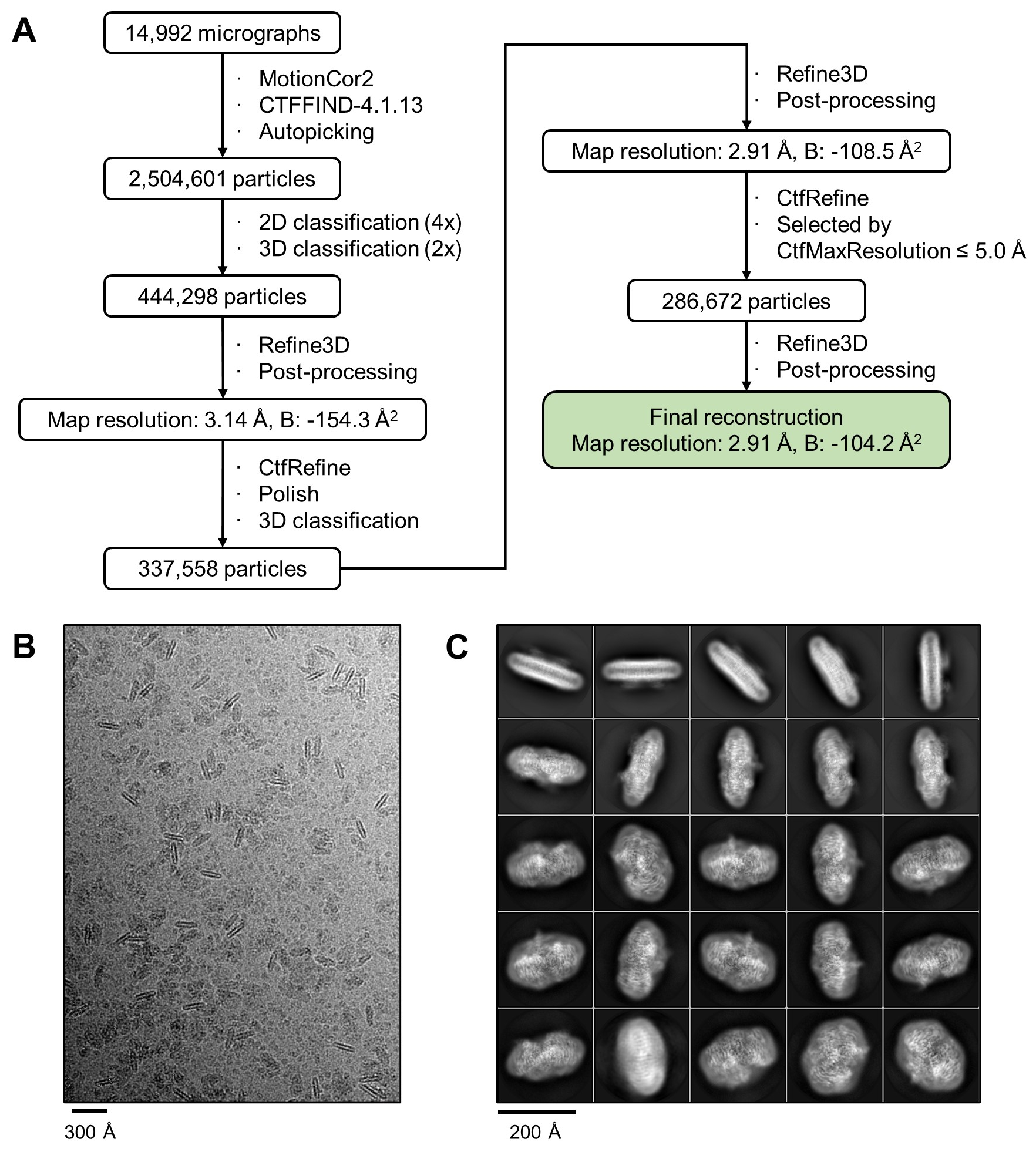


**Fig. S1. Cryo-EM data processing workflow, example micrograph image, and example 2D class images.** **A** Workflow for the cryo-EM data processing using RELION 3.1 (62). **B** An example micrograph after motion correction. **C** Example of 2D classes.


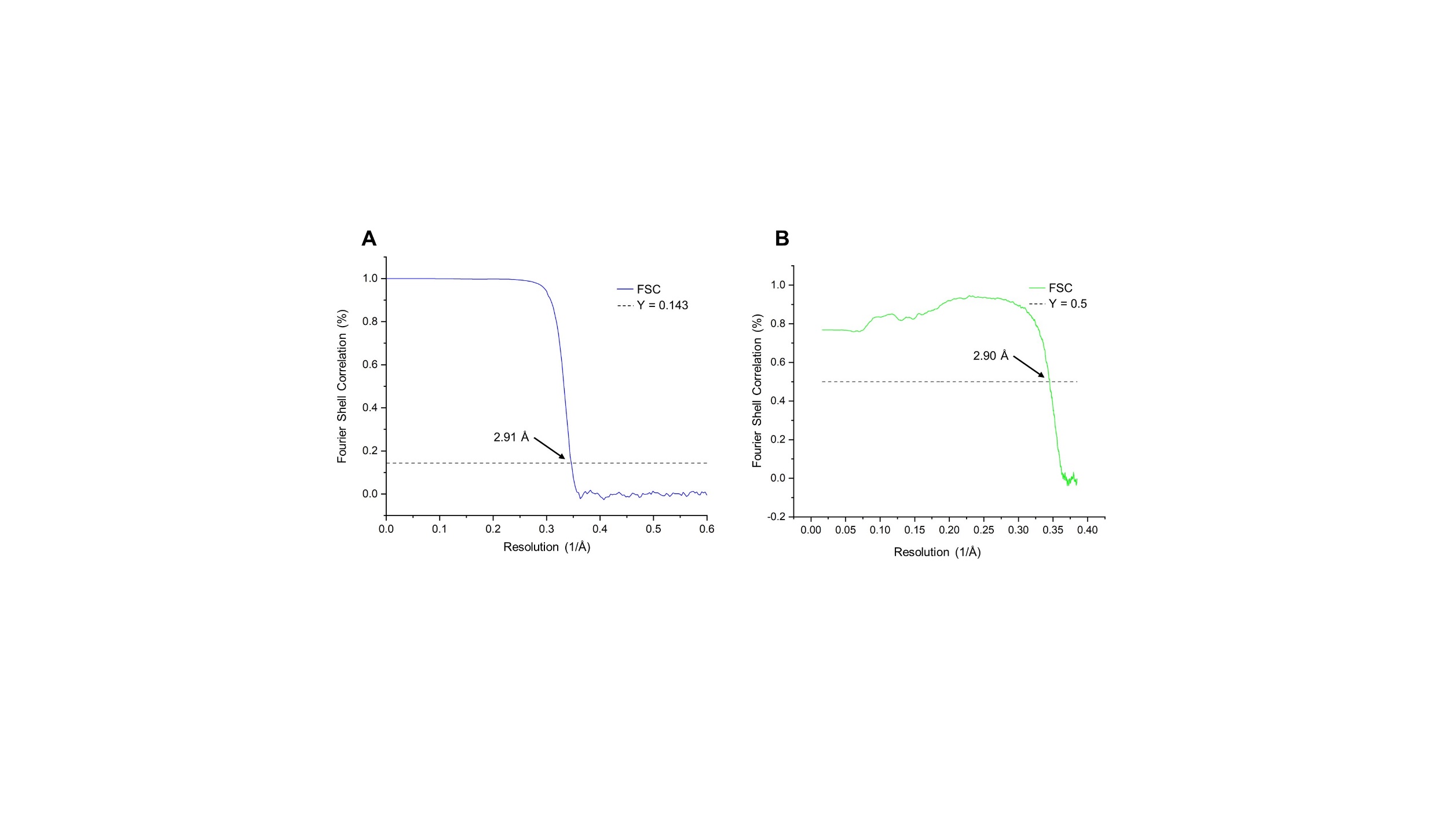


**Fig. S2. FSC curves for the *Synechococcus*** **7335 FRL-PSI structure.** **A** Map-map FSC. **B.** Map-model FSC.


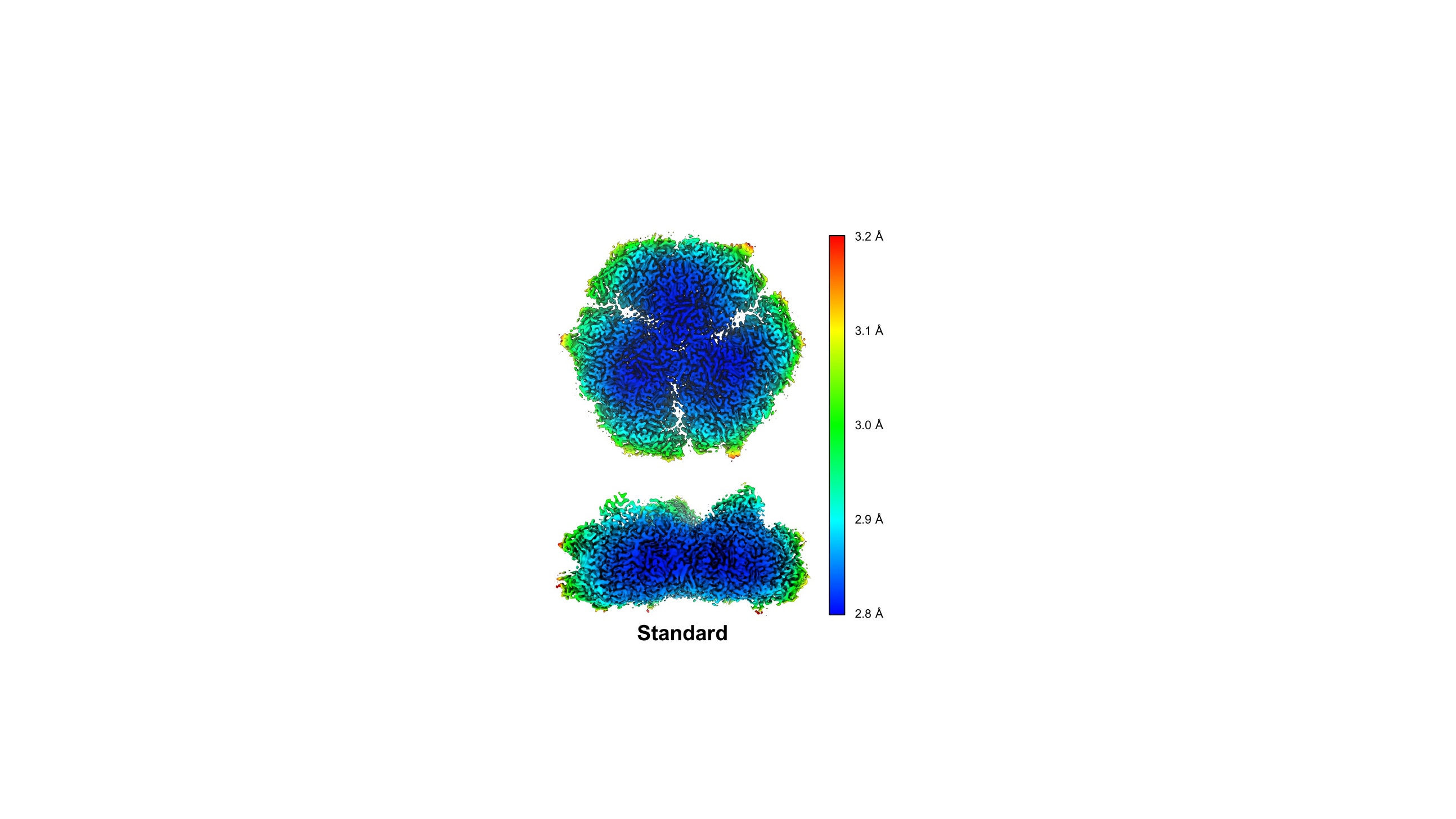


**Fig. S3. Local resolution of the *Synechococcus* 7335 FRL-PSI cryo-EM map.** A slice through the local resolution map is shown where the top image is a view from the stromal side and the bottom image is a view from the membrane plane.

**WP_006454866.1**

**MASYKVTLVNETENLNTTIEVADDEYILDAAEEQGIDLPYSCRAGACSTCAGKLTEGTVDQSDQSFLDDDQIEAGYVLTCVAYPTSDCTVMTHQEEELY**

WP_006456492.1

MATYNVTLINEDEGINETIEVADDQYIIEAAEDAGIDLPASCRAGSCSSCTGKLVSGSVNQEDQVFLDDDQMEAGFVLTCVAYATSDCTIQTHQEDALL

WP_038015354.1

MTTYQVRLINKKRKIDVTIPVEDDAYILDAAEENDIDLPYTCRAGACSSCVGKIVEGEVDQEDQSFLEDEQIEKGFALLCTSYPRSDLTIKTHMEAYLI

EDX86028.1

MMTTYQVRLINKKRKIDVTIPVEDDAYILDAAEENDIDLPYTCRAGACSSCVGKIVEGEVDQEDQSFLEDEQIEKGFALLCTSYPRSDLTIKTHMEAYLI

WP_038016504.1

MGNTFTAEVLHRGTTHTVEVPSDKPLLDTLQAAGLDLPFSCSAGVCTTCAALVTEGTVNQEDGMGVSPELQADGYALLCVALPTSDLKLETEKEDEVYARQFGQQG

WP_006453542.1

MVETYSVRIHHRQKNTVYTVRVPSDRYILQTAENQAADLPYACRNGACTSCAVRVLSGELHQPEAMGLSPDLREQGYALLCVSYPRSDLEVETQDEDEVYELQFGRYFGKGPTRSGILIDDD

**Fig. S4. Fd sequences identified in the blastp** (22) **search of *Synechococcus* 7335.** The NCBI accession code is listed for each sequence. The bold sequence fit the cryo-EM map best and was therefore used for modeling.


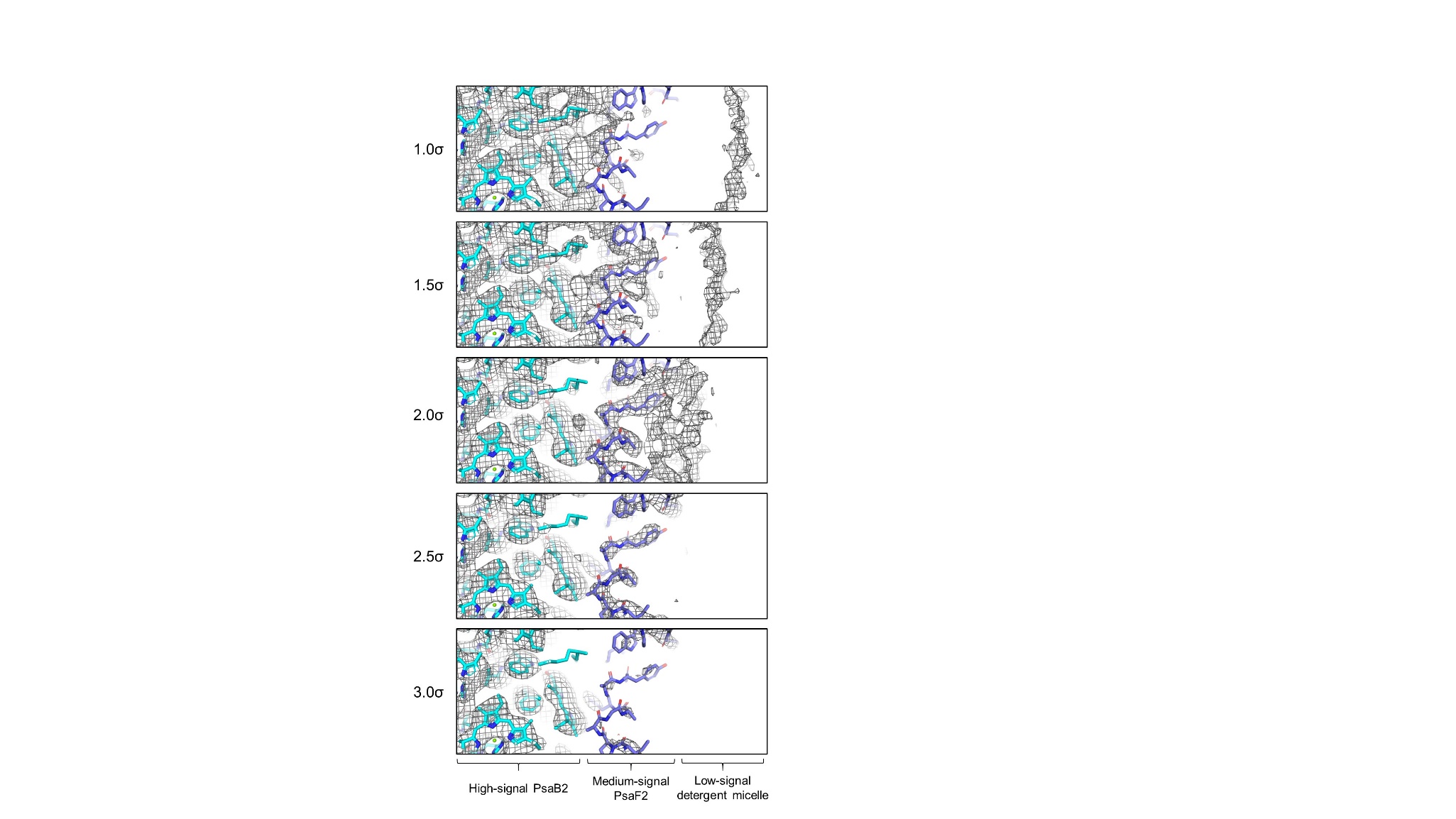


**Fig. S5. ESP for low-occupancy subunits PsaF2 and PsaJ2.** View of the transmembrane region near PsaF2. The model is shown within the unsharpened map at different contour levels. PsaF2 is shown in blue and PsaA2 is shown in cyan. As the map is increased in contour (i.e., low signal being hidden from view), the detergent belt can be seen at low signal which overlaps with PsaF2 which is present at medium signal. The high signal PsaA2 structural elements show signal at all these contour levels.


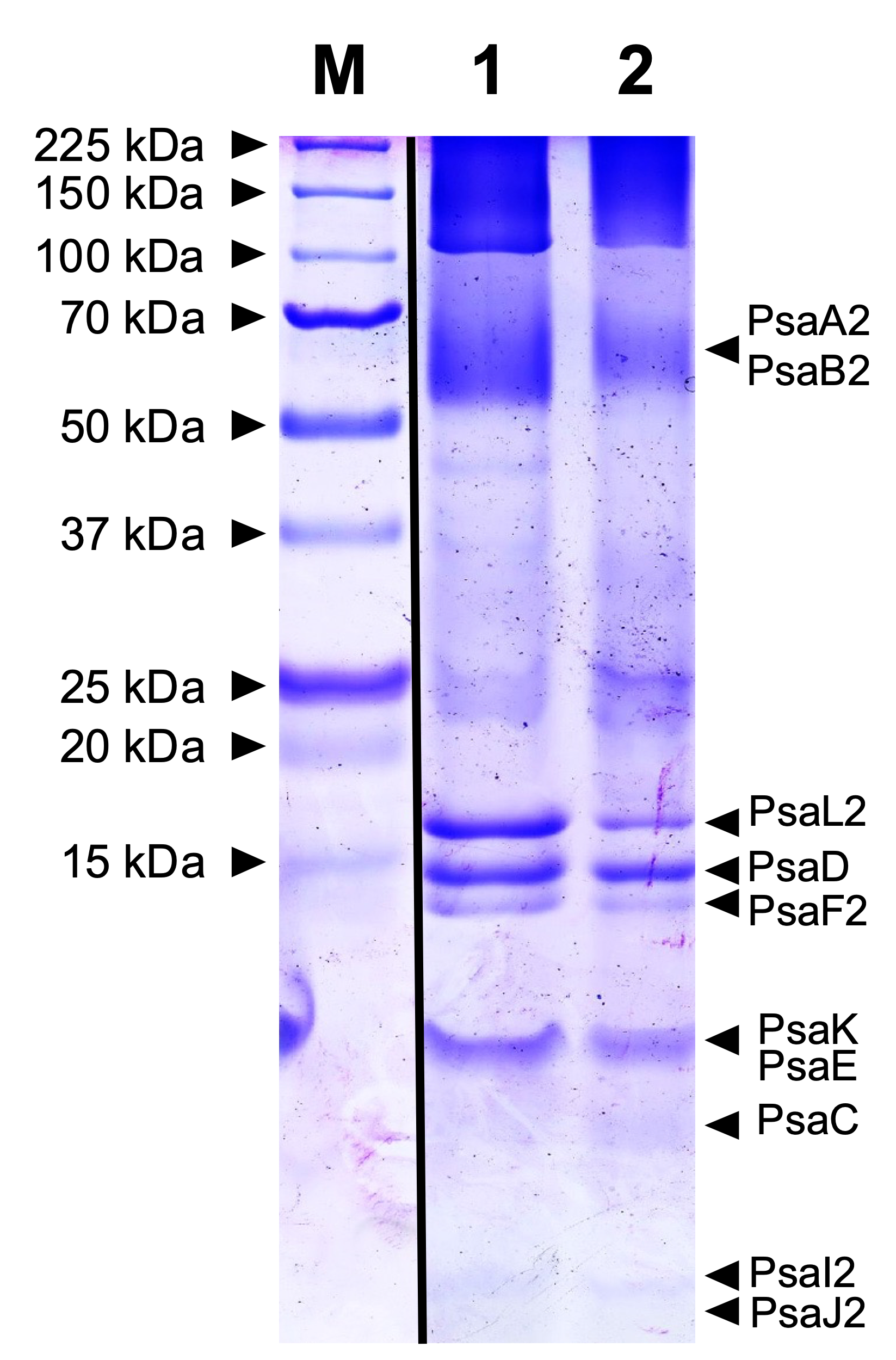


**Fig. S6. SDS-PAGE of *Synechococcus* 7335 FRL-PSI.** For lane 1, protein corresponding to 15 µg of Chl was loaded, and for lane 2, protein corresponding to 10 µg of Chl was loaded. Molecular mass markers (M) are shown on the left, and the proteins corresponding to the subunits of FRL-PSI are indicated on the right. Proteins were stained with Coomassie blue. The thick black line between lanes M and 1 indicates the excision of intervening lanes from the same gel that were removed for clarity.


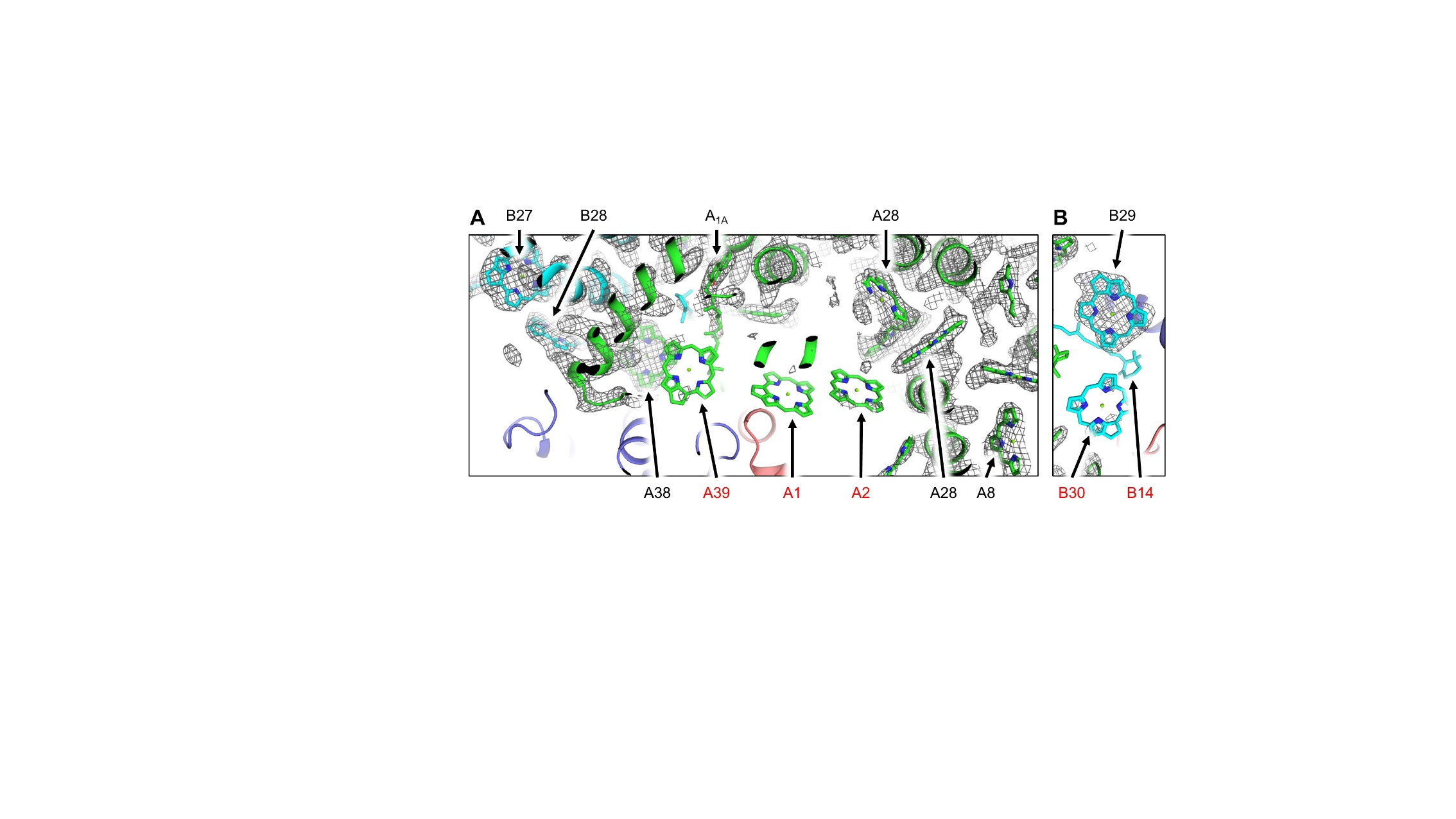


**Fig. S7.** **ESP for low occupancy pigment sites near PsaF2 and PsaJ2.** For both panels, the unsharpened map is shown at 6σ. For the model, the cartoon representation of the protein is shown and pigments are shown in stick representation. For Chls, only tetrapyrrole rings are shown for clarity. Red font denotes pigment sites that exhibit low occupancy and black font denotes pigments that exhibit high occupancy. **A** Stromal-side pigments near PsaF2 and PsaJ2. Three Chls appear at low occupancy: those in sites A39, A1, and A2. **B** Lumenal-side pigments near PsaF2 and PsaJ2. One Chl site, B30, and one β-carotene site, B14, appear at low occupancy.

PsaA

|  | ***S*.**  **7335 (FRL)** | ***H.***  ***hong.* (FRL)** | ***F.***  ***7521* (FRL)** | ***H.***  ***hong.* (WL)** | ***T.***  ***elongatus*** | ***S.***  **6803** | ***A.***  **7120** |
| --- | --- | --- | --- | --- | --- | --- | --- |
| ***S*. 7335 (FRL)** | 100.00 | 0.315 | 0.359 | 0.352 | 0.436 | 0.427 | 0.492 |
| ***H. hong.* (FRL)** | 0.315 | 100.00 | 0.443 | 0.282 | 0.409 | 0.428 | 0.423 |
| ***F. 7521* (FRL)** | 0.359 | 0.443 | 100.00 | 0.441 | 0.440 | 0.404 | 0.436 |
| ***H. hong.* (WL)** | 0.352 | 0.282 | 0.441 |  | 0.373 | 0.414 | 0.424 |
| ***T. elongatus*** | 0.436 | 0.409 | 0.440 | 0.373 |  | 0.285 | 0.345 |
| ***S.* 6803** | 0.427 | 0.428 | 0.404 | 0.414 | 0.285 |  | 0.374 |
| ***A.* 7120** | 0.492 | 0.423 | 0.436 | 0.424 | 0.345 | 0.374 |  |

PsaB

|  | ***S*.**  **7335 (FRL)** | ***H.***  ***hong.* (FRL)** | ***F.***  ***7521* (FRL)** | ***H.***  ***hong.* (WL)** | ***T.***  ***elongatus*** | ***S.***  **6803** | ***A.***  **7120** |
| --- | --- | --- | --- | --- | --- | --- | --- |
| ***S*. 7335 (FRL)** |  | 0.281 | 0.360 | 0.321 | 0.362 | 0.357 | 0.355 |
| ***H. hong.* (FRL)** | 0.281 |  | 0.468 | 0.287 | 0.385 | 0.409 | 0.436 |
| ***F. 7521* (FRL)** | 0.360 | 0.468 |  | 0.456 | 0.467 | 0.408 | 0.417 |
| ***H. hong.* (WL)** | 0.321 | 0.287 | 0.456 |  | 0.366 | 0.406 | 0.366 |
| ***T. elongatus*** | 0.362 | 0.385 | 0.467 | 0.366 |  | 0.277 | 0.307 |
| ***S.* 6803** | 0.357 | 0.409 | 0.408 | 0.406 | 0.277 |  | 0.300 |
| ***A.* 7120** | 0.355 | 0.436 | 0.417 | 0.366 | 0.307 | 0.300 |  |

PsaC

|  | ***S*.**  **7335 (FRL)** | ***H.***  ***hong.* (FRL)** | ***F.***  ***7521* (FRL)** | ***H.***  ***hong.* (WL)** | ***T.***  ***elongatus*** | ***S.***  **6803** | ***A.***  **7120** |
| --- | --- | --- | --- | --- | --- | --- | --- |
| ***S*. 7335 (FRL)** |  | 0.205 | 0.342 | 0.197 | 0.243 | 0.257 | 0.231 |
| ***H. hong.* (FRL)** | 0.205 |  | 0.317 | 0.125 | 0.267 | 0.282 | 0.270 |
| ***F. 7521* (FRL)** | 0.342 | 0.317 |  | 0.326 | 0.399 | 0.408 | 0.342 |
| ***H. hong.* (WL)** | 0.197 | 0.125 | 0.326 |  | 0.274 | 0.312 | 0.255 |
| ***T. elongatus*** | 0.243 | 0.267 | 0.399 | 0.274 |  | 0.249 | 0.250 |
| ***S.* 6803** | 0.257 | 0.282 | 0.408 | 0.312 | 0.249 |  | 0.276 |
| ***A.* 7120** | 0.231 | 0.270 | 0.342 | 0.255 | 0.250 | 0.276 |  |

PsaD

|  | ***S*.**  **7335 (FRL)** | ***H.***  ***hong.* (FRL)** | ***F.***  ***7521* (FRL)** | ***H.***  ***hong.* (WL)** | ***T.***  ***elongatus*** | ***S.***  **6803** | ***A.***  **7120** |
| --- | --- | --- | --- | --- | --- | --- | --- |
| ***S*. 7335 (FRL)** |  | 0.234 | 0.440 | 0.342 | 0.495 | 0.412 | 0.360 |
| ***H. hong.* (FRL)** | 0.234 |  | 0.286 | 0.163 | 0.390 | 0.312 | 0.285 |
| ***F. 7521* (FRL)** | 0.440 | 0.286 |  | 0.317 | 0.499 | 0.548 | 0.436 |
| ***H. hong.* (WL)** | 0.342 | 0.163 | 0.317 |  | 0.361 | 0.355 | 0.323 |
| ***T. elongatus*** | 0.495 | 0.390 | 0.499 | 0.361 |  | 0.461 | 0.441 |
| ***S.* 6803** | 0.412 | 0.312 | 0.548 | 0.355 | 0.461 |  | 0.386 |
| ***A.* 7120** | 0.360 | 0.285 | 0.436 | 0.323 | 0.441 | 0.386 |  |

PsaE

|  | ***S*.**  **7335 (FRL)** | ***H.***  ***hong.* (FRL)** | ***F.***  ***7521* (FRL)** | ***H.***  ***hong.* (WL)** | ***T.***  ***elongatus*** | ***S.***  **6803** | ***A.***  **7120** |
| --- | --- | --- | --- | --- | --- | --- | --- |
| ***S*. 7335 (FRL)** |  | 0.289 | 0.304 | 0.555 | 0.378 | 0.383 | 0.274 |
| ***H. hong.* (FRL)** | 0.289 |  | 0.375 | 0.528 | 0.450 | 0.425 | 0.332 |
| ***F. 7521* (FRL)** | 0.304 | 0.375 |  | 0.507 | 0.508 | 0.526 | 0.304 |
| ***H. hong.* (WL)** | 0.555 | 0.528 | 0.507 |  | 0.728 | 0.603 | 0.541 |
| ***T. elongatus*** | 0.378 | 0.450 | 0.508 | 0.728 |  | 0.435 | 0.359 |
| ***S.* 6803** | 0.383 | 0.425 | 0.526 | 0.603 | 0.435 |  | 0.347 |
| ***A.* 7120** | 0.274 | 0.332 | 0.304 | 0.541 | 0.359 | 0.347 |  |

PsaF

|  | ***S*.**  **7335 (FRL)** | ***H.***  ***hong.* (FRL)** | ***F.***  ***7521* (FRL)** | ***H.***  ***hong.* (WL)** | ***T.***  ***elongatus*** | ***S.***  **6803** | ***A.***  **7120** |
| --- | --- | --- | --- | --- | --- | --- | --- |
| ***S*. 7335 (FRL)** |  | N/A | 0.843 | N/A | 0.741 | 0.979 | 0.833 |
| ***H. hong.* (FRL)** | N/A |  | N/A | N/A | N/A | N/A | N/A |
| ***F. 7521* (FRL)** | 0.843 | N/A |  | N/A | 0.723 | 1.095 | 0.871 |
| ***H. hong.* (WL)** | N/A | N/A | N/A | N/A | N/A | N/A | N/A |
| ***T. elongatus*** | 0.741 | N/A | 0.723 | N/A |  | 0.574 | 0.356 |
| ***S.* 6803** | 0.979 | N/A | 1.095 | N/A | 0.574 |  | 0.488 |
| ***A.* 7120** | 0.833 | N/A | 0.871 | N/A | 0.356 | 0.488 |  |

PsaI

|  | ***S*.**  **7335 (FRL)** | ***H.***  ***hong.* (FRL)** | ***F.***  ***7521* (FRL)** | ***H.***  ***hong.* (WL)** | ***T.***  ***elongatus*** | ***S.***  **6803** | ***A.***  **7120** |
| --- | --- | --- | --- | --- | --- | --- | --- |
| ***S*. 7335 (FRL)** |  | 0.254 | 0.383 | 0.744 | 0.674 | 0.469 | 0.703 |
| ***H. hong.* (FRL)** | 0.254 |  | 0.327 | 0.717 | 0.760 | 0.481 | 0.706 |
| ***F. 7521* (FRL)** | 0.383 | 0.327 |  | 0.829 | 0.907 | 0.782 | 0.948 |
| ***H. hong.* (WL)** | 0.744 | 0.717 | 0.829 |  | 0.289 | 0.361 | 0.447 |
| ***T. elongatus*** | 0.674 | 0.760 | 0.907 | 0.289 |  | 0.252 | 0.371 |
| ***S.* 6803** | 0.469 | 0.481 | 0.782 | 0.361 | 0.252 |  | 0.347 |
| ***A.* 7120** | 0.703 | 0.706 | 0.948 | 0.447 | 0.371 | 0.347 |  |

PsaJ

|  | ***S*.**  **7335 (FRL)** | ***H.***  ***hong.* (FRL)** | ***F.***  ***7521* (FRL)** | ***H.***  ***hong.* (WL)** | ***T.***  ***elongatus*** | ***S.***  **6803** | ***A.***  **7120** |
| --- | --- | --- | --- | --- | --- | --- | --- |
| ***S*. 7335 (FRL)** |  | N/A | 0.568 | N/A | 0.615 | 0.616 | 0.911 |
| ***H. hong.* (FRL)** | N/A |  | N/A | N/A | N/A | N/A | N/A |
| ***F. 7521* (FRL)** | 0.568 | N/A |  | N/A | 0.695 | 0.695 | 0.857 |
| ***H. hong.* (WL)** | N/A | N/A | N/A |  | N/A | N/A | N/A |
| ***T. elongatus*** | 0.615 | N/A | 0.695 | N/A |  | 0.285 | 0.209 |
| ***S.* 6803** | 0.616 | N/A | 0.695 | N/A | 0.285 |  | 0.303 |
| ***A.* 7120** | 0.911 | N/A | 0.857 | N/A | 0.209 | 0.303 |  |

PsaK

|  | ***S*.**  **7335 (FRL)** | ***H.***  ***hong.* (FRL)** | ***F.***  ***7521* (FRL)** | ***H.***  ***hong.* (WL)** | ***T.***  ***elongatus*** | ***S.***  **6803** | ***A.***  **7120** |
| --- | --- | --- | --- | --- | --- | --- | --- |
| ***S*. 7335 (FRL)** |  | 0.797 | 3.145 | N/A | 0.726 | 0.311 | 0.640 |
| ***H. hong.* (FRL)** | 0.797 |  | 2.361 | N/A | 0.548 | 0.533 | 0.519 |
| ***F. 7521* (FRL)** | 3.145 | 2.361 |  | N/A | 3.116 | 3.357 | 0.760 |
| ***H. hong.* (WL)** | N/A | N/A | N/A |  | N/A | N/A | N/A |
| ***T. elongatus*** | 0.726 | 0.548 | 3.116 | N/A |  | 2.440 | 2.393 |
| ***S.* 6803** | 0.311 | 0.533 | 3.357 | N/A | 2.440 |  | 0.388 |
| ***A.* 7120** | 0.640 | 0.519 | 0.760 | N/A | 2.393 | 0.388 |  |

PsaL

|  | ***S*.**  **7335 (FRL)** | ***H.***  ***hong.* (FRL)** | ***F.***  ***7521* (FRL)** | ***H.***  ***hong.* (WL)** | ***T.***  ***elongatus*** | ***S.***  **6803** | ***A.***  **7120** |
| --- | --- | --- | --- | --- | --- | --- | --- |
| ***S*. 7335 (FRL)** |  | 0.270 | 0.397 | 0.455 | 0.663 | 0.780 | 0.935 |
| ***H. hong.* (FRL)** | 0.270 |  | 0.381 | 0.465 | 0.685 | 0.782 | 0.870 |
| ***F. 7521* (FRL)** | 0.397 | 0.381 |  | 0.492 | 0.817 | 0.765 | 2.464 |
| ***H. hong.* (WL)** | 0.455 | 0.465 | 0.492 |  | 0.506 | 0.523 | 1.087 |
| ***T. elongatus*** | 0.663 | 0.685 | 0.817 | 0.506 |  | 0.374 | 0.688 |
| ***S.* 6803** | 0.780 | 0.782 | 0.765 | 0.523 | 0.374 |  | 0.706 |
| ***A.* 7120** | 0.935 | 0.870 | 2.464 | 1.087 | 0.688 | 0.706 |  |

PsaM

|  | ***S*.**  **7335 (FRL)** | ***H.***  ***hong.* (FRL)** | ***F.***  ***7521* (FRL)** | ***H.***  ***hong.* (WL)** | ***T.***  ***elongatus*** | ***S.***  **6803** | ***A.***  **7120** |
| --- | --- | --- | --- | --- | --- | --- | --- |
| ***S*. 7335 (FRL)** |  | 0.272 | 0.277 | 0.288 | 0.323 | 0.412 | 0.286 |
| ***H. hong.* (FRL)** | 0.272 |  | 0.510 | 0.156 | 0.171 | 0.290 | 3.630 |
| ***F. 7521* (FRL)** | 0.277 | 0.510 |  | 0.508 | 0.479 | 0.396 | 0.372 |
| ***H. hong.* (WL)** | 0.288 | 0.156 | 0.508 |  | 0.155 | 0.301 | 0.231 |
| ***T. elongatus*** | 0.323 | 0.171 | 0.479 | 0.155 |  | 0.228 | 3.535 |
| ***S.* 6803** | 0.412 | 0.290 | 0.396 | 0.301 | 0.228 |  | 0.306 |
| ***A.* 7120** | 0.286 | 3.630 | 0.372 | 0.231 | 3.535 | 0.306 |  |

PsaX

|  | ***F. 7521* (FRL)** | ***T. elongatus*** |
| --- | --- | --- |
| ***F. 7521* (FRL)** |  | 0.429 |
| ***T. elongatus*** | 0.429 |  |

**Fig. S8.** Structural similarity of selected PSI subunits. C_α_ atoms of subunits only were superimposed. The root-mean square-deviation (RMSD) is reported in units of Å. Boxes shaded in pink denote FRL-specific subunits. Abbreviations: *Synechococcus* sp. PCC 7335, *S.* 7335; *H. hongdechloris.*, *H. hong.*; *Fischerella thermalis* PCC 7521*,* *F*. 7521; *Synechocystis* sp. PCC 6803, *S.* 6803; *Anabaena* sp. PCC 7120, *A*. 7120. The structures used correspond to PDBs 7S3D (trimeric FRL-PSI from *Synechococcus* 7335 presented here), 6KMX (trimeric FRL-PSI from *H. hongdechloris*), 7LX0 (trimeric FRL-PSI from *Fischerella* 7521), 6KMW (trimeric WL-PSI from *H. hongdechloris*), 1JB0 (trimeric PSI from the non-FaRLiP *T. elongatus*), 5OY0 (trimeric PSI from the non-FaRLiP *Synechocystis* 6803), and 6K61 (dimeric/tetrameric PSI from the non-FaRLiP strain *Anabaena* sp. PCC 7120).

PsaA

|  | ***S*.**  **7335 (FRL)** | ***H.***  ***hong.* (FRL)** | ***F.***  ***7521* (FRL)** | ***H.***  ***hong.* (WL)** | ***T.***  ***elongatus*** | ***S.***  **6803** | ***A.***  **7120** |
| --- | --- | --- | --- | --- | --- | --- | --- |
| ***S*. 7335 (FRL)** | 100.00 | 82.48 | 81.31 | 77.17 | 79.57 | 79.57 | 78.53 |
| ***H. hong.* (FRL)** | 82.48 | 100.00 | 79.69 | 77.20 | 77.20 | 75.73 | 76.03 |
| ***F. 7521* (FRL)** | 81.31 | 79.69 | 100.00 | 77.84 | 83.13 | 79.71 | 81.20 |
| ***H. hong.* (WL)** | 77.17 | 77.20 | 77.84 | 100.00 | 85.75 | 82.69 | 82.69 |
| ***T. elongatus*** | 79.57 | 77.20 | 83.13 | 85.75 | 100.00 | 87.75 | 87.22 |
| ***S.* 6803** | 79.57 | 75.73 | 79.71 | 82.69 | 87.75 | 100.00 | 84.69 |
| ***A.* 7120** | 78.53 | 76.03 | 81.20 | 82.69 | 87.22 | 84.69 | 100.00 |

PsaB

|  | ***S*.**  **7335 (FRL)** | ***H.***  ***hong.* (FRL)** | ***F.***  ***7521* (FRL)** | ***H.***  ***hong.* (WL)** | ***T.***  ***elongatus*** | ***S.***  **6803** | ***A.***  **7120** |
| --- | --- | --- | --- | --- | --- | --- | --- |
| ***S*. 7335 (FRL)** | 100.00 | 88.02 | 82.97 | 81.78 | 81.60 | 81.37 | 80.41 |
| ***H. hong.* (FRL)** | 88.02 | 100.00 | 81.89 | 81.78 | 82.41 | 80.82 | 80.00 |
| ***F. 7521* (FRL)** | 82.97 | 81.89 | 100.00 | 80.81 | 84.57 | 81.07 | 83.78 |
| ***H. hong.* (WL)** | 81.78 | 81.78 | 80.81 | 100.00 | 88.78 | 85.09 | 85.29 |
| ***T. elongatus*** | 81.60 | 82.41 | 84.57 | 88.78 | 100.00 | 87.38 | 90.00 |
| ***S.* 6803** | 81.37 | 80.82 | 81.07 | 85.09 | 87.38 | 100.00 | 86.99 |
| ***A.* 7120** | 80.41 | 80.00 | 83.78 | 85.29 | 90.00 | 86.99 | 100.00 |

PsaC

|  | ***S*.**  **7335 (FRL)** | ***H.***  ***hong.* (FRL)** | ***F.***  ***7521* (FRL)** | ***H.***  ***hong.* (WL)** | ***T.***  ***elongatus*** | ***S.***  **6803** | ***A.***  **7120** |
| --- | --- | --- | --- | --- | --- | --- | --- |
| ***S*. 7335 (FRL)** | 100.00 | 93.83 | 96.3 | 93.83 | 93.75 | 93.83 | 92.59 |
| ***H. hong.* (FRL)** | 93.83 | 100.00 | 95.06 | 100 | 95 | 97.53 | 93.83 |
| ***F. 7521* (FRL)** | 96.3 | 95.06 | 100.00 | 95.06 | 97.5 | 95.06 | 96.3 |
| ***H. hong.* (WL)** | 93.83 | 100 | 95.06 | 100.00 | 95 | 97.53 | 93.83 |
| ***T. elongatus*** | 93.75 | 95 | 97.5 | 95 | 100.00 | 95 | 96.25 |
| ***S.* 6803** | 93.83 | 95.06 | 97.53 | 97.53 | 95 | 100.00 | 96.3 |
| ***A.* 7120** | 92.59 | 93.83 | 96.3 | 93.83 | 96.25 | 96.3 | 100.00 |

PsaD

|  | ***S*.**  **7335 (FRL)** | ***H.***  ***hong.* (FRL)** | ***F.***  ***7521* (FRL)** | ***H.***  ***hong.* (WL)** | ***T.***  ***elongatus*** | ***S.***  **6803** | ***A.***  **7120** |
| --- | --- | --- | --- | --- | --- | --- | --- |
| ***S*. 7335 (FRL)** | 100.00 | 77.14 | 69.29 | 77.14 | 65.22 | 74.29 | 68.61 |
| ***H. hong.* (FRL)** | 77.14 | 100.00 | 76.26 | 100.00 | 65.94 | 78.01 | 71.94 |
| ***F. 7521* (FRL)** | 69.29 | 76.26 | 100.00 | 76.26 | 69.34 | 72.46 | 79.86 |
| ***H. hong.* (WL)** | 77.14 | 100.00 | 76.26 | 100.00 | 65.94 | 78.01 | 71.94 |
| ***T. elongatus*** | 65.22 | 65.94 | 69.34 | 65.94 | 100.00 | 67.39 | 67.15 |
| ***S.* 6803** | 74.29 | 78.01 | 72.46 | 78.01 | 67.39 | 100.00 | 65.94 |
| ***A.* 7120** | 68.61 | 71.94 | 79.86 | 71.94 | 67.15 | 65.94 | 100.00 |

PsaE

|  | ***S*.**  **7335 (FRL)** | ***H.***  ***hong.* (FRL)** | ***F.***  ***7521* (FRL)** | ***H.***  ***hong.* (WL)** | ***T.***  ***elongatus*** | ***S.***  **6803** | ***A.***  **7120** |
| --- | --- | --- | --- | --- | --- | --- | --- |
| ***S*. 7335 (FRL)** | 100.00 | 70.59 | 76.06 | 70.59 | 70.15 | 65.57 | 81.43 |
| ***H. hong.* (FRL)** | 70.59 | 100.00 | 72.06 | 100.00 | 71.64 | 65.57 | 77.94 |
| ***F. 7521* (FRL)** | 76.06 | 72.06 | 100.00 | 72.06 | 69.12 | 68.85 | 88.57 |
| ***H. hong.* (WL)** | 70.59 | 100.00 | 72.06 | 100.00 | 71.64 | 65.57 | 77.94 |
| ***T. elongatus*** | 70.15 | 71.64 | 69.12 | 71.64 | 100.00 | 67.16 | 73.13 |
| ***S.* 6803** | 65.57 | 65.57 | 68.85 | 65.57 | 67.16 | 100.00 | 70.49 |
| ***A.* 7120** | 81.43 | 77.94 | 88.57 | 77.94 | 73.13 | 70.49 | 100.00 |

PsaF

|  | ***S*.**  **7335 (FRL)** | ***H.***  ***hong.* (FRL)** | ***F.***  ***7521* (FRL)** | ***H.***  ***hong.* (WL)** | ***T.***  ***elongatus*** | ***S.***  **6803** | ***A.***  **7120** |
| --- | --- | --- | --- | --- | --- | --- | --- |
| ***S*. 7335 (FRL)** | 100.00 | 65.62 | 51.9 | 41.77 | 47.47 | 43.07 | 44.3 |
| ***H. hong.* (FRL)** | 65.62 | 100.00 | 54.43 | 46.2 | 47.47 | 41.61 | 45.57 |
| ***F. 7521* (FRL)** | 51.9 | 54.43 | 100.00 | 44.65 | 49.69 | 40.58 | 47.17 |
| ***H. hong.* (WL)** | 41.77 | 46.2 | 44.65 | 100.00 | 62.73 | 53.57 | 55.28 |
| ***T. elongatus*** | 47.47 | 47.47 | 49.69 | 62.73 | 100.00 | 59.44 | 67.07 |
| ***S.* 6803** | 43.07 | 41.61 | 40.58 | 53.57 | 59.44 | 100.00 | 60.84 |
| ***A.* 7120** | 44.3 | 45.57 | 47.17 | 55.28 | 67.07 | 60.84 | 100.00 |

PsaI

|  | ***S*.**  **7335 (FRL)** | ***H.***  ***hong.* (FRL)** | ***F.***  ***7521* (FRL)** | ***H.***  ***hong.* (WL)** | ***T.***  ***elongatus*** | ***S.***  **6803** | ***A.***  **7120** |
| --- | --- | --- | --- | --- | --- | --- | --- |
| ***S*. 7335 (FRL)** | 100.00 | 63.49 | 58.06 | 39.47 | 42.11 | 42.5 | 32.61 |
| ***H. hong.* (FRL)** | 63.49 | 100.00 | 62.07 | 36.84 | 42.11 | 42.5 | 32.61 |
| ***F. 7521* (FRL)** | 58.06 | 62.07 | 100.00 | 47.37 | 52.63 | 42.5 | 28.26 |
| ***H. hong.* (WL)** | 39.47 | 36.84 | 47.37 | 100.00 | 73.68 | 71.05 | 31.58 |
| ***T. elongatus*** | 42.11 | 42.11 | 52.63 | 73.68 | 100.00 | 68.42 | 44.74 |
| ***S.* 6803** | 42.5 | 42.5 | 42.5 | 71.05 | 68.42 | 100.00 | 35 |
| ***A.* 7120** | 32.61 | 32.61 | 28.26 | 31.58 | 44.74 | 35 | 100.00 |

PsaJ

|  | ***S*.**  **7335 (FRL)** | ***H.***  ***hong.* (FRL)** | ***F.***  ***7521* (FRL)** | ***H.***  ***hong.* (WL)** | ***T.***  ***elongatus*** | ***S.***  **6803** | ***A.***  **7120** |
| --- | --- | --- | --- | --- | --- | --- | --- |
| ***S*. 7335 (FRL)** | 100.00 | 80.43 | 60.87 | 45 | 41.46 | 37.5 | 42.86 |
| ***H. hong.* (FRL)** | 80.43 | 100.00 | 63.04 | 47.5 | 39.02 | 35 | 45.24 |
| ***F. 7521* (FRL)** | 60.87 | 63.04 | 100.00 | 45 | 41.46 | 37.5 | 47.73 |
| ***H. hong.* (WL)** | 45 | 47.5 | 45 | 100.00 | 77.5 | 65 | 72.5 |
| ***T. elongatus*** | 41.46 | 39.02 | 41.46 | 77.5 | 100.00 | 67.5 | 70.73 |
| ***S.* 6803** | 37.5 | 35 | 37.5 | 65 | 67.5 | 100.00 | 67.5 |
| ***A.* 7120** | 42.86 | 45.24 | 47.73 | 72.5 | 70.73 | 67.5 | 100.00 |

PsaK

|  | ***S*.**  **7335 (FRL)** | ***H.***  ***hong.* (FRL)** | ***F.***  ***7521* (FRL)** | ***H.***  ***hong.* (WL)** | ***T.***  ***elongatus*** | ***S.***  **6803** | ***A.***  **7120** |
| --- | --- | --- | --- | --- | --- | --- | --- |
| ***S*. 7335 (FRL)** | 100.00 | 43.33 | 38.18 | 50.85 | 42.59 | 37.04 | 40.68 |
| ***H. hong.* (FRL)** | 43.33 | 100.00 | 30.12 | 51.16 | 51.81 | 57.5 | 41.86 |
| ***F. 7521* (FRL)** | 38.18 | 30.12 | 100.00 | 35 | 26.32 | 32.88 | 35 |
| ***H. hong.* (WL)** | 50.85 | 51.16 | 35 | 100.00 | 50.62 | 42.31 | 54.65 |
| ***T. elongatus*** | 42.59 | 51.81 | 26.32 | 50.62 | 100.00 | 47.5 | 40.74 |
| ***S.* 6803** | 37.04 | 57.5 | 32.88 | 42.31 | 47.5 | 100.00 | 43.59 |
| ***A.* 7120** | 40.68 | 41.86 | 35 | 54.65 | 40.74 | 43.59 | 100.00 |

PsaL

|  | ***S*.**  **7335 (FRL)** | ***H.***  ***hong.* (FRL)** | ***F.***  ***7521* (FRL)** | ***H.***  ***hong.* (WL)** | ***T.***  ***elongatus*** | ***S.***  **6803** | ***A.***  **7120** |
| --- | --- | --- | --- | --- | --- | --- | --- |
| ***S*. 7335 (FRL)** | 100.00 | 81.61 | 72.41 | 40.88 | 46.75 | 44.59 | 42.07 |
| ***H. hong.* (FRL)** | 81.61 | 100.00 | 72.41 | 44.65 | 48.05 | 45.86 | 42.94 |
| ***F. 7521* (FRL)** | 72.41 | 41 | 100.00 | 47.8 | 52.6 | 49.04 | 42.68 |
| ***H. hong.* (WL)** | 40.88 | 44.65 | 47.8 | 100.00 | 54.55 | 56.77 | 41.06 |
| ***T. elongatus*** | 46.75 | 48.05 | 52.6 | 54.55 | 100.00 | 72.73 | 48.3 |
| ***S.* 6803** | 44.59 | 45.86 | 49.04 | 56.77 | 72.73 | 100.00 | 47.33 |
| ***A.* 7120** | 42.07 | 42.94 | 42.68 | 41.06 | 48.3 | 47.33 | 100.00 |

PsaM

|  | ***S*.**  **7335 (FRL)** | ***H.***  ***hong.* (FRL)** | ***F.***  ***7521* (FRL)** | ***H.***  ***hong.* (WL)** | ***T.***  ***elongatus*** | ***S.***  **6803** | ***A.***  **7120** |
| --- | --- | --- | --- | --- | --- | --- | --- |
| ***S*. 7335 (FRL)** | 100.00 | 61.29 | 67.74 | 61.29 | 61.29 | 70.97 | 64.52 |
| ***H. hong.* (FRL)** | 61.29 | 100.00 | 67.74 | 100 | 80.65 | 74.19 | 61.29 |
| ***F. 7521* (FRL)** | 67.74 | 67.74 | 100.00 | 67.74 | 67.74 | 70.97 | 90.32 |
| ***H. hong.* (WL)** | 61.29 | 100 | 67.74 | 100.00 | 80.65 | 74.19 | 61.29 |
| ***T. elongatus*** | 61.29 | 80.65 | 67.74 | 80.65 | 100.00 | 80.65 | 64.52 |
| ***S.* 6803** | 70.97 | 74.19 | 70.97 | 74.19 | 80.65 | 100.00 | 64.52 |
| ***A.* 7120** | 64.52 | 61.29 | 90.32 | 61.29 | 64.52 | 64.52 | 100.00 |

PsaX

|  | ***F. 7521* (FRL)** | ***T. elongatus*** |
| --- | --- | --- |
| ***F. 7521* (FRL)** | 100.00 | 62.86 |
| ***T. elongatus*** | 62.86 | 100.00 |

**Fig. S9.** Sequence identities of selected PSI subunits. Sequences were aligned and identity calculated using Clustal Omega (39). Abbreviations: *Synechococcus* sp. PCC 7335, *S.* 7335; *H. hongdechloris.*, *H. hong.*; *Fischerella thermalis* PCC 7521*,* *F*. 7521; *Synechocystis* sp. PCC 6803, *S.* 6803; *Anabaena* sp. PCC 7120, *A*. 7120.

**
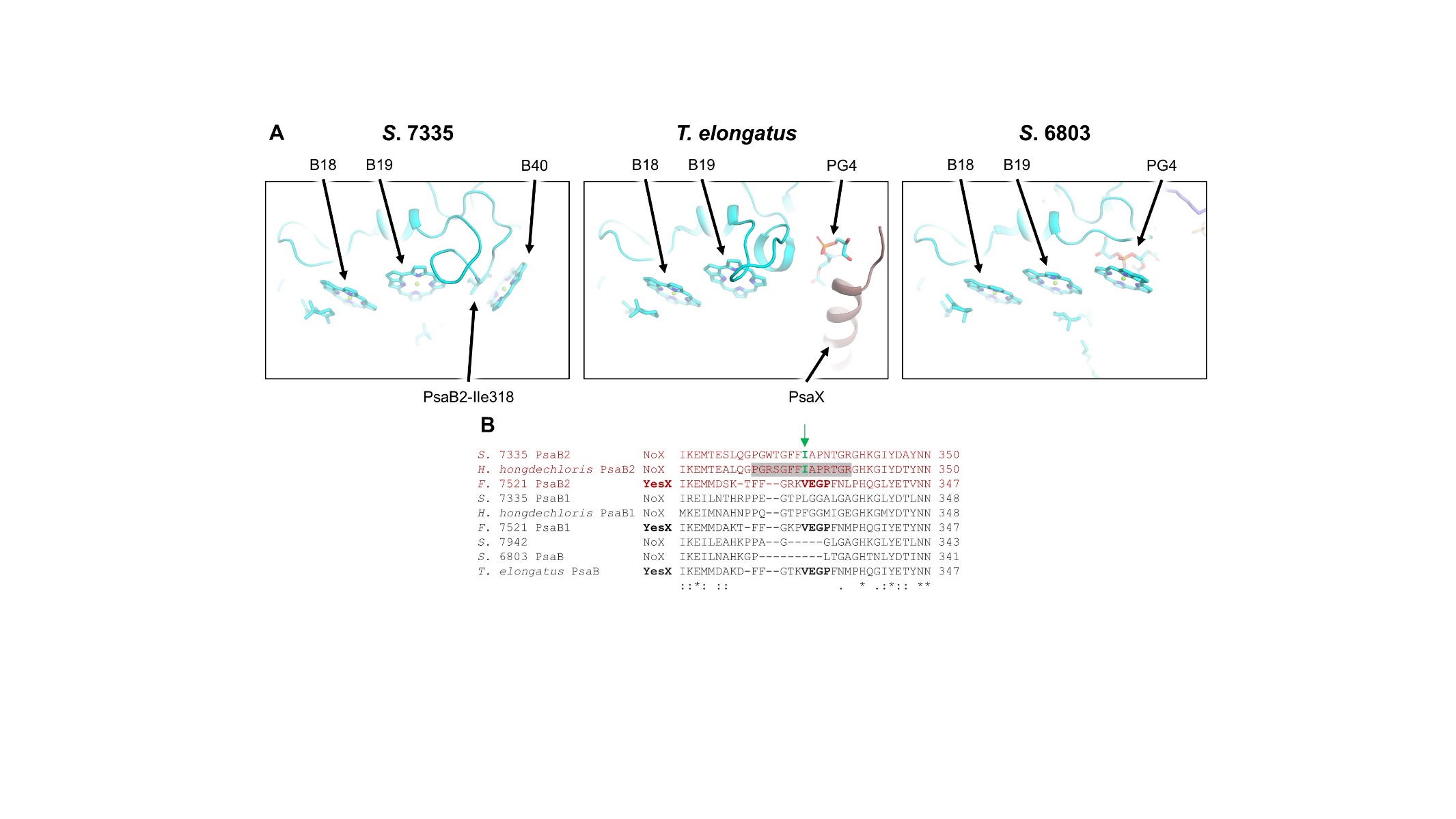
**

**Fig. S10. Example comparison of Chl B40 region. A** The region near Chl B40 site identified in *Synechococcus* 7335 FRL-PSI is shown for *Synechococcus* 7335 FRL-PSI, *T. elongatus* PSI, and *Synechocystis* sp. PCC 6803 PSI. The arrangement shown for *Synechococcus* 7335 is also probably similar to FRL-PSI from *H. hongdechloris*. The arrangement shown for *T. elongatus* is similar to all PsaX-containing PSIs, for example *Fischerella* 7521. The arrangement shown for *Synechocystis* sp. PCC 6803 (23) is similar to the PSI structures from *Synechococcus* sp. PCC 7942 (27) and all plant (28–30) and algal (31–34) PSI structures. **B.** Partial sequence alignment of the looping region that binds Chl B40 in *Synechococcus* 7335 FRL-PSI. FRL-specific sequences are colored in red and the region not modeled in the *H. hongdechloris* FRL-PSI structure is highlighted in grey. The Ile residue whose backbone carbonyl oxygen provides axial ligation to B40 in *Synechococcus* 7335 and probably *H.* *hongdechloris* FRL-PSI structures is shown in green with a green arrow. “YesX” or “NoX” corresponds to those structures that do or do not maintain PsaX, respectively. There appears to be a correlation between the VEGP sequence in PsaB and PsaX, which was tested against other sequence alignments in **Table S4**.

PsaA

*S.* 7335 PsaA2 FRL MTASPPK--RNQASAATEQSPIPTSFERWAKPGHFDRTLARGPKTTTWIWNLHADAHDFD 58

*H. hongdechloris* PsaA2 FRL MTTSPPEQ-RQRVRVEVDQNPNPTSFEKWAKPGHFERSLARGPKTTTWIWDLHADAHDFD 59

*F.* 7521 PsaA2 FRL MTLTP-ER-EQEVRVVVDNDPVPTSFQKWSQPGHFDRTLAKGAKTTTWIWNLHANAHDFD 58

*S.* 7335 PsaA1 WL MTTTPREREATKAKVVVDKNPTPTSFERWAKPGHFDRTLARGPKTTTWIWNLHADAHDFD 60

*H. hongdechloris* PsaA1 WL MTISPPEPGR-KVKVVVDSDPVNTSFERWAKPGHFERSLSKGPKTTTWIWDLHADAHDFD 59

*F.* 7521 PsaA1 WL MTISPPEREEKKARVIVDNDPVPTSFERWAKPGHFDRTLAKGPKTTTWIWNLHALAHDFD 60

*T. elongatus* PsaA MTISPPEREP-KVRVVVDNDPVPTSFEKWAKPGHFDRTLARGPQTTTWIWNLHALAHDFD 59

*S.* 6803 PsaA MTISPPEREA-KAKVSVDNNPVPTSFEKWGKPGHFDRTLARGPKTTTWIWNLHANAHDFD 59

** :* : .. . .:..* ***::*.:****:*:*::* :******:*** *****

*S.* 7335 PsaA2 FRL SHTNDLQDISRKIFSAHFGHLAVVFVWLSGMYFHGARFSNFSSWMADPTHIRPSAQVVWP 118

*H. hongdechloris* PsaA2 FRL SHTTDLEDISRKIFSAHFGHLAVIFLWLSGMYFHGARFSNFSSWMTDPIHIKPSAQVVWP 119

*F.* 7521 PsaA2 FRL THTSDLEDISRKIFAAHFGHLAVVFIWLSGMYFHGARFSNFEAWMANPTGIKPSAQVVWP 118

*S.* 7335 PsaA1 WL SHTSDLEDISRKIFSAHFGHLAVVFIWLSGMYFHGARFSNFEAWMSNPVGIKPSAQVVWP 120

*H. hongdechloris* PsaA1 WL SHTSDLEDISRKIFSAHFGHLAVIFIWLSGMYFHGAKFSNYEAWLSNPTGIKPSAQVVWP 119

*F.* 7521 PsaA1 WL THTSDLEDISRKIFAAHFGHLAVVTLWLSGMIFHGARFSNYEAWLSDPLNVRPSAQVVWP 120

*T. elongatus* PsaA THTSDLEDISRKIFSAHFGHLAVVFIWLSGMYFHGAKFSNYEAWLADPTGIKPSAQVVWP 119

*S.* 6803 PsaA SQTSDLEDVSRKIFSAHFGHLAVVFVWLSGMYFHGAKFSNYEGWLADPTHIKPSAQVVWP 119

::*.**:*:*****:********: :***** ****:***:..*:::* ::********

*S.* 7335 PsaA2 FRL LVGQDILNGDMGGGFRGIQITSGLFQMWRGEGFTNEFQLYCTAIGALVMAGLMIFAGWFH 178

*H. hongdechloris* PsaA2 FRL IFGQEILNADMGDGFRGIQITSGLFQMWRGEGFTHEFQLFWTAIGALVMAALMMFAGWFH 179

*F.* 7521 PsaA2 FRL IFGQEILNGDMGGGFHGIQITSGLFQMWRAAGFTNTFQLYCTAIGGLVMAALMLFAGWFH 178

*S.* 7335 PsaA1 WL VFGQEILNADVGGGFHGIQITSGLFQMWRASGITNSYQLYCTAIGGLVMAGLMLFAGWFH 180

*H. hongdechloris* PsaA1 WL IFGQEILNGDVGGGFHGIQITSGLFQMWRANGITNSFELYCTAIGALVMAGLMLFAGWFH 179

*F.* 7521 PsaA1 WL IVGQDILNGDVGGGFHGIQITSGLFQVWRGWGITNSFQLYCTAIGGLVLAGLLLFAGWFH 180

*T. elongatus* PsaA IVGQGILNGDVGGGFHGIQITSGLFQLWRASGITNEFQLYCTAIGGLVMAGLMLFAGWFH 179

*S.* 6803 PsaA IVGQGILNGDVGGGFHGIQITSGLFYLWRASGFTDSYQLYCTAIGGLVMAALMLFAGWFH 179

:.** ***.*:*.**:********* :**. *:*. ::*: ****.**:*.*::******

*S.* 7335 PsaA2 FRL YHVRSPKLEWFQNVQSMLNHHLAGLLGLGSLGWAGHLIHVALPTNKLLDAGVA------- 231

*H. hongdechloris* PsaA2 FRL YHVRAPKLDWFRNWESMMNHHLAGLLGLGSLGWAGHLIHVALPTNKLLDAGVP------- 232

*F.* 7521 PsaA2 FRL YHKRAPKLEWFQNTQSMLNHHLAGLLGLGSLGWTGHLIHVSLPTNKLLDTGVA------- 231

*S.* 7335 PsaA1 WL YHKAAPKLEWFQNVESMLNHHLAGLFGLGSLGWAGHQIHVSLPINKLLDAGVA------- 233

*H. hongdechloris* PsaA1 WL YHKKAPKLEWFQNVESMMNHHLAGLLGLGCLGYAGQQIHVSLPINACLDAIDAGKPLTVG 239

*F.* 7521 PsaA1 WL YHKRAPKLEWFQNVESMLNHHLQVLLGCGSLGWAGHIIHVSAPTNKLLDAGVA------- 233

*T. elongatus* PsaA YHKRAPKLEWFQNVESMLNHHLAGLLGLGSLAWAGHQIHVSLPINKLLDAGVA------- 232

*S.* 6803 PsaA YHVKAPKLEWFQNVESMMNHHLAGLLGLGSLGWAGHQIHVSMPINKLLDAGVA------- 232

** :***:**:* :**:**** *:* *.*.::*: ***: * * **:

*S.* 7335 PsaA2 FRL ------PQDIPLPHEFVLDKALMAELYPSF----AQGIRPFFTLNWATYSDFLTFNGGLN 281

*H. hongdechloris* PsaA2 FRL ------LEDIPLPHEFILNKSLMVDLYPSF----AEGVKPFFTLNWSAYADFLTFKGGLN 282

*F.* 7521 PsaA2 FRL ------LKDIPLPHEFILNPSLMNKLYPHADWGFVKGVVPFFTLQWGHFTDFLTFKGGLN 285

*S.* 7335 PsaA1 WL ------PQDIPLPHEFILDKALMTELYPSF----AQGLKPFFTLNWAAYSDFLTFKGGLN 283

*H. hongdechloris* PsaA1 WL GKVIDSVAAIPLPHEWILNPSLMTDIYPSF----AEGLKPFFTLNWSVYADFLTFNGGLN 295

*F.* 7521 PsaA1 WL ------VKDIPLPHEFILNSANLINLYPSF----AKGLAPFFTLNWGVYSDFLTFKGGLN 283

*T. elongatus* PsaA ------AKDIPLPHEFILNPSLMAELYPKVDWGFFSGVIPFFTFNWAAYSDFLTFNGGLN 286

*S.* 6803 PsaA ------PKDIPLPHEFILEPSKMAELYPSF----AQGLTPFFTLNWGVYSDFLTFKGGLN 282

******::*: : : .:** .*: ****::*. ::*****:****

*S.* 7335 PsaA2 FRL PVTGGLWMTDIAHHHVAIAVLFIFAGHMYRTNWGIGHSIRTMLEDARHPKMLPFLSFIGP 341

*H. hongdechloris* PsaA2 FRL PVTGGLWMTDIAHHHVAIAVLFIIAGHFYRTNWGIGHSFRELLDDARTPKMLPIFNFIGP 342

*F.* 7521 PsaA2 FRL PVTGGLWLTDVAHHHLAIAVMFIIAGHMYRTNWGIGHSIKEMLDDARTPNMLPFLSFIGP 345

*S.* 7335 PsaA1 WL PVTGGLWLSDTAHHHLAIAVMFLVAGHMYRTNWGIGHSIKQILDGHKGDPLL-----FGG 338

*H. hongdechloris* PsaA1 WL PQTGGLWLTDTAHHHLALAVLFIVAGHFYRTNWGIGHSFKEVLEAHKGP--------VTG 347

*F.* 7521 PsaA1 WL PVTGGLWMTDIAHHHLAIAVLFIIAGHQYRTNWGIGHSIKEILENHKGP--------FTG 335

*T. elongatus* PsaA PVTGGLWLSDTAHHHLAIAVLFIIAGHMYRTNWGIGHSLKEILEAHKGP--------FTG 338

*S.* 6803 PsaA PVTGGLWLSDTAHHHLAIAVLFIIAGHMYRTNWGIGHSMKEILEAHKGP--------FTG 334

* *****::* ****:*:**:*:.*** **********:: :*: : .

*S.* 7335 PsaA2 FRL VGHRGLFEVLTTSWHAQLSINLAMMGSLSIIVAQHMYSMPPYPYLATDYGTVTSLFTHHM 401

*H. hongdechloris* PsaA2 FRL VGHRGLDKIFETSWHANLAIHLVQFGTASLLVAHHMYAMPPYPYLATDYATVTSLFTHHV 402

*F.* 7521 PsaA2 FRL VGHKGLFEVLTTSWHAQLSINLAMLGSLSIIIAHHMYAMPPYPYLATDYGTVVSLFTHHV 405

*S.* 7335 PsaA1 WL EGHVGMYEFLTQSWHAQLAINLALGGSVTIIVAQHMYAMPPYPYLATDYGTQLSLFTHHM 398

*H. hongdechloris* PsaA1 WL EGHKGMYEIFTTSWHCQLSWNLAWIGSLSILVAHHMYSMPPYPYIATDYPTQLSLFTHHM 407

*F.* 7521 PsaA1 WL DGHKGLYENMTTSWHAQLGTNLAMLGSLTIIVAHHMYAMPPYPYLATDYATQLCIFTHHM 395

*T. elongatus* PsaA AGHKGLYEVLTTSWHAQLAINLAMMGSLSIIVAQHMYAMPPYPYLATDYPTQLSLFTHHM 398

*S.* 6803 PsaA EGHKGLYEILTTSWHAQLAINLALLGSLTIIVAQHMYAMPPYPYQAIDYATQLSLFTHHM 394

** *: : : ***.:*. :*. *: ::::*:***:****** * ** * .:****:

*S.* 7335 PsaA2 FRL WIGGFLIVGAAAHAGIFMVRDYDPAENVNNVLDRVLRHRDAIISHLVWVCQFLGFHSFAM 461

*H. hongdechloris* PsaA2 FRL WIAGFCIVGGAAHAAIFLVRDYNPADHVNNVLDRTLRHRDTVVSHLAWVCQFLGFHSFAM 462

*F.* 7521 PsaA2 FRL WIGGFLIVGGAAHAAIYMVRDYDPEQNFNNVLDRVLRHRDAIISHLAWVCQFLGFHSFAM 465

*S.* 7335 PsaA1 WL WIGGFLVVGAGAHGAIALIRDYDPAKHVNNVLDRVLRVRDAIISHLNWVCIFLGFHSFGL 458

*H. hongdechloris* PsaA1 WL WIGGFLIVGAGAHAAIFMVRDYDPATHINNLLDRVIRHRDAIISHLNWVCIFLGFHSFGL 467

*F.* 7521 PsaA1 WL WIGGFLIVGGAAHAAIFMVRDYDPVVNQNNVLDRVIRHRDAIISHLNWVCIFLGFHSFGL 455

*T. elongatus* PsaA WIGGFLVVGGAAHGAIFMVRDYDPAMNQNNVLDRVLRHRDAIISHLNWVCIFLGFHSFGL 458

*S.* 6803 PsaA WIGGFLIVGAGAHGAIFMVRDYDPAKNVNNLLDRMLRHRDAIISHLNWVCIFLGFHSFGL 454

**.** :**..**..* ::***:* : **:*** :* **:::*** *** *******.:

*S.* 7335 PsaA2 FRL YCHNDTMRAFGRPQDMFSDTGIQLQPIFAQWVQHIQTMAVGS-A-QVAEPLGDALGGIQN 519

*H. hongdechloris* PsaA2 FRL YCHNDTMRAFGRPQDMFSDTGIQLQPIFAQWVQQIQTMAVGA-NLQAAEPLGNVFGGLRN 521

*F.* 7521 PsaA2 FRL YCHNDTMRAFGRPQDMFSDTGIQLQPVFAQWLQHIHTMTIGNPSLQVAAPLGHAFGGLRN 525

*S.* 7335 PsaA1 WL YIHNDTMQALGRPQDMFSDTAIQLQPVFAQWIQSLHTAAP-------------------- 498

*H. hongdechloris* PsaA1 WL YVHNDTMRAFGRPQDMFSDTGIQLQPVFAQWVQNLHAAAA-------------------- 507

*F.* 7521 PsaA1 WL YVHNDTMRALGRPQDMFSDTAIQLQPVFAQWVQNLHTLAP-------------------- 495

*T. elongatus* PsaA YVHNDTMRAFGRPQDMFSDTGIQLQPVFAQWVQNLHTLAP-------------------- 498

*S.* 6803 PsaA YIHNDTMRALGRPQDMFSDTAIQLQPIFAQWVQHLHTLAP-------------------- 494

* *****:*:**********.*****:****:* ::: :

*S.* 7335 PsaA2 FRL IALSGVGTTAPGVASPASYAFGGGLVAVGGKVAMMPISLGTADFLIHHIHAFTIHVTVLV 579

*H. hongdechloris* PsaA2 FRL IDLAGVGVTAPGLGGPVSHAFGGGVVAIGDKIAMMPIQLGTADFLIHHIHAFTIHVTVLV 581

*F.* 7521 PsaA2 FRL LELTGLGTAAPNLHDPVSYAFGGGVVAVGGKVAMMPITLGTADFLIHHIHAFTIHVTVLV 585

*S.* 7335 PsaA1 WL ----SISGTAPNALAPVSYAFGGDVVAVGGKVAMMPITLGTADFMVHHIHAFTIHVTVLI 554

*H. hongdechloris* PsaA1 WL ----G--GTAPNAAAGVSPAFGGDILAVVGKVAMMPITLGTADFLVHHIHAFTIHVTVLI 561

*F.* 7521 PsaA1 WL ----G--ATAPNALEPVSYAFGGGILAVGGKVAMMPIALGTADFMIHHIHAFQIHVTTLI 549

*T. elongatus* PsaA ----G--GTAPNAAATASVAFGGDVVAVGGKVAMMPIVLGTADFMVHHIHAFTIHVTVLI 552

*S.* 6803 PsaA ----G--ATAPNALATASYAFGGETIAVAGKVAMMPITLGTADFMVHHIHAFTIHVTALI 548

. :**. .* **** :*: .*:***** ******::****** ****.*:

*S.* 7335 PsaA2 FRL LLKGVLFARNSRLIPDKSELGFRFPCDGPGRGGTCQVSAWDHVFLGLFWMYNSIAMVIFH 639

*H. hongdechloris* PsaA2 FRL LLKGVLFSRNSRLIPDKGELGFRFPCDGPGRGGTCQVSAWDHVFLGLFWMYNSLSIVIFH 641

*F.* 7521 PsaA2 FRL LLKGVLFARSSRLIPDKANLGFRFPCDGPGRGGTCQVSAWDHVFLGLFWMYNSLSMVIFH 645

*S.* 7335 PsaA1 WL LLKGVLYARNSRLIPDKSELGFRFPCDGPGRGGTCQVSAWDHVFLGLFWMYNSISIVIFH 614

*H. hongdechloris* PsaA1 WL LLKGVLFARNSRLIPDKGELGFRFPCDGPGRGGTCQVSGWDHVFLGLFWMYNSLSIVIFH 621

*F.* 7521 PsaA1 WL LLKGFLFARSSRLIPDKANLGFRFPCDGPGRGGTCQVSGWDHVFLGLFWMYNTISIAIFH 609

*T. elongatus* PsaA LLKGVLFARSSRLIPDKANLGFRFPCDGPGRGGTCQVSGWDHVFLGLFWMYNCISVVIFH 612

*S.* 6803 PsaA LLKGVLYARSSRLVPDKANLGFRFPCDGPGRGGTCQVSGWDHVFLGLFWMYNSLSIVIFH 608

****.*::*.***:***.:*******************.************* :::.***

*S.* 7335 PsaA2 FRL FFWKMQSDVWGAVDANGTVSHITGGNFAQSSITINGWLRDFLWAQATQVISSYGSALSAY 699

*H. hongdechloris* PsaA2 FRL FFWKMQSDVWGTVGADGTISHITGGNFAQASITNNGWLRDFLWAQASQVIGSYGSALSAY 701

*F.* 7521 PsaA2 FRL FFWKMQSDVWGTVGADGVVTHITGGNFATSSITNNGWLRDFLWAQSTQVITSYNTSLSAY 705

*S.* 7335 PsaA1 WL FSWKMQSDVWGTVSDNGTVSHITGGNFAASATTINGWLRDFLWAQASQVINTYGSALSAY 674

*H. hongdechloris* PsaA1 WL FSWKMQSDVWGSVSPDGSVSHITAGNFAQSAITINGWLRDFLWAQASQVIGSYGSALSAY 681

*F.* 7521 PsaA1 WL FSWKMQSDVWGTVDAAGNVSHVTGGNFAQSAITINGWLRDFLWAQATQVINSYGSALSAY 669

*T. elongatus* PsaA FSWKMQSDVWGTVAPDGTVSHITGGNFAQSAITINGWLRDFLWAQASQVIGSYGSALSAY 672

*S.* 6803 PsaA FSWKMQSDVWGTVSPDGSVTHVTLGNFAQSAITINGWLRDFLWAQAANVINSYGSALSAY 668

* *********:* * ::*:* **** :: * ***********:::** :*.::****

*S.* 7335 PsaA2 FRL GLMFLAGHFVFAFSLMFLFSGRGYWQELIESIVWAHNKLRITTAIQPRALSITQGRAVGA 759

*H. hongdechloris* PsaA2 FRL GLFFLAGHFIFGFSLMFLFSGRGYWQELIESIVWAHNKLKITTAIQPRALSITQGRAVGV 761

*F.* 7521 PsaA2 FRL GLMFLGGHFIFGFSLMFLFSGRGYWQELIESIVWAHNKLKVAPAIQPRALSIIHGRAVGV 765

*S.* 7335 PsaA1 WL GLMFLGAHFVWAFSLMFLFSGRGYWQELIESIVWAHNKLKVAPAIQPRALSITQGRAVGV 734

*H. hongdechloris* PsaA1 WL GLLFLGAHFVWAFSLMFLFSGRGYWQELIESIVWAHNKLKVAPAIQPRALSITQGRAVGV 741

*F.* 7521 PsaA1 WL GLMFLGAHFIWAFSLMFLFSGRGYWQELIESIVWAHNKLKVAPAIQPRALSIIQGRAVGV 729

*T. elongatus* PsaA GLLFLGAHFIWAFSLMFLFSGRGYWQELIESIVWAHNKLKVAPAIQPRALSIIQGRAVGV 732

*S.* 6803 PsaA GIMFLAGHFVFAFSLMFLFSGRGYWQELIESIVWAHNKLNVAPAIQPRALSIIQGRAVGV 728

*::**..**::.***************************.:: ********* :*****.

*S.* 7335 PsaA2 FRL AHYLLGSIVTTWAFFLARMAAIG 782

*H. hongdechloris* PsaA2 FRL AHYLLGGIVTTWAFFLARMAAIG 784

*F.* 7521 PsaA2 FRL AHYLLGGIVTTWAFFLARMTAFG 788

*S.* 7335 PsaA1 WL AHYLLGGIATTWAFFLARMLSVG 757

*H. hongdechloris* PsaA1 WL AHFLLGGIATTWAFFLARIIAVG 764

*F.* 7521 PsaA1 WL AHYLLGGIATTWAFFHAHILSIG 752

*T. elongatus* PsaA AHYLLGGIATTWAFFLARIISVG 755

*S.* 6803 PsaA AHYLLGGIVTTWAFFLARSLSIG 751

**:***.*.****** *: :.*

PsaB

*S*. 7335 PsaB2 FRL MATKFPKFSQELQQDPTTRRIFYSLATAHDFESHDGMTEESLYQRIFASHFGHLAIIFLW 60

*H. hongdechloris* PsaB2 FRL MATKFPKFSQDLQRDPTTRRLFYAIATAHDFESHDGMSEENLYQRIFASHFGHLAIIFLW 60

*F*. 7521 PsaB2 FRL MATKFPKFSQDLANDPTTRRIFYAIATAHDFESHDGMTEENLYQRIFASHFGHLAIIFLW 60

*S*. 7335 PsaB1 WL MATKFPKFSQALAQDPTTRRIWYGIATAHDFETHDGMTEENLYQKIFASHFGHLAIIFLW 60

*H. hongdechloris* PsaB1 WL MATKFPKFSQDLASDPTTRRIWYGIATAHDFESHDGMTEENLYQKIFASHFGHLAIIFLW 60

*F*. 7521 PsaB1 WL MATKFPKFSQDLAQDPTTRRIWYAMAMGNDFESHDGMTEENLYQKIFATHFGHLAIIFLW 60

*T. elongatus* PsaB MATKFPKFSQDLAQDPTTRRIWYAIAMAHDFESHDGMTEENLYQKIFASHFGHLAIIFLW 60

*S*. 6803 PsaB MATKFPKFSQDLAQDPTTRRIWYGIATAHDFETHDGMTEENLYQKIFASHFGHIAIIFLW 60

********** * ******::*.:* .:***:****:**.***:***:****:******

*S*. 7335 PsaB2 FRL TSGILFHVAWQGNFEAWIKDPLNISPIAHAIWDPQFGPAAMDAFTPAGAGNPVNFCYSGV 120

*H. hongdechloris* PsaB2 FRL ISGILFHVAWQGNFEQWIQDPLNNSPIAHAIWDAQFGPPAIAAYTQAGAMNPVDICYSGV 120

*F*. 7521 PsaB2 FRL ASGILFHVAWQGNFEVWIKDPVHVRPIAHAIWDAQFGSGAIKAFTQAGARNPVDICYSGV 120

*S*. 7335 PsaB1 WL TSGNLFHVAWQGNFPQWTQDPLNVKPIAHAIWDPHFGQPAVDAFSQAGSTSPVNIAYSGV 120

*H. hongdechloris* PsaB1 WL TSGNLFHVAWQGNFQQWVKDPLNISPIAHAIWDPQFGQSAVEAFSQAGANYPVDIAYSGV 120

*F*. 7521 PsaB1 WL ASSLLFHVAWQGNFEQWIKDPLHIRPIAHAIWDPHFGKPAIEAFTQGGASNPVNIAYSGV 120

*T. elongatus* PsaB VSGSLFHVAWQGNFEQWVQDPVNTRPIAHAIWDPQFGKAAVDAFTQAGASNPVDIAYSGV 120

*S*. 6803 PsaB TSGTLFHVAWQGNFEQWIKDPLNIRPIAHAIWDPHFGEGAVNAFTQAGASNPVNIAYSGV 120

*. ********** * :**:: ******** :** *: *:: .*: **::.****

*S*. 7335 PsaB2 FRL YHWWYTIGLRTNGDLFAGAMFLLLLAAVMLYAGWLHLQPRYRPSLAWFKNAESRLNHHLA 180

*H. hongdechloris* PsaB2 FRL YHWWYTIGMRTNNDLFMGSIFLLLLSSVMLYAGWLHLQPRFRPGLAWFKNAESRLNHHLA 180

*F*. 7521 PsaB2 FRL YHWWYTIGLRTNTELYVGALFLILLAAVFLFAGWLHLQPRYRPNLGWFKNSEARLNHHLA 180

*S*. 7335 PsaB1 WL YHWWYTIGMRTNGDLYAGAVGLLIFAAVMLFAGWLHLQPKFRPSLSWFKNAESRLNHHLA 180

*H. hongdechloris* PsaB1 WL YHWWYTIGMRTAGDLYGGALFLMIMAAVFLFAGWLHLQPRFRPSLAWFKNAESRMNHHLA 180

*F*. 7521 PsaB1 WL YHWWYTIGMRTNGDLYMGSIFLLVLSSLFLFAGWLHLQPKFRPSLAWFKMAESRLNHHLA 180

*T. elongatus* PsaB YHWWYTIGMRTNGDLYQGAIFLLILASLALFAGWLHLQPKFRPSLSWFKNAESRLNHHLA 180

*S*. 6803 PsaB YHWFYTIGMTTNQELYSGAVFLLVLASLFLFAGWLHLQPKFRPSLAWFKNAESRLNHHLA 180

***:****: * :*: *:: *:::::: *:********::**.*.*** :*:*:*****

*S*. 7335 PsaB2 FRL GLFGVSSLAWTGHLVHVAIPESRGQHVGWDNFLSMPPHPEGLKPFFTGNWGAYALNPDTS 240

*H. hongdechloris* PsaB2 FRL GLFGVSSLAWTGHLVHVALPESRGQHVGWDNFLSIRPHPEGLAPLFTGNWGAYAQNPDTA 240

*F*. 7521 PsaB2 FRL GLFGVSSLAWAGHLVHVAIPESRGQHVGWDNFLSTPPHPAGLWAFFTGNWGAYAQNPDTA 240

*S*. 7335 PsaB1 WL GLFGVSSLAWTGHLVHVAIPESRGVHVGWDNFLTMKPHPAGLQPFFTGNWGAYAQNPDTA 240

*H. hongdechloris* PsaB1 WL GLFGVSSLAWAGHLIHVAIPESRGQHVGWDNFLFTPPHPAGLTPFFTGNWGVYAQNPDTA 240

*F*. 7521 PsaB1 WL GLFGVSSLAWTGHLVHVAIPESRGQHVGWDNFLSTLPHPAGLQPFFTGNWGVYAENPDTA 240

*T. elongatus* PsaB GLFGVSSLAWAGHLIHVAIPESRGQHVGWDNFLSTMPHPAGLAPFFTGNWGVYAQNPDTA 240

*S*. 6803 PsaB GLFGVSSLAWAGHLVHVAIPEARGQHVGWDNFLSTPPHPAGLMPFFTGNWGVYAADPDTA 240

**********:***:***:**:** ******** *** ** :******.** :***:

*S*. 7335 PsaB2 FRL EHLFNTSQGAGTAILTFLGGFHPQTESLWLTDMAHHHLAIAVIFIIAGHMYRTNFGIGHS 300

*H. hongdechloris* PsaB2 FRL EHAFGTAQGAGSAILTFLGGFHPQTESLWLTDMAHHHLAIAVIFIVAGHMYRTNFGIGHN 300

*F*. 7521 PsaB2 FRL EHVFSTSQGAGTAILTFLGGFHPQTQSLWLTDMAHHHLAIAVVLIIAGHMYRTNWRIGHS 300

*S*. 7335 PsaB1 WL EHVFGTSQGAGDAILTFLGGFHPQTQSLWLTDMAHHHLAIAVLFIVAGHMYRTNFGIGHS 300

*H. hongdechloris* PsaB1 WL SHVFGSSTGAGSAILTFLGGFHPQTESLWLTDMAHHHLAIAVLFIVAGHMYRTNFGIGHN 300

*F*. 7521 PsaB1 WL SHVFGTSQGAGTAILTFLGGFHPQTESLWLTDMAHHHLAIAVLFIVAGHMYRTNFGIGHS 300

*T. elongatus* PsaB SHVFGTAQGAGTAILTFLGGFHPQTESLWLTDMAHHHLAIAVLFIVAGHMYRTQFGIGHS 300

*S*. 6803 PsaB GHIFGTSEGAGTAILTFLGGFHPQTESLWLTDIAHHHLAIAVIFIIAGHMYRTNWGIGHS 300

* *.:: *** *************:******:*********::*:*******:: ***.

*S*. 7335 PsaB2 FRL IKEMTESLQGPGWTGFFIAPNTGRGHKGIYDAYNNSLHFQLGWHLACLGVVTSLVAQHMY 360

*H. hongdechloris* PsaB2 FRL IKEMTEALQGPGRSGFFIAPRTGRGHKGIYDTYNNSLHFQLGWHLACLGVITSLVAQHMY 360

*F*. 7521 PsaB2 FRL IKEMMDSKT-FF--GRKVEGPFNLPHQGLYETVNNSLHFQLSLALACLGVASSLTAQHMY 357

*S*. 7335 PsaB1 WL IREILNTHRPPE--GTPLGGALGAGHKGLYDTLNNSLHFQLALALASLGVITSLVAQHMY 358

*H. hongdechloris* PsaB1 WL MKEIMNAHNPPQ--GTPFGGMIGEGHKGMYDTYNNSLHFQLGWHLACLGVITSLVAQHMY 358

*F*. 7521 PsaB1 WL IKEMMDAKT-FF--GKPVEGPFNMPHQGIYETYNNSLHFQLGWHLACLGVITSLVAQHMY 357

*T. elongatus* PsaB IKEMMDAKD-FF--GTKVEGPFNMPHQGIYETYNNSLHFQLGWHLACLGVITSLVAQHMY 357

*S*. 6803 PsaB IKEILNAHKGP---------LTGAGHTNLYDTINNSLHFQLGLALASLGVITSLVAQHMY 351

::*: :: . * .:*:: ********. **.*** :**.*****

*S*. 7335 PsaB2 FRL AMPPYAFIARDYTTTAALYTHHQYIAGFLMLGAFAHGGIFLIRDYDPVANENNVLARVLD 420

*H. hongdechloris* PsaB2 FRL AMPPYAFMARDYTTMSALYTHHQYIAGFLMIGAFAHGAIFLIRDYDPEANRDNVLARMLA 420

*F*. 7521 PsaB2 FRL SMPPYAFIAKDFTTMAALYTHHQYIAGFLMVGAFSHAAIFWIKDYDPEQNKGNVLERVLK 417

*S*. 7335 PsaB1 WL ALPPYAFIAQDFTTQAALYTHHQYIAGFIMMGAFAHGAIFLIRDYDPAANENNVLARVLD 418

*H. hongdechloris* PsaB1 WL SLPPYAFLAKSYTTQAALYTHHQYIAGFIMVGAFAHGAIFLIRDYDPASNSNNVLDRVLQ 418

*F*. 7521 PsaB1 WL SLPPYAFIAKDYTTQAALYTHHQYIAIFLMLGAFAHGAIFWVRDYDPEQNKGNVLERVLK 417

*T. elongatus* PsaB SLPPYAFIAQDHTTMAALYTHHQYIAGFLMVGAFAHGAIFLVRDYDPAQNKGNVLDRVLQ 417

*S*. 6803 PsaB SLPSYAFIAQDHTTQAALYTHHQYIAGFLMVGAFAHGAIFFVRDYDPVANKDNVLARMLE 411

::* ***:*:..** :********** *:*:***:*..** ::**** * .*** *:*

*S*. 7335 PsaB2 FRL HKEAIISHLSWVSLFLGFHTLALYVHNDCEVAFGSPDKQILVEPVFAQWIQAVHGKALYG 480

*H. hongdechloris* PsaB2 FRL HKEAIISHLSWVSLFLGFHTLGLYVHNDCEVALGSPEKQILIEPVFAQWTQAFHGKALYG 480

*F*. 7521 PsaB2 FRL HKEAIIAHLSWVSLFLGFHTLGLYVHNDVEVAFGAADKQILIEPVFAQFIQSANGKILYG 477

*S*. 7335 PsaB1 WL HKEAIISHLSWVSLFLGFHTLGLYVHNDVVVAFGTPEKQILVEPVFAQWVQAASGKALYG 478

*H. hongdechloris* PsaB1 WL HKEAIISHLSWVSLFLGFHTLGLYVHNDVMVAFGTPEKQILVEPVFAQWIQAAHGKLLYG 478

*F*. 7521 PsaB1 WL HKEAIISHLSWVSLFLGFHTLGLYVHNDVVVAFGTPEKQILIEPVFAQFIQASHGKVLYG 477

*T. elongatus* PsaB HKEAIISHLSWVSLFLGFHTLGLYVHNDVVVAFGTPEKQILIEPVFAQFIQAAHGKLLYG 477

*S*. 6803 PsaB HKEALISHLSWVSLFLGFHTLGLYVHNDVVVAFGTPEKQILIEPVFAQWIQATSGKALYG 471

****:*:**************.****** **:*: :****:******: *: ** ***

*S*. 7335 PsaB2 FRL ISSLLSNPDSIASTAWPNHANVWLPGWLEAINNGTNSLFLAIGPGDFLVHHAIALGLHVT 540

*H. hongdechloris* PsaB2 FRL INSLLSNPDSVASTAWPNYGNVWLSGWLEAVNNGANSLFLTIGPGDLLVHHAIALGLHVT 540

*F*. 7521 PsaB2 FRL FHTLLSNPDSIAFTAWPNHANVWLPGWLDAINDGTNSLFLTIGPGDFYVHHAIALGLHVT 537

*S*. 7335 PsaB1 WL FDTLLSNPDSIATTAWPNGGNVWLPGWLDAINSGSNSLFLAIGPGDFLVHHAIALGLHTT 538

*H. hongdechloris* PsaB1 WL FDTLLSNPGSIASTAWPNYGNVWLSGWLDAINSGDNSLFLTIGPGDFLVHHAIALGLHTT 538

*F*. 7521 PsaB1 WL LNVLLSNPDSVAYTAYPNYGNVWLSGWLDAINSGTNSLFLTIGPGDFLVHHAFALAIHTT 537

*T. elongatus* PsaB FDTLLSNPDSIASTAWPNYGNVWLPGWLDAINSGTNSLFLTIGPGDFLVHHAIALGLHTT 537

*S*. 6803 PsaB FDVLLSNPDSIAST----TGAAWLPGWLDAINSGTNSLFLTIGPGDFLVHHAIALGLHTT 527

: *****.*:* * . .** ***:*:*.* *****:*****: ****:**.:*.*

*S*. 7335 PsaB2 FRL TLILVKGALDARGSKLMPDKKDFGYAFPCDGPGRGGTCDISAWDSVYLATFWMLNTLGWV 600

*H. hongdechloris* PsaB2 FRL TLILVKGALDARGSKLMPDKKDFGYSFPCDGPGRGGTCDISAWDAFYLATFWMLNTLGWV 600

*F*. 7521 PsaB2 FRL TLILVKGALDARGSKLMPDKKDFGYAFPCDGPGRGGTCDISAWDASYLAVFWMLNTLGWV 597

*S*. 7335 PsaB1 WL TLILVKGALDGRGSKLMPDKKDFGYSFPCDGPGRGGTCDISGWDSFYLAMFWMLNTIGWV 598

*H. hongdechloris* PsaB1 WL TLILVKGALDARGSKLMPDKKDFGYSFPCDGPGRGGTCDISAWDAFYLAVFWMLNTIGWV 598

*F*. 7521 PsaB1 WL TLVLVKGALDARGSKLMPDKKDFGYAFPCDGPGRGGTCDISAWDAFYLATFWALNTVGWV 597

*T. elongatus* PsaB TLILVKGALDARGSKLMPDKKDFGYAFPCDGPGRGGTCDISAWDAFYLAMFWMLNTIGWV 597

*S*. 6803 PsaB ALILIKGALDARGSKLMPDKKDFGYSFPCDGPGRGGTCDISAWDAFYLAMFWMLNTLGWL 587

:*:*:*****.**************:***************.**: *** ** ***:**:

*S*. 7335 PsaB2 FRL TFYWHWKHLAIWSGNVAQFNEGSTYLMGWFRDYLWLNSAQLINGYNPYGTNNLAIWAWIF 660

*H. hongdechloris* PsaB2 FRL TFYWHWKHLSVWSGNVAQFNESSTYLMGWFRDYLWANSAQLINGYSPAGTNSLAVWAWMF 660

*F*. 7521 PsaB2 FRL TFYWHWKHLSIWQGNVAQFNESSTYLMGWFRDYLWANSAQLINGYNPYGTSNLAVWAWMF 657

*S*. 7335 PsaB1 WL TFYWHWKHLAIWSGNVAQFNESSNYLMGWLRDYLWLNSSQLINGYNPYGMNNLAVWAWMF 658

*H. hongdechloris* PsaB1 WL TFYWHWKHLAIWQGNVAQFNESSTYLMGWLRDYLWLNSSQLINGYNPYGMNNLAVWAWMF 658

*F*. 7521 PsaB1 WL TFYWHWKHLGIWQGNVAQFNESSTYLMGWFRDYLWANSAQLINGYNPYGMNNLSVWAWMF 657

*T. elongatus* PsaB TFYWHWKHLGVWEGNVAQFNESSTYLMGWLRDYLWLNSSQLINGYNPFGTNNLSVWAWMF 657

*S*. 6803 PsaB TFYWHWKHLGVWSGNVAQFNENSTYLMGWFRDYLWANSAQLINGYNPYGVNNLSVWAWMF 647

*********.:*.********.*.*****:***** **:******.* * ..*::***:*

*S*. 7335 PsaB2 FRL LFGHLVWAISFMFLITWRGYWQELIETLMWAHENTPLS-FGYPKDKPVALSIVQARLVGL 719

*H. hongdechloris* PsaB2 FRL LFGHLAWAVSFMFLITWRGYWQELIETLMWAHENTPLS-FGYPKDKPVALSIVQARLVGL 719

*F*. 7521 PsaB2 FRL LFGHLAWAVSFMFLITWRGYWQELIETLAWAHEQTPLS-FGYWRDKPVALSIVQARLVGL 716

*S*. 7335 PsaB1 WL LFGHLVWATGFMFLISWRGYWQELIETIVWAHERTPLANLVRWKDKPVAMSIVQGRLIGL 718

*H. hongdechloris* PsaB1 WL LLGHLVWATGFMFLISWRGYWQELIETLVWAHERTPLANLVRWKDKPVALSIVQARLVGL 718

*F*. 7521 PsaB1 WL LFGHLVWATGFMFLISWRGYWQELIETLVWAHERTPIANLVRWKDKPVALSIVQARLVGL 717

*T. elongatus* PsaB LFGHLVWATGFMFLISWRGYWQELIETLVWAHERTPLANLVRWKDKPVALSIVQARLVGL 717

*S*. 6803 PsaB LFGHLVWATGFMFLISWRGYWQELIETIVWAHERTPLANLVRWKDKPVALSIVQARLVGL 707

*:***.** .*****:***********: ****.**:: : :*****:****.**:**

*S*. 7335 PsaB2 FRL VHFTVGYIATYGAFLIASTGSRFP- 743

*H. hongdechloris* PsaB2 FRL THFTVGYIATYGAFLIASTSSRFP- 743

*F*. 7521 PsaB2 FRL THFTVGYIATYGAFLIASTASKFGQ 741

*S*. 7335 PsaB1 WL AHFTVGYVLTYAAFLIASTSSRFG- 742

*H. hongdechloris* PsaB1 WL AHFSVGYIITYAAFLIASTSSRFG- 742

*F*. 7521 PsaB1 WL VHFSVGYVLTYAAFLIASTAGKFG- 741

*T. elongatus* PsaB AHFSVGYILTYAAFLIASTAAKFG- 741

*S*. 6803 PsaB AHFTVGYVLTYAAFLIASTAGKFG- 731

.**:***: **.*******..:*

PsaF

*S*. 7335 PsaF2 FRL MHKTIRKFFSLLLAAFVWLSVVSPAVAASEGYTDTHLVPCASSPAFNERMQNAPEGYYF- 59

*H. hongdechloris* PsaF2 FRL ----MRALFAIVIMSFVWFNAIPPAIAA----GDTHLVPCQESTAFMENLQNAPKSYYF- 51

*F*. 7521 PsaF2 FRL ----MKRIFALILAIFIWFSAVSTALAE-----NTTLVPCYKSPAFVERMKNAPDSYYT- 50

*S*. 7335 PsaF1 WL ----MRRLFALALVLCLSLGFAAPATAGIAGDDVAGLVPCNESAAFQKRAAAAPTDE--- 53

*H. hongdechloris* PsaF1 WL ----MRRLFALVLGIISWFGIALPAA-----ADVAGLTPCSESAAFQQRAANAATEA--- 48

*F*. 7521 PsaF1 WL ----MRRLFALILAICLWFNFAPAAN-----ALGADLVPCSESSAFAQRAQVARNTTADP 51

*T. elongatus* PsaF ----MRRFLALLLVLTLWLGFTPLAS-----ADVAGLVPCKDSPAFQKRAAAAVNTTADP 51

*S*. 6803 PsaF ----MKHLLALLLAFTLWFNFAPSASA----DDFANLTPCSENPAYLAKSKNFLNTTNDP 52

:: :::: : :. * : *.** .. *: .

*S*. 7335 PsaF2 FRL ---DTPYQSYAANLLCGAEGLPHQQL--RFDRAIDVLIPFGIFFYVAGFIGWSGRAYLIS 114

*H. hongdechloris* PsaF2 FRL ---DQPYQAYSKNLLCGEDGLPHLQL--RLDRAVDIAIPFVIFFYFAGFVGWSGRAYLIN 106

*F*. 7521 PsaF2 FRL ---TKPLKAYS-QLLCGEDGLPRIALD-RLSLAVDVAIPIAIFLYTAGFIGWSGRSYLQA 105

*S*. 7335 PsaF1 WL AKARFEFYGN-TSLLCGPEGLPHLVVDGDLAHAGEFLIPSLLFLLIAGWIGWAGRSYVIA 112

*H. hongdechloris* PsaF1 WL AKARFDFYGS-SNLLCGDDGLPHLIVDGDLSHVGEFLIPSILFLYIAGWIGWAGRSYLIA 107

*F*. 7521 PsaF1 WL QSGQKRFERY-SQAYCGPEGLPHLIVDGRLDRAGDFLIPSILFLYIAGWIGWVGRAYLQT 110

*T. elongatus* PsaF ASGQKRFERY-SQALCGEDGLPHLVVDGRLSRAGDFLIPSVLFLYIAGWIGWVGRAYLIA 110

*S*. 6803 PsaF NSGKIRAERY-ASALCGPEGYPHLIVDGRFTHAGDFLIPSILFLYIAGWIGWVGRSYLIE 111

. ** :* *: : : . :. ** :*: **::** **:*:

*S*. 7335 PsaF2 FRL SNRN-SKPEETEIFIDVALAIKSFVQGLLWPLLAVKELTTGELTAPVSEVSVSPR 168

*H. hongdechloris* PsaF2 FRL SKKA-SKPEEMEIFINVPLAIQSFIQGLLWPLAAFRELTAKELTAKDSELSVSPR 160

*F*. 7521 PsaF2 FRL IKKQ-DKAEEKEVFIDVPLFISCMVMALFWPMAVIKELLAGELVAKDEEIPISVR 159

*S*. 7335 PsaF1 WL VRSE-KSPEEKEIVIDVPLAIKCSLSGATWPLLAFKEITSGEMFAKKEEITVSPR 166

*H. hongdechloris* PsaF1 WL VRSE-KKPEEKEIIIDVPLALKCSLSGFAWPLTAFRDIASGEMFAKDTEIPVSPR 161

*F*. 7521 PsaF1 WL IKKQGGDVEQKEIQIDVPLALPIMLSGFAWPAAAIKELLSGELTAKDEEIPISPR 165

*T. elongatus* PsaF VRNS-GEANEKEIIIDVPLAIKCMLTGFAWPLAALKELASGELTAKDNEITVSPR 164

*S*. 6803 PsaF IRES-KNPEMQEVVINVPLAIKKMLGGFLWPLAAVGEYTSGKLVMKDSEIPTSPR 165

. . : *: *:* * : : . ** .. : : :: *: * *

PsaI

*S*. 7335 PsaI2 FRL MVDATQLEGAYAAAWLPWIMIPMITYILPFPIFAIAFLWIEREGGEGGLDID----VMGS 56

*H. hongdechloris* PsaI2 FRL MADMTQLTGAYAAPWLPWIMIPLIFYILPFPIFAIIFLWIEREGNGV----N----DMGG 52

*F*. 7521 PsaI2 FRL MVDMTQLTGDYAASWLPWIMIPLVFYILPFPVFAILFLWIQKEASEEIKETDNNLAEIGE 60

*S*. 7335 PsaI1 WL ----------MSASFLPTILVPTVGLVFPAIAMAALFLYIERGQATTGGESA----PWGQ 46

*H. hongdechloris* PsaI1 WL ------MDGTYAASWLPWLLIPVVTWLMPAVVMGLLFFYIESDA---------------- 38

*S*. 7521 PsaI1 WL ---MASLLASYPASFLSPILVYSIGWIVPIVVFSFMLIYIEREDIA-------------- 43

*T. elongatus* PsaI ------MMGSYAASFLPWIFIPVVCWLMPTVVMGLLFLYIEGEA---------------- 38

*S*. 6803 PsaI ------MDGSYAASYLPWILIPMVGWLFPAVTMGLLFIHIESEGEG-------------- 40

* :* ::: : :.* :. :: *:

*S*. 7335 PsaI2 FRL NAMSNEAMGRDISS 70

*H. hongdechloris* PsaI2 FRL EPMKSDGNYPV--- 63

*F*. 7521 PsaI2 FRL LEVPNS-------- 66

*S*. 7335 PsaI1 WL VSE--DSQTDVV-- 56

*H. hongdechloris* PsaI1 WL -------------- 38

*S*. 7521 PsaI1 WL -------------- 43

*T. elongatus* PsaI -------------- 38

*S*. 6803 PsaI -------------- 40

PsaJ

*S*. 7335 PsaJ2 FRL ---MKYFAKYLTSAPIMATVALVSLSVVLIELNHFFPGLQYGTYFHSVP 46

*H. hongdechloris* PsaJ2 FRL ---MSYFVKYLTSAPVMATLALVILSVVMIELNHIFPGLQYGTYFHVAP 46

*F*. 7521 PsaJ2 FRL -MEARYLFRYLSSAPVVATLALIIISVILIVLNYLFPGLQYGTFFHSLP 48

*S*. 7335 PsaJ1 WL --MSSNLLKYLSTAPVIATVWMVITAGILIEFNRFFPDLL----LHP-- 41

*H. hongdechloris* PsaJ1 WL ---MDNLVKYLSTAPVVAAIWMAITAGVLIEFNRFFPDLL----FHP-- 40

*F*. 7521 PsaJ1 WL MDNQSPFFKFLSTAPVITTIWLFITAGILIEFNRFFPDLL----FHPLP 45

*T. elongatus* PsaJ ---MKHFLTYLSTAPVLAAIWMTITAGILIEFNRFYPDLL----FHPL- 41

*S*. 6803 PsaJ ---MDGLKSFLSTAPVMIMALLTFTAGILIEFNRFYPDLL----FHP-- 40

: :*::**:: : : ::* :* ::*.* :*

PsaL

*S*. 7335 PsaL2 FRL --------MSASDAYISDDPIQPYQGNPQLGNLATPINSSNLAKAFINNLPAYRPGLTPF 52

*H. hongdechloris* PsaL2 FRL --MTNTETSTWVDAYDQKDIIQPYRGNPELGNLATPVNSSNLVKTYINNLPAYRPGLTPF 58

*F*. 7521 PsaL2 FRL --------MSNAVDTVDNDIIKPFKGDPCLGNLSTPINDSPLAKAFINNLPAYRKGLTPF 52

*S*. 7335 PsaL1 WL -------------MPASSNFIKPYEGDPQIGNLETPLNSSGLSKAFLENLPAYRTGLSAQ 47

*H. hongdechloris* PsaL1 WL ---------------MTNQVVKPYLDEPELGHLSTPISDSAFVRSFIGNLPAYRKGMAPI 45

*F*. 7521 PsaL1 WL MAQAVDASKNLPSDPRNREVVFPATRDPQIGNLETPINSSALTKWFINNLPAYRPGITPF 60

*T. elongatus* PsaL ---------------MAEELVKPYNGDPFVGHLSTPISDSGLVKTFIGNLPAYRQGLSPI 45

*S*. 6803 PsaL -------------MAESNQVVQAYNGDPFVGHLSTPISDSAFTRTFIGNLPAYRKGLSPI 47

: : :* :*:* **:..* : : :: ****** *::

*S*. 7335 PsaL2 FRL LRGLEIGMAHGYFLVGPEVVFGPLKEGSHGANLSGLITAIYITVSACLGISIFALATFQG 112

*H. hongdechloris* PsaL2 FRL LRGLEIGMAHGYFLVGPEVVVGPLRETAHGANLSGLITAIYITVSACLGISIFALATFQG 118

*F*. 7521 PsaL2 FRL MRGLEIGMAHGYFLVGPEVVIGPLRESAHGANLSGLITAIYIAVSACLGISIFAITTFQG 112

*S*. 7335 PsaL1 WL RRGLEVGMAHGYLLYGPFALLGPLRDTD-VLGITGLLSAIGLVLILTVCLSIYGGADVSS 106

*H. hongdechloris* PsaL1 WL TRGLEIGLAHGYFLVGPEIIVGALRDYAPAPYLGGLVTAIAIVLLGTTGMGAHGLVSLKP 105

*F*. 7521 PsaL1 WL RRGLEVGMAHGYWIFGPFAKLGPLRNTV-NADLAGLLSTIGLLVILTIALSLYANSNPPE 119

*T. elongatus* PsaL LRGLEVGMAHGYFLIGPWVKLGPLRDSD-VANLGGLISGIALILVATACLAAYGLVSFQK 104

*S*. 6803 PsaL LRGLEVGMAHGYFLIGPWTLLGPLRDSE-YQYIGGLIGALALILVATAALSSYGLVTFQG 106

****:*:**** : ** .* *:: : **: : : : :. ..

*S*. 7335 PsaL2 FRL DPRGTYNSHSRDRLRPLRKKEDWYQLSGGILMGSLGGAIFAYALLENFELLDSILRGAVN 172

*H. hongdechloris* PsaL2 FRL DPRGAYNSNSPDRLRPLRSKDGWFQLSGGILLGSMGGAIFAYVLLENFGDLDAILRGAVN 178

*F*. 7521 PsaL2 FRL NPKGSYSSYSKDSLRPLRTREEWSQLNGGIFLGAMGGAIFAYLLLENFDALDAILRGAVN 172

*S*. 7335 PsaL1 WL EI--SRNTLPYQPPEALSTDEGWSEFAGSFLIGGIGGAIFAYFLSANLPLLLGSIAGA-- 162

*H. hongdechloris* PsaL1 WL VAES------SPKTDALMTSEGWSEMTAGFFLGGMSGAFMAYFLLSHFSEIDAIFRGFVN 159

*F*. 7521 PsaL1 WL PV--ASVTA-PHPSDAFHTKEGWSNFGSAFLIGGIGGAVTAYFLTANFGLIQGFFG---- 172

*T. elongatus* PsaL GG---------SSSDPLKTSEGWSQFTAGFFVGAMGSAFVAFFLLENFSVVDGIMTGLFN 155

*S*. 6803 PsaL EQ---------GSGDTLQTADGWSQFAAGFFVGGMGGAFVAYFLLENLSVVDGIFRGLFN 157

: . : * :: ..:::*.:..*. *: * :: : . :

*S*. 7335 PsaL2 FRL VG--------- 174

*H. hongdechloris* PsaL2 FRL VSQWLGGGVMG 189

*F*. 7521 PsaL2 FRL AS--------- 174

*S*. 7335 PsaL1 WL ----------- 162

*H. hongdechloris* PsaL1 WL ----------- 159

*F*. 7521 PsaL1 WL ----------- 172

*T. elongatus* PsaL ----------- 155

*S*. 6803 PsaL ----------- 157

**Fig. S11. Sequence alignment comparing FRL sequences to WL and non-FaRLiP sequences.** Far-red light isoform sequences are colored dark red. *T. elongatus* and *Synechocystis* sp. PCC 6803 do not exhibit FaRLiP. For the *Synechococcus* 7335 sequences, residues unable to be modeled are highlighted in grey. Abbreviations: *Synechococcus* 7335, *S.* 7335; *Fischerella* 7521, *F.* 7521; *Synechocystis* sp. PCC 6803, *S.* 6803.

**
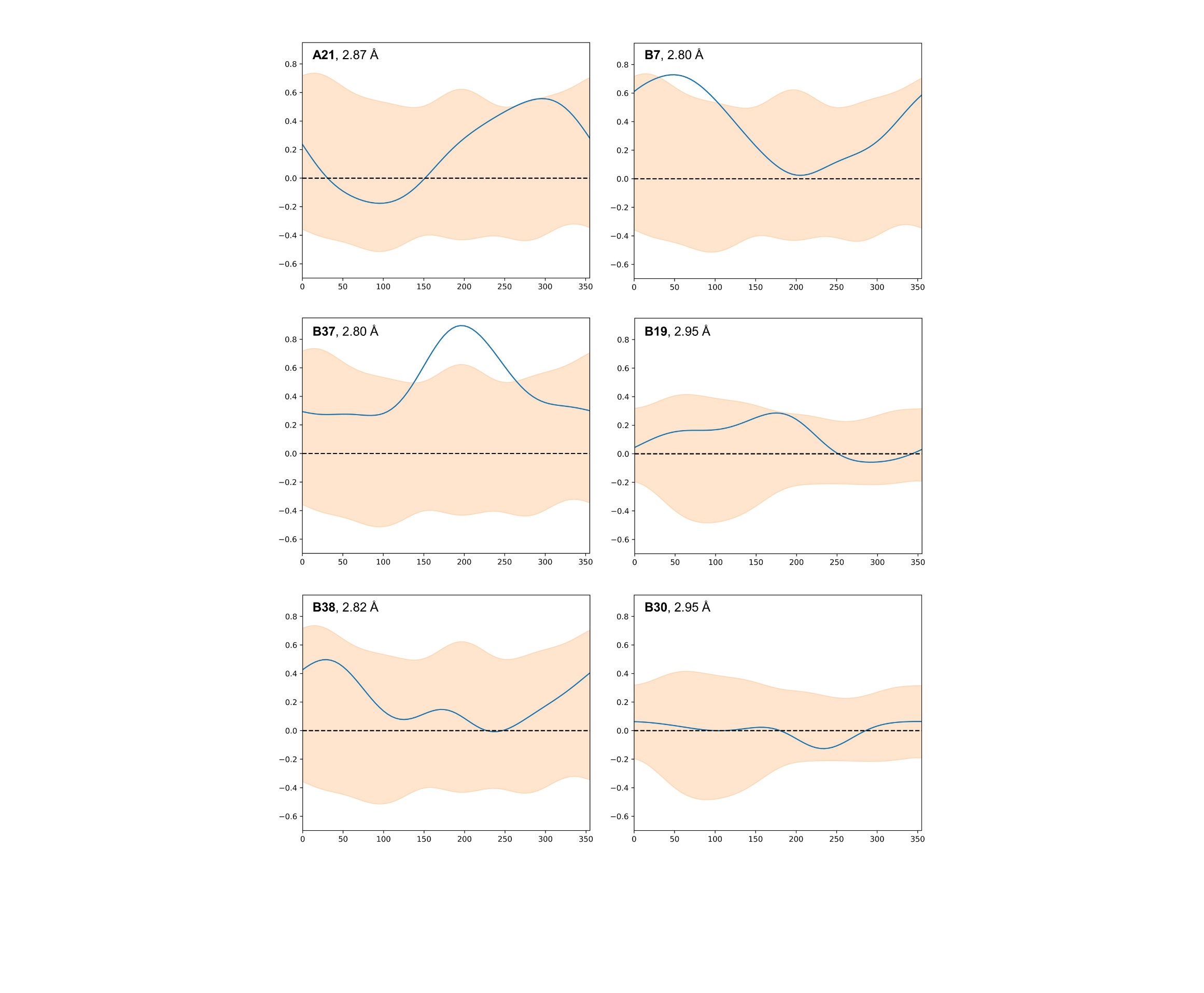
**

**Fig. S12. Cone scans for Chls at sites A21, B19, B7, B30, B37, and B38.** Each panel shows the C2 cone scan (blue line) overlaid with the null distribution derived from C7 methyl cone scans (orange area, see **Methods**). The Y-axis is in units of ESP and the X-axis is in units of degrees. The Chl site name and the local resolution for the central Mg of each Chl are indicated at the top left of each panel.


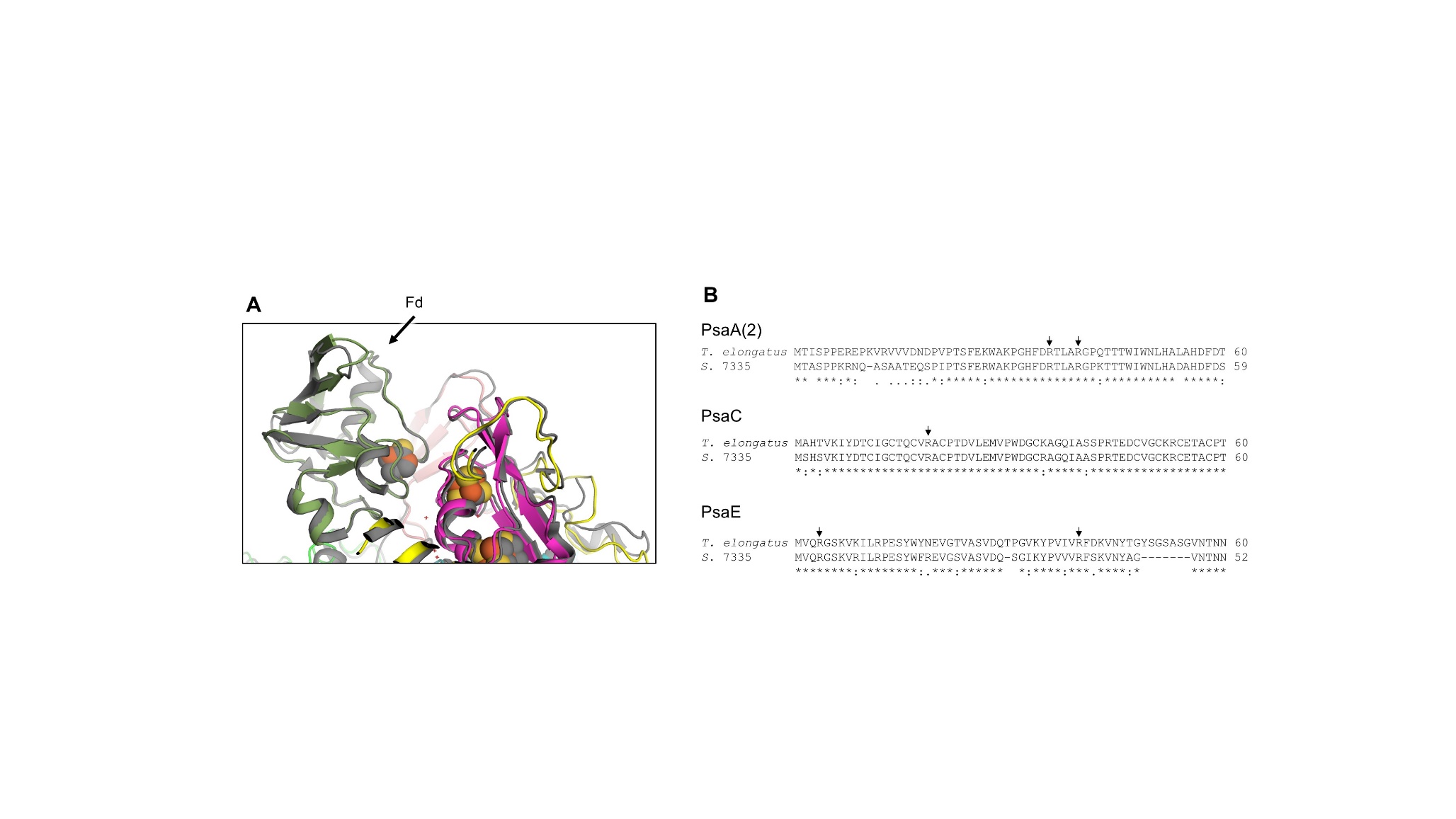


**Fig. S13. Conservation of Fd binding between *Synechococcus* 7335 and *T. elongatus* PSI.** **A** The superposition of *Synechococcus* 7335 FRL-PSI with Fd bound (colored) and *T. elongatus* PSI with Fd bound (grey). Only atoms in Fd were used in the superposition. Fe-S clusters are shown as spheres. **B** Partial sequence alignments showing core residues involved in Fd binding (black arrows) from the publication presenting the *T. elongatus* PSI-Fd X-ray crystal structure (PDB 5XF0). All of these Arg residues are fully conserved between the sequences.


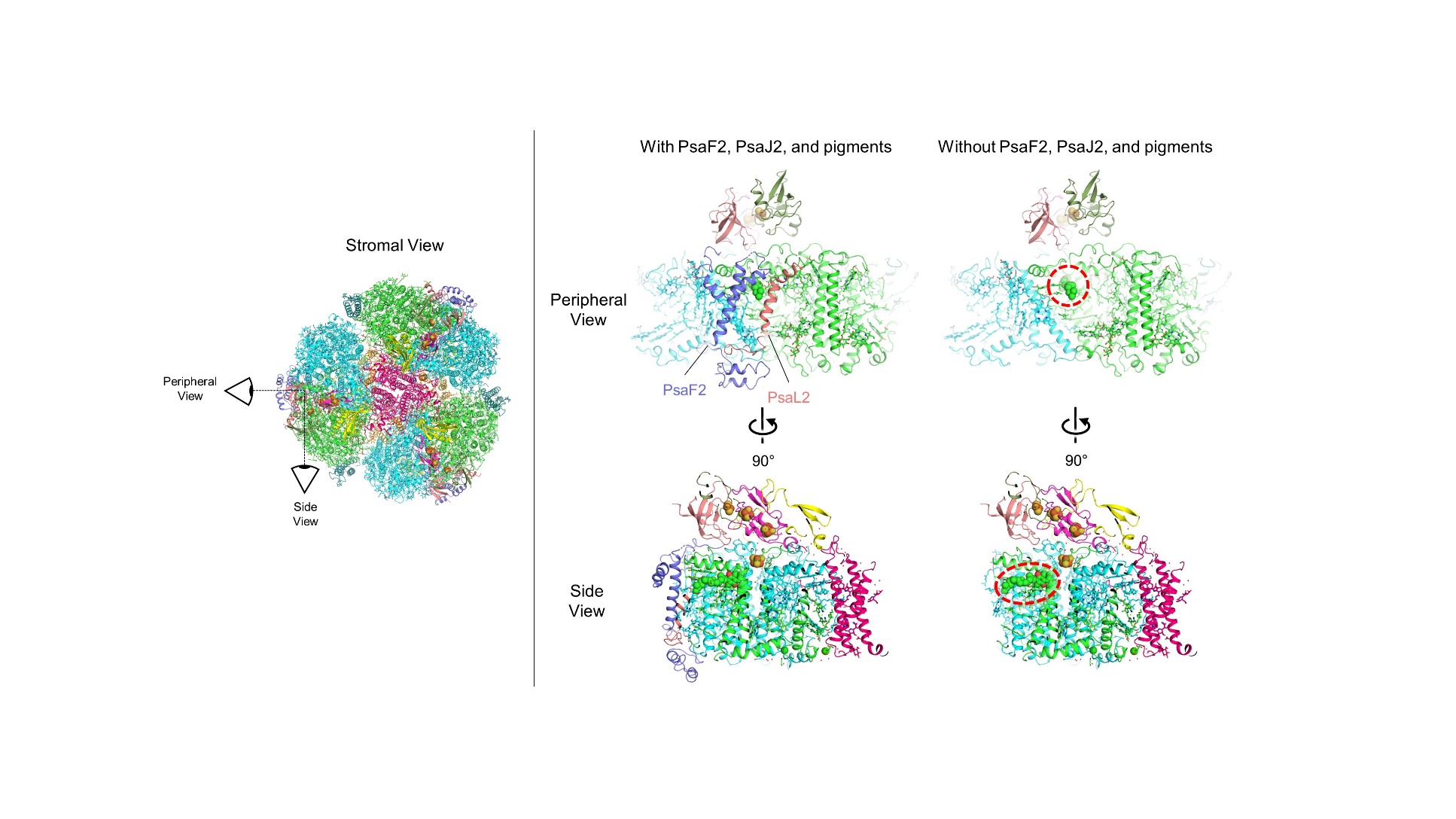


**Fig. S14. Proximity of the A_1A_ phylloquinone to the low occupancy structural elements.** In the left panel, a stromal view of the trimeric *Synechococcus* 7335 FRL-PSI structure is shown. The two eye and line symbols correspond to the two views shown in the right panel. In the right panel, only a monomer of the trimer is shown either with PsaF, PsaJ, Chls A1, A2, A39, and A30, and β-carotene 14 (left column), or without those structural elements (right column). The A_1A_ phylloquinone is shown in sphere representation. In the view without the low occupancy structural elements (right column), it is additionally circled with a red dashed line.

**
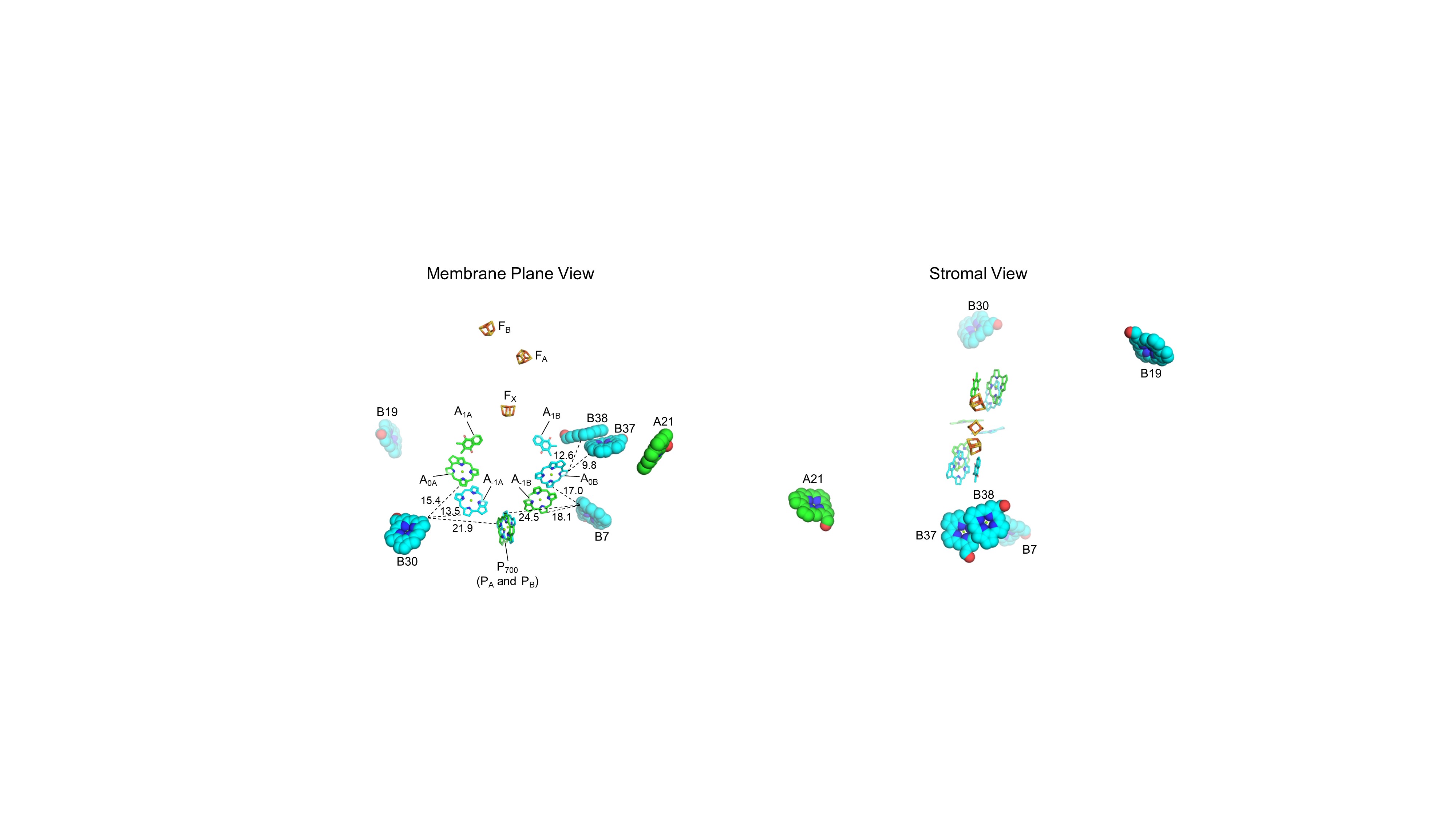
**

**Fig. S15. Chl *f* positions relative to the electron transfer chain cofactors.** The two panels show the electron transfer chain cofactors in stick representation and the Chl *f* molecules in sphere representation. The left panel shows a side view parallel to the membrane plane, and the right panel shows a stromal view perpendicular to the membrane plane. In addition to the Chl *f* sites, the electron transfer chain cofactors, and notable edge-to-edge distances (in units of Å) of Chl *f* molecules to the electron transfer chain, are additionally labeled in the membrane plane view (left). Note that the Chl *f* molecules at sites B7 and B30 are related by pseudo-C2 symmetry, and the Chl *f* molecules at sites A21 and B19 are related by pseudo-C2 symmetry. Tetrapyrrole ring substituents (except C2 formyl moieties of Chl *f* molecules) and isoprenoid tails are hidden for clarity.

**
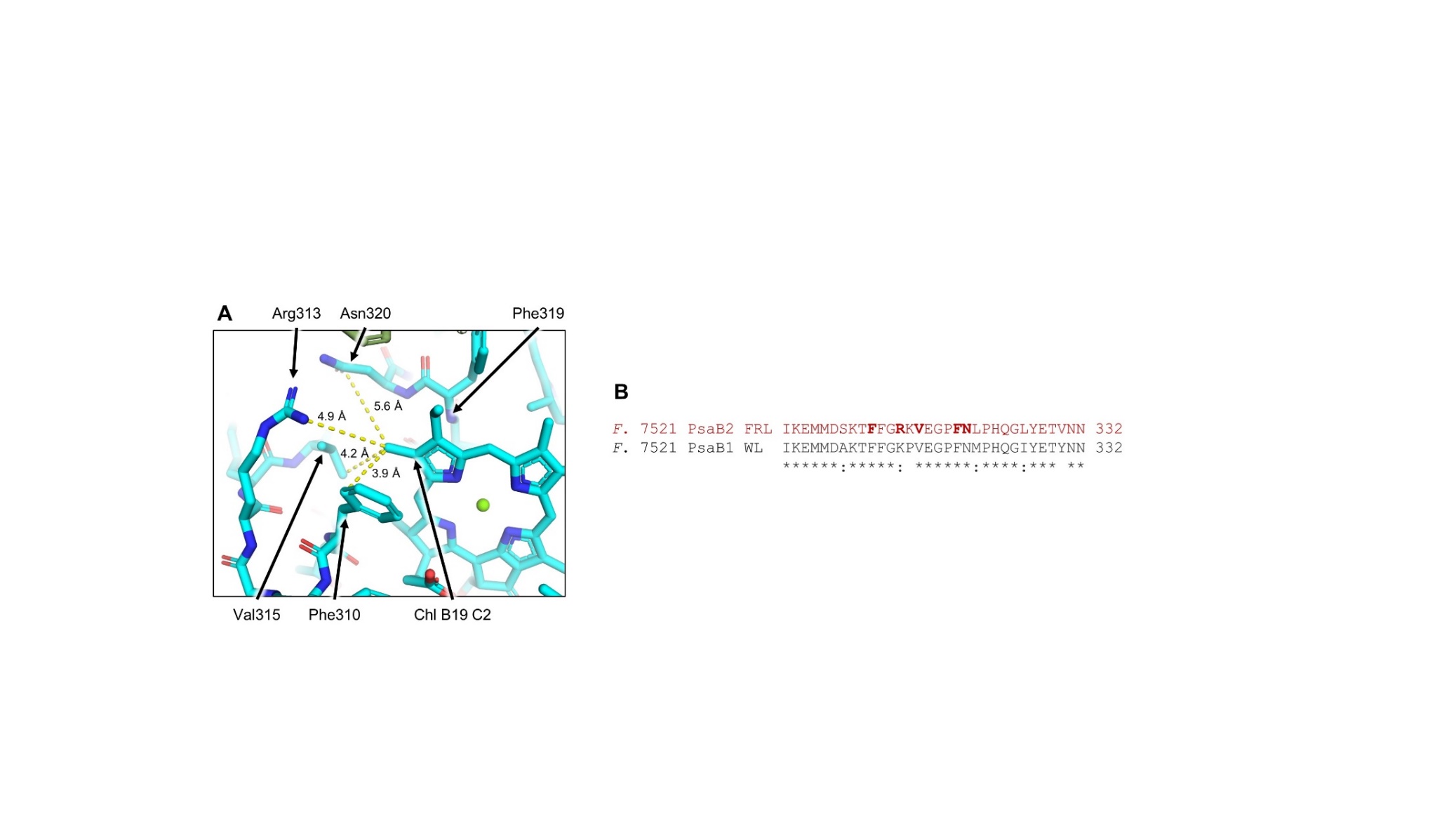
**

**Fig. S16. Structural features nearby the C2 moiety of Chl B19 in FRL-PSI from *Fischerella* 7521. A** Region of Chl B19 in *Fischerella* 7521 showing measurements to various structural features from the C2^1^ atom, suggesting it could accommodate Chl *f* with low specificity. All labels correspond to residues within PsaB2. **B** Partial sequence alignment of FRL (red font) and WL (black font) PsaB isoforms in *Fischerella* 7521. Bold residues correspond to those labeled in **A**. The Clustal Omega (39) similarity identifiers are shown below the sequence.

**Supporting Tables**

**Table S1. Cryo-EM data collection, refinement, and validation statistics for FRL-PSI from *Synechococcus* 7335.**

| **Data collection and processing** |  |  |
| --- | --- | --- |
| Magnification | x105,000 | |
| Voltage (kV) | 300 | |
| Electron exposure (e^-^Å^-2^) | 40.8 | |
| Defocus range (µm) | -1.5 to -2.5 | |
| Pixel size (Å) | 0.413 | |
| Symmetry imposed | C3 | |
| Initial particle images (no.) | 2,504,601 | |
| Final particle images (no.) | 286,672 | |
| Map resolution (Å) | 2.91 | |
| FSC threshold | 0.143 | |
| **Refinement** |  | |
| Initial model used (PDB code) | 6KMX, 7LX0, 5ZF0 | |
| Model resolution (Å) | 2.90 | |
| FSC threshold | 0.5 | |
| Map resolution range (Å) | 2.80-3.20 | |
| Map-sharpening *B* factor (Å^2^) | -104.2 | |
| Model composition |  | |
| Non-hydrogen atoms | 75,162 | |
| Protein residues | 7,074 | |
| Ligands | 417 | |
| *B* factors (Å^2^) |  | |
| Protein | 39 | |
| Ligands | 32 | |
| R.m.s. deviations |  | |
| Bond lengths (Å) | 0.010 | |
| Bond angles (°) | 2.134 | |
| **Validation** |  | |
| MolProbity | 2.45 | |
| Clashscore | 15.10 | |
| Rotamer outliers (%) | 2.64 | |
| Ramachandran plot |  | |
| Favored (%) | 93.06 | |
| Allowed (%) | 6.04 | |
| Disallowed (%) | 0.90 | |

**Table S2.** **Correlation between PsaB loop D sequence and the PsaX subunit.**

| **Organism** | **PsaX found?** | **Partial sequence alignment** |
| --- | --- | --- |
| *Nostocaceae* | Yes | NAKT--FFGKPV**EG**PFNMPHQ |
| *Cylindrospermum* sp. FACHB-282 | Yes | NSKSGLVPGSKS**EG**QFNLPHQ |
| *Phormidium* sp. LEGE 05292 | Yes | NAKD--FFGTKV**EG**PFNLPHQ |
| *Planktothrix* sp. UBA8407 | Yes | NSKDP-LFGIKN**EG**PFNLPHQ |
| *Sphaerospermopsis* | Yes | NAKT--FFGKSV**EG**PFNLPHQ |
| *Leptolyngbya* sp. O-77 | Yes | NSKAGLLS-KSS**EG**QFNLPHQ |

**Table S3. Comparison of stromal ridge subunits and Fd between *T. elongatus* PSI and *Synechococcus* 7335 FRL-PSI.**

|  | **Fd** | **PsaC** | **PsaD** | **PsaE** |
| --- | --- | --- | --- | --- |
| **Sequence identity (%)** | 77.55 | 93.83 | 65.22 | 72.06 |
| **RMSD (Å)** | 0.671 | 0.335 | 0.634 | 0.411 |

**Table S4. Edge-to-edge distances of Fe-S clusters in PSI structures with Fd bound.** Edge-to-edge distances include the S atoms from coordinating Cys sidechains. Distances are in units of Å.

|  | **F_X_ to F_A_** | **F_A_ to F_B_** | **F_B_ to [2Fe-2S]** |
| --- | --- | --- | --- |
| ***Synechococcus* 7335 with trimeric symmetry**  **(cryo-EM, PDB 7S3D)** | 10.0 | 6.7 | 5.7 |
| ***T. elongatus* monomer 1 (X-ray, PDB 5ZF0)** | 10.8 | 6.4 | 6.7 |
| ***T. elongatus* monomer 2 (X-ray, PDB 5ZF0)** | 10.5 | 6.9 | 5.6 |
| ***T. elongatus* monomer 3 (X-ray, PDB 5ZF0)** | 10.7 | 7.2 | 5.8 |
| ***Pisum sativum* (cryo-EM, PDB 6YAC)** | 10.7 | 6.5 | 5.6 |

**Supplementary Data**

**Supplementary Data 1. Jupyter Notebook for cone scans (external file).** A Jupyter Notebook presenting the raw data and cone scans for every Chl in the FRL-PSI structure from *Synechococcus* 7335 is provided.
