# Supplementary material for: Structure of a photosystem I-ferredoxin complex from a marine cyanobacterium provides insights into far-red light photoacclimation
Source: J Biol Chem. 2021 Nov 15;298(1):101408. doi: 10.1016/j.jbc.2021.101408 (PMC8689207; doi:10.1016/j.jbc.2021.101408)
Supplement: Data S1 [file mmc2.zip › cone_scan_data.html]

Index (1)


# Cone Scan data for FRL-PSI from *Synechococcus* sp. PCC 7335¶

This report contains the raw data for the cone scans produced for FRL-PSI from *Synechococcus* sp. PCC 7335, as referred to throughout the manuscript.

In [1]:

```
import numpy as np
import pandas as pd
import matplotlib.pyplot as plt
import matplotlib.patches as mpatches
import matplotlib.lines as mlines
```

#### Data availability¶

The raw data is contained in the following dictionary object. This is used to create a pandas DataFrame with the following columns:

- chl\_id: The chlorophyll binding sites of FRL-PSI from *Synechococcus* sp. PCC 7335
- loc\_res\_Mg: The local resolution of the ESP map at the central Mg of the chlorophyll
- bin: The bin index for each chlorophyll using a resolution cut-off of 2.91 Å
- scan\_angles: The different angles as which the ESP is sampled
- scan\_amp\_C2: The ESP sampled on the C2 substituent
- scan\_amp\_C7: The ESP sampled on the C7 substituent
- ci: The upper and lower bounds of the null distrubution

In [2]:

```
data = {"chl_id":{"A1011":"A11","A1012":"A12","A1013":"A13","A1101":"A01","A1102":"A02","A1103":"A03","A1104":"A04","A1105":"A05","A1106":"A06","A1107":"A07","A1108":"A08","A1109":"A09","A1110":"A10","A1111":"A11","A1112":"A12","A1113":"A13","A1114":"A14","A1115":"A15","A1116":"A16","A1117":"A17","A1118":"A18","A1119":"A19","A1120":"A20","A1121":"A21","A1122":"A22","A1123":"A23","A1124":"A24","A1125":"A25","A1126":"A26","A1127":"A27","A1128":"A28","A1129":"A29","A1130":"A30","A1131":"A31","A1132":"A32","A1133":"A33","A1134":"A34","A1135":"A35","A1136":"A36","A1137":"A37","A1138":"A38","A1139":"A39","A1140":"A40","A1141":"A41","B1021":"B21","B1022":"B22","B1023":"B23","B1201":"B01","B1202":"B02","B1203":"B03","B1204":"B04","B1205":"B05","B1206":"B06","B1207":"B07","B1208":"B08","B1209":"B09","B1210":"B10","B1211":"B11","B1212":"B12","B1213":"B13","B1214":"B14","B1215":"B15","B1216":"B16","B1217":"B17","B1218":"B18","B1219":"B19","B1220":"B20","B1221":"B21","B1222":"B22","B1223":"B23","B1224":"B24","B1225":"B25","B1226":"B26","B1227":"B27","B1228":"B28","B1229":"B29","B1230":"B30","B1231":"B31","B1232":"B32","B1233":"B33","B1234":"B34","B1235":"B35","B1236":"B36","B1237":"B37","B1238":"B38","B1239":"B39","B1240":"B40","K1401":"K01","L1501":"L01","L1502":"L02","L1503":"L03","M1501":"M01"},"loc_res_Mg":{"A1011":2.83069,"A1012":2.81667,"A1013":2.84799,"A1101":2.94216,"A1102":2.91775,"A1103":2.88435,"A1104":2.87858,"A1105":2.93427,"A1106":2.8951,"A1107":2.93245,"A1108":2.98728,"A1109":3.00415,"A1110":2.97582,"A1111":2.91355,"A1112":2.97204,"A1113":3.00048,"A1114":3.05558,"A1115":2.94058,"A1116":2.90095,"A1117":2.89613,"A1118":2.97091,"A1119":2.89017,"A1120":2.92757,"A1121":2.88394,"A1122":2.86221,"A1123":2.8808,"A1124":2.83864,"A1125":2.86306,"A1126":2.86305,"A1127":2.87209,"A1128":2.85976,"A1129":2.84612,"A1130":2.82946,"A1131":2.81696,"A1132":2.81978,"A1133":2.86908,"A1134":2.89288,"A1135":2.84585,"A1136":2.83072,"A1137":2.8321,"A1138":2.93882,"A1139":2.95402,"A1140":2.86794,"A1141":2.92708,"B1021":2.82589,"B1022":2.84025,"B1023":2.81452,"B1201":2.83542,"B1202":2.8556,"B1203":2.8398,"B1204":2.83331,"B1205":2.83266,"B1206":2.82791,"B1207":2.82068,"B1208":2.88112,"B1209":2.92325,"B1210":2.88329,"B1211":2.88572,"B1212":2.93722,"B1213":2.95531,"B1214":2.91701,"B1215":2.88197,"B1216":2.92693,"B1217":2.94904,"B1218":2.99252,"B1219":2.97563,"B1220":2.93319,"B1221":2.89957,"B1222":2.86402,"B1223":2.89915,"B1224":2.83489,"B1225":2.84886,"B1226":2.84517,"B1227":2.94558,"B1228":2.96414,"B1229":2.9565,"B1230":2.95061,"B1231":2.94944,"B1232":2.9815,"B1233":3.06274,"B1234":2.88137,"B1235":2.91634,"B1236":2.8978,"B1237":2.81778,"B1238":2.82126,"B1239":2.80997,"B1240":3.00882,"K1401":3.0432,"L1501":2.82427,"L1502":2.8198,"L1503":2.82468,"M1501":2.84425},"bin":{"A1011":0,"A1012":0,"A1013":0,"A1101":1,"A1102":1,"A1103":0,"A1104":0,"A1105":1,"A1106":0,"A1107":1,"A1108":1,"A1109":1,"A1110":1,"A1111":1,"A1112":1,"A1113":1,"A1114":1,"A1115":1,"A1116":0,"A1117":0,"A1118":1,"A1119":0,"A1120":1,"A1121":0,"A1122":0,"A1123":0,"A1124":0,"A1125":0,"A1126":0,"A1127":0,"A1128":0,"A1129":0,"A1130":0,"A1131":0,"A1132":0,"A1133":0,"A1134":0,"A1135":0,"A1136":0,"A1137":0,"A1138":1,"A1139":1,"A1140":0,"A1141":1,"B1021":0,"B1022":0,"B1023":0,"B1201":0,"B1202":0,"B1203":0,"B1204":0,"B1205":0,"B1206":0,"B1207":0,"B1208":0,"B1209":1,"B1210":0,"B1211":0,"B1212":1,"B1213":1,"B1214":1,"B1215":0,"B1216":1,"B1217":1,"B1218":1,"B1219":1,"B1220":1,"B1221":0,"B1222":0,"B1223":0,"B1224":0,"B1225":0,"B1226":0,"B1227":1,"B1228":1,"B1229":1,"B1230":1,"B1231":1,"B1232":1,"B1233":1,"B1234":0,"B1235":1,"B1236":0,"B1237":0,"B1238":0,"B1239":0,"B1240":1,"K1401":1,"L1501":0,"L1502":0,"L1503":0,"M1501":0},"scan_angles":{"A1011":[0.0,5.0,10.0,15.0,20.0,25.0,30.0,35.0,40.0,45.0,50.0,55.0,60.0,65.0,70.0,75.0,80.0,85.0,90.0,95.0,100.0,105.0,110.0,115.0,120.0,125.0,130.0,135.0,140.0,145.0,150.0,155.0,160.0,165.0,170.0,175.0,180.0,185.0,190.0,195.0,200.0,205.0,210.0,215.0,220.0,225.0,230.0,235.0,240.0,245.0,250.0,255.0,260.0,265.0,270.0,275.0,280.0,285.0,290.0,295.0,300.0,305.0,310.0,315.0,320.0,325.0,330.0,335.0,340.0,345.0,350.0,355.0],"A1012":[0.0,5.0,10.0,15.0,20.0,25.0,30.0,35.0,40.0,45.0,50.0,55.0,60.0,65.0,70.0,75.0,80.0,85.0,90.0,95.0,100.0,105.0,110.0,115.0,120.0,125.0,130.0,135.0,140.0,145.0,150.0,155.0,160.0,165.0,170.0,175.0,180.0,185.0,190.0,195.0,200.0,205.0,210.0,215.0,220.0,225.0,230.0,235.0,240.0,245.0,250.0,255.0,260.0,265.0,270.0,275.0,280.0,285.0,290.0,295.0,300.0,305.0,310.0,315.0,320.0,325.0,330.0,335.0,340.0,345.0,350.0,355.0],"A1013":[0.0,5.0,10.0,15.0,20.0,25.0,30.0,35.0,40.0,45.0,50.0,55.0,60.0,65.0,70.0,75.0,80.0,85.0,90.0,95.0,100.0,105.0,110.0,115.0,120.0,125.0,130.0,135.0,140.0,145.0,150.0,155.0,160.0,165.0,170.0,175.0,180.0,185.0,190.0,195.0,200.0,205.0,210.0,215.0,220.0,225.0,230.0,235.0,240.0,245.0,250.0,255.0,260.0,265.0,270.0,275.0,280.0,285.0,290.0,295.0,300.0,305.0,310.0,315.0,320.0,325.0,330.0,335.0,340.0,345.0,350.0,355.0],"A1101":[0.0,5.0,10.0,15.0,20.0,25.0,30.0,35.0,40.0,45.0,50.0,55.0,60.0,65.0,70.0,75.0,80.0,85.0,90.0,95.0,100.0,105.0,110.0,115.0,120.0,125.0,130.0,135.0,140.0,145.0,150.0,155.0,160.0,165.0,170.0,175.0,180.0,185.0,190.0,195.0,200.0,205.0,210.0,215.0,220.0,225.0,230.0,235.0,240.0,245.0,250.0,255.0,260.0,265.0,270.0,275.0,280.0,285.0,290.0,295.0,300.0,305.0,310.0,315.0,320.0,325.0,330.0,335.0,340.0,345.0,350.0,355.0],"A1102":[0.0,5.0,10.0,15.0,20.0,25.0,30.0,35.0,40.0,45.0,50.0,55.0,60.0,65.0,70.0,75.0,80.0,85.0,90.0,95.0,100.0,105.0,110.0,115.0,120.0,125.0,130.0,135.0,140.0,145.0,150.0,155.0,160.0,165.0,170.0,175.0,180.0,185.0,190.0,195.0,200.0,205.0,210.0,215.0,220.0,225.0,230.0,235.0,240.0,245.0,250.0,255.0,260.0,265.0,270.0,275.0,280.0,285.0,290.0,295.0,300.0,305.0,310.0,315.0,320.0,325.0,330.0,335.0,340.0,345.0,350.0,355.0],"A1103":[0.0,5.0,10.0,15.0,20.0,25.0,30.0,35.0,40.0,45.0,50.0,55.0,60.0,65.0,70.0,75.0,80.0,85.0,90.0,95.0,100.0,105.0,110.0,115.0,120.0,125.0,130.0,135.0,140.0,145.0,150.0,155.0,160.0,165.0,170.0,175.0,180.0,185.0,190.0,195.0,200.0,205.0,210.0,215.0,220.0,225.0,230.0,235.0,240.0,245.0,250.0,255.0,260.0,265.0,270.0,275.0,280.0,285.0,290.0,295.0,300.0,305.0,310.0,315.0,320.0,325.0,330.0,335.0,340.0,345.0,350.0,355.0],"A1104":[0.0,5.0,10.0,15.0,20.0,25.0,30.0,35.0,40.0,45.0,50.0,55.0,60.0,65.0,70.0,75.0,80.0,85.0,90.0,95.0,100.0,105.0,110.0,115.0,120.0,125.0,130.0,135.0,140.0,145.0,150.0,155.0,160.0,165.0,170.0,175.0,180.0,185.0,190.0,195.0,200.0,205.0,210.0,215.0,220.0,225.0,230.0,235.0,240.0,245.0,250.0,255.0,260.0,265.0,270.0,275.0,280.0,285.0,290.0,295.0,300.0,305.0,310.0,315.0,320.0,325.0,330.0,335.0,340.0,345.0,350.0,355.0],"A1105":[0.0,5.0,10.0,15.0,20.0,25.0,30.0,35.0,40.0,45.0,50.0,55.0,60.0,65.0,70.0,75.0,80.0,85.0,90.0,95.0,100.0,105.0,110.0,115.0,120.0,125.0,130.0,135.0,140.0,145.0,150.0,155.0,160.0,165.0,170.0,175.0,180.0,185.0,190.0,195.0,200.0,205.0,210.0,215.0,220.0,225.0,230.0,235.0,240.0,245.0,250.0,255.0,260.0,265.0,270.0,275.0,280.0,285.0,290.0,295.0,300.0,305.0,310.0,315.0,320.0,325.0,330.0,335.0,340.0,345.0,350.0,355.0],"A1106":[0.0,5.0,10.0,15.0,20.0,25.0,30.0,35.0,40.0,45.0,50.0,55.0,60.0,65.0,70.0,75.0,80.0,85.0,90.0,95.0,100.0,105.0,110.0,115.0,120.0,125.0,130.0,135.0,140.0,145.0,150.0,155.0,160.0,165.0,170.0,175.0,180.0,185.0,190.0,195.0,200.0,205.0,210.0,215.0,220.0,225.0,230.0,235.0,240.0,245.0,250.0,255.0,260.0,265.0,270.0,275.0,280.0,285.0,290.0,295.0,300.0,305.0,310.0,315.0,320.0,325.0,330.0,335.0,340.0,345.0,350.0,355.0],"A1107":[0.0,5.0,10.0,15.0,20.0,25.0,30.0,35.0,40.0,45.0,50.0,55.0,60.0,65.0,70.0,75.0,80.0,85.0,90.0,95.0,100.0,105.0,110.0,115.0,120.0,125.0,130.0,135.0,140.0,145.0,150.0,155.0,160.0,165.0,170.0,175.0,180.0,185.0,190.0,195.0,200.0,205.0,210.0,215.0,220.0,225.0,230.0,235.0,240.0,245.0,250.0,255.0,260.0,265.0,270.0,275.0,280.0,285.0,290.0,295.0,300.0,305.0,310.0,315.0,320.0,325.0,330.0,335.0,340.0,345.0,350.0,355.0],"A1108":[0.0,5.0,10.0,15.0,20.0,25.0,30.0,35.0,40.0,45.0,50.0,55.0,60.0,65.0,70.0,75.0,80.0,85.0,90.0,95.0,100.0,105.0,110.0,115.0,120.0,125.0,130.0,135.0,140.0,145.0,150.0,155.0,160.0,165.0,170.0,175.0,180.0,185.0,190.0,195.0,200.0,205.0,210.0,215.0,220.0,225.0,230.0,235.0,240.0,245.0,250.0,255.0,260.0,265.0,270.0,275.0,280.0,285.0,290.0,295.0,300.0,305.0,310.0,315.0,320.0,325.0,330.0,335.0,340.0,345.0,350.0,355.0],"A1109":[0.0,5.0,10.0,15.0,20.0,25.0,30.0,35.0,40.0,45.0,50.0,55.0,60.0,65.0,70.0,75.0,80.0,85.0,90.0,95.0,100.0,105.0,110.0,115.0,120.0,125.0,130.0,135.0,140.0,145.0,150.0,155.0,160.0,165.0,170.0,175.0,180.0,185.0,190.0,195.0,200.0,205.0,210.0,215.0,220.0,225.0,230.0,235.0,240.0,245.0,250.0,255.0,260.0,265.0,270.0,275.0,280.0,285.0,290.0,295.0,300.0,305.0,310.0,315.0,320.0,325.0,330.0,335.0,340.0,345.0,350.0,355.0],"A1110":[0.0,5.0,10.0,15.0,20.0,25.0,30.0,35.0,40.0,45.0,50.0,55.0,60.0,65.0,70.0,75.0,80.0,85.0,90.0,95.0,100.0,105.0,110.0,115.0,120.0,125.0,130.0,135.0,140.0,145.0,150.0,155.0,160.0,165.0,170.0,175.0,180.0,185.0,190.0,195.0,200.0,205.0,210.0,215.0,220.0,225.0,230.0,235.0,240.0,245.0,250.0,255.0,260.0,265.0,270.0,275.0,280.0,285.0,290.0,295.0,300.0,305.0,310.0,315.0,320.0,325.0,330.0,335.0,340.0,345.0,350.0,355.0],"A1111":[0.0,5.0,10.0,15.0,20.0,25.0,30.0,35.0,40.0,45.0,50.0,55.0,60.0,65.0,70.0,75.0,80.0,85.0,90.0,95.0,100.0,105.0,110.0,115.0,120.0,125.0,130.0,135.0,140.0,145.0,150.0,155.0,160.0,165.0,170.0,175.0,180.0,185.0,190.0,195.0,200.0,205.0,210.0,215.0,220.0,225.0,230.0,235.0,240.0,245.0,250.0,255.0,260.0,265.0,270.0,275.0,280.0,285.0,290.0,295.0,300.0,305.0,310.0,315.0,320.0,325.0,330.0,335.0,340.0,345.0,350.0,355.0],"A1112":[0.0,5.0,10.0,15.0,20.0,25.0,30.0,35.0,40.0,45.0,50.0,55.0,60.0,65.0,70.0,75.0,80.0,85.0,90.0,95.0,100.0,105.0,110.0,115.0,120.0,125.0,130.0,135.0,140.0,145.0,150.0,155.0,160.0,165.0,170.0,175.0,180.0,185.0,190.0,195.0,200.0,205.0,210.0,215.0,220.0,225.0,230.0,235.0,240.0,245.0,250.0,255.0,260.0,265.0,270.0,275.0,280.0,285.0,290.0,295.0,300.0,305.0,310.0,315.0,320.0,325.0,330.0,335.0,340.0,345.0,350.0,355.0],"A1113":[0.0,5.0,10.0,15.0,20.0,25.0,30.0,35.0,40.0,45.0,50.0,55.0,60.0,65.0,70.0,75.0,80.0,85.0,90.0,95.0,100.0,105.0,110.0,115.0,120.0,125.0,130.0,135.0,140.0,145.0,150.0,155.0,160.0,165.0,170.0,175.0,180.0,185.0,190.0,195.0,200.0,205.0,210.0,215.0,220.0,225.0,230.0,235.0,240.0,245.0,250.0,255.0,260.0,265.0,270.0,275.0,280.0,285.0,290.0,295.0,300.0,305.0,310.0,315.0,320.0,325.0,330.0,335.0,340.0,345.0,350.0,355.0],"A1114":[0.0,5.0,10.0,15.0,20.0,25.0,30.0,35.0,40.0,45.0,50.0,55.0,60.0,65.0,70.0,75.0,80.0,85.0,90.0,95.0,100.0,105.0,110.0,115.0,120.0,125.0,130.0,135.0,140.0,145.0,150.0,155.0,160.0,165.0,170.0,175.0,180.0,185.0,190.0,195.0,200.0,205.0,210.0,215.0,220.0,225.0,230.0,235.0,240.0,245.0,250.0,255.0,260.0,265.0,270.0,275.0,280.0,285.0,290.0,295.0,300.0,305.0,310.0,315.0,320.0,325.0,330.0,335.0,340.0,345.0,350.0,355.0],"A1115":[0.0,5.0,10.0,15.0,20.0,25.0,30.0,35.0,40.0,45.0,50.0,55.0,60.0,65.0,70.0,75.0,80.0,85.0,90.0,95.0,100.0,105.0,110.0,115.0,120.0,125.0,130.0,135.0,140.0,145.0,150.0,155.0,160.0,165.0,170.0,175.0,180.0,185.0,190.0,195.0,200.0,205.0,210.0,215.0,220.0,225.0,230.0,235.0,240.0,245.0,250.0,255.0,260.0,265.0,270.0,275.0,280.0,285.0,290.0,295.0,300.0,305.0,310.0,315.0,320.0,325.0,330.0,335.0,340.0,345.0,350.0,355.0],"A1116":[0.0,5.0,10.0,15.0,20.0,25.0,30.0,35.0,40.0,45.0,50.0,55.0,60.0,65.0,70.0,75.0,80.0,85.0,90.0,95.0,100.0,105.0,110.0,115.0,120.0,125.0,130.0,135.0,140.0,145.0,150.0,155.0,160.0,165.0,170.0,175.0,180.0,185.0,190.0,195.0,200.0,205.0,210.0,215.0,220.0,225.0,230.0,235.0,240.0,245.0,250.0,255.0,260.0,265.0,270.0,275.0,280.0,285.0,290.0,295.0,300.0,305.0,310.0,315.0,320.0,325.0,330.0,335.0,340.0,345.0,350.0,355.0],"A1117":[0.0,5.0,10.0,15.0,20.0,25.0,30.0,35.0,40.0,45.0,50.0,55.0,60.0,65.0,70.0,75.0,80.0,85.0,90.0,95.0,100.0,105.0,110.0,115.0,120.0,125.0,130.0,135.0,140.0,145.0,150.0,155.0,160.0,165.0,170.0,175.0,180.0,185.0,190.0,195.0,200.0,205.0,210.0,215.0,220.0,225.0,230.0,235.0,240.0,245.0,250.0,255.0,260.0,265.0,270.0,275.0,280.0,285.0,290.0,295.0,300.0,305.0,310.0,315.0,320.0,325.0,330.0,335.0,340.0,345.0,350.0,355.0],"A1118":[0.0,5.0,10.0,15.0,20.0,25.0,30.0,35.0,40.0,45.0,50.0,55.0,60.0,65.0,70.0,75.0,80.0,85.0,90.0,95.0,100.0,105.0,110.0,115.0,120.0,125.0,130.0,135.0,140.0,145.0,150.0,155.0,160.0,165.0,170.0,175.0,180.0,185.0,190.0,195.0,200.0,205.0,210.0,215.0,220.0,225.0,230.0,235.0,240.0,245.0,250.0,255.0,260.0,265.0,270.0,275.0,280.0,285.0,290.0,295.0,300.0,305.0,310.0,315.0,320.0,325.0,330.0,335.0,340.0,345.0,350.0,355.0],"A1119":[0.0,5.0,10.0,15.0,20.0,25.0,30.0,35.0,40.0,45.0,50.0,55.0,60.0,65.0,70.0,75.0,80.0,85.0,90.0,95.0,100.0,105.0,110.0,115.0,120.0,125.0,130.0,135.0,140.0,145.0,150.0,155.0,160.0,165.0,170.0,175.0,180.0,185.0,190.0,195.0,200.0,205.0,210.0,215.0,220.0,225.0,230.0,235.0,240.0,245.0,250.0,255.0,260.0,265.0,270.0,275.0,280.0,285.0,290.0,295.0,300.0,305.0,310.0,315.0,320.0,325.0,330.0,335.0,340.0,345.0,350.0,355.0],"A1120":[0.0,5.0,10.0,15.0,20.0,25.0,30.0,35.0,40.0,45.0,50.0,55.0,60.0,65.0,70.0,75.0,80.0,85.0,90.0,95.0,100.0,105.0,110.0,115.0,120.0,125.0,130.0,135.0,140.0,145.0,150.0,155.0,160.0,165.0,170.0,175.0,180.0,185.0,190.0,195.0,200.0,205.0,210.0,215.0,220.0,225.0,230.0,235.0,240.0,245.0,250.0,255.0,260.0,265.0,270.0,275.0,280.0,285.0,290.0,295.0,300.0,305.0,310.0,315.0,320.0,325.0,330.0,335.0,340.0,345.0,350.0,355.0],"A1121":[0.0,5.0,10.0,15.0,20.0,25.0,30.0,35.0,40.0,45.0,50.0,55.0,60.0,65.0,70.0,75.0,80.0,85.0,90.0,95.0,100.0,105.0,110.0,115.0,120.0,125.0,130.0,135.0,140.0,145.0,150.0,155.0,160.0,165.0,170.0,175.0,180.0,185.0,190.0,195.0,200.0,205.0,210.0,215.0,220.0,225.0,230.0,235.0,240.0,245.0,250.0,255.0,260.0,265.0,270.0,275.0,280.0,285.0,290.0,295.0,300.0,305.0,310.0,315.0,320.0,325.0,330.0,335.0,340.0,345.0,350.0,355.0],"A1122":[0.0,5.0,10.0,15.0,20.0,25.0,30.0,35.0,40.0,45.0,50.0,55.0,60.0,65.0,70.0,75.0,80.0,85.0,90.0,95.0,100.0,105.0,110.0,115.0,120.0,125.0,130.0,135.0,140.0,145.0,150.0,155.0,160.0,165.0,170.0,175.0,180.0,185.0,190.0,195.0,200.0,205.0,210.0,215.0,220.0,225.0,230.0,235.0,240.0,245.0,250.0,255.0,260.0,265.0,270.0,275.0,280.0,285.0,290.0,295.0,300.0,305.0,310.0,315.0,320.0,325.0,330.0,335.0,340.0,345.0,350.0,355.0],"A1123":[0.0,5.0,10.0,15.0,20.0,25.0,30.0,35.0,40.0,45.0,50.0,55.0,60.0,65.0,70.0,75.0,80.0,85.0,90.0,95.0,100.0,105.0,110.0,115.0,120.0,125.0,130.0,135.0,140.0,145.0,150.0,155.0,160.0,165.0,170.0,175.0,180.0,185.0,190.0,195.0,200.0,205.0,210.0,215.0,220.0,225.0,230.0,235.0,240.0,245.0,250.0,255.0,260.0,265.0,270.0,275.0,280.0,285.0,290.0,295.0,300.0,305.0,310.0,315.0,320.0,325.0,330.0,335.0,340.0,345.0,350.0,355.0],"A1124":[0.0,5.0,10.0,15.0,20.0,25.0,30.0,35.0,40.0,45.0,50.0,55.0,60.0,65.0,70.0,75.0,80.0,85.0,90.0,95.0,100.0,105.0,110.0,115.0,120.0,125.0,130.0,135.0,140.0,145.0,150.0,155.0,160.0,165.0,170.0,175.0,180.0,185.0,190.0,195.0,200.0,205.0,210.0,215.0,220.0,225.0,230.0,235.0,240.0,245.0,250.0,255.0,260.0,265.0,270.0,275.0,280.0,285.0,290.0,295.0,300.0,305.0,310.0,315.0,320.0,325.0,330.0,335.0,340.0,345.0,350.0,355.0],"A1125":[0.0,5.0,10.0,15.0,20.0,25.0,30.0,35.0,40.0,45.0,50.0,55.0,60.0,65.0,70.0,75.0,80.0,85.0,90.0,95.0,100.0,105.0,110.0,115.0,120.0,125.0,130.0,135.0,140.0,145.0,150.0,155.0,160.0,165.0,170.0,175.0,180.0,185.0,190.0,195.0,200.0,205.0,210.0,215.0,220.0,225.0,230.0,235.0,240.0,245.0,250.0,255.0,260.0,265.0,270.0,275.0,280.0,285.0,290.0,295.0,300.0,305.0,310.0,315.0,320.0,325.0,330.0,335.0,340.0,345.0,350.0,355.0],"A1126":[0.0,5.0,10.0,15.0,20.0,25.0,30.0,35.0,40.0,45.0,50.0,55.0,60.0,65.0,70.0,75.0,80.0,85.0,90.0,95.0,100.0,105.0,110.0,115.0,120.0,125.0,130.0,135.0,140.0,145.0,150.0,155.0,160.0,165.0,170.0,175.0,180.0,185.0,190.0,195.0,200.0,205.0,210.0,215.0,220.0,225.0,230.0,235.0,240.0,245.0,250.0,255.0,260.0,265.0,270.0,275.0,280.0,285.0,290.0,295.0,300.0,305.0,310.0,315.0,320.0,325.0,330.0,335.0,340.0,345.0,350.0,355.0],"A1127":[0.0,5.0,10.0,15.0,20.0,25.0,30.0,35.0,40.0,45.0,50.0,55.0,60.0,65.0,70.0,75.0,80.0,85.0,90.0,95.0,100.0,105.0,110.0,115.0,120.0,125.0,130.0,135.0,140.0,145.0,150.0,155.0,160.0,165.0,170.0,175.0,180.0,185.0,190.0,195.0,200.0,205.0,210.0,215.0,220.0,225.0,230.0,235.0,240.0,245.0,250.0,255.0,260.0,265.0,270.0,275.0,280.0,285.0,290.0,295.0,300.0,305.0,310.0,315.0,320.0,325.0,330.0,335.0,340.0,345.0,350.0,355.0],"A1128":[0.0,5.0,10.0,15.0,20.0,25.0,30.0,35.0,40.0,45.0,50.0,55.0,60.0,65.0,70.0,75.0,80.0,85.0,90.0,95.0,100.0,105.0,110.0,115.0,120.0,125.0,130.0,135.0,140.0,145.0,150.0,155.0,160.0,165.0,170.0,175.0,180.0,185.0,190.0,195.0,200.0,205.0,210.0,215.0,220.0,225.0,230.0,235.0,240.0,245.0,250.0,255.0,260.0,265.0,270.0,275.0,280.0,285.0,290.0,295.0,300.0,305.0,310.0,315.0,320.0,325.0,330.0,335.0,340.0,345.0,350.0,355.0],"A1129":[0.0,5.0,10.0,15.0,20.0,25.0,30.0,35.0,40.0,45.0,50.0,55.0,60.0,65.0,70.0,75.0,80.0,85.0,90.0,95.0,100.0,105.0,110.0,115.0,120.0,125.0,130.0,135.0,140.0,145.0,150.0,155.0,160.0,165.0,170.0,175.0,180.0,185.0,190.0,195.0,200.0,205.0,210.0,215.0,220.0,225.0,230.0,235.0,240.0,245.0,250.0,255.0,260.0,265.0,270.0,275.0,280.0,285.0,290.0,295.0,300.0,305.0,310.0,315.0,320.0,325.0,330.0,335.0,340.0,345.0,350.0,355.0],"A1130":[0.0,5.0,10.0,15.0,20.0,25.0,30.0,35.0,40.0,45.0,50.0,55.0,60.0,65.0,70.0,75.0,80.0,85.0,90.0,95.0,100.0,105.0,110.0,115.0,120.0,125.0,130.0,135.0,140.0,145.0,150.0,155.0,160.0,165.0,170.0,175.0,180.0,185.0,190.0,195.0,200.0,205.0,210.0,215.0,220.0,225.0,230.0,235.0,240.0,245.0,250.0,255.0,260.0,265.0,270.0,275.0,280.0,285.0,290.0,295.0,300.0,305.0,310.0,315.0,320.0,325.0,330.0,335.0,340.0,345.0,350.0,355.0],"A1131":[0.0,5.0,10.0,15.0,20.0,25.0,30.0,35.0,40.0,45.0,50.0,55.0,60.0,65.0,70.0,75.0,80.0,85.0,90.0,95.0,100.0,105.0,110.0,115.0,120.0,125.0,130.0,135.0,140.0,145.0,150.0,155.0,160.0,165.0,170.0,175.0,180.0,185.0,190.0,195.0,200.0,205.0,210.0,215.0,220.0,225.0,230.0,235.0,240.0,245.0,250.0,255.0,260.0,265.0,270.0,275.0,280.0,285.0,290.0,295.0,300.0,305.0,310.0,315.0,320.0,325.0,330.0,335.0,340.0,345.0,350.0,355.0],"A1132":[0.0,5.0,10.0,15.0,20.0,25.0,30.0,35.0,40.0,45.0,50.0,55.0,60.0,65.0,70.0,75.0,80.0,85.0,90.0,95.0,100.0,105.0,110.0,115.0,120.0,125.0,130.0,135.0,140.0,145.0,150.0,155.0,160.0,165.0,170.0,175.0,180.0,185.0,190.0,195.0,200.0,205.0,210.0,215.0,220.0,225.0,230.0,235.0,240.0,245.0,250.0,255.0,260.0,265.0,270.0,275.0,280.0,285.0,290.0,295.0,300.0,305.0,310.0,315.0,320.0,325.0,330.0,335.0,340.0,345.0,350.0,355.0],"A1133":[0.0,5.0,10.0,15.0,20.0,25.0,30.0,35.0,40.0,45.0,50.0,55.0,60.0,65.0,70.0,75.0,80.0,85.0,90.0,95.0,100.0,105.0,110.0,115.0,120.0,125.0,130.0,135.0,140.0,145.0,150.0,155.0,160.0,165.0,170.0,175.0,180.0,185.0,190.0,195.0,200.0,205.0,210.0,215.0,220.0,225.0,230.0,235.0,240.0,245.0,250.0,255.0,260.0,265.0,270.0,275.0,280.0,285.0,290.0,295.0,300.0,305.0,310.0,315.0,320.0,325.0,330.0,335.0,340.0,345.0,350.0,355.0],"A1134":[0.0,5.0,10.0,15.0,20.0,25.0,30.0,35.0,40.0,45.0,50.0,55.0,60.0,65.0,70.0,75.0,80.0,85.0,90.0,95.0,100.0,105.0,110.0,115.0,120.0,125.0,130.0,135.0,140.0,145.0,150.0,155.0,160.0,165.0,170.0,175.0,180.0,185.0,190.0,195.0,200.0,205.0,210.0,215.0,220.0,225.0,230.0,235.0,240.0,245.0,250.0,255.0,260.0,265.0,270.0,275.0,280.0,285.0,290.0,295.0,300.0,305.0,310.0,315.0,320.0,325.0,330.0,335.0,340.0,345.0,350.0,355.0],"A1135":[0.0,5.0,10.0,15.0,20.0,25.0,30.0,35.0,40.0,45.0,50.0,55.0,60.0,65.0,70.0,75.0,80.0,85.0,90.0,95.0,100.0,105.0,110.0,115.0,120.0,125.0,130.0,135.0,140.0,145.0,150.0,155.0,160.0,165.0,170.0,175.0,180.0,185.0,190.0,195.0,200.0,205.0,210.0,215.0,220.0,225.0,230.0,235.0,240.0,245.0,250.0,255.0,260.0,265.0,270.0,275.0,280.0,285.0,290.0,295.0,300.0,305.0,310.0,315.0,320.0,325.0,330.0,335.0,340.0,345.0,350.0,355.0],"A1136":[0.0,5.0,10.0,15.0,20.0,25.0,30.0,35.0,40.0,45.0,50.0,55.0,60.0,65.0,70.0,75.0,80.0,85.0,90.0,95.0,100.0,105.0,110.0,115.0,120.0,125.0,130.0,135.0,140.0,145.0,150.0,155.0,160.0,165.0,170.0,175.0,180.0,185.0,190.0,195.0,200.0,205.0,210.0,215.0,220.0,225.0,230.0,235.0,240.0,245.0,250.0,255.0,260.0,265.0,270.0,275.0,280.0,285.0,290.0,295.0,300.0,305.0,310.0,315.0,320.0,325.0,330.0,335.0,340.0,345.0,350.0,355.0],"A1137":[0.0,5.0,10.0,15.0,20.0,25.0,30.0,35.0,40.0,45.0,50.0,55.0,60.0,65.0,70.0,75.0,80.0,85.0,90.0,95.0,100.0,105.0,110.0,115.0,120.0,125.0,130.0,135.0,140.0,145.0,150.0,155.0,160.0,165.0,170.0,175.0,180.0,185.0,190.0,195.0,200.0,205.0,210.0,215.0,220.0,225.0,230.0,235.0,240.0,245.0,250.0,255.0,260.0,265.0,270.0,275.0,280.0,285.0,290.0,295.0,300.0,305.0,310.0,315.0,320.0,325.0,330.0,335.0,340.0,345.0,350.0,355.0],"A1138":[0.0,5.0,10.0,15.0,20.0,25.0,30.0,35.0,40.0,45.0,50.0,55.0,60.0,65.0,70.0,75.0,80.0,85.0,90.0,95.0,100.0,105.0,110.0,115.0,120.0,125.0,130.0,135.0,140.0,145.0,150.0,155.0,160.0,165.0,170.0,175.0,180.0,185.0,190.0,195.0,200.0,205.0,210.0,215.0,220.0,225.0,230.0,235.0,240.0,245.0,250.0,255.0,260.0,265.0,270.0,275.0,280.0,285.0,290.0,295.0,300.0,305.0,310.0,315.0,320.0,325.0,330.0,335.0,340.0,345.0,350.0,355.0],"A1139":[0.0,5.0,10.0,15.0,20.0,25.0,30.0,35.0,40.0,45.0,50.0,55.0,60.0,65.0,70.0,75.0,80.0,85.0,90.0,95.0,100.0,105.0,110.0,115.0,120.0,125.0,130.0,135.0,140.0,145.0,150.0,155.0,160.0,165.0,170.0,175.0,180.0,185.0,190.0,195.0,200.0,205.0,210.0,215.0,220.0,225.0,230.0,235.0,240.0,245.0,250.0,255.0,260.0,265.0,270.0,275.0,280.0,285.0,290.0,295.0,300.0,305.0,310.0,315.0,320.0,325.0,330.0,335.0,340.0,345.0,350.0,355.0],"A1140":[0.0,5.0,10.0,15.0,20.0,25.0,30.0,35.0,40.0,45.0,50.0,55.0,60.0,65.0,70.0,75.0,80.0,85.0,90.0,95.0,100.0,105.0,110.0,115.0,120.0,125.0,130.0,135.0,140.0,145.0,150.0,155.0,160.0,165.0,170.0,175.0,180.0,185.0,190.0,195.0,200.0,205.0,210.0,215.0,220.0,225.0,230.0,235.0,240.0,245.0,250.0,255.0,260.0,265.0,270.0,275.0,280.0,285.0,290.0,295.0,300.0,305.0,310.0,315.0,320.0,325.0,330.0,335.0,340.0,345.0,350.0,355.0],"A1141":[0.0,5.0,10.0,15.0,20.0,25.0,30.0,35.0,40.0,45.0,50.0,55.0,60.0,65.0,70.0,75.0,80.0,85.0,90.0,95.0,100.0,105.0,110.0,115.0,120.0,125.0,130.0,135.0,140.0,145.0,150.0,155.0,160.0,165.0,170.0,175.0,180.0,185.0,190.0,195.0,200.0,205.0,210.0,215.0,220.0,225.0,230.0,235.0,240.0,245.0,250.0,255.0,260.0,265.0,270.0,275.0,280.0,285.0,290.0,295.0,300.0,305.0,310.0,315.0,320.0,325.0,330.0,335.0,340.0,345.0,350.0,355.0],"B1021":[0.0,5.0,10.0,15.0,20.0,25.0,30.0,35.0,40.0,45.0,50.0,55.0,60.0,65.0,70.0,75.0,80.0,85.0,90.0,95.0,100.0,105.0,110.0,115.0,120.0,125.0,130.0,135.0,140.0,145.0,150.0,155.0,160.0,165.0,170.0,175.0,180.0,185.0,190.0,195.0,200.0,205.0,210.0,215.0,220.0,225.0,230.0,235.0,240.0,245.0,250.0,255.0,260.0,265.0,270.0,275.0,280.0,285.0,290.0,295.0,300.0,305.0,310.0,315.0,320.0,325.0,330.0,335.0,340.0,345.0,350.0,355.0],"B1022":[0.0,5.0,10.0,15.0,20.0,25.0,30.0,35.0,40.0,45.0,50.0,55.0,60.0,65.0,70.0,75.0,80.0,85.0,90.0,95.0,100.0,105.0,110.0,115.0,120.0,125.0,130.0,135.0,140.0,145.0,150.0,155.0,160.0,165.0,170.0,175.0,180.0,185.0,190.0,195.0,200.0,205.0,210.0,215.0,220.0,225.0,230.0,235.0,240.0,245.0,250.0,255.0,260.0,265.0,270.0,275.0,280.0,285.0,290.0,295.0,300.0,305.0,310.0,315.0,320.0,325.0,330.0,335.0,340.0,345.0,350.0,355.0],"B1023":[0.0,5.0,10.0,15.0,20.0,25.0,30.0,35.0,40.0,45.0,50.0,55.0,60.0,65.0,70.0,75.0,80.0,85.0,90.0,95.0,100.0,105.0,110.0,115.0,120.0,125.0,130.0,135.0,140.0,145.0,150.0,155.0,160.0,165.0,170.0,175.0,180.0,185.0,190.0,195.0,200.0,205.0,210.0,215.0,220.0,225.0,230.0,235.0,240.0,245.0,250.0,255.0,260.0,265.0,270.0,275.0,280.0,285.0,290.0,295.0,300.0,305.0,310.0,315.0,320.0,325.0,330.0,335.0,340.0,345.0,350.0,355.0],"B1201":[0.0,5.0,10.0,15.0,20.0,25.0,30.0,35.0,40.0,45.0,50.0,55.0,60.0,65.0,70.0,75.0,80.0,85.0,90.0,95.0,100.0,105.0,110.0,115.0,120.0,125.0,130.0,135.0,140.0,145.0,150.0,155.0,160.0,165.0,170.0,175.0,180.0,185.0,190.0,195.0,200.0,205.0,210.0,215.0,220.0,225.0,230.0,235.0,240.0,245.0,250.0,255.0,260.0,265.0,270.0,275.0,280.0,285.0,290.0,295.0,300.0,305.0,310.0,315.0,320.0,325.0,330.0,335.0,340.0,345.0,350.0,355.0],"B1202":[0.0,5.0,10.0,15.0,20.0,25.0,30.0,35.0,40.0,45.0,50.0,55.0,60.0,65.0,70.0,75.0,80.0,85.0,90.0,95.0,100.0,105.0,110.0,115.0,120.0,125.0,130.0,135.0,140.0,145.0,150.0,155.0,160.0,165.0,170.0,175.0,180.0,185.0,190.0,195.0,200.0,205.0,210.0,215.0,220.0,225.0,230.0,235.0,240.0,245.0,250.0,255.0,260.0,265.0,270.0,275.0,280.0,285.0,290.0,295.0,300.0,305.0,310.0,315.0,320.0,325.0,330.0,335.0,340.0,345.0,350.0,355.0],"B1203":[0.0,5.0,10.0,15.0,20.0,25.0,30.0,35.0,40.0,45.0,50.0,55.0,60.0,65.0,70.0,75.0,80.0,85.0,90.0,95.0,100.0,105.0,110.0,115.0,120.0,125.0,130.0,135.0,140.0,145.0,150.0,155.0,160.0,165.0,170.0,175.0,180.0,185.0,190.0,195.0,200.0,205.0,210.0,215.0,220.0,225.0,230.0,235.0,240.0,245.0,250.0,255.0,260.0,265.0,270.0,275.0,280.0,285.0,290.0,295.0,300.0,305.0,310.0,315.0,320.0,325.0,330.0,335.0,340.0,345.0,350.0,355.0],"B1204":[0.0,5.0,10.0,15.0,20.0,25.0,30.0,35.0,40.0,45.0,50.0,55.0,60.0,65.0,70.0,75.0,80.0,85.0,90.0,95.0,100.0,105.0,110.0,115.0,120.0,125.0,130.0,135.0,140.0,145.0,150.0,155.0,160.0,165.0,170.0,175.0,180.0,185.0,190.0,195.0,200.0,205.0,210.0,215.0,220.0,225.0,230.0,235.0,240.0,245.0,250.0,255.0,260.0,265.0,270.0,275.0,280.0,285.0,290.0,295.0,300.0,305.0,310.0,315.0,320.0,325.0,330.0,335.0,340.0,345.0,350.0,355.0],"B1205":[0.0,5.0,10.0,15.0,20.0,25.0,30.0,35.0,40.0,45.0,50.0,55.0,60.0,65.0,70.0,75.0,80.0,85.0,90.0,95.0,100.0,105.0,110.0,115.0,120.0,125.0,130.0,135.0,140.0,145.0,150.0,155.0,160.0,165.0,170.0,175.0,180.0,185.0,190.0,195.0,200.0,205.0,210.0,215.0,220.0,225.0,230.0,235.0,240.0,245.0,250.0,255.0,260.0,265.0,270.0,275.0,280.0,285.0,290.0,295.0,300.0,305.0,310.0,315.0,320.0,325.0,330.0,335.0,340.0,345.0,350.0,355.0],"B1206":[0.0,5.0,10.0,15.0,20.0,25.0,30.0,35.0,40.0,45.0,50.0,55.0,60.0,65.0,70.0,75.0,80.0,85.0,90.0,95.0,100.0,105.0,110.0,115.0,120.0,125.0,130.0,135.0,140.0,145.0,150.0,155.0,160.0,165.0,170.0,175.0,180.0,185.0,190.0,195.0,200.0,205.0,210.0,215.0,220.0,225.0,230.0,235.0,240.0,245.0,250.0,255.0,260.0,265.0,270.0,275.0,280.0,285.0,290.0,295.0,300.0,305.0,310.0,315.0,320.0,325.0,330.0,335.0,340.0,345.0,350.0,355.0],"B1207":[0.0,5.0,10.0,15.0,20.0,25.0,30.0,35.0,40.0,45.0,50.0,55.0,60.0,65.0,70.0,75.0,80.0,85.0,90.0,95.0,100.0,105.0,110.0,115.0,120.0,125.0,130.0,135.0,140.0,145.0,150.0,155.0,160.0,165.0,170.0,175.0,180.0,185.0,190.0,195.0,200.0,205.0,210.0,215.0,220.0,225.0,230.0,235.0,240.0,245.0,250.0,255.0,260.0,265.0,270.0,275.0,280.0,285.0,290.0,295.0,300.0,305.0,310.0,315.0,320.0,325.0,330.0,335.0,340.0,345.0,350.0,355.0],"B1208":[0.0,5.0,10.0,15.0,20.0,25.0,30.0,35.0,40.0,45.0,50.0,55.0,60.0,65.0,70.0,75.0,80.0,85.0,90.0,95.0,100.0,105.0,110.0,115.0,120.0,125.0,130.0,135.0,140.0,145.0,150.0,155.0,160.0,165.0,170.0,175.0,180.0,185.0,190.0,195.0,200.0,205.0,210.0,215.0,220.0,225.0,230.0,235.0,240.0,245.0,250.0,255.0,260.0,265.0,270.0,275.0,280.0,285.0,290.0,295.0,300.0,305.0,310.0,315.0,320.0,325.0,330.0,335.0,340.0,345.0,350.0,355.0],"B1209":[0.0,5.0,10.0,15.0,20.0,25.0,30.0,35.0,40.0,45.0,50.0,55.0,60.0,65.0,70.0,75.0,80.0,85.0,90.0,95.0,100.0,105.0,110.0,115.0,120.0,125.0,130.0,135.0,140.0,145.0,150.0,155.0,160.0,165.0,170.0,175.0,180.0,185.0,190.0,195.0,200.0,205.0,210.0,215.0,220.0,225.0,230.0,235.0,240.0,245.0,250.0,255.0,260.0,265.0,270.0,275.0,280.0,285.0,290.0,295.0,300.0,305.0,310.0,315.0,320.0,325.0,330.0,335.0,340.0,345.0,350.0,355.0],"B1210":[0.0,5.0,10.0,15.0,20.0,25.0,30.0,35.0,40.0,45.0,50.0,55.0,60.0,65.0,70.0,75.0,80.0,85.0,90.0,95.0,100.0,105.0,110.0,115.0,120.0,125.0,130.0,135.0,140.0,145.0,150.0,155.0,160.0,165.0,170.0,175.0,180.0,185.0,190.0,195.0,200.0,205.0,210.0,215.0,220.0,225.0,230.0,235.0,240.0,245.0,250.0,255.0,260.0,265.0,270.0,275.0,280.0,285.0,290.0,295.0,300.0,305.0,310.0,315.0,320.0,325.0,330.0,335.0,340.0,345.0,350.0,355.0],"B1211":[0.0,5.0,10.0,15.0,20.0,25.0,30.0,35.0,40.0,45.0,50.0,55.0,60.0,65.0,70.0,75.0,80.0,85.0,90.0,95.0,100.0,105.0,110.0,115.0,120.0,125.0,130.0,135.0,140.0,145.0,150.0,155.0,160.0,165.0,170.0,175.0,180.0,185.0,190.0,195.0,200.0,205.0,210.0,215.0,220.0,225.0,230.0,235.0,240.0,245.0,250.0,255.0,260.0,265.0,270.0,275.0,280.0,285.0,290.0,295.0,300.0,305.0,310.0,315.0,320.0,325.0,330.0,335.0,340.0,345.0,350.0,355.0],"B1212":[0.0,5.0,10.0,15.0,20.0,25.0,30.0,35.0,40.0,45.0,50.0,55.0,60.0,65.0,70.0,75.0,80.0,85.0,90.0,95.0,100.0,105.0,110.0,115.0,120.0,125.0,130.0,135.0,140.0,145.0,150.0,155.0,160.0,165.0,170.0,175.0,180.0,185.0,190.0,195.0,200.0,205.0,210.0,215.0,220.0,225.0,230.0,235.0,240.0,245.0,250.0,255.0,260.0,265.0,270.0,275.0,280.0,285.0,290.0,295.0,300.0,305.0,310.0,315.0,320.0,325.0,330.0,335.0,340.0,345.0,350.0,355.0],"B1213":[0.0,5.0,10.0,15.0,20.0,25.0,30.0,35.0,40.0,45.0,50.0,55.0,60.0,65.0,70.0,75.0,80.0,85.0,90.0,95.0,100.0,105.0,110.0,115.0,120.0,125.0,130.0,135.0,140.0,145.0,150.0,155.0,160.0,165.0,170.0,175.0,180.0,185.0,190.0,195.0,200.0,205.0,210.0,215.0,220.0,225.0,230.0,235.0,240.0,245.0,250.0,255.0,260.0,265.0,270.0,275.0,280.0,285.0,290.0,295.0,300.0,305.0,310.0,315.0,320.0,325.0,330.0,335.0,340.0,345.0,350.0,355.0],"B1214":[0.0,5.0,10.0,15.0,20.0,25.0,30.0,35.0,40.0,45.0,50.0,55.0,60.0,65.0,70.0,75.0,80.0,85.0,90.0,95.0,100.0,105.0,110.0,115.0,120.0,125.0,130.0,135.0,140.0,145.0,150.0,155.0,160.0,165.0,170.0,175.0,180.0,185.0,190.0,195.0,200.0,205.0,210.0,215.0,220.0,225.0,230.0,235.0,240.0,245.0,250.0,255.0,260.0,265.0,270.0,275.0,280.0,285.0,290.0,295.0,300.0,305.0,310.0,315.0,320.0,325.0,330.0,335.0,340.0,345.0,350.0,355.0],"B1215":[0.0,5.0,10.0,15.0,20.0,25.0,30.0,35.0,40.0,45.0,50.0,55.0,60.0,65.0,70.0,75.0,80.0,85.0,90.0,95.0,100.0,105.0,110.0,115.0,120.0,125.0,130.0,135.0,140.0,145.0,150.0,155.0,160.0,165.0,170.0,175.0,180.0,185.0,190.0,195.0,200.0,205.0,210.0,215.0,220.0,225.0,230.0,235.0,240.0,245.0,250.0,255.0,260.0,265.0,270.0,275.0,280.0,285.0,290.0,295.0,300.0,305.0,310.0,315.0,320.0,325.0,330.0,335.0,340.0,345.0,350.0,355.0],"B1216":[0.0,5.0,10.0,15.0,20.0,25.0,30.0,35.0,40.0,45.0,50.0,55.0,60.0,65.0,70.0,75.0,80.0,85.0,90.0,95.0,100.0,105.0,110.0,115.0,120.0,125.0,130.0,135.0,140.0,145.0,150.0,155.0,160.0,165.0,170.0,175.0,180.0,185.0,190.0,195.0,200.0,205.0,210.0,215.0,220.0,225.0,230.0,235.0,240.0,245.0,250.0,255.0,260.0,265.0,270.0,275.0,280.0,285.0,290.0,295.0,300.0,305.0,310.0,315.0,320.0,325.0,330.0,335.0,340.0,345.0,350.0,355.0],"B1217":[0.0,5.0,10.0,15.0,20.0,25.0,30.0,35.0,40.0,45.0,50.0,55.0,60.0,65.0,70.0,75.0,80.0,85.0,90.0,95.0,100.0,105.0,110.0,115.0,120.0,125.0,130.0,135.0,140.0,145.0,150.0,155.0,160.0,165.0,170.0,175.0,180.0,185.0,190.0,195.0,200.0,205.0,210.0,215.0,220.0,225.0,230.0,235.0,240.0,245.0,250.0,255.0,260.0,265.0,270.0,275.0,280.0,285.0,290.0,295.0,300.0,305.0,310.0,315.0,320.0,325.0,330.0,335.0,340.0,345.0,350.0,355.0],"B1218":[0.0,5.0,10.0,15.0,20.0,25.0,30.0,35.0,40.0,45.0,50.0,55.0,60.0,65.0,70.0,75.0,80.0,85.0,90.0,95.0,100.0,105.0,110.0,115.0,120.0,125.0,130.0,135.0,140.0,145.0,150.0,155.0,160.0,165.0,170.0,175.0,180.0,185.0,190.0,195.0,200.0,205.0,210.0,215.0,220.0,225.0,230.0,235.0,240.0,245.0,250.0,255.0,260.0,265.0,270.0,275.0,280.0,285.0,290.0,295.0,300.0,305.0,310.0,315.0,320.0,325.0,330.0,335.0,340.0,345.0,350.0,355.0],"B1219":[0.0,5.0,10.0,15.0,20.0,25.0,30.0,35.0,40.0,45.0,50.0,55.0,60.0,65.0,70.0,75.0,80.0,85.0,90.0,95.0,100.0,105.0,110.0,115.0,120.0,125.0,130.0,135.0,140.0,145.0,150.0,155.0,160.0,165.0,170.0,175.0,180.0,185.0,190.0,195.0,200.0,205.0,210.0,215.0,220.0,225.0,230.0,235.0,240.0,245.0,250.0,255.0,260.0,265.0,270.0,275.0,280.0,285.0,290.0,295.0,300.0,305.0,310.0,315.0,320.0,325.0,330.0,335.0,340.0,345.0,350.0,355.0],"B1220":[0.0,5.0,10.0,15.0,20.0,25.0,30.0,35.0,40.0,45.0,50.0,55.0,60.0,65.0,70.0,75.0,80.0,85.0,90.0,95.0,100.0,105.0,110.0,115.0,120.0,125.0,130.0,135.0,140.0,145.0,150.0,155.0,160.0,165.0,170.0,175.0,180.0,185.0,190.0,195.0,200.0,205.0,210.0,215.0,220.0,225.0,230.0,235.0,240.0,245.0,250.0,255.0,260.0,265.0,270.0,275.0,280.0,285.0,290.0,295.0,300.0,305.0,310.0,315.0,320.0,325.0,330.0,335.0,340.0,345.0,350.0,355.0],"B1221":[0.0,5.0,10.0,15.0,20.0,25.0,30.0,35.0,40.0,45.0,50.0,55.0,60.0,65.0,70.0,75.0,80.0,85.0,90.0,95.0,100.0,105.0,110.0,115.0,120.0,125.0,130.0,135.0,140.0,145.0,150.0,155.0,160.0,165.0,170.0,175.0,180.0,185.0,190.0,195.0,200.0,205.0,210.0,215.0,220.0,225.0,230.0,235.0,240.0,245.0,250.0,255.0,260.0,265.0,270.0,275.0,280.0,285.0,290.0,295.0,300.0,305.0,310.0,315.0,320.0,325.0,330.0,335.0,340.0,345.0,350.0,355.0],"B1222":[0.0,5.0,10.0,15.0,20.0,25.0,30.0,35.0,40.0,45.0,50.0,55.0,60.0,65.0,70.0,75.0,80.0,85.0,90.0,95.0,100.0,105.0,110.0,115.0,120.0,125.0,130.0,135.0,140.0,145.0,150.0,155.0,160.0,165.0,170.0,175.0,180.0,185.0,190.0,195.0,200.0,205.0,210.0,215.0,220.0,225.0,230.0,235.0,240.0,245.0,250.0,255.0,260.0,265.0,270.0,275.0,280.0,285.0,290.0,295.0,300.0,305.0,310.0,315.0,320.0,325.0,330.0,335.0,340.0,345.0,350.0,355.0],"B1223":[0.0,5.0,10.0,15.0,20.0,25.0,30.0,35.0,40.0,45.0,50.0,55.0,60.0,65.0,70.0,75.0,80.0,85.0,90.0,95.0,100.0,105.0,110.0,115.0,120.0,125.0,130.0,135.0,140.0,145.0,150.0,155.0,160.0,165.0,170.0,175.0,180.0,185.0,190.0,195.0,200.0,205.0,210.0,215.0,220.0,225.0,230.0,235.0,240.0,245.0,250.0,255.0,260.0,265.0,270.0,275.0,280.0,285.0,290.0,295.0,300.0,305.0,310.0,315.0,320.0,325.0,330.0,335.0,340.0,345.0,350.0,355.0],"B1224":[0.0,5.0,10.0,15.0,20.0,25.0,30.0,35.0,40.0,45.0,50.0,55.0,60.0,65.0,70.0,75.0,80.0,85.0,90.0,95.0,100.0,105.0,110.0,115.0,120.0,125.0,130.0,135.0,140.0,145.0,150.0,155.0,160.0,165.0,170.0,175.0,180.0,185.0,190.0,195.0,200.0,205.0,210.0,215.0,220.0,225.0,230.0,235.0,240.0,245.0,250.0,255.0,260.0,265.0,270.0,275.0,280.0,285.0,290.0,295.0,300.0,305.0,310.0,315.0,320.0,325.0,330.0,335.0,340.0,345.0,350.0,355.0],"B1225":[0.0,5.0,10.0,15.0,20.0,25.0,30.0,35.0,40.0,45.0,50.0,55.0,60.0,65.0,70.0,75.0,80.0,85.0,90.0,95.0,100.0,105.0,110.0,115.0,120.0,125.0,130.0,135.0,140.0,145.0,150.0,155.0,160.0,165.0,170.0,175.0,180.0,185.0,190.0,195.0,200.0,205.0,210.0,215.0,220.0,225.0,230.0,235.0,240.0,245.0,250.0,255.0,260.0,265.0,270.0,275.0,280.0,285.0,290.0,295.0,300.0,305.0,310.0,315.0,320.0,325.0,330.0,335.0,340.0,345.0,350.0,355.0],"B1226":[0.0,5.0,10.0,15.0,20.0,25.0,30.0,35.0,40.0,45.0,50.0,55.0,60.0,65.0,70.0,75.0,80.0,85.0,90.0,95.0,100.0,105.0,110.0,115.0,120.0,125.0,130.0,135.0,140.0,145.0,150.0,155.0,160.0,165.0,170.0,175.0,180.0,185.0,190.0,195.0,200.0,205.0,210.0,215.0,220.0,225.0,230.0,235.0,240.0,245.0,250.0,255.0,260.0,265.0,270.0,275.0,280.0,285.0,290.0,295.0,300.0,305.0,310.0,315.0,320.0,325.0,330.0,335.0,340.0,345.0,350.0,355.0],"B1227":[0.0,5.0,10.0,15.0,20.0,25.0,30.0,35.0,40.0,45.0,50.0,55.0,60.0,65.0,70.0,75.0,80.0,85.0,90.0,95.0,100.0,105.0,110.0,115.0,120.0,125.0,130.0,135.0,140.0,145.0,150.0,155.0,160.0,165.0,170.0,175.0,180.0,185.0,190.0,195.0,200.0,205.0,210.0,215.0,220.0,225.0,230.0,235.0,240.0,245.0,250.0,255.0,260.0,265.0,270.0,275.0,280.0,285.0,290.0,295.0,300.0,305.0,310.0,315.0,320.0,325.0,330.0,335.0,340.0,345.0,350.0,355.0],"B1228":[0.0,5.0,10.0,15.0,20.0,25.0,30.0,35.0,40.0,45.0,50.0,55.0,60.0,65.0,70.0,75.0,80.0,85.0,90.0,95.0,100.0,105.0,110.0,115.0,120.0,125.0,130.0,135.0,140.0,145.0,150.0,155.0,160.0,165.0,170.0,175.0,180.0,185.0,190.0,195.0,200.0,205.0,210.0,215.0,220.0,225.0,230.0,235.0,240.0,245.0,250.0,255.0,260.0,265.0,270.0,275.0,280.0,285.0,290.0,295.0,300.0,305.0,310.0,315.0,320.0,325.0,330.0,335.0,340.0,345.0,350.0,355.0],"B1229":[0.0,5.0,10.0,15.0,20.0,25.0,30.0,35.0,40.0,45.0,50.0,55.0,60.0,65.0,70.0,75.0,80.0,85.0,90.0,95.0,100.0,105.0,110.0,115.0,120.0,125.0,130.0,135.0,140.0,145.0,150.0,155.0,160.0,165.0,170.0,175.0,180.0,185.0,190.0,195.0,200.0,205.0,210.0,215.0,220.0,225.0,230.0,235.0,240.0,245.0,250.0,255.0,260.0,265.0,270.0,275.0,280.0,285.0,290.0,295.0,300.0,305.0,310.0,315.0,320.0,325.0,330.0,335.0,340.0,345.0,350.0,355.0],"B1230":[0.0,5.0,10.0,15.0,20.0,25.0,30.0,35.0,40.0,45.0,50.0,55.0,60.0,65.0,70.0,75.0,80.0,85.0,90.0,95.0,100.0,105.0,110.0,115.0,120.0,125.0,130.0,135.0,140.0,145.0,150.0,155.0,160.0,165.0,170.0,175.0,180.0,185.0,190.0,195.0,200.0,205.0,210.0,215.0,220.0,225.0,230.0,235.0,240.0,245.0,250.0,255.0,260.0,265.0,270.0,275.0,280.0,285.0,290.0,295.0,300.0,305.0,310.0,315.0,320.0,325.0,330.0,335.0,340.0,345.0,350.0,355.0],"B1231":[0.0,5.0,10.0,15.0,20.0,25.0,30.0,35.0,40.0,45.0,50.0,55.0,60.0,65.0,70.0,75.0,80.0,85.0,90.0,95.0,100.0,105.0,110.0,115.0,120.0,125.0,130.0,135.0,140.0,145.0,150.0,155.0,160.0,165.0,170.0,175.0,180.0,185.0,190.0,195.0,200.0,205.0,210.0,215.0,220.0,225.0,230.0,235.0,240.0,245.0,250.0,255.0,260.0,265.0,270.0,275.0,280.0,285.0,290.0,295.0,300.0,305.0,310.0,315.0,320.0,325.0,330.0,335.0,340.0,345.0,350.0,355.0],"B1232":[0.0,5.0,10.0,15.0,20.0,25.0,30.0,35.0,40.0,45.0,50.0,55.0,60.0,65.0,70.0,75.0,80.0,85.0,90.0,95.0,100.0,105.0,110.0,115.0,120.0,125.0,130.0,135.0,140.0,145.0,150.0,155.0,160.0,165.0,170.0,175.0,180.0,185.0,190.0,195.0,200.0,205.0,210.0,215.0,220.0,225.0,230.0,235.0,240.0,245.0,250.0,255.0,260.0,265.0,270.0,275.0,280.0,285.0,290.0,295.0,300.0,305.0,310.0,315.0,320.0,325.0,330.0,335.0,340.0,345.0,350.0,355.0],"B1233":[0.0,5.0,10.0,15.0,20.0,25.0,30.0,35.0,40.0,45.0,50.0,55.0,60.0,65.0,70.0,75.0,80.0,85.0,90.0,95.0,100.0,105.0,110.0,115.0,120.0,125.0,130.0,135.0,140.0,145.0,150.0,155.0,160.0,165.0,170.0,175.0,180.0,185.0,190.0,195.0,200.0,205.0,210.0,215.0,220.0,225.0,230.0,235.0,240.0,245.0,250.0,255.0,260.0,265.0,270.0,275.0,280.0,285.0,290.0,295.0,300.0,305.0,310.0,315.0,320.0,325.0,330.0,335.0,340.0,345.0,350.0,355.0],"B1234":[0.0,5.0,10.0,15.0,20.0,25.0,30.0,35.0,40.0,45.0,50.0,55.0,60.0,65.0,70.0,75.0,80.0,85.0,90.0,95.0,100.0,105.0,110.0,115.0,120.0,125.0,130.0,135.0,140.0,145.0,150.0,155.0,160.0,165.0,170.0,175.0,180.0,185.0,190.0,195.0,200.0,205.0,210.0,215.0,220.0,225.0,230.0,235.0,240.0,245.0,250.0,255.0,260.0,265.0,270.0,275.0,280.0,285.0,290.0,295.0,300.0,305.0,310.0,315.0,320.0,325.0,330.0,335.0,340.0,345.0,350.0,355.0],"B1235":[0.0,5.0,10.0,15.0,20.0,25.0,30.0,35.0,40.0,45.0,50.0,55.0,60.0,65.0,70.0,75.0,80.0,85.0,90.0,95.0,100.0,105.0,110.0,115.0,120.0,125.0,130.0,135.0,140.0,145.0,150.0,155.0,160.0,165.0,170.0,175.0,180.0,185.0,190.0,195.0,200.0,205.0,210.0,215.0,220.0,225.0,230.0,235.0,240.0,245.0,250.0,255.0,260.0,265.0,270.0,275.0,280.0,285.0,290.0,295.0,300.0,305.0,310.0,315.0,320.0,325.0,330.0,335.0,340.0,345.0,350.0,355.0],"B1236":[0.0,5.0,10.0,15.0,20.0,25.0,30.0,35.0,40.0,45.0,50.0,55.0,60.0,65.0,70.0,75.0,80.0,85.0,90.0,95.0,100.0,105.0,110.0,115.0,120.0,125.0,130.0,135.0,140.0,145.0,150.0,155.0,160.0,165.0,170.0,175.0,180.0,185.0,190.0,195.0,200.0,205.0,210.0,215.0,220.0,225.0,230.0,235.0,240.0,245.0,250.0,255.0,260.0,265.0,270.0,275.0,280.0,285.0,290.0,295.0,300.0,305.0,310.0,315.0,320.0,325.0,330.0,335.0,340.0,345.0,350.0,355.0],"B1237":[0.0,5.0,10.0,15.0,20.0,25.0,30.0,35.0,40.0,45.0,50.0,55.0,60.0,65.0,70.0,75.0,80.0,85.0,90.0,95.0,100.0,105.0,110.0,115.0,120.0,125.0,130.0,135.0,140.0,145.0,150.0,155.0,160.0,165.0,170.0,175.0,180.0,185.0,190.0,195.0,200.0,205.0,210.0,215.0,220.0,225.0,230.0,235.0,240.0,245.0,250.0,255.0,260.0,265.0,270.0,275.0,280.0,285.0,290.0,295.0,300.0,305.0,310.0,315.0,320.0,325.0,330.0,335.0,340.0,345.0,350.0,355.0],"B1238":[0.0,5.0,10.0,15.0,20.0,25.0,30.0,35.0,40.0,45.0,50.0,55.0,60.0,65.0,70.0,75.0,80.0,85.0,90.0,95.0,100.0,105.0,110.0,115.0,120.0,125.0,130.0,135.0,140.0,145.0,150.0,155.0,160.0,165.0,170.0,175.0,180.0,185.0,190.0,195.0,200.0,205.0,210.0,215.0,220.0,225.0,230.0,235.0,240.0,245.0,250.0,255.0,260.0,265.0,270.0,275.0,280.0,285.0,290.0,295.0,300.0,305.0,310.0,315.0,320.0,325.0,330.0,335.0,340.0,345.0,350.0,355.0],"B1239":[0.0,5.0,10.0,15.0,20.0,25.0,30.0,35.0,40.0,45.0,50.0,55.0,60.0,65.0,70.0,75.0,80.0,85.0,90.0,95.0,100.0,105.0,110.0,115.0,120.0,125.0,130.0,135.0,140.0,145.0,150.0,155.0,160.0,165.0,170.0,175.0,180.0,185.0,190.0,195.0,200.0,205.0,210.0,215.0,220.0,225.0,230.0,235.0,240.0,245.0,250.0,255.0,260.0,265.0,270.0,275.0,280.0,285.0,290.0,295.0,300.0,305.0,310.0,315.0,320.0,325.0,330.0,335.0,340.0,345.0,350.0,355.0],"B1240":[0.0,5.0,10.0,15.0,20.0,25.0,30.0,35.0,40.0,45.0,50.0,55.0,60.0,65.0,70.0,75.0,80.0,85.0,90.0,95.0,100.0,105.0,110.0,115.0,120.0,125.0,130.0,135.0,140.0,145.0,150.0,155.0,160.0,165.0,170.0,175.0,180.0,185.0,190.0,195.0,200.0,205.0,210.0,215.0,220.0,225.0,230.0,235.0,240.0,245.0,250.0,255.0,260.0,265.0,270.0,275.0,280.0,285.0,290.0,295.0,300.0,305.0,310.0,315.0,320.0,325.0,330.0,335.0,340.0,345.0,350.0,355.0],"K1401":[0.0,5.0,10.0,15.0,20.0,25.0,30.0,35.0,40.0,45.0,50.0,55.0,60.0,65.0,70.0,75.0,80.0,85.0,90.0,95.0,100.0,105.0,110.0,115.0,120.0,125.0,130.0,135.0,140.0,145.0,150.0,155.0,160.0,165.0,170.0,175.0,180.0,185.0,190.0,195.0,200.0,205.0,210.0,215.0,220.0,225.0,230.0,235.0,240.0,245.0,250.0,255.0,260.0,265.0,270.0,275.0,280.0,285.0,290.0,295.0,300.0,305.0,310.0,315.0,320.0,325.0,330.0,335.0,340.0,345.0,350.0,355.0],"L1501":[0.0,5.0,10.0,15.0,20.0,25.0,30.0,35.0,40.0,45.0,50.0,55.0,60.0,65.0,70.0,75.0,80.0,85.0,90.0,95.0,100.0,105.0,110.0,115.0,120.0,125.0,130.0,135.0,140.0,145.0,150.0,155.0,160.0,165.0,170.0,175.0,180.0,185.0,190.0,195.0,200.0,205.0,210.0,215.0,220.0,225.0,230.0,235.0,240.0,245.0,250.0,255.0,260.0,265.0,270.0,275.0,280.0,285.0,290.0,295.0,300.0,305.0,310.0,315.0,320.0,325.0,330.0,335.0,340.0,345.0,350.0,355.0],"L1502":[0.0,5.0,10.0,15.0,20.0,25.0,30.0,35.0,40.0,45.0,50.0,55.0,60.0,65.0,70.0,75.0,80.0,85.0,90.0,95.0,100.0,105.0,110.0,115.0,120.0,125.0,130.0,135.0,140.0,145.0,150.0,155.0,160.0,165.0,170.0,175.0,180.0,185.0,190.0,195.0,200.0,205.0,210.0,215.0,220.0,225.0,230.0,235.0,240.0,245.0,250.0,255.0,260.0,265.0,270.0,275.0,280.0,285.0,290.0,295.0,300.0,305.0,310.0,315.0,320.0,325.0,330.0,335.0,340.0,345.0,350.0,355.0],"L1503":[0.0,5.0,10.0,15.0,20.0,25.0,30.0,35.0,40.0,45.0,50.0,55.0,60.0,65.0,70.0,75.0,80.0,85.0,90.0,95.0,100.0,105.0,110.0,115.0,120.0,125.0,130.0,135.0,140.0,145.0,150.0,155.0,160.0,165.0,170.0,175.0,180.0,185.0,190.0,195.0,200.0,205.0,210.0,215.0,220.0,225.0,230.0,235.0,240.0,245.0,250.0,255.0,260.0,265.0,270.0,275.0,280.0,285.0,290.0,295.0,300.0,305.0,310.0,315.0,320.0,325.0,330.0,335.0,340.0,345.0,350.0,355.0],"M1501":[0.0,5.0,10.0,15.0,20.0,25.0,30.0,35.0,40.0,45.0,50.0,55.0,60.0,65.0,70.0,75.0,80.0,85.0,90.0,95.0,100.0,105.0,110.0,115.0,120.0,125.0,130.0,135.0,140.0,145.0,150.0,155.0,160.0,165.0,170.0,175.0,180.0,185.0,190.0,195.0,200.0,205.0,210.0,215.0,220.0,225.0,230.0,235.0,240.0,245.0,250.0,255.0,260.0,265.0,270.0,275.0,280.0,285.0,290.0,295.0,300.0,305.0,310.0,315.0,320.0,325.0,330.0,335.0,340.0,345.0,350.0,355.0]},"scan_amp_C2":{"A1011":[0.0129,0.01332,0.01395,0.01476,0.0157,0.0167,0.01772,0.01865,0.01942,0.01998,0.02025,0.0202,0.01979,0.01904,0.01795,0.01656,0.01494,0.01315,0.01131,0.00951,0.00788,0.00649,0.00548,0.0049,0.00484,0.0053,0.00628,0.00774,0.00959,0.01172,0.01399,0.01623,0.01832,0.02009,0.02144,0.02229,0.02259,0.02236,0.02165,0.02052,0.01909,0.01748,0.01578,0.01414,0.01263,0.01132,0.01028,0.00953,0.00908,0.00892,0.00903,0.00939,0.00996,0.01066,0.01147,0.01233,0.01317,0.01396,0.01463,0.01517,0.01553,0.0157,0.01569,0.0155,0.01517,0.01473,0.01423,0.01373,0.01327,0.01291,0.01272,0.0127],"A1012":[-0.02389,-0.02806,-0.03202,-0.03567,-0.03892,-0.04172,-0.04402,-0.04579,-0.04699,-0.04766,-0.0478,-0.04745,-0.04668,-0.04554,-0.04411,-0.04244,-0.04066,-0.03881,-0.03698,-0.03524,-0.03364,-0.0322,-0.03095,-0.02987,-0.02891,-0.02802,-0.02709,-0.02603,-0.02473,-0.02306,-0.02096,-0.01835,-0.0152,-0.0115,-0.00732,-0.00275,0.00209,0.00704,0.01193,0.01665,0.021,0.02489,0.02824,0.03098,0.03308,0.03457,0.03549,0.03589,0.03587,0.03546,0.03475,0.03381,0.03269,0.03144,0.03006,0.02861,0.02706,0.0254,0.0236,0.02167,0.01955,0.0172,0.01464,0.01178,0.00866,0.00523,0.00156,-0.00236,-0.00648,-0.01078,-0.01516,-0.01955],"A1013":[0.02966,0.03034,0.03056,0.03032,0.02962,0.02848,0.02693,0.02502,0.0228,0.02032,0.01766,0.01488,0.01204,0.00925,0.00657,0.00407,0.00186,-0.00002,-0.00148,-0.00243,-0.00285,-0.00268,-0.00188,-0.00045,0.00164,0.00433,0.0076,0.0114,0.01559,0.02008,0.0247,0.02929,0.03365,0.03763,0.04097,0.04355,0.04519,0.04579,0.0453,0.0437,0.04107,0.03753,0.03318,0.02829,0.02305,0.01764,0.01233,0.00732,0.00273,-0.00125,-0.00455,-0.00709,-0.00887,-0.00988,-0.01016,-0.00976,-0.00875,-0.00721,-0.00523,-0.00289,-0.00025,0.00259,0.00556,0.00864,0.0117,0.01473,0.01763,0.02036,0.02287,0.02511,0.02701,0.02854],"A1101":[-0.00236,-0.00218,-0.00194,-0.00164,-0.00129,-0.0009,-0.00049,-0.00008,0.00033,0.00072,0.00108,0.0014,0.00167,0.00189,0.00205,0.00217,0.00224,0.00225,0.00223,0.00216,0.00207,0.00196,0.00184,0.00173,0.00164,0.00159,0.00159,0.00165,0.00177,0.00196,0.00221,0.00252,0.00288,0.00327,0.00366,0.00406,0.00443,0.00476,0.00504,0.00527,0.00543,0.00553,0.00557,0.00555,0.00547,0.00535,0.00517,0.00496,0.00472,0.00444,0.00413,0.00379,0.00343,0.00305,0.00265,0.00224,0.00181,0.00137,0.00094,0.0005,0.00007,-0.00034,-0.00074,-0.00111,-0.00145,-0.00175,-0.00201,-0.00222,-0.00238,-0.00247,-0.0025,-0.00247],"A1102":[0.03146,0.03127,0.03077,0.02996,0.02889,0.02761,0.02616,0.0246,0.02299,0.02136,0.01978,0.01827,0.01685,0.01552,0.0143,0.01318,0.01215,0.01119,0.01029,0.00944,0.00862,0.00783,0.00706,0.00633,0.00562,0.00494,0.00431,0.00372,0.00316,0.00266,0.00218,0.00172,0.00127,0.00082,0.00036,-0.00011,-0.00058,-0.00103,-0.00145,-0.00181,-0.00209,-0.00223,-0.00223,-0.00207,-0.00173,-0.00121,-0.00052,0.00032,0.0013,0.00237,0.00355,0.00479,0.00608,0.0074,0.00875,0.01013,0.01152,0.01295,0.0144,0.01588,0.01741,0.01895,0.02052,0.02211,0.02369,0.02522,0.02668,0.02802,0.0292,0.03018,0.03089,0.03134],"A1103":[0.00222,0.00235,0.00249,0.00269,0.00298,0.00339,0.00394,0.00464,0.00548,0.00644,0.00749,0.00861,0.00975,0.01088,0.01199,0.01304,0.01402,0.01491,0.01573,0.01645,0.01708,0.01764,0.01813,0.01856,0.01896,0.01933,0.01971,0.02009,0.0205,0.02093,0.02136,0.02178,0.02216,0.02245,0.02261,0.02261,0.02239,0.02195,0.02126,0.02032,0.01914,0.01776,0.01622,0.01456,0.01283,0.01105,0.00929,0.00757,0.00592,0.00438,0.00296,0.00166,0.00052,-0.00047,-0.0013,-0.00195,-0.00244,-0.00275,-0.00288,-0.00285,-0.00267,-0.00235,-0.00192,-0.00141,-0.00084,-0.00026,0.0003,0.00081,0.00125,0.00161,0.00188,0.00208],"A1104":[0.00868,0.00946,0.01018,0.01081,0.01134,0.01178,0.01212,0.01234,0.01246,0.01248,0.01241,0.01227,0.01209,0.01189,0.01169,0.01153,0.01142,0.01139,0.01145,0.01161,0.01186,0.01222,0.01268,0.01323,0.01387,0.0146,0.01541,0.0163,0.01725,0.01823,0.01923,0.0202,0.0211,0.0219,0.02253,0.02296,0.02315,0.02308,0.02273,0.0221,0.02123,0.02014,0.01886,0.01745,0.01595,0.01439,0.01282,0.01127,0.00976,0.00832,0.00697,0.0057,0.00455,0.00351,0.0026,0.00183,0.00121,0.00075,0.00044,0.0003,0.00033,0.00051,0.00084,0.00132,0.00192,0.00262,0.00342,0.00426,0.00515,0.00606,0.00696,0.00784],"A1105":[0.0164,0.01512,0.01378,0.01245,0.01116,0.00993,0.0088,0.00778,0.00687,0.00609,0.00542,0.00487,0.00443,0.00411,0.0039,0.00382,0.00388,0.00407,0.00441,0.0049,0.00553,0.00632,0.00723,0.00826,0.00937,0.01052,0.01169,0.01283,0.01388,0.01481,0.01556,0.01609,0.01636,0.01635,0.01604,0.01545,0.01457,0.01344,0.01212,0.01065,0.00911,0.00756,0.00606,0.00469,0.0035,0.00253,0.00182,0.00139,0.00126,0.00141,0.00184,0.00256,0.00351,0.00467,0.00602,0.0075,0.00908,0.01073,0.01237,0.01397,0.0155,0.0169,0.01811,0.01912,0.01988,0.02037,0.02058,0.02049,0.02013,0.0195,0.01864,0.01759],"A1106":[0.01871,0.01758,0.01637,0.01512,0.01389,0.0127,0.01156,0.01052,0.00958,0.00875,0.00805,0.00748,0.00706,0.00677,0.00663,0.00663,0.00676,0.00703,0.00742,0.00792,0.0085,0.00916,0.00987,0.01061,0.01136,0.01207,0.01274,0.01333,0.01381,0.01414,0.01428,0.01421,0.01388,0.01328,0.01239,0.01121,0.00975,0.00805,0.00615,0.00413,0.00205,0.00001,-0.00192,-0.00367,-0.00516,-0.00633,-0.00715,-0.00759,-0.00763,-0.00726,-0.00651,-0.00538,-0.00392,-0.00217,-0.00016,0.00202,0.00436,0.00675,0.00915,0.01148,0.01368,0.0157,0.01746,0.01896,0.02015,0.021,0.02153,0.02173,0.02162,0.02122,0.02058,0.01973],"A1107":[-0.00761,-0.00722,-0.00663,-0.00586,-0.00491,-0.00382,-0.00261,-0.00132,0.00002,0.00138,0.00271,0.004,0.00521,0.00631,0.00732,0.00819,0.00894,0.00956,0.01005,0.01041,0.01065,0.01077,0.01078,0.0107,0.01052,0.01026,0.00992,0.00953,0.00907,0.00857,0.00802,0.00744,0.00682,0.00618,0.00552,0.00485,0.00419,0.00355,0.00295,0.00242,0.00195,0.00157,0.00128,0.00108,0.00097,0.00091,0.00091,0.00094,0.00098,0.00101,0.00101,0.00097,0.00089,0.00074,0.00053,0.00026,-0.00008,-0.00049,-0.00096,-0.0015,-0.0021,-0.00276,-0.00345,-0.00416,-0.00488,-0.00558,-0.00623,-0.00681,-0.00728,-0.00762,-0.0078,-0.0078],"A1108":[0.00583,0.00383,0.00173,-0.00045,-0.00269,-0.00497,-0.00728,-0.00957,-0.01184,-0.01407,-0.01621,-0.01823,-0.02012,-0.02182,-0.02333,-0.02461,-0.02563,-0.02639,-0.02687,-0.02706,-0.02695,-0.02654,-0.02582,-0.0248,-0.02348,-0.02187,-0.01998,-0.0178,-0.0154,-0.01278,-0.01001,-0.00713,-0.0042,-0.00126,0.00156,0.00427,0.00679,0.00906,0.01109,0.01285,0.01433,0.01555,0.01655,0.01733,0.01795,0.01842,0.01878,0.01907,0.01929,0.01947,0.01962,0.01975,0.01987,0.01996,0.02004,0.02009,0.02009,0.02005,0.01995,0.01976,0.01949,0.01911,0.01862,0.01799,0.01723,0.01632,0.01526,0.01405,0.01269,0.01117,0.00952,0.00774],"A1109":[0.00221,0.00199,0.00174,0.00147,0.00123,0.00103,0.00089,0.00085,0.00093,0.00112,0.00143,0.00186,0.00239,0.003,0.00368,0.00439,0.00511,0.00581,0.00647,0.00706,0.00757,0.00799,0.0083,0.0085,0.0086,0.0086,0.0085,0.00834,0.00811,0.00783,0.00753,0.00722,0.00691,0.00661,0.00633,0.00609,0.00588,0.00571,0.00558,0.00549,0.00543,0.00538,0.00534,0.00531,0.00525,0.00516,0.00503,0.00485,0.00462,0.00434,0.00401,0.00365,0.00326,0.00286,0.00248,0.00212,0.00181,0.00156,0.00138,0.00128,0.00125,0.0013,0.00142,0.00158,0.00178,0.00198,0.00218,0.00234,0.00245,0.0025,0.00247,0.00238],"A1110":[0.01964,0.0204,0.02112,0.0218,0.02245,0.02304,0.0236,0.0241,0.02456,0.02495,0.02529,0.02556,0.02576,0.02588,0.02592,0.02587,0.02574,0.02553,0.02523,0.02485,0.02441,0.0239,0.02333,0.02272,0.02206,0.02138,0.02066,0.01993,0.01917,0.01837,0.01753,0.01664,0.01566,0.01458,0.01339,0.01208,0.01063,0.00908,0.00743,0.00571,0.00398,0.00227,0.00063,-0.00086,-0.00217,-0.00326,-0.00409,-0.00465,-0.00491,-0.00488,-0.00457,-0.004,-0.0032,-0.0022,-0.00104,0.00023,0.00159,0.00301,0.00443,0.00584,0.00723,0.00856,0.00983,0.01105,0.0122,0.01329,0.01433,0.01531,0.01626,0.01716,0.01802,0.01885],"A1111":[0.01196,0.01226,0.01259,0.01293,0.01323,0.01347,0.01363,0.01367,0.01356,0.0133,0.01287,0.01225,0.01146,0.0105,0.00937,0.0081,0.00671,0.00523,0.00368,0.00211,0.00053,-0.00102,-0.0025,-0.00389,-0.00516,-0.00627,-0.0072,-0.00794,-0.00847,-0.00879,-0.00891,-0.00883,-0.00859,-0.00821,-0.00774,-0.0072,-0.00665,-0.0061,-0.00558,-0.00511,-0.00467,-0.00425,-0.00384,-0.00339,-0.00289,-0.0023,-0.0016,-0.00078,0.00018,0.00124,0.0024,0.00363,0.00488,0.00613,0.00732,0.00843,0.0094,0.01024,0.01091,0.01141,0.01175,0.01193,0.01199,0.01195,0.01184,0.01169,0.01155,0.01144,0.01139,0.01142,0.01152,0.0117],"A1112":[-0.01061,-0.01175,-0.01281,-0.01379,-0.01468,-0.01549,-0.01622,-0.01688,-0.01748,-0.01804,-0.01856,-0.01907,-0.01955,-0.02,-0.02042,-0.02078,-0.02107,-0.02125,-0.02128,-0.02115,-0.02081,-0.02023,-0.0194,-0.01829,-0.01688,-0.01519,-0.01321,-0.01097,-0.00851,-0.00587,-0.00312,-0.00032,0.00245,0.00514,0.00762,0.00984,0.01178,0.01334,0.01454,0.01536,0.01579,0.01588,0.01565,0.01515,0.01443,0.01354,0.01253,0.01144,0.01032,0.00923,0.00816,0.00717,0.00627,0.00545,0.00473,0.00409,0.00352,0.003,0.0025,0.00199,0.00146,0.00087,0.0002,-0.00055,-0.0014,-0.00236,-0.0034,-0.00453,-0.00571,-0.00694,-0.00818,-0.00941],"A1113":[0.01435,0.01451,0.01454,0.01439,0.01404,0.01349,0.01272,0.01174,0.01059,0.00927,0.00784,0.00635,0.00483,0.00335,0.00194,0.00063,-0.0005,-0.00146,-0.00222,-0.00275,-0.00306,-0.00316,-0.00305,-0.00274,-0.00228,-0.00168,-0.00098,-0.00022,0.00054,0.00126,0.0019,0.00241,0.00276,0.00291,0.00286,0.0026,0.00214,0.00151,0.00075,-0.00009,-0.00095,-0.00177,-0.00249,-0.00305,-0.0034,-0.00353,-0.00341,-0.00304,-0.00244,-0.00164,-0.00067,0.00043,0.0016,0.0028,0.00399,0.00513,0.00619,0.00716,0.00802,0.00876,0.00941,0.00997,0.01045,0.01088,0.01129,0.01169,0.01209,0.0125,0.01291,0.01333,0.01372,0.01407],"A1114":[-0.00422,-0.00537,-0.00652,-0.00764,-0.0087,-0.00966,-0.01052,-0.01126,-0.01187,-0.01236,-0.01274,-0.01303,-0.01325,-0.01341,-0.01352,-0.01358,-0.01359,-0.01356,-0.01344,-0.01322,-0.01287,-0.01237,-0.01167,-0.01076,-0.00961,-0.00822,-0.00659,-0.00473,-0.00267,-0.00046,0.00185,0.00417,0.00644,0.00858,0.01051,0.01213,0.01342,0.01431,0.01479,0.01485,0.01452,0.01383,0.01283,0.01161,0.01022,0.00872,0.00722,0.00576,0.0044,0.00319,0.00215,0.00131,0.00069,0.00026,0.00003,-0.00004,0.00002,0.00019,0.00043,0.0007,0.00096,0.00119,0.00134,0.00141,0.00135,0.00115,0.00081,0.00031,-0.00034,-0.00115,-0.00208,-0.00311],"A1115":[0.02313,0.02608,0.02898,0.03181,0.03447,0.03694,0.0392,0.04117,0.04285,0.04421,0.04524,0.04594,0.04632,0.04637,0.04613,0.0456,0.0448,0.04376,0.04249,0.04102,0.03936,0.03752,0.03554,0.03341,0.03116,0.0288,0.02635,0.02381,0.0212,0.01853,0.01581,0.01307,0.01029,0.0075,0.00475,0.00204,-0.0006,-0.00312,-0.00549,-0.00769,-0.00968,-0.01143,-0.01295,-0.01419,-0.01517,-0.01591,-0.0164,-0.01666,-0.01671,-0.01657,-0.01626,-0.01579,-0.01517,-0.01442,-0.01354,-0.01254,-0.01142,-0.01018,-0.00883,-0.00737,-0.00578,-0.00407,-0.00223,-0.00026,0.00185,0.00409,0.00647,0.00899,0.01162,0.01439,0.01724,0.02016],"A1116":[0.0134,0.01356,0.01388,0.01427,0.01468,0.01503,0.01527,0.01535,0.01521,0.01484,0.01424,0.0134,0.01234,0.0111,0.0097,0.00816,0.0065,0.00475,0.0029,0.00098,-0.00101,-0.00309,-0.00521,-0.00738,-0.00957,-0.01171,-0.01377,-0.01569,-0.0174,-0.01885,-0.01995,-0.02065,-0.02092,-0.0207,-0.01999,-0.01879,-0.01711,-0.015,-0.01251,-0.00967,-0.00658,-0.0033,0.0001,0.00353,0.00694,0.01027,0.01346,0.01646,0.01923,0.0217,0.02391,0.02577,0.02728,0.02844,0.02922,0.02963,0.02968,0.02936,0.02871,0.02776,0.02656,0.02515,0.02361,0.02198,0.02035,0.01877,0.01732,0.01606,0.01502,0.01422,0.0137,0.01343],"A1117":[0.02174,0.01948,0.01639,0.01247,0.00784,0.00262,-0.00309,-0.00906,-0.01513,-0.02113,-0.02686,-0.03215,-0.0369,-0.04096,-0.04427,-0.0468,-0.04847,-0.04931,-0.04931,-0.0485,-0.0469,-0.04458,-0.04154,-0.03789,-0.03363,-0.02887,-0.02364,-0.01807,-0.01225,-0.00634,-0.00046,0.0052,0.01053,0.01532,0.01942,0.02272,0.02511,0.02656,0.02705,0.02663,0.02539,0.02343,0.02091,0.01798,0.01481,0.01154,0.00832,0.00531,0.00258,0.00027,-0.00161,-0.00297,-0.00381,-0.00414,-0.00396,-0.0033,-0.0022,-0.00072,0.00109,0.00316,0.00546,0.0079,0.01041,0.01295,0.01543,0.01775,0.01984,0.02158,0.0229,0.02366,0.02378,0.02315],"A1118":[0.00679,0.00852,0.01037,0.0123,0.01426,0.01622,0.01813,0.01995,0.02164,0.02314,0.02443,0.02547,0.02623,0.0267,0.02686,0.02673,0.0263,0.02562,0.02469,0.02356,0.02228,0.02088,0.01942,0.01793,0.01645,0.01503,0.01369,0.01244,0.0113,0.01027,0.00934,0.00851,0.00775,0.00705,0.00641,0.00581,0.00526,0.00475,0.0043,0.00391,0.00359,0.00335,0.00318,0.00308,0.00304,0.00305,0.00308,0.0031,0.0031,0.00306,0.00297,0.0028,0.00257,0.00226,0.00189,0.00148,0.00104,0.00058,0.00014,-0.00026,-0.0006,-0.00086,-0.00101,-0.00102,-0.00089,-0.00058,-0.0001,0.00057,0.00143,0.00251,0.00376,0.00519],"A1119":[0.00755,0.00815,0.00871,0.00922,0.00965,0.01004,0.01033,0.01053,0.01068,0.01074,0.01075,0.01071,0.01065,0.01056,0.01046,0.01032,0.01016,0.00996,0.0097,0.00939,0.009,0.00854,0.00801,0.00741,0.00679,0.00615,0.00554,0.00498,0.00454,0.00421,0.00404,0.00403,0.00418,0.00446,0.00485,0.00529,0.00575,0.00618,0.00651,0.00674,0.00682,0.00674,0.00651,0.00612,0.00562,0.00501,0.00434,0.00364,0.00294,0.00227,0.00166,0.00112,0.00067,0.00033,0.00009,-0.00003,-0.00005,0.00002,0.0002,0.00045,0.00078,0.00118,0.00163,0.00212,0.00266,0.00322,0.00381,0.00441,0.00504,0.00566,0.0063,0.00693],"A1120":[0.02119,0.02358,0.02558,0.02712,0.02816,0.02869,0.0287,0.02822,0.02731,0.02601,0.0244,0.02256,0.02057,0.01854,0.01651,0.01455,0.01276,0.01116,0.00981,0.00875,0.00799,0.00753,0.0074,0.00757,0.00803,0.00878,0.00977,0.01096,0.01229,0.0137,0.01514,0.0165,0.01774,0.01875,0.01947,0.01984,0.01982,0.01939,0.01856,0.01733,0.01577,0.01393,0.01186,0.00966,0.0074,0.00514,0.00295,0.00086,-0.00108,-0.00282,-0.00438,-0.00573,-0.00686,-0.00779,-0.0085,-0.00899,-0.00926,-0.00929,-0.00907,-0.00858,-0.0078,-0.00671,-0.0053,-0.00355,-0.00149,0.00089,0.00354,0.00639,0.00941,0.01249,0.01555,0.01848],"A1121":[0.02375,0.01949,0.01532,0.0113,0.00751,0.00398,0.00075,-0.00215,-0.00474,-0.00705,-0.00906,-0.01083,-0.01237,-0.01369,-0.01483,-0.01578,-0.01655,-0.01713,-0.0175,-0.01765,-0.01756,-0.0172,-0.01657,-0.01564,-0.01441,-0.01287,-0.01102,-0.00887,-0.00645,-0.00379,-0.00092,0.00208,0.00519,0.00834,0.01147,0.01452,0.01749,0.02031,0.02299,0.02551,0.02788,0.03011,0.03223,0.03424,0.03616,0.03802,0.03983,0.04157,0.04327,0.04491,0.04651,0.04805,0.0495,0.05086,0.05212,0.05323,0.05419,0.05495,0.05547,0.05571,0.05562,0.05517,0.05432,0.05303,0.0513,0.04911,0.04647,0.04342,0.03998,0.0362,0.0322,0.02802],"A1122":[0.01391,0.01687,0.0196,0.02206,0.02414,0.02583,0.02713,0.02801,0.0285,0.02864,0.02848,0.02808,0.0275,0.02682,0.02611,0.02542,0.02483,0.02436,0.02406,0.02394,0.02403,0.02431,0.02479,0.02543,0.02623,0.02715,0.02816,0.02925,0.03037,0.03147,0.03253,0.0335,0.03433,0.03499,0.03542,0.03558,0.03544,0.03497,0.03415,0.03297,0.03145,0.02962,0.02747,0.02509,0.02251,0.01977,0.01693,0.01405,0.01115,0.00831,0.00554,0.00288,0.00037,-0.00196,-0.00409,-0.00596,-0.00758,-0.00891,-0.00991,-0.01056,-0.01084,-0.01071,-0.01017,-0.0092,-0.0078,-0.00599,-0.0038,-0.00129,0.0015,0.00452,0.00764,0.0108],"A1123":[-0.01594,-0.0144,-0.01264,-0.0107,-0.00859,-0.00633,-0.00397,-0.00156,0.00085,0.00321,0.00546,0.00755,0.00947,0.01116,0.01262,0.01385,0.01483,0.01558,0.01611,0.01644,0.01658,0.01657,0.01642,0.01617,0.01584,0.01547,0.01507,0.01468,0.01431,0.01397,0.01365,0.01334,0.013,0.01261,0.01212,0.01149,0.0107,0.00972,0.00857,0.00723,0.00575,0.00418,0.00252,0.00086,-0.00079,-0.0024,-0.00395,-0.00541,-0.00682,-0.00816,-0.00945,-0.0107,-0.01192,-0.0131,-0.01427,-0.01538,-0.01644,-0.01743,-0.01834,-0.01914,-0.01982,-0.02037,-0.02078,-0.02104,-0.02116,-0.02112,-0.02092,-0.02056,-0.02002,-0.0193,-0.01839,-0.01727],"A1124":[0.02126,0.01841,0.01557,0.01284,0.01036,0.00821,0.00644,0.00512,0.00426,0.00385,0.00385,0.00422,0.00489,0.00579,0.00686,0.00802,0.00922,0.01039,0.0115,0.01251,0.01339,0.01415,0.01477,0.01529,0.01573,0.01612,0.01651,0.01696,0.0175,0.01817,0.019,0.02,0.02117,0.02248,0.02387,0.02529,0.02667,0.02794,0.02902,0.02987,0.03044,0.0307,0.03065,0.03032,0.02972,0.02893,0.02798,0.02696,0.02593,0.02499,0.02417,0.02356,0.02319,0.0231,0.02329,0.02377,0.02453,0.0255,0.02666,0.02791,0.02918,0.03039,0.03145,0.03227,0.03276,0.03286,0.03252,0.0317,0.0304,0.02864,0.02648,0.02399],"A1125":[-0.00911,-0.00782,-0.00617,-0.00419,-0.00192,0.00056,0.0032,0.0059,0.00859,0.01119,0.01363,0.01584,0.01781,0.01945,0.02079,0.02179,0.02246,0.0228,0.02282,0.02255,0.022,0.02119,0.02015,0.01889,0.01744,0.01584,0.0141,0.01223,0.01028,0.00826,0.00617,0.00406,0.0019,-0.00029,-0.00249,-0.00468,-0.00684,-0.00894,-0.01095,-0.01282,-0.01449,-0.01593,-0.01708,-0.01791,-0.01838,-0.01848,-0.01823,-0.01763,-0.01674,-0.01562,-0.01432,-0.01292,-0.0115,-0.01013,-0.00887,-0.00779,-0.00692,-0.0063,-0.00595,-0.00586,-0.00602,-0.00639,-0.00694,-0.00762,-0.00836,-0.00911,-0.00979,-0.01034,-0.0107,-0.0108,-0.01059,-0.01004],"A1126":[0.01043,0.00695,0.00355,0.00026,-0.00282,-0.00564,-0.00818,-0.01041,-0.01228,-0.01382,-0.01498,-0.01576,-0.01615,-0.01614,-0.01572,-0.01488,-0.01361,-0.01193,-0.00984,-0.00737,-0.00458,-0.00149,0.00181,0.00523,0.00873,0.01222,0.01562,0.01888,0.02191,0.02469,0.02715,0.02927,0.03105,0.03247,0.03353,0.03424,0.03463,0.03475,0.03462,0.0343,0.03384,0.03333,0.03279,0.03229,0.03188,0.0316,0.03146,0.03149,0.03167,0.03202,0.03249,0.03309,0.03376,0.03446,0.03516,0.0358,0.03637,0.03679,0.03704,0.03707,0.03685,0.03635,0.03554,0.0344,0.03292,0.03109,0.02891,0.02642,0.02363,0.02057,0.01731,0.01391],"A1127":[0.00176,-0.00136,-0.00463,-0.00797,-0.01124,-0.0144,-0.01734,-0.01998,-0.02229,-0.02423,-0.02577,-0.02693,-0.02771,-0.02815,-0.02826,-0.02809,-0.02767,-0.02702,-0.02615,-0.02508,-0.02381,-0.02234,-0.02067,-0.01878,-0.01666,-0.01432,-0.01175,-0.00895,-0.00596,-0.00282,0.00043,0.00369,0.00692,0.01006,0.013,0.0157,0.0181,0.02018,0.02191,0.02332,0.0244,0.02518,0.0257,0.02598,0.02604,0.02591,0.02559,0.0251,0.02445,0.02366,0.02276,0.02176,0.02072,0.01967,0.01867,0.01776,0.01697,0.01634,0.01587,0.01556,0.01539,0.01529,0.01523,0.01512,0.0149,0.01446,0.01376,0.0127,0.01127,0.00944,0.00722,0.00464],"A1128":[-0.03249,-0.03274,-0.03277,-0.03258,-0.03219,-0.0316,-0.03086,-0.03,-0.02907,-0.02814,-0.02723,-0.02641,-0.02569,-0.02511,-0.02464,-0.02428,-0.02397,-0.02368,-0.02334,-0.0229,-0.02232,-0.02152,-0.0205,-0.01922,-0.01765,-0.01582,-0.01374,-0.01142,-0.00893,-0.00635,-0.00373,-0.00121,0.00116,0.00326,0.005,0.00631,0.00716,0.00752,0.0074,0.00684,0.00592,0.00473,0.00335,0.00187,0.00038,-0.00106,-0.00239,-0.00361,-0.00467,-0.00565,-0.00652,-0.00734,-0.00815,-0.009,-0.00995,-0.01096,-0.01211,-0.01339,-0.0148,-0.0163,-0.01788,-0.01951,-0.02115,-0.02277,-0.02433,-0.02582,-0.02721,-0.02847,-0.0296,-0.03059,-0.0314,-0.03204],"A1129":[0.01393,0.01771,0.02158,0.02545,0.02913,0.03254,0.03555,0.03796,0.03969,0.04064,0.04076,0.04,0.0384,0.03597,0.03282,0.02904,0.02478,0.02017,0.01535,0.01049,0.00566,0.001,-0.0034,-0.00748,-0.01119,-0.01447,-0.0173,-0.01968,-0.02161,-0.02312,-0.02423,-0.02499,-0.02548,-0.02576,-0.0259,-0.026,-0.02612,-0.02634,-0.0267,-0.02721,-0.02792,-0.02876,-0.02973,-0.03076,-0.03177,-0.03272,-0.03351,-0.03409,-0.03438,-0.03437,-0.034,-0.0333,-0.03226,-0.0309,-0.0293,-0.02746,-0.0255,-0.02341,-0.02128,-0.01915,-0.01703,-0.01495,-0.01289,-0.01084,-0.00876,-0.00661,-0.00433,-0.00189,0.00078,0.00369,0.00685,0.01028],"A1130":[0.00006,0.0007,0.00136,0.00201,0.00263,0.0032,0.00369,0.00408,0.00434,0.00447,0.00445,0.00429,0.004,0.00359,0.0031,0.00254,0.00197,0.00143,0.00097,0.00062,0.00043,0.00044,0.00068,0.00116,0.00192,0.00293,0.00422,0.00575,0.00748,0.00937,0.01136,0.01334,0.01525,0.017,0.01848,0.01963,0.0204,0.02076,0.02071,0.02029,0.01956,0.01861,0.01751,0.01635,0.01521,0.01413,0.01316,0.01229,0.01151,0.0108,0.0101,0.0094,0.00864,0.00781,0.0069,0.00592,0.00487,0.00378,0.00269,0.00165,0.00067,-0.00019,-0.00092,-0.00149,-0.00189,-0.00213,-0.0022,-0.00211,-0.00188,-0.00153,-0.00108,-0.00054],"A1131":[0.05786,0.0544,0.05014,0.04509,0.03935,0.03297,0.02607,0.01878,0.01126,0.00366,-0.00384,-0.01103,-0.01778,-0.02388,-0.02921,-0.03368,-0.03714,-0.03964,-0.0411,-0.04159,-0.04116,-0.03994,-0.03802,-0.03557,-0.03268,-0.02952,-0.02618,-0.02278,-0.01939,-0.01608,-0.01288,-0.00979,-0.00683,-0.00398,-0.00124,0.00143,0.00404,0.00659,0.00909,0.01156,0.01397,0.01632,0.01861,0.02081,0.02291,0.02493,0.02683,0.02864,0.03038,0.03205,0.03369,0.03533,0.03698,0.03868,0.04046,0.04231,0.04425,0.04629,0.04839,0.05055,0.05272,0.05488,0.05695,0.0589,0.06065,0.06212,0.06324,0.0639,0.06405,0.06358,0.06243,0.06054],"A1132":[0.0243,0.02622,0.02786,0.02918,0.03014,0.03071,0.03087,0.03063,0.03003,0.02908,0.02786,0.02645,0.0249,0.02337,0.02189,0.02061,0.01957,0.01888,0.01857,0.01867,0.01921,0.02017,0.02155,0.02329,0.02535,0.02769,0.03027,0.03302,0.03585,0.03874,0.04161,0.04438,0.04696,0.04932,0.05133,0.0529,0.05396,0.05442,0.05419,0.05324,0.05155,0.04913,0.04601,0.04229,0.03807,0.03348,0.0287,0.02386,0.01913,0.01468,0.01061,0.00701,0.00398,0.00153,-0.00031,-0.00157,-0.00227,-0.0025,-0.00225,-0.00163,-0.00066,0.00061,0.00212,0.00388,0.00583,0.00795,0.01021,0.01259,0.01501,0.01746,0.01986,0.02215],"A1133":[0.03257,0.03402,0.03495,0.03539,0.03531,0.03471,0.03366,0.03216,0.0303,0.02809,0.02564,0.02301,0.02028,0.01753,0.0148,0.01221,0.00978,0.00759,0.00566,0.00403,0.00274,0.00177,0.00115,0.00086,0.00089,0.00123,0.00184,0.00269,0.00372,0.00489,0.00613,0.00734,0.00848,0.00944,0.01016,0.01058,0.01065,0.01034,0.00967,0.00861,0.00723,0.00557,0.00369,0.00165,-0.00049,-0.00265,-0.00476,-0.00678,-0.00863,-0.01026,-0.01164,-0.01272,-0.01345,-0.0138,-0.01374,-0.01326,-0.01234,-0.01098,-0.0092,-0.00701,-0.00445,-0.00156,0.00159,0.00498,0.0085,0.01208,0.01567,0.01915,0.02246,0.02553,0.02827,0.03064],"A1134":[0.02164,0.02098,0.01981,0.01814,0.016,0.01344,0.0105,0.00729,0.00385,0.00025,-0.0034,-0.00706,-0.01063,-0.01404,-0.01725,-0.0202,-0.02282,-0.02508,-0.02693,-0.02834,-0.02928,-0.02973,-0.02968,-0.02914,-0.02811,-0.02662,-0.02471,-0.02241,-0.01981,-0.01698,-0.01399,-0.01097,-0.00797,-0.00512,-0.00251,-0.00023,0.00166,0.00311,0.00409,0.0046,0.00466,0.00431,0.00361,0.00262,0.00144,0.00013,-0.00121,-0.00251,-0.0037,-0.0047,-0.00547,-0.00597,-0.00616,-0.00603,-0.00557,-0.00479,-0.0037,-0.00232,-0.00069,0.00115,0.00318,0.00534,0.00756,0.00983,0.01206,0.0142,0.0162,0.01799,0.0195,0.02068,0.02146,0.02179],"A1135":[0.01394,0.01334,0.0126,0.01171,0.0106,0.00929,0.00776,0.00604,0.00414,0.0021,-0.00001,-0.00218,-0.00433,-0.00644,-0.00844,-0.01033,-0.01202,-0.0135,-0.01475,-0.01574,-0.01644,-0.01684,-0.0169,-0.01661,-0.01594,-0.01487,-0.01338,-0.01144,-0.00905,-0.00624,-0.00302,0.00053,0.00434,0.00832,0.01234,0.01627,0.02003,0.02344,0.02648,0.0291,0.03125,0.03297,0.03429,0.03528,0.03602,0.03658,0.03702,0.0374,0.03774,0.03803,0.03828,0.03843,0.03845,0.03827,0.03785,0.03716,0.03618,0.03489,0.03333,0.03154,0.02958,0.02754,0.02548,0.02349,0.02165,0.02,0.01858,0.0174,0.01644,0.01568,0.01505,0.0145],"A1136":[0.02979,0.03591,0.04136,0.04601,0.04968,0.05227,0.05374,0.05404,0.05322,0.05137,0.04863,0.04514,0.04113,0.03683,0.03244,0.02818,0.02426,0.02084,0.01807,0.01606,0.01486,0.01448,0.01492,0.01611,0.01798,0.02039,0.02323,0.02635,0.02957,0.03269,0.03557,0.038,0.03984,0.04093,0.04114,0.04042,0.03869,0.03601,0.0324,0.028,0.02292,0.01739,0.01154,0.00561,-0.00024,-0.00587,-0.01113,-0.01594,-0.02023,-0.02397,-0.02718,-0.02986,-0.03203,-0.03374,-0.035,-0.03583,-0.03623,-0.0362,-0.0357,-0.03468,-0.03312,-0.03094,-0.02811,-0.0246,-0.02039,-0.01549,-0.00996,-0.0039,0.00261,0.00943,0.01635,0.02319],"A1137":[0.04059,0.03915,0.03754,0.03582,0.03408,0.03237,0.03073,0.02919,0.02774,0.02637,0.02503,0.02368,0.02228,0.02079,0.01916,0.01741,0.01553,0.01357,0.01154,0.00953,0.00761,0.00585,0.00433,0.00311,0.00225,0.00182,0.00184,0.00234,0.00327,0.00463,0.00637,0.0084,0.01064,0.01302,0.01541,0.01773,0.01992,0.02188,0.02357,0.02497,0.02604,0.02681,0.02728,0.02748,0.02744,0.02722,0.02686,0.02642,0.02594,0.02549,0.02511,0.02487,0.02481,0.02496,0.02537,0.02605,0.027,0.02821,0.02965,0.03128,0.03305,0.03489,0.0367,0.03843,0.04,0.04133,0.04235,0.04302,0.0433,0.04318,0.04266,0.04178],"A1138":[0.01474,0.01391,0.01294,0.01187,0.01072,0.00954,0.00836,0.0072,0.0061,0.00507,0.00415,0.00335,0.00267,0.00213,0.00172,0.00145,0.00132,0.00133,0.00147,0.00174,0.00213,0.00262,0.00322,0.0039,0.00465,0.00546,0.00631,0.00719,0.00806,0.00891,0.00972,0.01045,0.0111,0.01165,0.01207,0.01237,0.01255,0.01261,0.01255,0.01242,0.01221,0.01196,0.01168,0.01141,0.01115,0.01092,0.01072,0.01057,0.01045,0.01038,0.01034,0.01035,0.0104,0.01051,0.01067,0.01089,0.01119,0.01156,0.012,0.01251,0.01307,0.01366,0.01426,0.01485,0.01538,0.01583,0.01617,0.01636,0.0164,0.01625,0.01593,0.01542],"A1139":[0.00764,0.00776,0.00787,0.00799,0.0081,0.00821,0.00832,0.00841,0.00849,0.00854,0.00857,0.00858,0.00855,0.00849,0.0084,0.00828,0.00814,0.00797,0.00778,0.00759,0.0074,0.00722,0.00708,0.00699,0.00696,0.00702,0.00719,0.00747,0.00787,0.00838,0.00899,0.00967,0.01039,0.01111,0.01177,0.01232,0.01273,0.01295,0.01294,0.01269,0.0122,0.01147,0.01053,0.00942,0.00819,0.00687,0.00554,0.00426,0.00305,0.00199,0.0011,0.00042,-0.00004,-0.00028,-0.0003,-0.00011,0.00024,0.00075,0.00136,0.00205,0.00277,0.0035,0.00419,0.00484,0.00542,0.00593,0.00635,0.0067,0.00698,0.0072,0.00737,0.00752],"A1140":[0.0248,0.02555,0.02602,0.02618,0.02597,0.02539,0.02441,0.02303,0.02128,0.01913,0.01667,0.01393,0.01097,0.00789,0.00475,0.00162,-0.00136,-0.00417,-0.00671,-0.00892,-0.01074,-0.01217,-0.01317,-0.01374,-0.01389,-0.01366,-0.0131,-0.01223,-0.01114,-0.00988,-0.00851,-0.00713,-0.00577,-0.00449,-0.00334,-0.00235,-0.00154,-0.00093,-0.0005,-0.00024,-0.00012,-0.00013,-0.00022,-0.00036,-0.00053,-0.00069,-0.00081,-0.00088,-0.00086,-0.00076,-0.00054,-0.0002,0.00026,0.00085,0.00157,0.0024,0.00335,0.00441,0.00555,0.00678,0.00809,0.00946,0.01087,0.01234,0.01384,0.01537,0.0169,0.01842,0.01991,0.02133,0.02265,0.02382],"A1141":[0.01665,0.01623,0.01561,0.0148,0.01382,0.01267,0.01139,0.01001,0.00855,0.00704,0.00553,0.00407,0.00269,0.00143,0.00032,-0.00059,-0.00129,-0.00176,-0.00196,-0.00191,-0.00161,-0.00106,-0.00028,0.00072,0.00189,0.00322,0.00465,0.00616,0.00768,0.00917,0.01059,0.01188,0.01301,0.01393,0.01463,0.01507,0.01526,0.0152,0.0149,0.0144,0.01372,0.01291,0.01199,0.01102,0.01005,0.0091,0.00821,0.00741,0.00673,0.00619,0.00579,0.00556,0.00549,0.00558,0.00584,0.00624,0.00678,0.00745,0.00822,0.00908,0.00999,0.01094,0.0119,0.01284,0.01375,0.01458,0.01532,0.01594,0.01643,0.01676,0.01691,0.01688],"B1021":[0.04079,0.03879,0.03702,0.03548,0.03417,0.03303,0.03198,0.03096,0.02986,0.0286,0.02709,0.02527,0.02311,0.02062,0.0178,0.01471,0.01146,0.00813,0.00485,0.00176,-0.00102,-0.00339,-0.0052,-0.00641,-0.00696,-0.00685,-0.00611,-0.00481,-0.00305,-0.00097,0.0013,0.00358,0.00578,0.00774,0.0094,0.01071,0.01166,0.0123,0.0127,0.01295,0.01316,0.01344,0.01389,0.01459,0.01561,0.01697,0.01868,0.02075,0.02311,0.02575,0.02858,0.03159,0.03469,0.0378,0.04089,0.04387,0.0467,0.0493,0.05159,0.05352,0.05504,0.05608,0.05661,0.05664,0.05613,0.05514,0.05371,0.05192,0.04985,0.04758,0.04526,0.04297],"B1022":[0.0115,0.00942,0.00741,0.00544,0.00354,0.00171,-0.00006,-0.00175,-0.00338,-0.00495,-0.00646,-0.00792,-0.00933,-0.01069,-0.012,-0.01324,-0.01438,-0.01543,-0.01633,-0.01705,-0.01756,-0.01781,-0.01778,-0.01742,-0.01671,-0.01564,-0.01417,-0.0123,-0.01007,-0.0075,-0.00463,-0.00151,0.0018,0.0052,0.00863,0.012,0.01525,0.0183,0.02111,0.02366,0.02592,0.02791,0.02964,0.03112,0.0324,0.03351,0.03448,0.03533,0.03609,0.03675,0.03732,0.0378,0.03816,0.0384,0.0385,0.03842,0.03815,0.03768,0.037,0.03608,0.03496,0.03362,0.03209,0.03038,0.02852,0.02654,0.02447,0.02233,0.02016,0.01797,0.01578,0.01363],"B1023":[0.04019,0.03978,0.03915,0.03824,0.03707,0.03563,0.03393,0.03202,0.02991,0.02762,0.02522,0.02273,0.02019,0.01764,0.0151,0.01259,0.01016,0.00778,0.00549,0.0033,0.00121,-0.00079,-0.00265,-0.00434,-0.00586,-0.00713,-0.00809,-0.00869,-0.00888,-0.00858,-0.00775,-0.0064,-0.00452,-0.00215,0.00062,0.0037,0.00697,0.01027,0.01351,0.01655,0.01928,0.02166,0.02363,0.0252,0.02637,0.02722,0.02779,0.02816,0.02841,0.02861,0.02882,0.02908,0.02943,0.02989,0.03046,0.03113,0.03188,0.03269,0.03353,0.03436,0.03518,0.03596,0.03668,0.03736,0.03799,0.03856,0.03908,0.03954,0.03992,0.0402,0.04036,0.04037],"B1201":[0.02524,0.02261,0.01999,0.01743,0.01501,0.01273,0.01067,0.00887,0.00736,0.00617,0.00533,0.00484,0.0047,0.00491,0.00542,0.00623,0.00727,0.00849,0.00985,0.01128,0.01273,0.01417,0.01556,0.01685,0.01804,0.0191,0.02003,0.02084,0.0215,0.02204,0.02244,0.02269,0.02279,0.02275,0.02256,0.02223,0.02179,0.02129,0.02076,0.02027,0.01989,0.01969,0.01973,0.02006,0.02072,0.02173,0.02308,0.02473,0.02665,0.02876,0.031,0.0333,0.03557,0.03772,0.03971,0.04146,0.04293,0.04408,0.04488,0.04533,0.04543,0.04518,0.04459,0.04369,0.04249,0.04101,0.03929,0.03734,0.03519,0.03286,0.03041,0.02786],"B1202":[0.02142,0.02165,0.0219,0.0222,0.02255,0.02298,0.02347,0.02399,0.02453,0.02505,0.02549,0.02583,0.02601,0.02602,0.02585,0.02548,0.02493,0.02421,0.02334,0.02236,0.02131,0.0202,0.01911,0.01808,0.01714,0.01636,0.0158,0.01549,0.01546,0.01574,0.01631,0.01715,0.01821,0.01942,0.02069,0.02194,0.02308,0.02403,0.02472,0.02513,0.02524,0.02508,0.02466,0.02403,0.02326,0.02239,0.02148,0.02058,0.01971,0.01891,0.01819,0.01756,0.01702,0.01659,0.01624,0.016,0.01586,0.01583,0.01589,0.01606,0.01633,0.01669,0.01712,0.01762,0.01816,0.01871,0.01924,0.01974,0.02019,0.02058,0.0209,0.02118],"B1203":[0.00821,0.00983,0.01136,0.01275,0.01396,0.01497,0.01577,0.01634,0.01667,0.01679,0.01669,0.01641,0.01596,0.01537,0.01469,0.01394,0.01316,0.01238,0.01165,0.01098,0.01043,0.01,0.00974,0.00966,0.00979,0.01014,0.01075,0.01161,0.01272,0.01407,0.01564,0.01738,0.01925,0.02119,0.02313,0.02499,0.0267,0.02818,0.02938,0.03027,0.0308,0.03095,0.03073,0.03015,0.02923,0.02799,0.02648,0.02473,0.02278,0.0207,0.01849,0.01619,0.01388,0.01159,0.00934,0.00718,0.00516,0.00331,0.00167,0.00028,-0.00083,-0.00163,-0.0021,-0.00224,-0.00203,-0.0015,-0.00067,0.00042,0.00175,0.00325,0.00486,0.00654],"B1204":[0.02477,0.02173,0.01862,0.01548,0.01241,0.00945,0.00664,0.00405,0.0017,-0.0004,-0.00223,-0.00377,-0.00502,-0.00593,-0.00654,-0.00679,-0.00668,-0.0062,-0.00534,-0.00411,-0.00253,-0.00063,0.00153,0.00391,0.00642,0.00896,0.01147,0.01388,0.01606,0.01799,0.01959,0.02084,0.02172,0.02226,0.02246,0.02239,0.0221,0.02166,0.02116,0.02066,0.02023,0.01995,0.01984,0.01997,0.02035,0.02099,0.02186,0.02297,0.02428,0.02574,0.02731,0.02897,0.03064,0.03229,0.0339,0.0354,0.03678,0.03801,0.03906,0.03991,0.04053,0.0409,0.041,0.04082,0.04032,0.0395,0.03834,0.03684,0.03501,0.03284,0.03039,0.02769],"B1205":[0.00571,0.0027,-0.00026,-0.00314,-0.00588,-0.00844,-0.01082,-0.01296,-0.01486,-0.01651,-0.01789,-0.01899,-0.01977,-0.02021,-0.02032,-0.02004,-0.01937,-0.0183,-0.01681,-0.01493,-0.01266,-0.01005,-0.00713,-0.00397,-0.00063,0.00279,0.00624,0.00962,0.01285,0.01586,0.01857,0.02094,0.02293,0.02452,0.02572,0.02653,0.02699,0.02714,0.02704,0.02673,0.02629,0.02574,0.02516,0.02457,0.02402,0.02355,0.02318,0.02293,0.02283,0.02289,0.02312,0.02349,0.02401,0.02465,0.02536,0.02609,0.02679,0.02741,0.02788,0.02816,0.02819,0.02794,0.02737,0.02647,0.02523,0.02366,0.02177,0.0196,0.01716,0.01449,0.01166,0.00872],"B1206":[0.02883,0.02403,0.01894,0.0136,0.00814,0.00261,-0.0029,-0.00831,-0.01357,-0.01866,-0.02345,-0.02797,-0.03216,-0.03598,-0.03943,-0.04249,-0.0451,-0.04726,-0.04894,-0.0501,-0.05073,-0.0508,-0.05027,-0.04913,-0.04736,-0.04497,-0.04198,-0.03842,-0.03438,-0.02995,-0.02522,-0.02039,-0.01556,-0.01086,-0.00644,-0.00237,0.00129,0.00449,0.00727,0.0097,0.0118,0.0137,0.01547,0.0172,0.01895,0.02081,0.02276,0.02486,0.02708,0.0294,0.03181,0.03427,0.03673,0.03916,0.04151,0.04374,0.04582,0.04771,0.04935,0.05071,0.05174,0.0524,0.05266,0.05246,0.0518,0.05066,0.049,0.04684,0.04418,0.04101,0.03739,0.03332],"B1207":[0.06105,0.06323,0.06518,0.06692,0.06845,0.06977,0.07089,0.07177,0.07242,0.07278,0.07283,0.07255,0.07192,0.07093,0.06958,0.06788,0.06588,0.06359,0.06106,0.05832,0.05539,0.05232,0.04914,0.04589,0.04257,0.03924,0.0359,0.0326,0.02932,0.02611,0.02297,0.01997,0.01707,0.01433,0.01178,0.00946,0.00739,0.00564,0.00424,0.0032,0.00258,0.00237,0.00256,0.00308,0.00393,0.00504,0.0063,0.00765,0.00904,0.0104,0.01171,0.01296,0.01413,0.01527,0.01642,0.0176,0.0189,0.02036,0.02202,0.02393,0.0261,0.02854,0.03123,0.03415,0.03724,0.04045,0.04372,0.04696,0.05012,0.05315,0.056,0.05864],"B1208":[0.01132,0.0088,0.00605,0.00311,0.00001,-0.00322,-0.00655,-0.00986,-0.01315,-0.01635,-0.01937,-0.02217,-0.0247,-0.02691,-0.02877,-0.03025,-0.03134,-0.03203,-0.03234,-0.03227,-0.03185,-0.03109,-0.03002,-0.02867,-0.02704,-0.0252,-0.02313,-0.02086,-0.01844,-0.01587,-0.01322,-0.01053,-0.00782,-0.00517,-0.0026,-0.00018,0.00208,0.00415,0.00604,0.00776,0.00931,0.01074,0.01207,0.01334,0.01456,0.01576,0.01695,0.01813,0.01929,0.02041,0.02149,0.0225,0.02341,0.02423,0.02492,0.02549,0.02591,0.02619,0.02632,0.0263,0.02613,0.02581,0.02535,0.02473,0.02397,0.02304,0.02195,0.02069,0.01924,0.01759,0.01572,0.01365],"B1209":[0.0122,0.01417,0.01628,0.01851,0.02075,0.02295,0.02506,0.02698,0.02869,0.03014,0.03131,0.03221,0.03282,0.03318,0.03332,0.03326,0.03304,0.0327,0.03227,0.03178,0.03125,0.03069,0.03014,0.02959,0.02904,0.02851,0.02797,0.02742,0.02683,0.02619,0.02545,0.02458,0.02353,0.02228,0.0208,0.01907,0.01708,0.01484,0.01241,0.00978,0.00705,0.00429,0.00155,-0.00106,-0.00347,-0.00562,-0.00745,-0.00891,-0.00997,-0.01062,-0.01087,-0.01073,-0.01025,-0.00946,-0.00843,-0.00723,-0.00591,-0.00454,-0.00318,-0.00188,-0.00067,0.00043,0.0014,0.00228,0.00307,0.00382,0.0046,0.00544,0.0064,0.00754,0.00888,0.01043],"B1210":[-0.00085,-0.0019,-0.00247,-0.00258,-0.00224,-0.0015,-0.00044,0.00086,0.00231,0.00381,0.00527,0.00662,0.00778,0.00869,0.00935,0.00973,0.00981,0.00962,0.00915,0.00844,0.0075,0.00635,0.00502,0.00355,0.00197,0.00032,-0.00138,-0.00306,-0.0047,-0.00626,-0.0077,-0.00898,-0.0101,-0.01102,-0.01174,-0.01222,-0.01246,-0.01244,-0.01214,-0.01154,-0.01062,-0.00937,-0.00776,-0.00582,-0.00354,-0.00095,0.0019,0.00496,0.00816,0.01138,0.01458,0.01765,0.02046,0.02298,0.0251,0.02675,0.0279,0.0285,0.02855,0.02805,0.02703,0.02552,0.02358,0.02127,0.01869,0.01592,0.01305,0.0102,0.00745,0.00487,0.00259,0.00067],"B1211":[-0.01329,-0.01526,-0.01678,-0.01789,-0.01858,-0.01889,-0.01888,-0.01858,-0.01804,-0.01733,-0.01647,-0.01551,-0.01447,-0.01338,-0.01226,-0.0111,-0.00994,-0.00879,-0.00765,-0.00654,-0.00547,-0.00445,-0.00349,-0.00262,-0.00182,-0.00112,-0.00052,-0.00004,0.00032,0.00053,0.00059,0.00046,0.00013,-0.0004,-0.00114,-0.00207,-0.00317,-0.00439,-0.00565,-0.00689,-0.00802,-0.00894,-0.00955,-0.00979,-0.00955,-0.00884,-0.00761,-0.00588,-0.00369,-0.00113,0.00172,0.00477,0.00784,0.01082,0.01357,0.01595,0.01787,0.01923,0.01996,0.02004,0.01944,0.0182,0.01638,0.01403,0.01126,0.00817,0.00487,0.00147,-0.0019,-0.00516,-0.00819,-0.01092],"B1212":[0.03657,0.03754,0.03795,0.03776,0.03693,0.03549,0.03343,0.03083,0.02773,0.02424,0.02044,0.01646,0.01239,0.00836,0.00445,0.00078,-0.00254,-0.00549,-0.00798,-0.00997,-0.01141,-0.01233,-0.0127,-0.01255,-0.01191,-0.01083,-0.00935,-0.00755,-0.00549,-0.00327,-0.00097,0.00131,0.00349,0.00547,0.00716,0.00852,0.00947,0.00998,0.01006,0.00971,0.00897,0.00789,0.00656,0.00505,0.00347,0.00188,0.00037,-0.00098,-0.00213,-0.003,-0.00359,-0.00387,-0.00383,-0.00348,-0.00282,-0.00186,-0.00062,0.00089,0.00263,0.0046,0.00679,0.00917,0.0117,0.01438,0.01715,0.02,0.02287,0.02568,0.02838,0.03092,0.03318,0.03508],"B1213":[0.02284,0.02431,0.02534,0.02592,0.02607,0.02581,0.02517,0.02421,0.02299,0.02158,0.02004,0.01842,0.0168,0.01523,0.01374,0.01239,0.0112,0.01022,0.00944,0.00891,0.00863,0.00859,0.00881,0.00928,0.00997,0.01087,0.01196,0.01321,0.01457,0.016,0.01744,0.01884,0.02013,0.02126,0.02216,0.02276,0.02304,0.02295,0.02249,0.02165,0.02045,0.01891,0.01708,0.01503,0.0128,0.01044,0.00804,0.00564,0.0033,0.00108,-0.00099,-0.00285,-0.00448,-0.00583,-0.00689,-0.00761,-0.00799,-0.008,-0.00763,-0.00686,-0.00571,-0.00419,-0.00233,-0.00013,0.00234,0.00501,0.00783,0.01068,0.01352,0.01624,0.01874,0.02097],"B1214":[-0.00264,-0.00383,-0.00467,-0.00518,-0.00535,-0.00522,-0.00481,-0.00417,-0.00333,-0.00232,-0.00117,0.0001,0.00148,0.00295,0.0045,0.00612,0.00776,0.00939,0.01096,0.01239,0.01362,0.01459,0.01521,0.01542,0.01518,0.01445,0.01322,0.0115,0.00932,0.00674,0.00381,0.00064,-0.00268,-0.00606,-0.00934,-0.01243,-0.01524,-0.01763,-0.01953,-0.02087,-0.02158,-0.02164,-0.02104,-0.01979,-0.01792,-0.0155,-0.01257,-0.00928,-0.00568,-0.00195,0.00185,0.00556,0.00907,0.01227,0.01506,0.01738,0.01915,0.02036,0.02098,0.02104,0.02056,0.01958,0.01818,0.01641,0.01436,0.01211,0.00974,0.00735,0.00499,0.00274,0.00068,-0.00112],"B1215":[0.02205,0.01987,0.01712,0.01383,0.01005,0.00588,0.00141,-0.00321,-0.00788,-0.01248,-0.01687,-0.02096,-0.02466,-0.02789,-0.03061,-0.03279,-0.03438,-0.03541,-0.03584,-0.0357,-0.03499,-0.03373,-0.03192,-0.0296,-0.02677,-0.0235,-0.0198,-0.01573,-0.0114,-0.00691,-0.00234,0.00213,0.00641,0.01038,0.01388,0.01686,0.01922,0.02093,0.02198,0.0224,0.02223,0.02156,0.02045,0.01902,0.01736,0.01557,0.01375,0.01199,0.01036,0.00894,0.0078,0.00697,0.00649,0.00638,0.00664,0.00726,0.00821,0.00946,0.01093,0.0126,0.01438,0.01622,0.01804,0.01979,0.0214,0.02281,0.02396,0.02477,0.02521,0.0252,0.02469,0.02366],"B1216":[-0.0203,-0.01844,-0.01628,-0.01391,-0.01142,-0.0089,-0.00638,-0.00398,-0.00172,0.00037,0.00229,0.00401,0.00558,0.00696,0.0082,0.00931,0.0103,0.01118,0.01195,0.01262,0.01317,0.01362,0.01394,0.01416,0.01426,0.01423,0.01408,0.01379,0.01337,0.01279,0.01206,0.01115,0.01005,0.00875,0.00725,0.00554,0.00364,0.00157,-0.00063,-0.00291,-0.00522,-0.00749,-0.00965,-0.01162,-0.01336,-0.01481,-0.01595,-0.01675,-0.01723,-0.01741,-0.01733,-0.01706,-0.01668,-0.01624,-0.01584,-0.01554,-0.0154,-0.01548,-0.01579,-0.01634,-0.01711,-0.01807,-0.01916,-0.02031,-0.02142,-0.02242,-0.02321,-0.02371,-0.02386,-0.02361,-0.02293,-0.02181],"B1217":[-0.00466,-0.00438,-0.00394,-0.00335,-0.0026,-0.00171,-0.0007,0.00041,0.0016,0.00284,0.0041,0.00535,0.00655,0.00768,0.00872,0.00964,0.01041,0.01104,0.01149,0.01178,0.0119,0.01185,0.01163,0.01127,0.01078,0.01015,0.00942,0.00859,0.00768,0.00672,0.0057,0.00467,0.00363,0.00261,0.00164,0.00073,-0.0001,-0.00082,-0.00142,-0.00189,-0.00224,-0.00246,-0.00255,-0.00254,-0.00245,-0.00228,-0.00207,-0.00182,-0.00156,-0.00131,-0.00109,-0.00089,-0.00074,-0.00063,-0.00059,-0.0006,-0.00066,-0.00079,-0.00097,-0.00121,-0.0015,-0.00183,-0.0022,-0.0026,-0.00301,-0.00342,-0.00381,-0.00417,-0.00446,-0.00468,-0.0048,-0.0048],"B1218":[-0.00248,-0.00169,-0.00092,-0.00019,0.00049,0.00109,0.00161,0.00203,0.00236,0.0026,0.00276,0.00285,0.00288,0.00286,0.00282,0.00276,0.00272,0.0027,0.00274,0.00285,0.00306,0.00337,0.00381,0.00438,0.0051,0.00595,0.00693,0.00804,0.00925,0.01054,0.01187,0.01319,0.01446,0.01564,0.01664,0.01746,0.01803,0.01833,0.01834,0.01805,0.01749,0.01667,0.01562,0.01439,0.01301,0.01153,0.00999,0.00843,0.00689,0.00538,0.00395,0.00258,0.0013,0.00012,-0.00097,-0.00196,-0.00286,-0.00366,-0.00436,-0.00497,-0.00548,-0.00589,-0.00617,-0.00634,-0.00638,-0.0063,-0.00607,-0.00572,-0.00525,-0.00467,-0.004,-0.00326],"B1219":[0.00442,0.0058,0.00719,0.00855,0.00986,0.01111,0.01225,0.01326,0.01414,0.01487,0.01544,0.01586,0.01614,0.0163,0.01638,0.0164,0.01639,0.0164,0.01646,0.0166,0.01684,0.01719,0.01768,0.0183,0.01906,0.01993,0.02092,0.02198,0.02309,0.02423,0.02533,0.02635,0.02723,0.02794,0.02839,0.02856,0.02838,0.02784,0.02693,0.02562,0.02396,0.02198,0.01973,0.01727,0.01468,0.01204,0.00941,0.00689,0.00452,0.00237,0.00046,-0.00119,-0.00255,-0.00365,-0.0045,-0.00513,-0.00555,-0.00581,-0.00592,-0.0059,-0.00576,-0.00551,-0.00516,-0.0047,-0.00412,-0.00343,-0.00261,-0.00168,-0.00064,0.00051,0.00175,0.00306],"B1220":[0.00756,0.00525,0.00297,0.00075,-0.00131,-0.00318,-0.0048,-0.00613,-0.00714,-0.00781,-0.00814,-0.00814,-0.00784,-0.00727,-0.00648,-0.00553,-0.00445,-0.00331,-0.00215,-0.001,0.00009,0.00113,0.0021,0.00299,0.00383,0.00462,0.0054,0.00617,0.00696,0.00779,0.00864,0.00951,0.01041,0.0113,0.01218,0.01301,0.01381,0.01456,0.01526,0.01594,0.0166,0.01727,0.01797,0.01871,0.01952,0.02038,0.02129,0.02224,0.02319,0.02413,0.02501,0.02583,0.02654,0.02712,0.02755,0.02783,0.02795,0.02789,0.02767,0.02728,0.02671,0.02599,0.02511,0.02404,0.02282,0.02143,0.01987,0.01815,0.01628,0.01425,0.01211,0.00987],"B1221":[0.00812,0.00996,0.01193,0.014,0.01612,0.01824,0.02032,0.02228,0.02408,0.02567,0.02699,0.02803,0.02875,0.02915,0.02924,0.02902,0.02852,0.02775,0.02674,0.02553,0.02414,0.0226,0.02096,0.01923,0.01745,0.01568,0.01394,0.01227,0.0107,0.00928,0.00798,0.00685,0.00585,0.00498,0.00421,0.00347,0.00276,0.00203,0.00125,0.0004,-0.0005,-0.00146,-0.00247,-0.00348,-0.00446,-0.0054,-0.00626,-0.007,-0.00762,-0.0081,-0.00845,-0.00866,-0.00873,-0.00866,-0.00848,-0.00819,-0.00778,-0.0073,-0.00673,-0.00609,-0.00538,-0.00462,-0.00382,-0.00296,-0.00206,-0.00111,-0.0001,0.00098,0.00215,0.00345,0.00486,0.00641],"B1222":[0.01851,0.01594,0.01332,0.01075,0.00828,0.006,0.00391,0.00205,0.0004,-0.00107,-0.00238,-0.00356,-0.00469,-0.00576,-0.00683,-0.0079,-0.009,-0.01011,-0.01123,-0.0123,-0.01331,-0.01421,-0.01495,-0.01547,-0.01574,-0.01569,-0.0153,-0.01456,-0.01345,-0.012,-0.01023,-0.00823,-0.00604,-0.00374,-0.00145,0.00079,0.00289,0.0048,0.00647,0.00791,0.0091,0.01007,0.01084,0.01146,0.01196,0.01238,0.01277,0.01317,0.0136,0.01409,0.01467,0.01536,0.01617,0.01711,0.01818,0.01936,0.02064,0.022,0.02339,0.02476,0.02605,0.02721,0.02816,0.02886,0.02923,0.02923,0.02884,0.02803,0.02681,0.02519,0.02323,0.02097],"B1223":[0.01449,0.01294,0.01147,0.01009,0.00884,0.00773,0.00676,0.00592,0.00519,0.00454,0.00395,0.0034,0.00287,0.00234,0.00181,0.00126,0.0007,0.00012,-0.00045,-0.00102,-0.00158,-0.00211,-0.00261,-0.00305,-0.00343,-0.00372,-0.00391,-0.00397,-0.0039,-0.00369,-0.00332,-0.00281,-0.00216,-0.0014,-0.00056,0.00034,0.00126,0.00216,0.00301,0.00381,0.00455,0.0052,0.00581,0.00637,0.00691,0.00748,0.00807,0.00874,0.00948,0.01032,0.01127,0.01232,0.01346,0.01466,0.01592,0.0172,0.01846,0.01968,0.0208,0.02179,0.02262,0.02324,0.02362,0.02375,0.02361,0.02319,0.0225,0.02157,0.02041,0.01907,0.0176,0.01606],"B1224":[0.04254,0.03629,0.02984,0.02323,0.01665,0.01018,0.00389,-0.00207,-0.00766,-0.01278,-0.01736,-0.02136,-0.02468,-0.02727,-0.02913,-0.03021,-0.03051,-0.03002,-0.02878,-0.02685,-0.02429,-0.02115,-0.01754,-0.01359,-0.00934,-0.00497,-0.00051,0.00391,0.00821,0.01234,0.01618,0.01972,0.02292,0.02577,0.0282,0.03025,0.03196,0.03331,0.03439,0.03526,0.03596,0.03659,0.03721,0.0379,0.03875,0.03979,0.04108,0.04264,0.04447,0.04657,0.04892,0.05149,0.05421,0.05704,0.05992,0.06273,0.06545,0.06798,0.07024,0.07215,0.07364,0.07463,0.07505,0.07485,0.07397,0.07239,0.07009,0.06707,0.06335,0.05894,0.05398,0.04848],"B1225":[0.01991,0.0153,0.01057,0.00585,0.00127,-0.0031,-0.0072,-0.01095,-0.01435,-0.01741,-0.02011,-0.02251,-0.02463,-0.02647,-0.02807,-0.02944,-0.03055,-0.03141,-0.03199,-0.03225,-0.03216,-0.03171,-0.03084,-0.02956,-0.02783,-0.02567,-0.0231,-0.0201,-0.01677,-0.01316,-0.00933,-0.0054,-0.00144,0.00248,0.00621,0.0097,0.01292,0.01582,0.01839,0.02068,0.02268,0.02447,0.02609,0.02759,0.02899,0.03034,0.03163,0.03288,0.03406,0.03517,0.03621,0.03717,0.03805,0.03884,0.03957,0.04024,0.04087,0.04146,0.04201,0.04249,0.04288,0.04313,0.04317,0.04293,0.04234,0.04132,0.0398,0.03777,0.03517,0.03201,0.02839,0.02432],"B1226":[-0.00667,-0.00718,-0.00764,-0.00803,-0.00839,-0.00873,-0.00907,-0.00945,-0.0099,-0.01045,-0.0111,-0.01187,-0.01273,-0.01369,-0.01468,-0.01568,-0.01656,-0.0173,-0.01779,-0.01797,-0.01776,-0.0171,-0.01595,-0.01428,-0.01209,-0.0094,-0.00626,-0.00277,0.001,0.00487,0.00873,0.01235,0.01562,0.01835,0.0204,0.02169,0.02215,0.02182,0.02073,0.01899,0.01675,0.0142,0.01151,0.00886,0.0064,0.00426,0.00254,0.00128,0.00048,0.00011,0.00013,0.00043,0.00094,0.00157,0.0022,0.00275,0.00316,0.00341,0.00341,0.00321,0.0028,0.00219,0.00145,0.0006,-0.00033,-0.00126,-0.0022,-0.0031,-0.00395,-0.00474,-0.00545,-0.0061],"B1227":[-0.01115,-0.00901,-0.00663,-0.00402,-0.00128,0.00151,0.00432,0.00699,0.00949,0.01173,0.01367,0.01525,0.01647,0.01731,0.01781,0.018,0.01792,0.01762,0.01717,0.01662,0.01599,0.01535,0.0147,0.01406,0.01344,0.01283,0.01222,0.01158,0.01089,0.01012,0.00924,0.00823,0.00703,0.00563,0.00402,0.00216,0.00006,-0.00225,-0.00476,-0.00744,-0.01023,-0.01304,-0.01583,-0.0185,-0.02097,-0.02321,-0.02512,-0.02666,-0.02782,-0.02856,-0.02892,-0.02891,-0.02857,-0.02796,-0.02713,-0.02617,-0.02514,-0.0241,-0.02309,-0.02218,-0.02137,-0.02068,-0.0201,-0.01962,-0.01918,-0.01874,-0.01824,-0.01764,-0.01685,-0.01584,-0.01457,-0.01301],"B1228":[0.01039,0.01034,0.01018,0.00992,0.00956,0.00912,0.00861,0.00804,0.00744,0.00682,0.0062,0.00561,0.00506,0.00458,0.00418,0.00388,0.0037,0.00363,0.0037,0.00389,0.00421,0.00465,0.00519,0.00583,0.00654,0.0073,0.00809,0.0089,0.00969,0.01044,0.01114,0.01175,0.01226,0.01263,0.01286,0.01292,0.01282,0.01256,0.01215,0.01162,0.01099,0.01031,0.00961,0.00893,0.00829,0.00772,0.00723,0.00684,0.00653,0.00631,0.00615,0.00606,0.00602,0.00602,0.00605,0.00611,0.0062,0.00633,0.00648,0.00668,0.00691,0.00719,0.0075,0.00785,0.00822,0.00861,0.009,0.00937,0.0097,0.00999,0.01021,0.01035],"B1229":[0.0042,0.005,0.00568,0.00626,0.00674,0.00715,0.00752,0.00784,0.00814,0.00843,0.00871,0.00899,0.00928,0.00957,0.00985,0.01014,0.01043,0.01072,0.011,0.01128,0.01155,0.01182,0.01208,0.01234,0.01258,0.01281,0.01302,0.01319,0.01332,0.01337,0.01334,0.01318,0.01289,0.01242,0.01176,0.0109,0.00982,0.00854,0.00707,0.00544,0.00369,0.00186,0.00001,-0.00181,-0.00355,-0.00518,-0.00665,-0.00794,-0.00903,-0.00992,-0.01061,-0.01111,-0.01143,-0.01159,-0.0116,-0.01148,-0.01124,-0.01089,-0.01042,-0.00983,-0.00912,-0.00829,-0.00735,-0.0063,-0.00515,-0.00393,-0.00265,-0.00137,-0.0001,0.00112,0.00225,0.00328],"B1230":[0.00628,0.00617,0.00602,0.00582,0.00559,0.00531,0.005,0.00466,0.00428,0.00389,0.00348,0.00305,0.00261,0.00218,0.00174,0.00133,0.00095,0.00061,0.00033,0.00011,-0.00003,-0.00008,-0.00004,0.00008,0.00029,0.00056,0.00089,0.00125,0.0016,0.00192,0.00217,0.00231,0.00228,0.00207,0.00163,0.00096,0.00003,-0.00112,-0.00248,-0.004,-0.0056,-0.0072,-0.00874,-0.01011,-0.01124,-0.01206,-0.01253,-0.01262,-0.01233,-0.01168,-0.01071,-0.00947,-0.00804,-0.00647,-0.00484,-0.00323,-0.00168,-0.00024,0.00106,0.0022,0.00317,0.00398,0.00463,0.00514,0.00553,0.00583,0.00605,0.0062,0.0063,0.00636,0.00637,0.00635],"B1231":[0.01916,0.01975,0.02003,0.02005,0.01982,0.0194,0.01881,0.01811,0.01734,0.01651,0.01567,0.01484,0.01403,0.01327,0.01257,0.01194,0.01139,0.01094,0.0106,0.01039,0.0103,0.01035,0.01055,0.01089,0.01138,0.012,0.01274,0.01359,0.01448,0.01539,0.01625,0.01699,0.01755,0.01784,0.01781,0.01742,0.01661,0.0154,0.01379,0.01182,0.00954,0.00705,0.00442,0.00176,-0.00084,-0.00331,-0.00555,-0.00751,-0.00915,-0.01042,-0.01132,-0.01184,-0.01199,-0.0118,-0.01128,-0.01046,-0.00938,-0.00806,-0.00653,-0.00482,-0.00296,-0.00095,0.00115,0.00333,0.00555,0.00777,0.00993,0.012,0.01391,0.01562,0.01709,0.01828],"B1232":[0.02194,0.02063,0.01889,0.01673,0.0142,0.0114,0.00835,0.00516,0.00189,-0.00137,-0.00457,-0.00764,-0.01053,-0.01317,-0.01558,-0.01769,-0.0195,-0.02098,-0.02213,-0.02293,-0.02338,-0.02349,-0.02323,-0.02264,-0.02169,-0.0204,-0.01878,-0.01685,-0.01464,-0.0122,-0.00956,-0.00683,-0.00403,-0.00127,0.00137,0.0038,0.00594,0.00772,0.00907,0.00999,0.01044,0.01045,0.01004,0.00927,0.00821,0.00695,0.0056,0.00423,0.00294,0.00182,0.00094,0.00035,0.0001,0.00021,0.00068,0.00149,0.00263,0.00406,0.00572,0.00756,0.00954,0.01156,0.01359,0.01558,0.01743,0.01912,0.02059,0.02177,0.02263,0.0231,0.02316,0.02278],"B1233":[0.02268,0.0227,0.02231,0.02149,0.02028,0.01871,0.01683,0.01469,0.01236,0.0099,0.00737,0.00483,0.00237,0.00001,-0.00218,-0.00418,-0.00592,-0.00737,-0.00852,-0.00932,-0.00975,-0.00981,-0.00948,-0.00876,-0.00765,-0.00617,-0.00434,-0.00218,0.00024,0.00288,0.00566,0.00851,0.01134,0.01409,0.01662,0.01889,0.02081,0.02231,0.02336,0.02396,0.02408,0.02376,0.02303,0.02195,0.02058,0.01898,0.01723,0.01539,0.01353,0.01172,0.01,0.00842,0.00705,0.00589,0.00499,0.00438,0.00407,0.00407,0.00438,0.00501,0.00592,0.00711,0.00852,0.01013,0.01187,0.01369,0.01551,0.01727,0.01889,0.02031,0.02144,0.02225],"B1234":[-0.01683,-0.01803,-0.01899,-0.01971,-0.02016,-0.02034,-0.02027,-0.01996,-0.01947,-0.0188,-0.01802,-0.01717,-0.01626,-0.01536,-0.01446,-0.01361,-0.01281,-0.01207,-0.0114,-0.01081,-0.01031,-0.00987,-0.00952,-0.00923,-0.00901,-0.00882,-0.00866,-0.00849,-0.0083,-0.00805,-0.00773,-0.00731,-0.00678,-0.00616,-0.00544,-0.00464,-0.0038,-0.00293,-0.00207,-0.00123,-0.00044,0.0003,0.00097,0.00157,0.00211,0.0026,0.00304,0.00344,0.00381,0.00415,0.00445,0.00471,0.00491,0.00504,0.00507,0.00498,0.00475,0.00437,0.00381,0.00306,0.00214,0.00102,-0.00025,-0.0017,-0.00327,-0.00496,-0.00673,-0.00854,-0.01036,-0.01214,-0.01384,-0.01541],"B1235":[0.02377,0.02656,0.02905,0.03115,0.03277,0.03384,0.03431,0.03417,0.03342,0.0321,0.03029,0.02807,0.02556,0.02288,0.02011,0.01739,0.01481,0.01244,0.01035,0.00861,0.00724,0.00625,0.00567,0.00547,0.00565,0.00618,0.00705,0.00821,0.00959,0.01116,0.01282,0.0145,0.01609,0.01751,0.01864,0.0194,0.01972,0.01952,0.01878,0.0175,0.0157,0.01345,0.01081,0.0079,0.00486,0.00175,-0.00126,-0.00407,-0.00661,-0.00876,-0.0105,-0.0118,-0.01264,-0.01305,-0.01305,-0.01268,-0.01199,-0.01102,-0.00982,-0.00843,-0.00686,-0.00513,-0.00326,-0.00121,0.00099,0.00339,0.00596,0.0087,0.01161,0.01464,0.01772,0.02079],"B1236":[0.02168,0.02034,0.01882,0.01713,0.01535,0.01349,0.01159,0.00971,0.00786,0.00606,0.00434,0.00272,0.00123,-0.00013,-0.00132,-0.00236,-0.00319,-0.00383,-0.00425,-0.00446,-0.00444,-0.00422,-0.00378,-0.00313,-0.00233,-0.00135,-0.00022,0.00103,0.00239,0.00382,0.0053,0.00678,0.00824,0.00965,0.01094,0.01212,0.01313,0.01397,0.01464,0.01512,0.01545,0.01562,0.01568,0.01566,0.01557,0.01546,0.01535,0.01527,0.01524,0.01528,0.0154,0.01562,0.01593,0.01634,0.01685,0.01744,0.01812,0.01885,0.01964,0.02045,0.02126,0.02206,0.0228,0.02348,0.02405,0.02449,0.02477,0.02484,0.0247,0.02432,0.02369,0.02281],"B1237":[0.02933,0.02873,0.0282,0.02778,0.02748,0.02731,0.02726,0.02729,0.02737,0.02746,0.02752,0.02752,0.02745,0.02729,0.02708,0.02685,0.02667,0.02662,0.02678,0.02721,0.02802,0.02926,0.03099,0.03324,0.036,0.03926,0.043,0.04715,0.05163,0.05636,0.0612,0.06602,0.07072,0.07514,0.07912,0.08259,0.08543,0.08757,0.08895,0.08957,0.08944,0.08859,0.08707,0.08498,0.08238,0.07936,0.07599,0.07238,0.06862,0.06479,0.06094,0.05718,0.05357,0.0502,0.04706,0.04427,0.04184,0.03977,0.03807,0.0367,0.03564,0.03485,0.03427,0.03382,0.03346,0.03312,0.03275,0.03233,0.03184,0.03127,0.03064,0.02998],"B1238":[0.04264,0.04466,0.04644,0.04791,0.04899,0.0496,0.04971,0.04928,0.0483,0.04676,0.04471,0.04221,0.03931,0.03613,0.03273,0.02923,0.02574,0.02235,0.01915,0.01624,0.01369,0.01155,0.00988,0.00869,0.00798,0.00776,0.00797,0.00856,0.00944,0.01052,0.01168,0.01279,0.01375,0.01445,0.0148,0.01473,0.01423,0.0133,0.01199,0.01038,0.00856,0.00666,0.00478,0.00305,0.00157,0.0004,-0.00038,-0.00075,-0.00071,-0.00028,0.00051,0.00163,0.00298,0.00454,0.00624,0.00803,0.00986,0.01171,0.01356,0.01541,0.01725,0.01909,0.02096,0.02288,0.02485,0.02689,0.02903,0.03123,0.0335,0.03582,0.03815,0.04045],"B1239":[0.03432,0.03583,0.03656,0.03651,0.03566,0.03409,0.0319,0.02922,0.0262,0.02299,0.01973,0.01659,0.01367,0.01109,0.00888,0.00711,0.00579,0.00492,0.00448,0.00444,0.00476,0.00539,0.00626,0.00734,0.00856,0.00986,0.01121,0.01254,0.01381,0.01497,0.01599,0.01681,0.01741,0.01776,0.01786,0.01765,0.01717,0.01641,0.01538,0.01415,0.0127,0.01114,0.00949,0.0078,0.00612,0.00449,0.00297,0.00155,0.00028,-0.00083,-0.00178,-0.00257,-0.00318,-0.00363,-0.00389,-0.00396,-0.00381,-0.00343,-0.00277,-0.00179,-0.00047,0.00123,0.00331,0.0058,0.00868,0.01189,0.01536,0.01895,0.0226,0.02612,0.02934,0.03212],"B1240":[0.00707,0.00762,0.00822,0.00884,0.00949,0.01014,0.01078,0.0114,0.01199,0.01255,0.01306,0.01353,0.01394,0.0143,0.01461,0.01486,0.01506,0.01519,0.01527,0.01526,0.01519,0.01502,0.01477,0.01442,0.01397,0.01342,0.01277,0.01201,0.01117,0.01023,0.00921,0.00811,0.00695,0.00575,0.00452,0.0033,0.00209,0.00096,-0.00009,-0.00102,-0.0018,-0.00241,-0.00284,-0.00308,-0.00314,-0.00301,-0.00274,-0.00234,-0.00184,-0.00127,-0.00067,-0.00006,0.00054,0.00111,0.00163,0.0021,0.00251,0.00287,0.00318,0.00344,0.00368,0.00388,0.00406,0.00424,0.00442,0.00461,0.00483,0.00508,0.00537,0.00572,0.00611,0.00656],"K1401":[0.00455,0.00405,0.00353,0.00298,0.00239,0.00177,0.00111,0.00042,-0.00029,-0.00102,-0.00175,-0.00245,-0.00313,-0.00375,-0.0043,-0.00478,-0.00517,-0.00545,-0.00563,-0.00571,-0.00568,-0.00555,-0.00534,-0.00504,-0.00467,-0.00425,-0.00379,-0.0033,-0.00279,-0.00228,-0.00176,-0.00126,-0.00076,-0.00027,0.0002,0.00067,0.00114,0.00162,0.0021,0.00261,0.00315,0.00372,0.00433,0.00499,0.00569,0.00644,0.00723,0.00805,0.00889,0.00973,0.01055,0.01132,0.01201,0.0126,0.01306,0.01337,0.01352,0.01349,0.0133,0.01294,0.01244,0.01182,0.01112,0.01037,0.00959,0.00882,0.00808,0.00738,0.00673,0.00613,0.00558,0.00506],"L1501":[0.0068,0.00865,0.01057,0.01255,0.01453,0.01647,0.01833,0.02004,0.02157,0.02287,0.02389,0.02462,0.02503,0.02511,0.02487,0.02432,0.0235,0.02243,0.02115,0.01971,0.01816,0.01653,0.01487,0.01324,0.01166,0.01017,0.0088,0.00756,0.00644,0.00543,0.00451,0.00364,0.00276,0.00182,0.00078,-0.0004,-0.00174,-0.00325,-0.00489,-0.00663,-0.00841,-0.01015,-0.01181,-0.0133,-0.01459,-0.01563,-0.01639,-0.01688,-0.0171,-0.01707,-0.01681,-0.01637,-0.01576,-0.01502,-0.01418,-0.01328,-0.01233,-0.01134,-0.01034,-0.00932,-0.00831,-0.00731,-0.0063,-0.00528,-0.00424,-0.00316,-0.00202,-0.00082,0.00048,0.00189,0.00341,0.00505],"L1502":[0.04098,0.04115,0.04072,0.03968,0.03807,0.03596,0.0334,0.0305,0.02736,0.02409,0.0208,0.01757,0.01451,0.01171,0.00921,0.00708,0.00535,0.00401,0.00308,0.00254,0.00235,0.00248,0.00292,0.00361,0.0045,0.00555,0.00674,0.00799,0.00924,0.01043,0.01148,0.01231,0.01286,0.01308,0.0129,0.01232,0.01134,0.01001,0.00838,0.00652,0.00458,0.00261,0.00072,-0.00098,-0.00243,-0.00358,-0.00441,-0.0049,-0.00505,-0.00488,-0.00441,-0.00368,-0.00271,-0.00152,-0.00013,0.00144,0.00321,0.00516,0.00729,0.0096,0.0121,0.01476,0.01756,0.0205,0.0235,0.02651,0.02946,0.03226,0.03483,0.03707,0.03889,0.04021],"L1503":[0.02561,0.02386,0.02189,0.01974,0.01748,0.01514,0.01275,0.01037,0.00803,0.00572,0.0035,0.00136,-0.00066,-0.00255,-0.00429,-0.00586,-0.00722,-0.00835,-0.00921,-0.00978,-0.01,-0.00986,-0.00932,-0.00838,-0.00703,-0.00529,-0.00319,-0.00076,0.00186,0.00461,0.00734,0.00992,0.01224,0.01414,0.01552,0.01631,0.01646,0.01598,0.01491,0.01333,0.01135,0.00911,0.00675,0.00443,0.00229,0.00043,-0.00105,-0.00207,-0.0026,-0.00262,-0.00213,-0.00114,0.00027,0.00207,0.0042,0.00657,0.00912,0.01178,0.01447,0.0171,0.01964,0.02198,0.02408,0.02592,0.02741,0.02854,0.02928,0.02962,0.02956,0.0291,0.02828,0.0271],"M1501":[-0.01432,-0.01465,-0.01506,-0.01558,-0.01625,-0.01706,-0.018,-0.01906,-0.02018,-0.02133,-0.02246,-0.02352,-0.02445,-0.02523,-0.02582,-0.0262,-0.02637,-0.02633,-0.02609,-0.02566,-0.02508,-0.02436,-0.02355,-0.02267,-0.02176,-0.02083,-0.01992,-0.01903,-0.01819,-0.0174,-0.01664,-0.01594,-0.01527,-0.01462,-0.01399,-0.01335,-0.01271,-0.01203,-0.01133,-0.01058,-0.00978,-0.00893,-0.00802,-0.00706,-0.00606,-0.00503,-0.004,-0.00299,-0.00201,-0.00113,-0.00034,0.00029,0.00075,0.001,0.00101,0.00078,0.00029,-0.00044,-0.00139,-0.00252,-0.00381,-0.00518,-0.00657,-0.00794,-0.00923,-0.01039,-0.01138,-0.0122,-0.01285,-0.01334,-0.01372,-0.01403]},"scan_amp_C7":{"A1011":[0.05098,0.05026,0.04912,0.04752,0.04548,0.043,0.04012,0.03694,0.03349,0.02986,0.02614,0.02242,0.01878,0.01531,0.01206,0.00906,0.00638,0.00403,0.00202,0.00034,-0.001,-0.00204,-0.00278,-0.00324,-0.00344,-0.00341,-0.00314,-0.00265,-0.00194,-0.00102,0.00012,0.00147,0.00304,0.00482,0.00678,0.00891,0.01118,0.01355,0.01597,0.0184,0.02078,0.02307,0.02525,0.02727,0.0291,0.03076,0.03223,0.03353,0.03467,0.03567,0.03656,0.03737,0.03812,0.03883,0.03952,0.0402,0.04089,0.04159,0.04231,0.04306,0.04384,0.04465,0.04548,0.04634,0.04721,0.04809,0.04895,0.04973,0.05042,0.05095,0.05126,0.05129],"A1012":[0.03589,0.03423,0.03175,0.02853,0.02474,0.0205,0.01596,0.01128,0.00659,0.00199,-0.00238,-0.00643,-0.01008,-0.01322,-0.0158,-0.01778,-0.01907,-0.01966,-0.01954,-0.01869,-0.01717,-0.01496,-0.01218,-0.00884,-0.00506,-0.00093,0.00348,0.00809,0.01274,0.0174,0.02197,0.0263,0.03038,0.03413,0.03746,0.04034,0.0427,0.04453,0.04577,0.04641,0.04643,0.0458,0.04453,0.04264,0.04014,0.03706,0.0335,0.02953,0.02525,0.02085,0.01641,0.01212,0.00818,0.00469,0.00184,-0.00023,-0.00144,-0.00171,-0.00103,0.00057,0.00302,0.00621,0.00997,0.01414,0.01846,0.02274,0.02677,0.03029,0.03316,0.03524,0.0364,0.03662],"A1013":[0.03642,0.03842,0.03937,0.03916,0.03778,0.03526,0.03169,0.02725,0.0221,0.01642,0.01045,0.0044,-0.00159,-0.00731,-0.01265,-0.01751,-0.02178,-0.02539,-0.02829,-0.03043,-0.03176,-0.03226,-0.0319,-0.03071,-0.02863,-0.02574,-0.02205,-0.01762,-0.01258,-0.00701,-0.00104,0.00516,0.01148,0.01776,0.02385,0.02958,0.03487,0.03953,0.04351,0.04673,0.0491,0.05058,0.05117,0.05085,0.04967,0.04767,0.04495,0.04158,0.0377,0.03346,0.02892,0.02428,0.01968,0.01524,0.01106,0.00731,0.00409,0.00147,-0.00044,-0.00162,-0.00198,-0.00151,-0.00022,0.00189,0.00472,0.00821,0.01223,0.0166,0.02113,0.02561,0.02981,0.03348],"A1101":[0.01043,0.01117,0.01175,0.01214,0.01235,0.01236,0.01219,0.01185,0.01135,0.01071,0.00995,0.0091,0.00817,0.00719,0.00619,0.00518,0.00418,0.00321,0.00228,0.00142,0.00062,-0.0001,-0.00075,-0.00132,-0.00181,-0.00223,-0.00257,-0.00285,-0.00307,-0.00324,-0.00337,-0.00346,-0.00352,-0.00357,-0.0036,-0.00363,-0.00366,-0.00369,-0.00372,-0.00377,-0.00382,-0.00388,-0.00396,-0.00404,-0.00412,-0.00421,-0.00429,-0.00436,-0.00441,-0.00444,-0.00445,-0.00441,-0.00433,-0.00421,-0.00402,-0.00377,-0.00346,-0.00307,-0.00261,-0.00207,-0.00144,-0.00074,0.00006,0.00094,0.00191,0.00294,0.00404,0.00517,0.00632,0.00746,0.00854,0.00954],"A1102":[-0.0059,-0.00679,-0.00769,-0.0086,-0.00947,-0.01028,-0.01097,-0.01151,-0.01185,-0.01196,-0.01181,-0.01137,-0.01064,-0.00963,-0.00833,-0.00678,-0.00501,-0.00307,-0.00098,0.00117,0.00334,0.00548,0.00753,0.00943,0.01115,0.01265,0.01391,0.01491,0.01565,0.01614,0.0164,0.01645,0.01633,0.01605,0.01567,0.0152,0.01468,0.01413,0.01356,0.01297,0.0124,0.01182,0.01124,0.01068,0.01011,0.00954,0.00898,0.00841,0.00786,0.00731,0.00677,0.00624,0.00572,0.00522,0.00472,0.00424,0.00376,0.00328,0.0028,0.00231,0.00182,0.00131,0.00079,0.00026,-0.0003,-0.00088,-0.00148,-0.00211,-0.00278,-0.00349,-0.00425,-0.00505],"A1103":[0.01038,0.01036,0.01041,0.01057,0.01083,0.01118,0.0116,0.01208,0.01258,0.0131,0.01361,0.01409,0.01455,0.01496,0.01534,0.01564,0.0159,0.01608,0.0162,0.01623,0.01619,0.01605,0.01582,0.01549,0.01506,0.01454,0.01392,0.01321,0.01243,0.01156,0.01063,0.00966,0.00864,0.0076,0.00654,0.00549,0.00447,0.00347,0.00253,0.00163,0.0008,0.00004,-0.00064,-0.00123,-0.00172,-0.0021,-0.00236,-0.00248,-0.00245,-0.00227,-0.00192,-0.00141,-0.00075,0.00005,0.00099,0.00202,0.00312,0.00426,0.00539,0.00649,0.00753,0.00846,0.00926,0.00992,0.01041,0.01075,0.01093,0.01098,0.01094,0.0108,0.01065,0.0105],"A1104":[0.04673,0.04535,0.04323,0.04044,0.03711,0.03335,0.02927,0.02503,0.02075,0.01652,0.01247,0.00866,0.00514,0.00199,-0.00083,-0.00331,-0.00545,-0.0073,-0.00891,-0.01032,-0.01156,-0.01268,-0.0137,-0.01465,-0.01551,-0.01628,-0.01694,-0.01746,-0.01781,-0.01794,-0.01784,-0.01747,-0.01683,-0.01592,-0.01477,-0.01341,-0.0119,-0.01031,-0.00873,-0.00723,-0.00589,-0.00478,-0.00395,-0.00342,-0.00319,-0.00324,-0.00353,-0.00397,-0.00449,-0.00499,-0.00535,-0.00549,-0.0053,-0.0047,-0.00363,-0.00206,0.00004,0.00267,0.00581,0.00937,0.01333,0.01754,0.0219,0.02632,0.03063,0.0347,0.03839,0.04157,0.04414,0.046,0.04706,0.04731],"A1105":[0.00208,0.00237,0.00259,0.00274,0.00282,0.00284,0.0028,0.0027,0.00258,0.00243,0.00228,0.00213,0.00201,0.0019,0.00183,0.00178,0.00176,0.00176,0.00177,0.00179,0.0018,0.00181,0.00181,0.0018,0.00179,0.00178,0.00177,0.00177,0.00178,0.0018,0.00183,0.00188,0.00193,0.002,0.00207,0.00215,0.00223,0.00231,0.00237,0.00243,0.00246,0.00246,0.00243,0.00234,0.0022,0.002,0.00174,0.00142,0.00104,0.00062,0.00017,-0.0003,-0.00077,-0.00123,-0.00165,-0.00202,-0.00233,-0.00255,-0.00269,-0.00273,-0.00267,-0.00252,-0.00228,-0.00195,-0.00156,-0.00111,-0.00063,-0.00013,0.00037,0.00086,0.00131,0.00172],"A1106":[0.00888,0.00718,0.00552,0.00394,0.00245,0.00104,-0.0003,-0.00159,-0.00286,-0.00414,-0.00546,-0.00683,-0.00826,-0.00973,-0.01123,-0.01272,-0.01414,-0.01545,-0.01658,-0.01749,-0.01812,-0.01844,-0.01842,-0.01803,-0.01729,-0.0162,-0.01479,-0.01308,-0.01115,-0.00902,-0.00676,-0.00443,-0.00207,0.00028,0.00256,0.00475,0.0068,0.00872,0.01046,0.01204,0.01343,0.01464,0.01569,0.01656,0.01727,0.01785,0.01832,0.01869,0.01899,0.01925,0.01948,0.01971,0.01994,0.02018,0.02044,0.02069,0.02095,0.02118,0.02137,0.02149,0.0215,0.0214,0.02113,0.02069,0.02005,0.01921,0.01817,0.01693,0.01553,0.01398,0.01232,0.01062],"A1107":[0.01485,0.01495,0.01491,0.01469,0.01427,0.01365,0.01281,0.01179,0.01059,0.00925,0.00782,0.00634,0.00484,0.00339,0.00202,0.00076,-0.00034,-0.00126,-0.00199,-0.00252,-0.00283,-0.00294,-0.00286,-0.0026,-0.00218,-0.00162,-0.00094,-0.00017,0.00066,0.00154,0.00244,0.00333,0.00421,0.00505,0.00583,0.00654,0.00717,0.00771,0.00816,0.0085,0.00875,0.00891,0.009,0.00903,0.00902,0.00899,0.00896,0.00894,0.00895,0.00899,0.00907,0.00919,0.00935,0.00954,0.00975,0.00998,0.01022,0.01047,0.01071,0.01095,0.01119,0.01143,0.01167,0.01193,0.01221,0.01252,0.01285,0.01321,0.01358,0.01396,0.01432,0.01462],"A1108":[0.01374,0.01301,0.01211,0.01107,0.00994,0.00875,0.00753,0.00631,0.00514,0.00402,0.00298,0.00203,0.00119,0.00046,-0.00017,-0.00069,-0.00112,-0.00148,-0.00178,-0.00204,-0.00228,-0.00253,-0.00281,-0.00312,-0.00348,-0.00389,-0.00435,-0.00485,-0.00538,-0.00593,-0.00648,-0.00701,-0.00751,-0.00796,-0.00836,-0.0087,-0.00898,-0.0092,-0.00936,-0.00947,-0.00954,-0.00956,-0.00953,-0.00946,-0.00934,-0.00915,-0.0089,-0.00858,-0.00816,-0.00765,-0.00702,-0.00628,-0.00542,-0.00442,-0.0033,-0.00207,-0.00071,0.00074,0.00226,0.00383,0.00543,0.00701,0.00853,0.00997,0.01126,0.01239,0.01332,0.01401,0.01446,0.01465,0.01458,0.01428],"A1109":[0.00879,0.00896,0.00902,0.00895,0.00875,0.0084,0.0079,0.00726,0.00648,0.00557,0.00455,0.00343,0.00221,0.00092,-0.00044,-0.00186,-0.00331,-0.00479,-0.00628,-0.00777,-0.00922,-0.01064,-0.01199,-0.01325,-0.01439,-0.01539,-0.01623,-0.01689,-0.01735,-0.01761,-0.01767,-0.01753,-0.0172,-0.0167,-0.01607,-0.01533,-0.01451,-0.01364,-0.01275,-0.01187,-0.01103,-0.01022,-0.00947,-0.00877,-0.00812,-0.00752,-0.00695,-0.00639,-0.00584,-0.00528,-0.0047,-0.0041,-0.00348,-0.00282,-0.00213,-0.00142,-0.0007,0.00005,0.00078,0.00152,0.00225,0.00296,0.00365,0.00431,0.00496,0.00558,0.00617,0.00673,0.00725,0.00773,0.00816,0.00852],"A1110":[0.00035,0.00217,0.00397,0.00567,0.00722,0.00858,0.0097,0.01054,0.01108,0.01134,0.01129,0.01098,0.01044,0.00968,0.00876,0.00772,0.00659,0.00543,0.00425,0.0031,0.00199,0.00095,-0.00001,-0.00087,-0.00163,-0.00226,-0.00276,-0.00312,-0.00334,-0.00343,-0.00337,-0.00319,-0.00288,-0.00248,-0.002,-0.00148,-0.00095,-0.00044,0.00001,0.00037,0.00062,0.00072,0.00068,0.00048,0.00013,-0.00037,-0.00099,-0.00171,-0.00251,-0.00337,-0.00426,-0.00516,-0.00604,-0.00689,-0.0077,-0.00843,-0.00909,-0.00966,-0.01013,-0.01048,-0.01071,-0.0108,-0.01073,-0.01048,-0.01005,-0.00941,-0.00856,-0.00751,-0.00625,-0.0048,-0.00319,-0.00147],"A1111":[-0.00771,-0.01223,-0.01667,-0.02103,-0.02512,-0.02892,-0.03239,-0.03546,-0.03813,-0.04041,-0.0423,-0.04383,-0.04503,-0.04594,-0.0466,-0.04703,-0.04727,-0.04735,-0.04728,-0.04709,-0.04676,-0.04632,-0.04574,-0.04503,-0.04416,-0.04312,-0.04188,-0.04042,-0.03873,-0.0368,-0.03459,-0.03212,-0.02939,-0.02641,-0.02324,-0.01991,-0.01647,-0.013,-0.00958,-0.00626,-0.00313,-0.00025,0.00236,0.00465,0.00663,0.00831,0.00972,0.0109,0.01191,0.01279,0.01359,0.01438,0.01517,0.016,0.01687,0.01775,0.01864,0.01949,0.02024,0.02081,0.02116,0.02119,0.02085,0.02007,0.01882,0.01706,0.01478,0.01199,0.00875,0.00505,0.00103,-0.00326],"A1112":[0.00799,0.01017,0.01221,0.01407,0.01566,0.01697,0.01797,0.01866,0.01906,0.0192,0.0191,0.01881,0.01836,0.01778,0.01709,0.01631,0.01544,0.0145,0.0135,0.01246,0.01138,0.0103,0.00924,0.00825,0.00733,0.00655,0.00591,0.00545,0.00519,0.00513,0.00526,0.00557,0.00603,0.00661,0.00727,0.00795,0.00862,0.00922,0.00971,0.01006,0.01023,0.0102,0.00994,0.00946,0.00877,0.00786,0.00677,0.00552,0.00413,0.00267,0.00115,-0.00037,-0.00186,-0.00326,-0.00457,-0.00572,-0.0067,-0.00749,-0.00807,-0.00842,-0.00854,-0.00841,-0.00804,-0.00742,-0.00655,-0.00542,-0.00404,-0.00244,-0.00062,0.00139,0.00353,0.00575],"A1113":[0.00405,0.00209,-0.00018,-0.00273,-0.0055,-0.00843,-0.01144,-0.01442,-0.01731,-0.02002,-0.02245,-0.02452,-0.02618,-0.02734,-0.028,-0.02813,-0.02774,-0.02685,-0.02548,-0.02372,-0.02161,-0.01924,-0.0167,-0.01408,-0.01145,-0.00891,-0.00652,-0.00433,-0.00239,-0.0007,0.00072,0.00188,0.00279,0.00348,0.00398,0.00432,0.00451,0.00458,0.00455,0.00442,0.00421,0.00393,0.00359,0.0032,0.00278,0.00234,0.00191,0.0015,0.00113,0.00084,0.00062,0.00051,0.00052,0.00065,0.00092,0.00132,0.00185,0.00251,0.00326,0.00408,0.00496,0.00583,0.00667,0.00742,0.00806,0.00852,0.00877,0.00877,0.00849,0.00789,0.00696,0.00568],"A1114":[0.00909,0.01055,0.0119,0.01315,0.01424,0.01519,0.01596,0.01657,0.01699,0.01723,0.0173,0.01719,0.01691,0.01649,0.01594,0.01527,0.01451,0.01369,0.01282,0.01192,0.01103,0.01014,0.0093,0.00851,0.00778,0.00713,0.00657,0.0061,0.00571,0.00542,0.0052,0.00506,0.00498,0.00493,0.00489,0.00486,0.00479,0.00467,0.00448,0.0042,0.00382,0.00334,0.00274,0.00203,0.00123,0.00033,-0.00062,-0.00162,-0.00263,-0.00362,-0.00459,-0.00548,-0.00627,-0.00695,-0.00749,-0.00786,-0.00806,-0.00808,-0.00791,-0.00754,-0.00699,-0.00625,-0.00535,-0.00428,-0.00307,-0.00174,-0.00031,0.0012,0.00276,0.00437,0.00598,0.00756],"A1115":[0.01072,0.00877,0.00654,0.00405,0.00137,-0.00143,-0.00426,-0.00704,-0.00971,-0.01222,-0.01449,-0.0165,-0.01824,-0.01971,-0.02092,-0.02189,-0.02265,-0.02321,-0.02361,-0.02384,-0.0239,-0.0238,-0.02351,-0.02301,-0.02231,-0.02136,-0.02017,-0.01872,-0.01705,-0.01516,-0.01308,-0.01087,-0.00857,-0.00622,-0.00392,-0.00169,0.00039,0.00227,0.00392,0.00532,0.00645,0.0073,0.00788,0.00823,0.00837,0.00835,0.0082,0.00798,0.00773,0.00749,0.00731,0.00722,0.00724,0.00739,0.00768,0.00811,0.00867,0.00934,0.0101,0.01093,0.01179,0.01264,0.01345,0.01418,0.01478,0.01521,0.01544,0.01543,0.01514,0.01454,0.01361,0.01234],"A1116":[0.02278,0.02225,0.02169,0.02109,0.02046,0.0198,0.01909,0.01833,0.01752,0.01664,0.01569,0.01469,0.01363,0.01253,0.0114,0.01023,0.00906,0.00788,0.00671,0.00555,0.00441,0.0033,0.00223,0.00123,0.00029,-0.00055,-0.00127,-0.00185,-0.00227,-0.0025,-0.00254,-0.00235,-0.00195,-0.00132,-0.00047,0.00057,0.00182,0.00322,0.00475,0.00639,0.0081,0.00984,0.01158,0.01327,0.01489,0.0164,0.0178,0.01905,0.02015,0.0211,0.02189,0.02255,0.02308,0.0235,0.02383,0.02409,0.0243,0.02448,0.02463,0.02477,0.0249,0.025,0.02508,0.02513,0.02513,0.02507,0.02494,0.02474,0.02447,0.02413,0.02374,0.02328],"A1117":[0.03063,0.03227,0.03351,0.03434,0.03472,0.03466,0.03415,0.03319,0.03181,0.03002,0.02785,0.02536,0.02256,0.01954,0.01631,0.01291,0.0094,0.00584,0.00223,-0.00135,-0.00489,-0.00837,-0.01173,-0.01497,-0.01807,-0.02098,-0.0237,-0.02619,-0.02846,-0.03047,-0.03221,-0.03365,-0.03482,-0.0357,-0.03631,-0.03665,-0.03678,-0.0367,-0.03647,-0.03613,-0.0357,-0.03522,-0.03471,-0.03417,-0.03361,-0.03301,-0.03235,-0.03161,-0.03076,-0.02977,-0.0286,-0.02724,-0.02567,-0.02388,-0.02186,-0.01962,-0.01717,-0.0145,-0.01166,-0.00864,-0.00549,-0.00222,0.00111,0.00451,0.00791,0.01128,0.0146,0.01779,0.02084,0.02369,0.0263,0.02862],"A1118":[-0.00074,-0.00014,0.0004,0.00089,0.0013,0.00167,0.00198,0.00224,0.00247,0.00268,0.00287,0.00305,0.00322,0.00337,0.0035,0.00358,0.00362,0.00359,0.00348,0.00327,0.00297,0.00256,0.00205,0.00146,0.00079,0.00007,-0.00068,-0.00142,-0.00213,-0.00278,-0.00335,-0.00381,-0.00415,-0.00437,-0.00446,-0.00444,-0.00433,-0.00413,-0.00389,-0.00362,-0.00334,-0.00308,-0.00285,-0.00266,-0.00252,-0.00244,-0.00242,-0.00246,-0.00257,-0.00273,-0.00296,-0.00324,-0.00356,-0.00391,-0.00429,-0.00467,-0.00503,-0.00535,-0.00563,-0.00584,-0.00596,-0.00599,-0.00591,-0.00573,-0.00544,-0.00505,-0.00456,-0.00401,-0.00339,-0.00273,-0.00205,-0.00139],"A1119":[0.03316,0.03423,0.03487,0.03502,0.03458,0.03355,0.03187,0.02957,0.02667,0.0232,0.01926,0.01493,0.01031,0.00555,0.00072,-0.00408,-0.00866,-0.01297,-0.01692,-0.02042,-0.0234,-0.02583,-0.02766,-0.02889,-0.02952,-0.02957,-0.02908,-0.0281,-0.02669,-0.02491,-0.02284,-0.02059,-0.01819,-0.01572,-0.01326,-0.01086,-0.00856,-0.00641,-0.00444,-0.00265,-0.00107,0.0003,0.00147,0.00244,0.00322,0.00383,0.00431,0.00467,0.00495,0.00517,0.00539,0.00562,0.0059,0.00626,0.00671,0.00728,0.00797,0.00879,0.00976,0.01087,0.01212,0.0135,0.01501,0.01666,0.01842,0.02029,0.02224,0.02423,0.02624,0.0282,0.03006,0.03174],"A1120":[0.00589,0.00702,0.00797,0.00868,0.00917,0.00943,0.00947,0.00934,0.00906,0.00871,0.00832,0.00796,0.00769,0.00756,0.0076,0.00786,0.00834,0.00905,0.00998,0.01112,0.01243,0.01386,0.01539,0.01694,0.01847,0.01992,0.02125,0.02243,0.0234,0.02416,0.02467,0.02492,0.02491,0.02463,0.0241,0.0233,0.02227,0.02101,0.01955,0.01792,0.01615,0.01428,0.01234,0.01037,0.00841,0.00648,0.00462,0.00282,0.00112,-0.00045,-0.00192,-0.00327,-0.00446,-0.00552,-0.00642,-0.00715,-0.0077,-0.00807,-0.00824,-0.00821,-0.00798,-0.00755,-0.00692,-0.00609,-0.0051,-0.00394,-0.00265,-0.00126,0.00021,0.00171,0.00319,0.00459],"A1121":[0.00662,0.00588,0.0051,0.0043,0.00347,0.00263,0.00177,0.00091,0.00006,-0.00078,-0.00161,-0.00241,-0.00319,-0.00393,-0.00464,-0.00532,-0.00597,-0.0066,-0.00721,-0.0078,-0.0084,-0.00901,-0.00962,-0.01026,-0.01093,-0.01162,-0.01234,-0.01309,-0.01386,-0.01462,-0.01539,-0.01612,-0.01679,-0.01739,-0.01789,-0.01826,-0.01847,-0.01851,-0.01835,-0.018,-0.01744,-0.0167,-0.01577,-0.01469,-0.01347,-0.01213,-0.01069,-0.00919,-0.00764,-0.00606,-0.00447,-0.00289,-0.00133,0.0002,0.00167,0.00306,0.00437,0.00558,0.00667,0.00762,0.00843,0.00907,0.00955,0.00985,0.00999,0.00996,0.00979,0.00949,0.00908,0.00857,0.00798,0.00733],"A1122":[0.02114,0.02148,0.02161,0.02147,0.02101,0.02017,0.01892,0.01728,0.01524,0.01286,0.01021,0.0074,0.0045,0.00165,-0.00107,-0.00354,-0.00567,-0.00739,-0.00862,-0.00933,-0.00948,-0.0091,-0.00815,-0.00667,-0.00473,-0.00234,0.00042,0.00348,0.00674,0.01013,0.01352,0.01685,0.02002,0.02296,0.02558,0.02782,0.02966,0.03105,0.03201,0.03253,0.03265,0.03241,0.03185,0.03102,0.02998,0.02879,0.0275,0.02617,0.02483,0.02355,0.02233,0.02121,0.02021,0.01934,0.01861,0.01802,0.01756,0.01722,0.017,0.01687,0.01684,0.01688,0.01699,0.01717,0.01741,0.01772,0.01809,0.01853,0.01903,0.01957,0.02013,0.02067],"A1123":[-0.00446,-0.00121,0.00214,0.00552,0.00876,0.01177,0.01447,0.01677,0.01864,0.02006,0.02102,0.02156,0.02171,0.02152,0.02107,0.02039,0.01958,0.01868,0.01774,0.01682,0.01594,0.01513,0.01442,0.01382,0.01331,0.01292,0.01261,0.01239,0.01224,0.01215,0.01211,0.01211,0.01214,0.0122,0.01227,0.01234,0.0124,0.01243,0.01242,0.01235,0.0122,0.01196,0.01162,0.01117,0.01063,0.00998,0.00924,0.00841,0.00749,0.00649,0.00539,0.00419,0.00289,0.00148,-0.00006,-0.0017,-0.00344,-0.00528,-0.00715,-0.00901,-0.01081,-0.01247,-0.01391,-0.01507,-0.01585,-0.01619,-0.01604,-0.01537,-0.01415,-0.01239,-0.01015,-0.00748],"A1124":[0.02819,0.02774,0.02724,0.02676,0.02635,0.02609,0.02601,0.02613,0.02646,0.02701,0.02772,0.02858,0.02952,0.03049,0.03144,0.03229,0.03298,0.03347,0.03371,0.03365,0.03328,0.0326,0.0316,0.03028,0.02867,0.02683,0.02476,0.0225,0.02013,0.01767,0.01519,0.01273,0.01034,0.00804,0.00587,0.00388,0.00205,0.0004,-0.00107,-0.00239,-0.00356,-0.0046,-0.00551,-0.00631,-0.00698,-0.00753,-0.00791,-0.00811,-0.00809,-0.00782,-0.00725,-0.00639,-0.0052,-0.00369,-0.00187,0.00022,0.00255,0.00509,0.00775,0.01047,0.01323,0.01589,0.01843,0.02077,0.02287,0.02467,0.02615,0.02727,0.02805,0.02848,0.02862,0.02851],"A1125":[-0.02798,-0.02851,-0.02858,-0.02808,-0.02692,-0.02506,-0.02251,-0.01933,-0.01561,-0.01142,-0.00695,-0.00231,0.00239,0.00692,0.01127,0.01529,0.01889,0.02202,0.02463,0.02669,0.02821,0.02918,0.02964,0.02961,0.02915,0.0283,0.02713,0.02569,0.02406,0.0223,0.02045,0.01859,0.01673,0.01493,0.0132,0.01151,0.00987,0.00822,0.00651,0.00467,0.00265,0.00038,-0.00219,-0.00506,-0.00824,-0.01173,-0.0154,-0.01918,-0.02296,-0.02656,-0.02989,-0.03281,-0.03518,-0.03693,-0.03802,-0.03842,-0.03816,-0.03731,-0.03595,-0.03424,-0.03228,-0.03028,-0.02836,-0.02667,-0.02533,-0.02441,-0.02397,-0.024,-0.02444,-0.02521,-0.02616,-0.02714],"A1126":[0.01428,0.0155,0.01699,0.01874,0.02065,0.02266,0.02466,0.02653,0.02819,0.02958,0.03061,0.03125,0.0315,0.03137,0.0309,0.03014,0.02915,0.028,0.02675,0.02545,0.02414,0.02286,0.02163,0.02044,0.01928,0.01816,0.01703,0.01589,0.01471,0.01347,0.01217,0.0108,0.00937,0.00788,0.00635,0.00479,0.00324,0.00173,0.00026,-0.00115,-0.00246,-0.00369,-0.0048,-0.00578,-0.00662,-0.00731,-0.00784,-0.00819,-0.00834,-0.00829,-0.00801,-0.0075,-0.00676,-0.00579,-0.00461,-0.00325,-0.00173,-0.00011,0.00158,0.00326,0.00489,0.00639,0.00772,0.00886,0.00978,0.01048,0.01101,0.01141,0.01176,0.01214,0.01264,0.01333],"A1127":[-0.00103,-0.00005,0.00102,0.0021,0.00315,0.00411,0.00492,0.00558,0.00605,0.00634,0.00647,0.00645,0.00631,0.00609,0.00582,0.00552,0.00524,0.00498,0.00475,0.00457,0.00445,0.00437,0.00432,0.00431,0.00431,0.00433,0.00434,0.00434,0.00432,0.00428,0.00422,0.00412,0.00399,0.00382,0.0036,0.00333,0.00298,0.00256,0.00205,0.00144,0.00076,0.00001,-0.00079,-0.00159,-0.00238,-0.00311,-0.00373,-0.00421,-0.00453,-0.00467,-0.00464,-0.00443,-0.00409,-0.00363,-0.00312,-0.00259,-0.0021,-0.00168,-0.00139,-0.00124,-0.00124,-0.00139,-0.00166,-0.00202,-0.00241,-0.00279,-0.00308,-0.00324,-0.00322,-0.00299,-0.00254,-0.00188],"A1128":[0.02851,0.02546,0.0223,0.01906,0.01578,0.0125,0.00927,0.00612,0.00311,0.00028,-0.00231,-0.00457,-0.0065,-0.00803,-0.00915,-0.00985,-0.01015,-0.01008,-0.00968,-0.009,-0.0081,-0.00704,-0.00589,-0.0047,-0.00351,-0.00237,-0.00132,-0.00036,0.00048,0.0012,0.0018,0.00229,0.00269,0.00302,0.00329,0.00353,0.00377,0.00403,0.00434,0.00473,0.00523,0.00586,0.00664,0.0076,0.00876,0.01011,0.01167,0.01343,0.01538,0.01748,0.01974,0.02212,0.02458,0.02709,0.02959,0.03204,0.0344,0.03661,0.03861,0.04036,0.04182,0.04291,0.04361,0.04389,0.04373,0.04313,0.04209,0.04063,0.0388,0.03661,0.03414,0.03143],"A1129":[0.00889,0.00786,0.00678,0.00564,0.00445,0.0032,0.00189,0.00055,-0.00082,-0.0022,-0.00354,-0.00482,-0.00598,-0.00699,-0.00781,-0.00839,-0.00871,-0.00875,-0.0085,-0.00796,-0.00718,-0.00615,-0.00494,-0.00361,-0.00219,-0.00076,0.00064,0.00195,0.00312,0.00412,0.00494,0.00557,0.00602,0.00632,0.00649,0.00658,0.00663,0.00668,0.00675,0.00688,0.00706,0.00731,0.0076,0.00795,0.00832,0.00872,0.00913,0.00955,0.00998,0.01043,0.01089,0.01137,0.01187,0.01239,0.01292,0.01344,0.01393,0.01439,0.01478,0.01509,0.01529,0.01538,0.01533,0.01516,0.01485,0.01443,0.01388,0.01323,0.01249,0.01168,0.0108,0.00988],"A1130":[0.01665,0.01512,0.01358,0.01205,0.01054,0.00906,0.00763,0.00624,0.00488,0.00356,0.00229,0.00107,-0.0001,-0.0012,-0.00221,-0.00315,-0.00397,-0.00468,-0.00527,-0.00573,-0.00607,-0.00627,-0.00633,-0.00627,-0.00606,-0.00571,-0.0052,-0.00454,-0.0037,-0.00267,-0.00146,-0.00005,0.00155,0.00333,0.00527,0.00735,0.00955,0.01178,0.01403,0.01625,0.01837,0.02036,0.02217,0.02378,0.02515,0.02631,0.02723,0.02794,0.02846,0.02881,0.02903,0.02915,0.02919,0.02916,0.02911,0.02902,0.02891,0.02877,0.02859,0.02837,0.0281,0.02775,0.02731,0.02677,0.02612,0.02535,0.02444,0.02341,0.02225,0.02097,0.0196,0.01816],"A1131":[0.02813,0.02743,0.02665,0.02578,0.02483,0.02378,0.02263,0.02137,0.01999,0.01851,0.01693,0.01528,0.0136,0.01194,0.01033,0.00883,0.0075,0.00636,0.00548,0.00488,0.00459,0.00461,0.00493,0.00556,0.00644,0.00755,0.00884,0.01027,0.01175,0.01324,0.01472,0.0161,0.01739,0.01856,0.01957,0.02046,0.0212,0.02181,0.0223,0.02268,0.02296,0.02314,0.02322,0.02321,0.02313,0.02297,0.02275,0.02249,0.02221,0.02194,0.02171,0.02154,0.02148,0.02153,0.02172,0.02205,0.02255,0.02319,0.02395,0.02481,0.02571,0.02664,0.0275,0.0283,0.02896,0.02946,0.02977,0.0299,0.02985,0.02962,0.02924,0.02874],"A1132":[0.00918,0.00827,0.00742,0.00661,0.00585,0.00514,0.00445,0.00382,0.00323,0.00269,0.00224,0.00189,0.00167,0.00162,0.00176,0.00213,0.00274,0.00359,0.0047,0.00604,0.00762,0.00939,0.01133,0.0134,0.01556,0.01776,0.01999,0.0222,0.02434,0.02642,0.02838,0.03022,0.03193,0.03352,0.03496,0.03627,0.03745,0.03851,0.03945,0.04027,0.04095,0.04148,0.04187,0.04208,0.04211,0.04196,0.04161,0.04107,0.04033,0.03943,0.03836,0.03715,0.03583,0.03442,0.03292,0.03139,0.02983,0.02825,0.02669,0.02515,0.02365,0.0222,0.0208,0.01944,0.01814,0.01687,0.01566,0.01448,0.01334,0.01222,0.01117,0.01015],"A1133":[0.0245,0.02355,0.02239,0.02104,0.01953,0.01788,0.01613,0.01432,0.01245,0.01057,0.0087,0.00689,0.00514,0.00352,0.00202,0.00067,-0.00049,-0.00148,-0.00229,-0.00293,-0.00342,-0.0038,-0.00407,-0.00429,-0.00447,-0.00463,-0.0048,-0.00497,-0.00515,-0.00534,-0.00551,-0.00567,-0.00579,-0.00585,-0.00587,-0.00583,-0.00573,-0.0056,-0.00542,-0.00523,-0.00502,-0.00481,-0.00459,-0.00435,-0.00409,-0.00379,-0.00342,-0.00297,-0.0024,-0.0017,-0.00085,0.00018,0.00136,0.00272,0.00424,0.00591,0.0077,0.0096,0.01156,0.01354,0.01551,0.01742,0.01921,0.02087,0.02234,0.02357,0.02456,0.02526,0.02568,0.02581,0.02564,0.0252],"A1134":[-0.0032,-0.00328,-0.00336,-0.00343,-0.0035,-0.00355,-0.00358,-0.00357,-0.0035,-0.00336,-0.00315,-0.00286,-0.00248,-0.002,-0.00144,-0.00079,-0.00006,0.00074,0.0016,0.00251,0.00346,0.00444,0.00544,0.00644,0.00744,0.00841,0.00936,0.01026,0.01111,0.0119,0.0126,0.01323,0.01377,0.01421,0.01456,0.01481,0.01497,0.01503,0.015,0.01488,0.01468,0.01438,0.014,0.01354,0.013,0.0124,0.01174,0.01101,0.01024,0.00944,0.00859,0.00772,0.00685,0.00596,0.00507,0.00421,0.00336,0.00255,0.00177,0.00105,0.00038,-0.00023,-0.00078,-0.00127,-0.00169,-0.00204,-0.00234,-0.00257,-0.00276,-0.00291,-0.00302,-0.00312],"A1135":[0.03863,0.03896,0.03875,0.03801,0.03673,0.03494,0.0327,0.0301,0.02722,0.02416,0.02106,0.01801,0.01508,0.01238,0.00995,0.00783,0.00607,0.00465,0.00356,0.0028,0.00235,0.00219,0.00228,0.00259,0.00311,0.00378,0.00458,0.00546,0.00637,0.00726,0.00808,0.00876,0.00927,0.00957,0.00962,0.0094,0.00892,0.00819,0.00723,0.00608,0.0048,0.00343,0.00204,0.00069,-0.00055,-0.00162,-0.00248,-0.00308,-0.00337,-0.00333,-0.00296,-0.00225,-0.00122,0.0001,0.0017,0.00351,0.0055,0.00765,0.00988,0.01219,0.01456,0.01695,0.01935,0.02177,0.02418,0.02656,0.02888,0.03109,0.03317,0.03502,0.0366,0.03782],"A1136":[0.0149,0.01345,0.01189,0.01025,0.00859,0.00693,0.0053,0.00373,0.00226,0.0009,-0.00031,-0.00131,-0.00208,-0.00258,-0.00278,-0.00265,-0.00219,-0.00138,-0.00023,0.00122,0.00296,0.00491,0.00704,0.00929,0.01158,0.01388,0.01611,0.01824,0.02021,0.02199,0.02357,0.02491,0.02604,0.02692,0.02758,0.02799,0.02818,0.02813,0.02784,0.02732,0.02657,0.0256,0.02443,0.02309,0.02161,0.02003,0.01841,0.0168,0.01525,0.01384,0.01259,0.01156,0.01079,0.0103,0.01011,0.01022,0.01061,0.01126,0.01212,0.01314,0.01426,0.01541,0.0165,0.01749,0.01829,0.01885,0.01914,0.01912,0.01881,0.0182,0.01732,0.01621],"A1137":[0.0445,0.04569,0.04656,0.04704,0.0471,0.0467,0.04585,0.04456,0.04286,0.04081,0.03849,0.03598,0.03336,0.03074,0.02817,0.02576,0.02355,0.02159,0.01993,0.01857,0.0175,0.01671,0.01619,0.01587,0.01572,0.01567,0.0157,0.01574,0.01573,0.01564,0.01544,0.01509,0.01457,0.01389,0.01304,0.01202,0.01084,0.00953,0.00811,0.0066,0.00505,0.00348,0.00194,0.0005,-0.0008,-0.0019,-0.00277,-0.00332,-0.00357,-0.00345,-0.00294,-0.00205,-0.00078,0.00084,0.00279,0.00501,0.00745,0.01006,0.01276,0.01553,0.01828,0.02101,0.02363,0.02619,0.02864,0.03099,0.03326,0.03543,0.03751,0.03949,0.04135,0.04303],"A1138":[0.01176,0.0102,0.00848,0.00662,0.00469,0.0027,0.00071,-0.00124,-0.00314,-0.00495,-0.00666,-0.00824,-0.00972,-0.01107,-0.01231,-0.01343,-0.01443,-0.01532,-0.01608,-0.0167,-0.01715,-0.01742,-0.01749,-0.01732,-0.01689,-0.0162,-0.01524,-0.01399,-0.0125,-0.01076,-0.00883,-0.00675,-0.00457,-0.00234,-0.00015,0.00196,0.00392,0.00569,0.00722,0.0085,0.00951,0.01025,0.01075,0.01103,0.01112,0.01107,0.01093,0.01073,0.01052,0.01034,0.01021,0.01016,0.0102,0.01036,0.01062,0.01099,0.01146,0.01201,0.01264,0.0133,0.01398,0.01463,0.01524,0.01575,0.01614,0.01637,0.0164,0.01623,0.01582,0.01516,0.01426,0.01312],"A1139":[0.0101,0.00898,0.00762,0.00601,0.00416,0.00213,-0.0001,-0.00244,-0.00483,-0.00727,-0.00964,-0.01191,-0.01402,-0.0159,-0.01752,-0.01885,-0.01982,-0.02043,-0.02065,-0.02047,-0.01989,-0.01891,-0.01754,-0.0158,-0.01372,-0.01134,-0.00871,-0.00586,-0.00289,0.00015,0.00319,0.00613,0.00895,0.01156,0.01388,0.01588,0.01755,0.01885,0.01979,0.02038,0.02064,0.02063,0.02038,0.01994,0.01935,0.01867,0.01794,0.01719,0.01646,0.01576,0.01512,0.01455,0.01404,0.01361,0.01326,0.01297,0.01275,0.01259,0.01249,0.01243,0.01242,0.01244,0.01249,0.01255,0.01261,0.01265,0.01265,0.01258,0.01241,0.01211,0.01164,0.01098],"A1140":[0.00767,0.00796,0.00807,0.00799,0.00774,0.00732,0.00675,0.00605,0.00523,0.00433,0.00337,0.00237,0.00137,0.00039,-0.00055,-0.00143,-0.00223,-0.00293,-0.00354,-0.00402,-0.00438,-0.00461,-0.0047,-0.00464,-0.00442,-0.00403,-0.00348,-0.00276,-0.00188,-0.00085,0.00031,0.00157,0.00292,0.00431,0.00572,0.00711,0.00843,0.00964,0.01072,0.01163,0.01234,0.01283,0.01309,0.01311,0.01287,0.0124,0.01171,0.01082,0.00976,0.00858,0.0073,0.00597,0.00465,0.00337,0.00219,0.00114,0.00027,-0.0004,-0.00085,-0.00104,-0.001,-0.00072,-0.00023,0.00044,0.00126,0.00217,0.00314,0.0041,0.00503,0.00588,0.00663,0.00723],"A1141":[0.01206,0.01164,0.01096,0.01004,0.00893,0.00765,0.00626,0.00481,0.00334,0.00188,0.00051,-0.00076,-0.00189,-0.00286,-0.00365,-0.00425,-0.00466,-0.00488,-0.00492,-0.0048,-0.00452,-0.00411,-0.0036,-0.00299,-0.0023,-0.00157,-0.00081,-0.00002,0.00077,0.00156,0.00233,0.00307,0.00377,0.00441,0.00499,0.00548,0.00588,0.00616,0.00632,0.00634,0.00621,0.00594,0.00552,0.00497,0.0043,0.00354,0.00271,0.00186,0.00101,0.0002,-0.00052,-0.00113,-0.00159,-0.00187,-0.00197,-0.00186,-0.00154,-0.00101,-0.00029,0.00062,0.00168,0.00286,0.00413,0.00544,0.00676,0.00802,0.0092,0.01023,0.01107,0.01171,0.01209,0.01222],"B1021":[0.04565,0.04638,0.04674,0.04667,0.04611,0.04503,0.0434,0.04124,0.03855,0.03535,0.03171,0.02769,0.02337,0.01886,0.01421,0.00952,0.00489,0.0004,-0.00388,-0.00784,-0.01146,-0.01468,-0.01746,-0.01976,-0.02159,-0.02293,-0.02379,-0.0242,-0.02416,-0.02373,-0.02292,-0.02178,-0.02033,-0.0186,-0.01663,-0.01445,-0.01206,-0.00955,-0.00691,-0.0042,-0.00144,0.00129,0.00399,0.00659,0.00906,0.01141,0.01358,0.01557,0.01739,0.01902,0.0205,0.02182,0.02301,0.02408,0.02507,0.02599,0.02687,0.02773,0.02859,0.02947,0.03039,0.03137,0.03242,0.03356,0.03479,0.03612,0.03753,0.03899,0.04049,0.04197,0.04337,0.04462],"B1022":[0.00421,0.00157,-0.00165,-0.00533,-0.00935,-0.01356,-0.01786,-0.02208,-0.02617,-0.03006,-0.03364,-0.03688,-0.03972,-0.04214,-0.04411,-0.04559,-0.04654,-0.04693,-0.04672,-0.04591,-0.04448,-0.04241,-0.03973,-0.03646,-0.03264,-0.02836,-0.02367,-0.01866,-0.01344,-0.00816,-0.00288,0.0022,0.00704,0.0115,0.01548,0.0189,0.02171,0.02383,0.02527,0.02601,0.02605,0.02541,0.02413,0.02227,0.01989,0.01704,0.01383,0.01038,0.00677,0.00315,-0.00038,-0.00371,-0.00671,-0.00924,-0.01127,-0.01266,-0.0134,-0.01346,-0.01284,-0.0116,-0.0098,-0.00756,-0.00503,-0.00234,0.00033,0.00281,0.00493,0.00655,0.00756,0.00786,0.0074,0.00618],"B1023":[0.04344,0.04645,0.04845,0.04936,0.0491,0.04771,0.04524,0.04184,0.03767,0.03293,0.02787,0.02272,0.01772,0.01309,0.00899,0.00552,0.00283,0.00092,-0.00016,-0.00047,-0.00004,0.00107,0.0028,0.00507,0.00789,0.01111,0.01472,0.01873,0.02298,0.02745,0.03204,0.03658,0.04101,0.04512,0.04875,0.05173,0.0539,0.05512,0.05528,0.05432,0.05223,0.04905,0.04487,0.03985,0.03414,0.02793,0.02148,0.01499,0.00865,0.00267,-0.00276,-0.00757,-0.0116,-0.0148,-0.01713,-0.01859,-0.01915,-0.01889,-0.0178,-0.01596,-0.01341,-0.0102,-0.00643,-0.0021,0.00267,0.00782,0.01325,0.01881,0.02439,0.02983,0.03494,0.03953],"B1201":[0.00394,0.002,-0.00018,-0.00256,-0.00508,-0.0077,-0.01037,-0.01298,-0.01547,-0.0178,-0.01989,-0.02166,-0.02309,-0.02412,-0.02473,-0.02491,-0.02466,-0.02399,-0.02292,-0.02149,-0.01976,-0.01778,-0.01561,-0.01333,-0.01099,-0.00866,-0.0064,-0.00425,-0.00224,-0.0004,0.00127,0.00275,0.00407,0.00523,0.00625,0.00715,0.00793,0.00859,0.00914,0.00957,0.00989,0.01008,0.01015,0.01009,0.00991,0.00963,0.00926,0.00885,0.00842,0.008,0.00763,0.00733,0.00713,0.00705,0.00709,0.00725,0.00751,0.00786,0.00828,0.00873,0.00918,0.0096,0.00996,0.01022,0.01035,0.01033,0.01013,0.00973,0.00909,0.0082,0.00705,0.00564],"B1202":[0.01658,0.01821,0.01972,0.02108,0.02226,0.02329,0.02416,0.02486,0.0254,0.0258,0.02607,0.02623,0.0263,0.02628,0.0262,0.02604,0.02581,0.02552,0.02516,0.02473,0.02421,0.02362,0.02296,0.02223,0.02143,0.02059,0.01972,0.01882,0.0179,0.01699,0.01608,0.01519,0.0143,0.01341,0.01251,0.01158,0.01062,0.0096,0.0085,0.0073,0.006,0.00459,0.00306,0.00142,-0.00029,-0.00206,-0.00385,-0.0056,-0.00727,-0.00881,-0.01016,-0.01127,-0.01212,-0.01266,-0.01287,-0.01276,-0.01232,-0.01156,-0.0105,-0.00918,-0.00761,-0.00585,-0.00394,-0.0019,0.00023,0.0024,0.0046,0.00676,0.00888,0.01095,0.01292,0.01481],"B1203":[0.02892,0.0293,0.02914,0.02846,0.02727,0.02561,0.02354,0.02113,0.01844,0.01555,0.01255,0.00951,0.00651,0.00362,0.00087,-0.0017,-0.00403,-0.00614,-0.00803,-0.00967,-0.01107,-0.01225,-0.01318,-0.01387,-0.0143,-0.01445,-0.01431,-0.01387,-0.0131,-0.012,-0.01058,-0.00886,-0.00685,-0.00461,-0.00221,0.00027,0.00276,0.00514,0.0073,0.00916,0.01062,0.01161,0.01208,0.01202,0.01144,0.01038,0.00892,0.00713,0.00513,0.00305,0.001,-0.00092,-0.00257,-0.00387,-0.00476,-0.00518,-0.00509,-0.00449,-0.00338,-0.00181,0.0002,0.00257,0.00523,0.00811,0.0111,0.01413,0.01711,0.01992,0.0225,0.02476,0.02661,0.02801],"B1204":[0.00871,0.00762,0.0065,0.00533,0.00413,0.00289,0.0016,0.0003,-0.00102,-0.00232,-0.00355,-0.00467,-0.00563,-0.00637,-0.00686,-0.00704,-0.00691,-0.00644,-0.00563,-0.00451,-0.00311,-0.00146,0.00037,0.00233,0.00434,0.00632,0.0082,0.00992,0.0114,0.01258,0.01343,0.0139,0.01398,0.01368,0.01299,0.01195,0.01061,0.00904,0.00728,0.00542,0.00353,0.00169,-0.00002,-0.00153,-0.00279,-0.00374,-0.00434,-0.00458,-0.00445,-0.00394,-0.00309,-0.00194,-0.00053,0.00107,0.00281,0.00461,0.0064,0.00813,0.00972,0.01114,0.01234,0.01327,0.01393,0.01432,0.01443,0.01428,0.0139,0.01333,0.01259,0.01173,0.01078,0.00977],"B1205":[0.01669,0.01115,0.0053,-0.00075,-0.00684,-0.01285,-0.0187,-0.02421,-0.02933,-0.03396,-0.03804,-0.04154,-0.04443,-0.04671,-0.04842,-0.0496,-0.05027,-0.05051,-0.05036,-0.04986,-0.04905,-0.04797,-0.04663,-0.04502,-0.04316,-0.04103,-0.0386,-0.03586,-0.03281,-0.02944,-0.02575,-0.02177,-0.01753,-0.01309,-0.00852,-0.00389,0.0007,0.00514,0.00938,0.01331,0.01689,0.02007,0.02283,0.02514,0.02705,0.0286,0.02982,0.03079,0.03156,0.03221,0.0328,0.03338,0.034,0.0347,0.03549,0.03638,0.03736,0.0384,0.03944,0.04043,0.04129,0.04194,0.0423,0.04228,0.04179,0.04078,0.03918,0.03697,0.03411,0.03061,0.02652,0.02186],"B1206":[0.02768,0.03269,0.03703,0.04064,0.04342,0.0454,0.04654,0.04688,0.04644,0.04529,0.0435,0.04116,0.03834,0.03518,0.03175,0.02816,0.02457,0.02103,0.0177,0.01467,0.01209,0.00998,0.00847,0.00759,0.00738,0.0078,0.00887,0.0105,0.01258,0.01501,0.01764,0.02035,0.02297,0.02536,0.02736,0.02887,0.02978,0.03,0.02948,0.02819,0.02611,0.02329,0.01975,0.01557,0.01087,0.00571,0.00024,-0.00539,-0.01107,-0.01661,-0.02192,-0.02684,-0.03125,-0.03504,-0.03813,-0.04043,-0.04188,-0.04244,-0.04209,-0.04081,-0.03863,-0.03558,-0.03174,-0.02712,-0.02188,-0.0161,-0.00991,-0.00346,0.00309,0.00966,0.01603,0.02206],"B1207":[0.02804,0.03043,0.03254,0.0343,0.03563,0.0365,0.03687,0.03674,0.03614,0.0351,0.03369,0.03198,0.03006,0.02803,0.02595,0.02392,0.02201,0.02027,0.01876,0.01753,0.01659,0.01596,0.01564,0.01563,0.0159,0.01643,0.01717,0.01811,0.01916,0.02028,0.02142,0.0225,0.02347,0.02426,0.02482,0.02508,0.02501,0.02454,0.02367,0.02237,0.02065,0.01852,0.01604,0.01328,0.01031,0.00721,0.0041,0.00109,-0.00175,-0.00429,-0.00648,-0.00824,-0.00953,-0.01033,-0.01061,-0.0104,-0.00973,-0.00864,-0.00718,-0.00542,-0.00341,-0.0012,0.00113,0.0036,0.00615,0.00878,0.01149,0.01424,0.01704,0.01988,0.02268,0.02543],"B1208":[0.00633,0.00485,0.00334,0.00186,0.00048,-0.00076,-0.00182,-0.00266,-0.00328,-0.00366,-0.00383,-0.00381,-0.00365,-0.00337,-0.00302,-0.00265,-0.0023,-0.002,-0.00177,-0.00163,-0.00159,-0.00164,-0.00177,-0.00195,-0.00217,-0.00239,-0.00258,-0.00271,-0.00276,-0.0027,-0.00253,-0.00223,-0.00183,-0.00131,-0.00071,-0.00006,0.00063,0.00132,0.00198,0.0026,0.00316,0.00365,0.00407,0.00443,0.00474,0.00502,0.00527,0.00552,0.00579,0.00608,0.0064,0.00676,0.00717,0.00762,0.00811,0.00863,0.00917,0.00973,0.01029,0.01083,0.01134,0.01179,0.01216,0.01241,0.01251,0.01245,0.01219,0.01172,0.01103,0.01012,0.00901,0.00774],"B1209":[0.0128,0.01581,0.01879,0.02166,0.02431,0.02665,0.02861,0.0301,0.03108,0.03153,0.03145,0.03086,0.0298,0.02832,0.0265,0.0244,0.02212,0.01971,0.01725,0.01478,0.01235,0.01,0.00774,0.00561,0.00358,0.0017,-0.00006,-0.0017,-0.00322,-0.0046,-0.00585,-0.00697,-0.00796,-0.00882,-0.00954,-0.01014,-0.01062,-0.01101,-0.01131,-0.01154,-0.01173,-0.0119,-0.01206,-0.01222,-0.0124,-0.01261,-0.01283,-0.01306,-0.0133,-0.01352,-0.01372,-0.01387,-0.01396,-0.01397,-0.01389,-0.0137,-0.0134,-0.01297,-0.01242,-0.01174,-0.01091,-0.00995,-0.00883,-0.00755,-0.00609,-0.00445,-0.0026,-0.00054,0.00176,0.00426,0.00696,0.00981],"B1210":[-0.03088,-0.03327,-0.0352,-0.03661,-0.03744,-0.03768,-0.03736,-0.03654,-0.03528,-0.03367,-0.03181,-0.02982,-0.0278,-0.02586,-0.02408,-0.02255,-0.02134,-0.02048,-0.02002,-0.01996,-0.0203,-0.02102,-0.02207,-0.02341,-0.02498,-0.0267,-0.0285,-0.03029,-0.032,-0.03354,-0.03485,-0.03586,-0.03654,-0.03685,-0.03679,-0.03638,-0.03563,-0.03462,-0.03338,-0.03199,-0.0305,-0.02898,-0.02746,-0.02599,-0.02457,-0.02321,-0.02191,-0.02065,-0.0194,-0.01816,-0.01688,-0.01556,-0.01422,-0.01285,-0.01147,-0.01012,-0.00887,-0.00773,-0.00681,-0.00616,-0.00584,-0.00591,-0.00642,-0.00741,-0.00888,-0.01081,-0.01318,-0.01587,-0.01884,-0.02197,-0.0251,-0.02811],"B1211":[0.01329,0.01417,0.01482,0.01521,0.01529,0.01504,0.01449,0.01367,0.01262,0.01143,0.01021,0.00901,0.00791,0.00696,0.00619,0.0056,0.00517,0.00486,0.00461,0.00435,0.00401,0.00354,0.00287,0.002,0.00086,-0.00053,-0.00215,-0.004,-0.00603,-0.00819,-0.01043,-0.01269,-0.01493,-0.01709,-0.0191,-0.02091,-0.02248,-0.02376,-0.02471,-0.02532,-0.02556,-0.02543,-0.02493,-0.02409,-0.02293,-0.02148,-0.01979,-0.0179,-0.01587,-0.01376,-0.01161,-0.00946,-0.00737,-0.00539,-0.00355,-0.00188,-0.0004,0.00088,0.00196,0.00287,0.00362,0.00425,0.00481,0.00535,0.00591,0.00654,0.00727,0.00811,0.00906,0.0101,0.01119,0.01228],"B1212":[-0.00529,-0.00723,-0.00916,-0.01104,-0.01281,-0.01445,-0.01594,-0.01725,-0.01838,-0.01933,-0.02012,-0.02074,-0.02122,-0.02157,-0.02182,-0.02198,-0.02205,-0.02203,-0.02192,-0.02172,-0.0214,-0.02095,-0.02036,-0.01959,-0.01864,-0.01749,-0.01614,-0.01459,-0.01285,-0.01095,-0.0089,-0.00677,-0.00458,-0.00237,-0.00022,0.00183,0.00373,0.00543,0.00689,0.00811,0.00905,0.00973,0.01015,0.01035,0.01035,0.0102,0.00994,0.0096,0.00923,0.00885,0.00851,0.00821,0.00798,0.00781,0.00772,0.00769,0.00771,0.00776,0.00781,0.00783,0.00778,0.00764,0.00737,0.00693,0.00632,0.0055,0.00448,0.00325,0.00183,0.00023,-0.00151,-0.00336],"B1213":[0.017,0.01539,0.01327,0.01064,0.00759,0.00418,0.00048,-0.00337,-0.00727,-0.01114,-0.01484,-0.0183,-0.02144,-0.0242,-0.02655,-0.02847,-0.02995,-0.031,-0.03163,-0.03185,-0.03169,-0.03117,-0.03031,-0.02914,-0.02768,-0.02597,-0.02403,-0.02189,-0.0196,-0.01721,-0.01473,-0.01226,-0.00979,-0.00737,-0.00508,-0.00293,-0.00096,0.0008,0.0023,0.00357,0.00455,0.00527,0.00572,0.00593,0.0059,0.00569,0.00532,0.00485,0.00432,0.00378,0.00329,0.00289,0.00263,0.00254,0.00266,0.00299,0.00356,0.00435,0.00536,0.00656,0.00793,0.00941,0.01096,0.01254,0.01406,0.01549,0.01674,0.01774,0.01844,0.01877,0.01867,0.01809],"B1214":[0.00012,0.00086,0.00185,0.00308,0.00451,0.0061,0.0078,0.00952,0.01123,0.01285,0.01433,0.01562,0.01668,0.01749,0.01802,0.01827,0.01824,0.01794,0.01737,0.01658,0.01556,0.01436,0.01301,0.01155,0.01003,0.00849,0.00697,0.00551,0.00415,0.00293,0.00185,0.00095,0.00022,-0.00032,-0.00068,-0.00088,-0.00092,-0.00082,-0.0006,-0.00029,0.00009,0.00053,0.00099,0.00146,0.00191,0.00233,0.00271,0.00304,0.0033,0.0035,0.00363,0.0037,0.00371,0.00368,0.00359,0.00346,0.0033,0.0031,0.00287,0.0026,0.0023,0.00197,0.0016,0.00121,0.0008,0.00039,0.0,-0.00033,-0.00058,-0.00069,-0.00063,-0.00037],"B1215":[0.00845,0.00952,0.01037,0.01101,0.0114,0.01156,0.0115,0.01124,0.01079,0.01019,0.00948,0.00867,0.0078,0.0069,0.00599,0.0051,0.00425,0.00347,0.00275,0.00212,0.00159,0.00116,0.00083,0.0006,0.00045,0.00039,0.00039,0.00043,0.00051,0.00059,0.00067,0.00073,0.00075,0.00073,0.00065,0.0005,0.00025,-0.00009,-0.00056,-0.00116,-0.00191,-0.00279,-0.00381,-0.00495,-0.00617,-0.00746,-0.00876,-0.01002,-0.01122,-0.01228,-0.01319,-0.01391,-0.0144,-0.01466,-0.01468,-0.01445,-0.01399,-0.01331,-0.01243,-0.01137,-0.01015,-0.0088,-0.00734,-0.00578,-0.00415,-0.00247,-0.00076,0.00094,0.00262,0.00425,0.00579,0.0072],"B1216":[0.00507,0.00527,0.00551,0.00576,0.00594,0.006,0.00587,0.00546,0.00473,0.00362,0.00211,0.00022,-0.00206,-0.00467,-0.00756,-0.01063,-0.01382,-0.01701,-0.02014,-0.02307,-0.02577,-0.02816,-0.03017,-0.03175,-0.03289,-0.03356,-0.03376,-0.03353,-0.03287,-0.03183,-0.03046,-0.02881,-0.02694,-0.02489,-0.02274,-0.02053,-0.01832,-0.01616,-0.01409,-0.01213,-0.01034,-0.00871,-0.00725,-0.00597,-0.00485,-0.00388,-0.00303,-0.00229,-0.00161,-0.00098,-0.00037,0.00024,0.00085,0.00148,0.00212,0.00275,0.00337,0.00396,0.00448,0.00494,0.00532,0.00558,0.00574,0.00579,0.00574,0.00562,0.00545,0.00527,0.00509,0.00496,0.00491,0.00494],"B1217":[-0.01904,-0.01938,-0.01931,-0.01884,-0.01799,-0.0168,-0.01534,-0.01365,-0.01181,-0.00989,-0.00797,-0.00609,-0.00432,-0.00271,-0.00127,-0.00003,0.00098,0.00176,0.00231,0.00265,0.00276,0.00268,0.00241,0.00199,0.00144,0.00077,0.00003,-0.00076,-0.00156,-0.00233,-0.00305,-0.00369,-0.00422,-0.00462,-0.00488,-0.00499,-0.00494,-0.00474,-0.0044,-0.00393,-0.00336,-0.00269,-0.00196,-0.00119,-0.0004,0.00039,0.00115,0.00186,0.0025,0.00305,0.00349,0.00382,0.00401,0.00406,0.00396,0.0037,0.00328,0.00267,0.00188,0.0009,-0.00027,-0.00163,-0.00317,-0.00487,-0.00669,-0.00859,-0.01053,-0.01242,-0.01423,-0.01586,-0.01724,-0.01832],"B1218":[0.01238,0.01274,0.01299,0.0131,0.01309,0.01293,0.01262,0.01218,0.01162,0.01094,0.01018,0.00936,0.00851,0.00766,0.00685,0.0061,0.00545,0.00491,0.0045,0.00423,0.00411,0.00413,0.00427,0.00452,0.00485,0.00523,0.00562,0.00601,0.00636,0.00664,0.00683,0.00692,0.00689,0.00673,0.00644,0.00603,0.00549,0.00484,0.00407,0.00321,0.00227,0.00127,0.00023,-0.00081,-0.00182,-0.00278,-0.00363,-0.00436,-0.00494,-0.00533,-0.00553,-0.00553,-0.00531,-0.0049,-0.00429,-0.00353,-0.00262,-0.0016,-0.00049,0.00067,0.00185,0.00304,0.00419,0.00531,0.00638,0.00738,0.00832,0.00918,0.00998,0.0107,0.01135,0.01191],"B1219":[0.00736,0.00659,0.00573,0.0048,0.0038,0.00273,0.0016,0.00042,-0.00081,-0.00206,-0.00331,-0.00455,-0.00577,-0.00692,-0.00799,-0.00898,-0.00985,-0.01059,-0.0112,-0.01167,-0.01202,-0.01224,-0.01234,-0.01235,-0.01225,-0.01209,-0.01185,-0.01155,-0.01121,-0.01081,-0.01038,-0.00991,-0.0094,-0.00884,-0.00824,-0.00761,-0.00692,-0.0062,-0.00544,-0.00464,-0.00382,-0.00299,-0.00215,-0.00131,-0.00047,0.00035,0.00115,0.00193,0.00269,0.00342,0.00413,0.00482,0.00549,0.00614,0.00677,0.00738,0.00796,0.00851,0.00902,0.00947,0.00985,0.01016,0.01039,0.01052,0.01055,0.01048,0.0103,0.01004,0.00967,0.00922,0.00868,0.00806],"B1220":[0.03121,0.02905,0.02643,0.0234,0.02005,0.01645,0.01265,0.00879,0.00492,0.00111,-0.00254,-0.00599,-0.0092,-0.01209,-0.01468,-0.01693,-0.01885,-0.02043,-0.02167,-0.02257,-0.02313,-0.02338,-0.02326,-0.0228,-0.02199,-0.02081,-0.01929,-0.01736,-0.0151,-0.01249,-0.00959,-0.00642,-0.00306,0.00044,0.00397,0.00744,0.01079,0.01387,0.01663,0.019,0.0209,0.02231,0.02322,0.02365,0.02363,0.02322,0.02252,0.0216,0.02056,0.0195,0.01852,0.01769,0.01711,0.01681,0.01683,0.01719,0.01792,0.01897,0.02031,0.0219,0.02368,0.02556,0.02746,0.02931,0.03101,0.03248,0.03365,0.03444,0.03479,0.03467,0.03404,0.03289],"B1221":[0.01221,0.01332,0.01441,0.01541,0.01627,0.01691,0.01732,0.01746,0.01733,0.01694,0.01633,0.01553,0.01458,0.01354,0.01246,0.01136,0.01031,0.00932,0.00839,0.00757,0.00683,0.00618,0.00562,0.00515,0.00477,0.00448,0.00429,0.00422,0.00427,0.00446,0.00482,0.00534,0.00603,0.00691,0.00794,0.00911,0.01039,0.01174,0.01312,0.0145,0.01581,0.01703,0.01813,0.01907,0.01985,0.02045,0.02088,0.02112,0.02121,0.02113,0.02091,0.02055,0.02007,0.01946,0.01875,0.01793,0.01702,0.01603,0.01498,0.01389,0.01281,0.01176,0.0108,0.00997,0.0093,0.00883,0.00861,0.00864,0.00893,0.00947,0.01022,0.01116],"B1222":[0.0172,0.01718,0.01716,0.01719,0.01726,0.01737,0.0175,0.01762,0.01771,0.01775,0.01772,0.01762,0.01745,0.01725,0.01703,0.01684,0.01672,0.01669,0.0168,0.01706,0.01748,0.01805,0.01877,0.01958,0.02044,0.02129,0.02208,0.02275,0.02322,0.02345,0.02339,0.02303,0.02232,0.02126,0.01989,0.01819,0.01621,0.01399,0.01156,0.00895,0.00624,0.00346,0.00066,-0.00208,-0.00472,-0.00718,-0.0094,-0.01133,-0.01289,-0.01403,-0.01473,-0.01493,-0.01464,-0.01384,-0.01257,-0.01086,-0.00878,-0.00639,-0.00377,-0.00104,0.00175,0.00448,0.00704,0.00938,0.01144,0.01318,0.01457,0.01562,0.01636,0.01683,0.01708,0.01718],"B1223":[-0.00389,-0.00452,-0.00497,-0.00515,-0.00504,-0.00458,-0.00376,-0.00261,-0.00114,0.0006,0.00257,0.00468,0.00688,0.00908,0.01123,0.01323,0.01502,0.01654,0.01775,0.01859,0.01906,0.01915,0.01887,0.01823,0.0173,0.01611,0.01474,0.01325,0.01171,0.01018,0.00874,0.00742,0.00625,0.00525,0.00443,0.00376,0.00322,0.00275,0.00232,0.00186,0.00132,0.00066,-0.00014,-0.00111,-0.00224,-0.0035,-0.00486,-0.00627,-0.00766,-0.00894,-0.01007,-0.01096,-0.01158,-0.01187,-0.01182,-0.01144,-0.01075,-0.00978,-0.00861,-0.0073,-0.00592,-0.00458,-0.00335,-0.00229,-0.00148,-0.00095,-0.00073,-0.00079,-0.00113,-0.00168,-0.00238,-0.00314],"B1224":[-0.00034,0.00016,0.00057,0.00079,0.00078,0.00046,-0.00016,-0.00108,-0.00227,-0.00364,-0.0051,-0.00651,-0.00778,-0.00876,-0.00937,-0.0095,-0.0091,-0.00814,-0.0066,-0.00454,-0.002,0.00096,0.00422,0.00766,0.01118,0.01465,0.01796,0.02101,0.02371,0.02598,0.02779,0.0291,0.02992,0.03026,0.03016,0.02967,0.02884,0.02774,0.02642,0.02494,0.02335,0.0217,0.02,0.01832,0.01666,0.01504,0.01348,0.01199,0.01059,0.00929,0.00808,0.00697,0.00598,0.00509,0.00428,0.00356,0.00291,0.00231,0.00175,0.00122,0.00069,0.00018,-0.00031,-0.00078,-0.0012,-0.00155,-0.00179,-0.0019,-0.00186,-0.00165,-0.00131,-0.00085],"B1225":[0.01326,0.01192,0.01059,0.0093,0.00809,0.00698,0.00601,0.00521,0.00461,0.00425,0.00414,0.00431,0.00476,0.00549,0.00648,0.00769,0.00905,0.01052,0.012,0.01342,0.01468,0.0157,0.01639,0.01669,0.01656,0.01595,0.01488,0.01334,0.01139,0.00908,0.00649,0.00373,0.00086,-0.00203,-0.00482,-0.00745,-0.00986,-0.01201,-0.01386,-0.0154,-0.0166,-0.01746,-0.018,-0.01819,-0.01803,-0.01754,-0.0167,-0.01552,-0.014,-0.01218,-0.01006,-0.00766,-0.00506,-0.0023,0.00057,0.00345,0.00628,0.009,0.01151,0.01376,0.01572,0.01729,0.01849,0.0193,0.0197,0.01975,0.01945,0.01887,0.01804,0.01701,0.01585,0.01458],"B1226":[0.05409,0.05209,0.04965,0.04679,0.04359,0.04009,0.03634,0.03248,0.02853,0.02459,0.02076,0.01709,0.01366,0.01052,0.00771,0.00523,0.00311,0.00136,-0.00006,-0.00114,-0.00191,-0.00239,-0.00259,-0.00256,-0.0023,-0.00185,-0.00123,-0.00047,0.00038,0.0013,0.00226,0.00321,0.00412,0.00495,0.00568,0.00627,0.00672,0.007,0.00713,0.00712,0.007,0.00682,0.00662,0.00646,0.00641,0.00651,0.00683,0.00742,0.00831,0.00954,0.0111,0.01303,0.01528,0.01786,0.02072,0.02382,0.0271,0.03051,0.03397,0.03743,0.04082,0.04404,0.04704,0.04975,0.05212,0.05407,0.05559,0.05662,0.05714,0.05715,0.05664,0.05562],"B1227":[0.00468,0.00509,0.00538,0.0055,0.00543,0.00519,0.00477,0.0042,0.00352,0.00278,0.00204,0.00133,0.00074,0.0003,0.00005,0.00002,0.00024,0.00069,0.00137,0.00224,0.00329,0.00444,0.00566,0.00689,0.0081,0.00923,0.01025,0.01114,0.01189,0.0125,0.01298,0.01333,0.01358,0.01376,0.01387,0.01394,0.01396,0.01395,0.01389,0.01379,0.01363,0.01341,0.01312,0.01275,0.01232,0.01181,0.01125,0.01064,0.00998,0.00929,0.00858,0.00785,0.00711,0.00637,0.00562,0.00489,0.00419,0.00351,0.00289,0.00233,0.00186,0.00149,0.00124,0.00112,0.00113,0.00128,0.00156,0.00195,0.00244,0.003,0.00359,0.00416],"B1228":[0.00344,0.00314,0.00272,0.00217,0.00149,0.00069,-0.00019,-0.00115,-0.00214,-0.00314,-0.0041,-0.00498,-0.00577,-0.00642,-0.00694,-0.0073,-0.00749,-0.00754,-0.00743,-0.00719,-0.00683,-0.00637,-0.00583,-0.00522,-0.00455,-0.00384,-0.0031,-0.00231,-0.00149,-0.00063,0.00026,0.00117,0.00213,0.0031,0.00407,0.00503,0.00596,0.00683,0.00763,0.00832,0.00889,0.00934,0.00964,0.00981,0.00985,0.00976,0.00958,0.00931,0.00898,0.00861,0.00822,0.00781,0.00741,0.00702,0.00664,0.00628,0.00593,0.0056,0.00529,0.005,0.00474,0.0045,0.0043,0.00413,0.00401,0.00392,0.00387,0.00384,0.00383,0.00381,0.00375,0.00364],"B1229":[0.00282,0.00302,0.00306,0.00298,0.00279,0.00252,0.0022,0.00185,0.00151,0.00118,0.0009,0.00069,0.00054,0.00047,0.0005,0.00064,0.00088,0.00123,0.00169,0.00227,0.00295,0.00372,0.00457,0.00546,0.00639,0.0073,0.00817,0.00897,0.00964,0.01017,0.01051,0.01065,0.01056,0.01023,0.00966,0.00887,0.00786,0.00665,0.00528,0.00377,0.00215,0.00047,-0.00125,-0.00296,-0.00464,-0.00624,-0.00775,-0.00912,-0.01035,-0.01139,-0.01224,-0.01288,-0.0133,-0.01351,-0.01349,-0.01325,-0.0128,-0.01215,-0.01134,-0.01036,-0.00926,-0.00805,-0.00679,-0.00549,-0.00418,-0.00291,-0.0017,-0.0006,0.00039,0.00125,0.00194,0.00246],"B1230":[-0.00193,-0.00322,-0.00458,-0.00597,-0.00738,-0.00878,-0.01015,-0.01149,-0.01276,-0.01397,-0.0151,-0.01611,-0.017,-0.01772,-0.01826,-0.01859,-0.01869,-0.01853,-0.0181,-0.01742,-0.01648,-0.01532,-0.01398,-0.01249,-0.01091,-0.00931,-0.00773,-0.00625,-0.00489,-0.00371,-0.00273,-0.00198,-0.00146,-0.00118,-0.00111,-0.00124,-0.00154,-0.00197,-0.00251,-0.00312,-0.00375,-0.00437,-0.00496,-0.00549,-0.00592,-0.00625,-0.00647,-0.00655,-0.00651,-0.00634,-0.00605,-0.00563,-0.00511,-0.0045,-0.00379,-0.00302,-0.0022,-0.00135,-0.00049,0.00033,0.00111,0.0018,0.00237,0.00279,0.00305,0.00311,0.00296,0.0026,0.00204,0.00128,0.00035,-0.00073],"B1231":[0.01466,0.01456,0.01433,0.01396,0.01348,0.01291,0.01226,0.01155,0.01078,0.00998,0.00915,0.00831,0.00748,0.00668,0.0059,0.00518,0.00453,0.00396,0.00347,0.00307,0.00274,0.00249,0.00231,0.00217,0.00207,0.002,0.00193,0.00186,0.00178,0.00169,0.00159,0.00147,0.00133,0.0012,0.00106,0.00092,0.00079,0.00067,0.00055,0.00045,0.00035,0.00025,0.00016,0.00008,0.0,-0.00007,-0.00013,-0.00017,-0.00017,-0.00013,-0.00005,0.0001,0.00033,0.00064,0.00104,0.00153,0.00213,0.00284,0.00364,0.00454,0.00552,0.00656,0.00764,0.00875,0.00983,0.01087,0.01183,0.01268,0.0134,0.01397,0.01436,0.0146],"B1232":[0.00463,0.00478,0.00489,0.00496,0.00499,0.00499,0.00498,0.00496,0.00493,0.00492,0.00491,0.0049,0.00491,0.00492,0.00493,0.00494,0.00495,0.00495,0.00496,0.00498,0.005,0.00505,0.00511,0.0052,0.00533,0.00548,0.00566,0.00586,0.00608,0.0063,0.00652,0.00671,0.00687,0.00697,0.00699,0.00692,0.00674,0.00646,0.00606,0.00554,0.00493,0.00422,0.00344,0.00263,0.00181,0.001,0.00025,-0.00043,-0.00102,-0.00149,-0.00185,-0.00208,-0.00218,-0.00217,-0.00205,-0.00185,-0.00156,-0.00121,-0.00082,-0.00039,0.00006,0.00052,0.00098,0.00144,0.00189,0.00233,0.00275,0.00315,0.00353,0.00387,0.00417,0.00442],"B1233":[0.01558,0.01548,0.01515,0.0146,0.01387,0.01298,0.01199,0.01094,0.00989,0.00887,0.00795,0.00715,0.00649,0.00601,0.00572,0.0056,0.00566,0.00588,0.00624,0.00671,0.00727,0.00789,0.00855,0.00921,0.00985,0.01044,0.01097,0.01141,0.01174,0.01196,0.01204,0.01201,0.01183,0.01154,0.01113,0.01061,0.01001,0.00934,0.00861,0.00785,0.00708,0.00632,0.00557,0.00486,0.0042,0.00361,0.00308,0.00263,0.00226,0.00197,0.00178,0.00168,0.00168,0.00178,0.00198,0.0023,0.00272,0.00326,0.00392,0.00468,0.00554,0.0065,0.00753,0.00861,0.00972,0.01083,0.01191,0.0129,0.01379,0.01453,0.01509,0.01545],"B1234":[-0.00258,-0.00037,0.00205,0.0046,0.00723,0.00983,0.01235,0.01465,0.01667,0.01836,0.01962,0.0204,0.02068,0.02044,0.01971,0.0185,0.01688,0.01494,0.01275,0.01043,0.00807,0.00575,0.00357,0.00159,-0.00015,-0.00161,-0.00279,-0.0037,-0.00437,-0.00483,-0.00512,-0.00527,-0.00532,-0.00529,-0.0052,-0.00506,-0.00487,-0.00461,-0.0043,-0.00391,-0.00344,-0.00289,-0.00229,-0.00162,-0.00092,-0.00019,0.00051,0.00117,0.00176,0.00223,0.00257,0.00275,0.00273,0.00252,0.00209,0.00146,0.00063,-0.00037,-0.00152,-0.00276,-0.00403,-0.00528,-0.00643,-0.00744,-0.00823,-0.00875,-0.00896,-0.00881,-0.0083,-0.0074,-0.00614,-0.00452],"B1235":[-0.00126,-0.00205,-0.00275,-0.00334,-0.00382,-0.00417,-0.0044,-0.00451,-0.00451,-0.00441,-0.0042,-0.00391,-0.00355,-0.00312,-0.00265,-0.00214,-0.00162,-0.00111,-0.00063,-0.0002,0.00015,0.00041,0.00056,0.00059,0.00049,0.00026,-0.0001,-0.00058,-0.00116,-0.00184,-0.00258,-0.00337,-0.00418,-0.00498,-0.00575,-0.00647,-0.00711,-0.00766,-0.00811,-0.00845,-0.00866,-0.00876,-0.00874,-0.00859,-0.00833,-0.00795,-0.00746,-0.00687,-0.00619,-0.00542,-0.00459,-0.00369,-0.00276,-0.0018,-0.00084,0.00009,0.00099,0.00183,0.00259,0.00323,0.00376,0.00413,0.00435,0.00439,0.00426,0.00396,0.00349,0.00287,0.00214,0.00132,0.00045,-0.00042],"B1236":[0.00813,0.00755,0.00695,0.00633,0.00574,0.00519,0.0047,0.00431,0.00402,0.00387,0.00388,0.00405,0.00441,0.00495,0.00568,0.00658,0.00763,0.00881,0.01007,0.01137,0.01266,0.0139,0.01502,0.01599,0.01676,0.01731,0.0176,0.01761,0.01736,0.01684,0.01607,0.01508,0.01389,0.01254,0.01107,0.00952,0.00793,0.00634,0.00477,0.00325,0.00181,0.00045,-0.0008,-0.00193,-0.00294,-0.0038,-0.0045,-0.00503,-0.00539,-0.00555,-0.00552,-0.00527,-0.00483,-0.00418,-0.00334,-0.00233,-0.00119,0.00008,0.00142,0.00277,0.00412,0.00539,0.00654,0.00755,0.00837,0.00899,0.00939,0.00959,0.00959,0.00941,0.00909,0.00866],"B1237":[0.01796,0.01457,0.01073,0.00658,0.00236,-0.00179,-0.00573,-0.00935,-0.01261,-0.01549,-0.01798,-0.02012,-0.02195,-0.02353,-0.02489,-0.02606,-0.02706,-0.02788,-0.0285,-0.02886,-0.02892,-0.0286,-0.02784,-0.02656,-0.02471,-0.02228,-0.01923,-0.01557,-0.01139,-0.00673,-0.00172,0.00346,0.00871,0.01381,0.01859,0.02287,0.02649,0.02928,0.03117,0.03211,0.03205,0.03107,0.02921,0.0266,0.02341,0.01976,0.01589,0.01195,0.0081,0.00454,0.00136,-0.00132,-0.00338,-0.0048,-0.00551,-0.0055,-0.0048,-0.00342,-0.00143,0.00109,0.00405,0.00728,0.01067,0.01402,0.01715,0.0199,0.0221,0.02355,0.02418,0.02393,0.02277,0.02076],"B1238":[0.00997,0.0108,0.01171,0.01265,0.01358,0.01446,0.01526,0.01594,0.0165,0.01693,0.01723,0.01741,0.01748,0.01746,0.01737,0.01722,0.01705,0.01686,0.01667,0.01651,0.01638,0.01631,0.0163,0.01639,0.01658,0.01688,0.0173,0.01785,0.01853,0.0193,0.02017,0.02109,0.02203,0.02294,0.02377,0.02448,0.02501,0.02533,0.0254,0.0252,0.02472,0.02398,0.023,0.02182,0.02048,0.01903,0.01754,0.01607,0.01467,0.01339,0.01226,0.0113,0.01054,0.00995,0.00954,0.00926,0.00908,0.00898,0.00891,0.00884,0.00875,0.00863,0.00847,0.00829,0.0081,0.00794,0.00784,0.00783,0.00795,0.00822,0.00865,0.00923],"B1239":[0.04668,0.04772,0.04833,0.04851,0.04825,0.0476,0.0466,0.04531,0.0438,0.04212,0.04035,0.03853,0.03673,0.03499,0.03331,0.03171,0.03021,0.02877,0.0274,0.02606,0.02473,0.02339,0.02203,0.02063,0.01918,0.0177,0.0162,0.01467,0.01313,0.01162,0.01013,0.00868,0.00729,0.00594,0.00468,0.00348,0.00237,0.00133,0.00039,-0.00047,-0.00123,-0.0019,-0.00247,-0.00292,-0.00326,-0.00347,-0.00354,-0.00346,-0.0032,-0.00275,-0.0021,-0.00123,-0.00014,0.00118,0.00274,0.00451,0.00649,0.00868,0.01103,0.01355,0.01621,0.01897,0.02181,0.02473,0.02765,0.03058,0.03346,0.03621,0.03883,0.04125,0.0434,0.04522],"B1240":[0.00552,0.00633,0.00717,0.00803,0.0089,0.00976,0.01059,0.01135,0.01203,0.01259,0.013,0.01325,0.01332,0.01321,0.01291,0.01243,0.01178,0.01098,0.01004,0.00899,0.00785,0.00664,0.00541,0.00416,0.00292,0.00171,0.00056,-0.00053,-0.00155,-0.00248,-0.00333,-0.00409,-0.00476,-0.00534,-0.00584,-0.00626,-0.00659,-0.00684,-0.00701,-0.00712,-0.00716,-0.00715,-0.00709,-0.00699,-0.00687,-0.00673,-0.00658,-0.00642,-0.00627,-0.00612,-0.00598,-0.00583,-0.00567,-0.00549,-0.00529,-0.00505,-0.00476,-0.00441,-0.004,-0.00353,-0.00299,-0.0024,-0.00176,-0.00108,-0.00038,0.00034,0.00107,0.0018,0.00252,0.00325,0.00399,0.00474],"K1401":[0.0029,0.00365,0.00442,0.00518,0.0059,0.00656,0.00713,0.00762,0.00799,0.00828,0.00848,0.00863,0.00875,0.00887,0.00902,0.00924,0.00954,0.00994,0.01045,0.01105,0.01173,0.01246,0.01323,0.01398,0.0147,0.01532,0.01582,0.01615,0.0163,0.01625,0.01598,0.01547,0.01476,0.01381,0.01268,0.01136,0.0099,0.00831,0.00664,0.00492,0.00317,0.00145,-0.00022,-0.00179,-0.00324,-0.00453,-0.00565,-0.00657,-0.00729,-0.00778,-0.00808,-0.00817,-0.00808,-0.00782,-0.00743,-0.00692,-0.00633,-0.00569,-0.00502,-0.00436,-0.00371,-0.00311,-0.00254,-0.00202,-0.00153,-0.00107,-0.00061,-0.00014,0.00036,0.00091,0.00152,0.00218],"L1501":[0.04567,0.04813,0.05026,0.05201,0.05328,0.05404,0.05427,0.05396,0.05315,0.05186,0.05019,0.0482,0.04595,0.04357,0.04109,0.0386,0.03616,0.0338,0.03155,0.02945,0.02751,0.02574,0.02417,0.02279,0.02158,0.0206,0.01984,0.01932,0.01904,0.01902,0.01925,0.01973,0.02043,0.02133,0.02236,0.02345,0.02453,0.02553,0.02635,0.02694,0.02723,0.02718,0.02678,0.02603,0.02498,0.02367,0.02216,0.02053,0.01885,0.01723,0.01571,0.01437,0.01326,0.01244,0.01193,0.01174,0.01188,0.01235,0.01313,0.01422,0.01558,0.01722,0.01909,0.02118,0.02349,0.02597,0.02862,0.03139,0.03426,0.03719,0.04011,0.04296],"L1502":[0.02533,0.0244,0.02325,0.02192,0.02045,0.01887,0.01725,0.01564,0.01412,0.01274,0.01155,0.01063,0.00998,0.00964,0.00961,0.00988,0.0104,0.01114,0.01205,0.01305,0.01409,0.01511,0.01605,0.01687,0.01751,0.01796,0.01819,0.0182,0.018,0.01758,0.01696,0.01618,0.01526,0.01424,0.01313,0.01197,0.01078,0.00958,0.00842,0.00728,0.00623,0.00526,0.00441,0.00371,0.00319,0.00285,0.00273,0.00283,0.00315,0.0037,0.00445,0.0054,0.00653,0.0078,0.00921,0.0107,0.01227,0.01389,0.01552,0.01714,0.01872,0.02023,0.02164,0.02294,0.02408,0.02503,0.02579,0.02632,0.02662,0.02668,0.02648,0.02603],"L1503":[0.00184,0.003,0.00433,0.00582,0.00742,0.00911,0.01086,0.01263,0.01437,0.01605,0.01764,0.01911,0.02044,0.0216,0.02261,0.02345,0.02413,0.02466,0.02503,0.02527,0.02537,0.02535,0.02518,0.02489,0.02445,0.02386,0.02313,0.02223,0.02117,0.01996,0.0186,0.01712,0.01553,0.01385,0.01213,0.0104,0.00869,0.00704,0.00547,0.00401,0.00269,0.0015,0.00046,-0.00042,-0.00116,-0.00176,-0.00223,-0.00259,-0.00285,-0.00302,-0.00311,-0.00315,-0.00314,-0.00309,-0.00302,-0.00293,-0.00284,-0.00274,-0.00265,-0.00257,-0.00249,-0.00242,-0.00235,-0.00227,-0.00217,-0.00202,-0.00183,-0.00154,-0.00116,-0.00064,0.00002,0.00084],"M1501":[0.01094,0.0092,0.00742,0.00559,0.00376,0.00195,0.00018,-0.00152,-0.00311,-0.00457,-0.00585,-0.00694,-0.0078,-0.00841,-0.00876,-0.00883,-0.00861,-0.00811,-0.00734,-0.00631,-0.00505,-0.00356,-0.0019,-0.00011,0.00179,0.00373,0.00567,0.00756,0.00935,0.01098,0.01244,0.01368,0.01468,0.01546,0.016,0.01632,0.01645,0.01642,0.01628,0.01605,0.01578,0.0155,0.01525,0.01504,0.01492,0.01488,0.01494,0.0151,0.01537,0.01574,0.0162,0.01674,0.01735,0.018,0.01867,0.01934,0.01998,0.02056,0.02105,0.02142,0.02166,0.02173,0.02162,0.02133,0.02083,0.02015,0.01927,0.01823,0.01702,0.01567,0.01419,0.01261]},"ci":{"A1011":[[-0.035821865,0.071839365],[-0.0370473444,0.0728355586],[-0.0382597229,0.0734518657],[-0.039401731,0.0736328025],[-0.040417054,0.0733334825],[-0.0412987169,0.0725694312],[-0.0420621077,0.0713810363],[-0.0427337695,0.0698523409],[-0.043371275,0.0680773464],[-0.0440229466,0.0661672323],[-0.0447376031,0.0642461745],[-0.0455416788,0.0624102503],[-0.0464382879,0.0607247165],[-0.0474060065,0.0592524351],[-0.048397797,0.0579906541],[-0.0493560527,0.0569235527],[-0.0502032578,0.0560346863],[-0.0508775259,0.0552743117],[-0.051322987,0.0546044155],[-0.0514892243,0.05398351],[-0.0513523665,0.0533802236],[-0.0508990581,0.0527683438],[-0.0501364846,0.0521418417],[-0.0490909571,0.0514995285],[-0.0478082102,0.050859996],[-0.0463716804,0.050278109],[-0.0448616775,0.0498081061],[-0.0433851272,0.04952727],[-0.0420673957,0.0495131099],[-0.0410029728,0.0498354728],[-0.0402739969,0.0505422112],[-0.0399157579,0.0516225436],[-0.039921428,0.0530435709],[-0.040232795,0.0547088664],[-0.0407662796,0.0564894939],[-0.0414125802,0.0582390087],[-0.0420656521,0.0598217236],[-0.0426362598,0.0610991169],[-0.0430455251,0.061970168],[-0.0432611863,0.0623733292],[-0.0432431172,0.0622591887],[-0.0430202662,0.0616456233],[-0.0426253659,0.0605817944],[-0.0421132565,0.0591507565],[-0.0415622807,0.0574726378],[-0.0410586398,0.0556836398],[-0.0406875532,0.0539446961],[-0.0405155771,0.0523937914],[-0.040588337,0.0511504799],[-0.0409074899,0.0503085614],[-0.0414272976,0.0498776547],[-0.0420792198,0.0498581483],[-0.0427617185,0.0502010042],[-0.0433544117,0.0508161974],[-0.0437615103,0.051625796],[-0.0438952835,0.0525402835],[-0.0437051226,0.0534933369],[-0.0431605075,0.0544308646],[-0.0422749302,0.0553124302],[-0.0411036951,0.0561429808],[-0.0397055853,0.0569320139],[-0.0381868091,0.0577028805],[-0.0366571629,0.0584971629],[-0.035212257,0.0593693999],[-0.0339701317,0.0603529888],[-0.0330085765,0.0614835765],[-0.0323991912,0.0627834769],[-0.0321613061,0.0642184489],[-0.0323119887,0.0657844887],[-0.0328135134,0.0674117276],[-0.0336156361,0.0690245646],[-0.034641557,0.0705304855]],"A1012":[[-0.035821865,0.071839365],[-0.0370473444,0.0728355586],[-0.0382597229,0.0734518657],[-0.039401731,0.0736328025],[-0.040417054,0.0733334825],[-0.0412987169,0.0725694312],[-0.0420621077,0.0713810363],[-0.0427337695,0.0698523409],[-0.043371275,0.0680773464],[-0.0440229466,0.0661672323],[-0.0447376031,0.0642461745],[-0.0455416788,0.0624102503],[-0.0464382879,0.0607247165],[-0.0474060065,0.0592524351],[-0.048397797,0.0579906541],[-0.0493560527,0.0569235527],[-0.0502032578,0.0560346863],[-0.0508775259,0.0552743117],[-0.051322987,0.0546044155],[-0.0514892243,0.05398351],[-0.0513523665,0.0533802236],[-0.0508990581,0.0527683438],[-0.0501364846,0.0521418417],[-0.0490909571,0.0514995285],[-0.0478082102,0.050859996],[-0.0463716804,0.050278109],[-0.0448616775,0.0498081061],[-0.0433851272,0.04952727],[-0.0420673957,0.0495131099],[-0.0410029728,0.0498354728],[-0.0402739969,0.0505422112],[-0.0399157579,0.0516225436],[-0.039921428,0.0530435709],[-0.040232795,0.0547088664],[-0.0407662796,0.0564894939],[-0.0414125802,0.0582390087],[-0.0420656521,0.0598217236],[-0.0426362598,0.0610991169],[-0.0430455251,0.061970168],[-0.0432611863,0.0623733292],[-0.0432431172,0.0622591887],[-0.0430202662,0.0616456233],[-0.0426253659,0.0605817944],[-0.0421132565,0.0591507565],[-0.0415622807,0.0574726378],[-0.0410586398,0.0556836398],[-0.0406875532,0.0539446961],[-0.0405155771,0.0523937914],[-0.040588337,0.0511504799],[-0.0409074899,0.0503085614],[-0.0414272976,0.0498776547],[-0.0420792198,0.0498581483],[-0.0427617185,0.0502010042],[-0.0433544117,0.0508161974],[-0.0437615103,0.051625796],[-0.0438952835,0.0525402835],[-0.0437051226,0.0534933369],[-0.0431605075,0.0544308646],[-0.0422749302,0.0553124302],[-0.0411036951,0.0561429808],[-0.0397055853,0.0569320139],[-0.0381868091,0.0577028805],[-0.0366571629,0.0584971629],[-0.035212257,0.0593693999],[-0.0339701317,0.0603529888],[-0.0330085765,0.0614835765],[-0.0323991912,0.0627834769],[-0.0321613061,0.0642184489],[-0.0323119887,0.0657844887],[-0.0328135134,0.0674117276],[-0.0336156361,0.0690245646],[-0.034641557,0.0705304855]],"A1013":[[-0.035821865,0.071839365],[-0.0370473444,0.0728355586],[-0.0382597229,0.0734518657],[-0.039401731,0.0736328025],[-0.040417054,0.0733334825],[-0.0412987169,0.0725694312],[-0.0420621077,0.0713810363],[-0.0427337695,0.0698523409],[-0.043371275,0.0680773464],[-0.0440229466,0.0661672323],[-0.0447376031,0.0642461745],[-0.0455416788,0.0624102503],[-0.0464382879,0.0607247165],[-0.0474060065,0.0592524351],[-0.048397797,0.0579906541],[-0.0493560527,0.0569235527],[-0.0502032578,0.0560346863],[-0.0508775259,0.0552743117],[-0.051322987,0.0546044155],[-0.0514892243,0.05398351],[-0.0513523665,0.0533802236],[-0.0508990581,0.0527683438],[-0.0501364846,0.0521418417],[-0.0490909571,0.0514995285],[-0.0478082102,0.050859996],[-0.0463716804,0.050278109],[-0.0448616775,0.0498081061],[-0.0433851272,0.04952727],[-0.0420673957,0.0495131099],[-0.0410029728,0.0498354728],[-0.0402739969,0.0505422112],[-0.0399157579,0.0516225436],[-0.039921428,0.0530435709],[-0.040232795,0.0547088664],[-0.0407662796,0.0564894939],[-0.0414125802,0.0582390087],[-0.0420656521,0.0598217236],[-0.0426362598,0.0610991169],[-0.0430455251,0.061970168],[-0.0432611863,0.0623733292],[-0.0432431172,0.0622591887],[-0.0430202662,0.0616456233],[-0.0426253659,0.0605817944],[-0.0421132565,0.0591507565],[-0.0415622807,0.0574726378],[-0.0410586398,0.0556836398],[-0.0406875532,0.0539446961],[-0.0405155771,0.0523937914],[-0.040588337,0.0511504799],[-0.0409074899,0.0503085614],[-0.0414272976,0.0498776547],[-0.0420792198,0.0498581483],[-0.0427617185,0.0502010042],[-0.0433544117,0.0508161974],[-0.0437615103,0.051625796],[-0.0438952835,0.0525402835],[-0.0437051226,0.0534933369],[-0.0431605075,0.0544308646],[-0.0422749302,0.0553124302],[-0.0411036951,0.0561429808],[-0.0397055853,0.0569320139],[-0.0381868091,0.0577028805],[-0.0366571629,0.0584971629],[-0.035212257,0.0593693999],[-0.0339701317,0.0603529888],[-0.0330085765,0.0614835765],[-0.0323991912,0.0627834769],[-0.0321613061,0.0642184489],[-0.0323119887,0.0657844887],[-0.0328135134,0.0674117276],[-0.0336156361,0.0690245646],[-0.034641557,0.0705304855]],"A1101":[[-0.0196781264,0.0319114597],[-0.0205089742,0.0323295297],[-0.02172625,0.03293625],[-0.0233535508,0.0337446619],[-0.0253325218,0.0347169663],[-0.0276053384,0.0358108939],[-0.0300899683,0.0369594127],[-0.0326695279,0.0380850834],[-0.0352462515,0.0391195848],[-0.0377460842,0.0400183064],[-0.0400668268,0.0407301601],[-0.0421446679,0.0412302235],[-0.0439474602,0.0415146824],[-0.0454305979,0.0415861535],[-0.0466050886,0.0414756442],[-0.0474691779,0.0412091779],[-0.0480460817,0.0408316372],[-0.0483642993,0.0403809659],[-0.0484500241,0.0398922464],[-0.0483408049,0.0394052493],[-0.0480486146,0.0389286146],[-0.0476129827,0.0384890938],[-0.0470312161,0.0380812161],[-0.0463046984,0.0376930318],[-0.0454318752,0.0373113197],[-0.0444007171,0.036904606],[-0.0432011176,0.036449451],[-0.0418186152,0.0359230597],[-0.0402703729,0.0353131507],[-0.0385704162,0.0346287496],[-0.0367355569,0.0338611124],[-0.0348208068,0.0330408068],[-0.0328721349,0.0322015794],[-0.0309372252,0.0313661141],[-0.0291095883,0.0305912549],[-0.0274296646,0.029894109],[-0.0259501977,0.0293068643],[-0.0247004636,0.0288254636],[-0.0236934001,0.0284389557],[-0.0229135382,0.0281207604],[-0.022341535,0.0278320905],[-0.0219369262,0.0275363706],[-0.0216576849,0.0271910182],[-0.0214675182,0.0267830737],[-0.021334747,0.0262986359],[-0.0212406796,0.0257467907],[-0.0211768347,0.0251618347],[-0.0211268267,0.0245584934],[-0.0211047236,0.0239886125],[-0.0210929594,0.0234785149],[-0.021117889,0.023077889],[-0.0211600866,0.0228067532],[-0.021222692,0.0226893587],[-0.0213056371,0.0227423038],[-0.0213979529,0.0229612863],[-0.0214840433,0.0233351544],[-0.0215662652,0.0238673764],[-0.0216260642,0.024524953],[-0.021665765,0.0252874317],[-0.0216611151,0.0261105595],[-0.0216137,0.0269775889],[-0.0215111765,0.0278311765],[-0.0213490151,0.0286451262],[-0.0211207305,0.0293818416],[-0.0208308727,0.0300153171],[-0.0204879411,0.0305251634],[-0.0201107584,0.0309068695],[-0.0197342967,0.0311659634],[-0.0193943742,0.0313265964],[-0.0191481463,0.0314270352],[-0.0190633394,0.0315161172],[-0.0192226152,0.0316615041]],"A1102":[[-0.0196781264,0.0319114597],[-0.0205089742,0.0323295297],[-0.02172625,0.03293625],[-0.0233535508,0.0337446619],[-0.0253325218,0.0347169663],[-0.0276053384,0.0358108939],[-0.0300899683,0.0369594127],[-0.0326695279,0.0380850834],[-0.0352462515,0.0391195848],[-0.0377460842,0.0400183064],[-0.0400668268,0.0407301601],[-0.0421446679,0.0412302235],[-0.0439474602,0.0415146824],[-0.0454305979,0.0415861535],[-0.0466050886,0.0414756442],[-0.0474691779,0.0412091779],[-0.0480460817,0.0408316372],[-0.0483642993,0.0403809659],[-0.0484500241,0.0398922464],[-0.0483408049,0.0394052493],[-0.0480486146,0.0389286146],[-0.0476129827,0.0384890938],[-0.0470312161,0.0380812161],[-0.0463046984,0.0376930318],[-0.0454318752,0.0373113197],[-0.0444007171,0.036904606],[-0.0432011176,0.036449451],[-0.0418186152,0.0359230597],[-0.0402703729,0.0353131507],[-0.0385704162,0.0346287496],[-0.0367355569,0.0338611124],[-0.0348208068,0.0330408068],[-0.0328721349,0.0322015794],[-0.0309372252,0.0313661141],[-0.0291095883,0.0305912549],[-0.0274296646,0.029894109],[-0.0259501977,0.0293068643],[-0.0247004636,0.0288254636],[-0.0236934001,0.0284389557],[-0.0229135382,0.0281207604],[-0.022341535,0.0278320905],[-0.0219369262,0.0275363706],[-0.0216576849,0.0271910182],[-0.0214675182,0.0267830737],[-0.021334747,0.0262986359],[-0.0212406796,0.0257467907],[-0.0211768347,0.0251618347],[-0.0211268267,0.0245584934],[-0.0211047236,0.0239886125],[-0.0210929594,0.0234785149],[-0.021117889,0.023077889],[-0.0211600866,0.0228067532],[-0.021222692,0.0226893587],[-0.0213056371,0.0227423038],[-0.0213979529,0.0229612863],[-0.0214840433,0.0233351544],[-0.0215662652,0.0238673764],[-0.0216260642,0.024524953],[-0.021665765,0.0252874317],[-0.0216611151,0.0261105595],[-0.0216137,0.0269775889],[-0.0215111765,0.0278311765],[-0.0213490151,0.0286451262],[-0.0211207305,0.0293818416],[-0.0208308727,0.0300153171],[-0.0204879411,0.0305251634],[-0.0201107584,0.0309068695],[-0.0197342967,0.0311659634],[-0.0193943742,0.0313265964],[-0.0191481463,0.0314270352],[-0.0190633394,0.0315161172],[-0.0192226152,0.0316615041]],"A1103":[[-0.035821865,0.071839365],[-0.0370473444,0.0728355586],[-0.0382597229,0.0734518657],[-0.039401731,0.0736328025],[-0.040417054,0.0733334825],[-0.0412987169,0.0725694312],[-0.0420621077,0.0713810363],[-0.0427337695,0.0698523409],[-0.043371275,0.0680773464],[-0.0440229466,0.0661672323],[-0.0447376031,0.0642461745],[-0.0455416788,0.0624102503],[-0.0464382879,0.0607247165],[-0.0474060065,0.0592524351],[-0.048397797,0.0579906541],[-0.0493560527,0.0569235527],[-0.0502032578,0.0560346863],[-0.0508775259,0.0552743117],[-0.051322987,0.0546044155],[-0.0514892243,0.05398351],[-0.0513523665,0.0533802236],[-0.0508990581,0.0527683438],[-0.0501364846,0.0521418417],[-0.0490909571,0.0514995285],[-0.0478082102,0.050859996],[-0.0463716804,0.050278109],[-0.0448616775,0.0498081061],[-0.0433851272,0.04952727],[-0.0420673957,0.0495131099],[-0.0410029728,0.0498354728],[-0.0402739969,0.0505422112],[-0.0399157579,0.0516225436],[-0.039921428,0.0530435709],[-0.040232795,0.0547088664],[-0.0407662796,0.0564894939],[-0.0414125802,0.0582390087],[-0.0420656521,0.0598217236],[-0.0426362598,0.0610991169],[-0.0430455251,0.061970168],[-0.0432611863,0.0623733292],[-0.0432431172,0.0622591887],[-0.0430202662,0.0616456233],[-0.0426253659,0.0605817944],[-0.0421132565,0.0591507565],[-0.0415622807,0.0574726378],[-0.0410586398,0.0556836398],[-0.0406875532,0.0539446961],[-0.0405155771,0.0523937914],[-0.040588337,0.0511504799],[-0.0409074899,0.0503085614],[-0.0414272976,0.0498776547],[-0.0420792198,0.0498581483],[-0.0427617185,0.0502010042],[-0.0433544117,0.0508161974],[-0.0437615103,0.051625796],[-0.0438952835,0.0525402835],[-0.0437051226,0.0534933369],[-0.0431605075,0.0544308646],[-0.0422749302,0.0553124302],[-0.0411036951,0.0561429808],[-0.0397055853,0.0569320139],[-0.0381868091,0.0577028805],[-0.0366571629,0.0584971629],[-0.035212257,0.0593693999],[-0.0339701317,0.0603529888],[-0.0330085765,0.0614835765],[-0.0323991912,0.0627834769],[-0.0321613061,0.0642184489],[-0.0323119887,0.0657844887],[-0.0328135134,0.0674117276],[-0.0336156361,0.0690245646],[-0.034641557,0.0705304855]],"A1104":[[-0.035821865,0.071839365],[-0.0370473444,0.0728355586],[-0.0382597229,0.0734518657],[-0.039401731,0.0736328025],[-0.040417054,0.0733334825],[-0.0412987169,0.0725694312],[-0.0420621077,0.0713810363],[-0.0427337695,0.0698523409],[-0.043371275,0.0680773464],[-0.0440229466,0.0661672323],[-0.0447376031,0.0642461745],[-0.0455416788,0.0624102503],[-0.0464382879,0.0607247165],[-0.0474060065,0.0592524351],[-0.048397797,0.0579906541],[-0.0493560527,0.0569235527],[-0.0502032578,0.0560346863],[-0.0508775259,0.0552743117],[-0.051322987,0.0546044155],[-0.0514892243,0.05398351],[-0.0513523665,0.0533802236],[-0.0508990581,0.0527683438],[-0.0501364846,0.0521418417],[-0.0490909571,0.0514995285],[-0.0478082102,0.050859996],[-0.0463716804,0.050278109],[-0.0448616775,0.0498081061],[-0.0433851272,0.04952727],[-0.0420673957,0.0495131099],[-0.0410029728,0.0498354728],[-0.0402739969,0.0505422112],[-0.0399157579,0.0516225436],[-0.039921428,0.0530435709],[-0.040232795,0.0547088664],[-0.0407662796,0.0564894939],[-0.0414125802,0.0582390087],[-0.0420656521,0.0598217236],[-0.0426362598,0.0610991169],[-0.0430455251,0.061970168],[-0.0432611863,0.0623733292],[-0.0432431172,0.0622591887],[-0.0430202662,0.0616456233],[-0.0426253659,0.0605817944],[-0.0421132565,0.0591507565],[-0.0415622807,0.0574726378],[-0.0410586398,0.0556836398],[-0.0406875532,0.0539446961],[-0.0405155771,0.0523937914],[-0.040588337,0.0511504799],[-0.0409074899,0.0503085614],[-0.0414272976,0.0498776547],[-0.0420792198,0.0498581483],[-0.0427617185,0.0502010042],[-0.0433544117,0.0508161974],[-0.0437615103,0.051625796],[-0.0438952835,0.0525402835],[-0.0437051226,0.0534933369],[-0.0431605075,0.0544308646],[-0.0422749302,0.0553124302],[-0.0411036951,0.0561429808],[-0.0397055853,0.0569320139],[-0.0381868091,0.0577028805],[-0.0366571629,0.0584971629],[-0.035212257,0.0593693999],[-0.0339701317,0.0603529888],[-0.0330085765,0.0614835765],[-0.0323991912,0.0627834769],[-0.0321613061,0.0642184489],[-0.0323119887,0.0657844887],[-0.0328135134,0.0674117276],[-0.0336156361,0.0690245646],[-0.034641557,0.0705304855]],"A1105":[[-0.0196781264,0.0319114597],[-0.0205089742,0.0323295297],[-0.02172625,0.03293625],[-0.0233535508,0.0337446619],[-0.0253325218,0.0347169663],[-0.0276053384,0.0358108939],[-0.0300899683,0.0369594127],[-0.0326695279,0.0380850834],[-0.0352462515,0.0391195848],[-0.0377460842,0.0400183064],[-0.0400668268,0.0407301601],[-0.0421446679,0.0412302235],[-0.0439474602,0.0415146824],[-0.0454305979,0.0415861535],[-0.0466050886,0.0414756442],[-0.0474691779,0.0412091779],[-0.0480460817,0.0408316372],[-0.0483642993,0.0403809659],[-0.0484500241,0.0398922464],[-0.0483408049,0.0394052493],[-0.0480486146,0.0389286146],[-0.0476129827,0.0384890938],[-0.0470312161,0.0380812161],[-0.0463046984,0.0376930318],[-0.0454318752,0.0373113197],[-0.0444007171,0.036904606],[-0.0432011176,0.036449451],[-0.0418186152,0.0359230597],[-0.0402703729,0.0353131507],[-0.0385704162,0.0346287496],[-0.0367355569,0.0338611124],[-0.0348208068,0.0330408068],[-0.0328721349,0.0322015794],[-0.0309372252,0.0313661141],[-0.0291095883,0.0305912549],[-0.0274296646,0.029894109],[-0.0259501977,0.0293068643],[-0.0247004636,0.0288254636],[-0.0236934001,0.0284389557],[-0.0229135382,0.0281207604],[-0.022341535,0.0278320905],[-0.0219369262,0.0275363706],[-0.0216576849,0.0271910182],[-0.0214675182,0.0267830737],[-0.021334747,0.0262986359],[-0.0212406796,0.0257467907],[-0.0211768347,0.0251618347],[-0.0211268267,0.0245584934],[-0.0211047236,0.0239886125],[-0.0210929594,0.0234785149],[-0.021117889,0.023077889],[-0.0211600866,0.0228067532],[-0.021222692,0.0226893587],[-0.0213056371,0.0227423038],[-0.0213979529,0.0229612863],[-0.0214840433,0.0233351544],[-0.0215662652,0.0238673764],[-0.0216260642,0.024524953],[-0.021665765,0.0252874317],[-0.0216611151,0.0261105595],[-0.0216137,0.0269775889],[-0.0215111765,0.0278311765],[-0.0213490151,0.0286451262],[-0.0211207305,0.0293818416],[-0.0208308727,0.0300153171],[-0.0204879411,0.0305251634],[-0.0201107584,0.0309068695],[-0.0197342967,0.0311659634],[-0.0193943742,0.0313265964],[-0.0191481463,0.0314270352],[-0.0190633394,0.0315161172],[-0.0192226152,0.0316615041]],"A1106":[[-0.035821865,0.071839365],[-0.0370473444,0.0728355586],[-0.0382597229,0.0734518657],[-0.039401731,0.0736328025],[-0.040417054,0.0733334825],[-0.0412987169,0.0725694312],[-0.0420621077,0.0713810363],[-0.0427337695,0.0698523409],[-0.043371275,0.0680773464],[-0.0440229466,0.0661672323],[-0.0447376031,0.0642461745],[-0.0455416788,0.0624102503],[-0.0464382879,0.0607247165],[-0.0474060065,0.0592524351],[-0.048397797,0.0579906541],[-0.0493560527,0.0569235527],[-0.0502032578,0.0560346863],[-0.0508775259,0.0552743117],[-0.051322987,0.0546044155],[-0.0514892243,0.05398351],[-0.0513523665,0.0533802236],[-0.0508990581,0.0527683438],[-0.0501364846,0.0521418417],[-0.0490909571,0.0514995285],[-0.0478082102,0.050859996],[-0.0463716804,0.050278109],[-0.0448616775,0.0498081061],[-0.0433851272,0.04952727],[-0.0420673957,0.0495131099],[-0.0410029728,0.0498354728],[-0.0402739969,0.0505422112],[-0.0399157579,0.0516225436],[-0.039921428,0.0530435709],[-0.040232795,0.0547088664],[-0.0407662796,0.0564894939],[-0.0414125802,0.0582390087],[-0.0420656521,0.0598217236],[-0.0426362598,0.0610991169],[-0.0430455251,0.061970168],[-0.0432611863,0.0623733292],[-0.0432431172,0.0622591887],[-0.0430202662,0.0616456233],[-0.0426253659,0.0605817944],[-0.0421132565,0.0591507565],[-0.0415622807,0.0574726378],[-0.0410586398,0.0556836398],[-0.0406875532,0.0539446961],[-0.0405155771,0.0523937914],[-0.040588337,0.0511504799],[-0.0409074899,0.0503085614],[-0.0414272976,0.0498776547],[-0.0420792198,0.0498581483],[-0.0427617185,0.0502010042],[-0.0433544117,0.0508161974],[-0.0437615103,0.051625796],[-0.0438952835,0.0525402835],[-0.0437051226,0.0534933369],[-0.0431605075,0.0544308646],[-0.0422749302,0.0553124302],[-0.0411036951,0.0561429808],[-0.0397055853,0.0569320139],[-0.0381868091,0.0577028805],[-0.0366571629,0.0584971629],[-0.035212257,0.0593693999],[-0.0339701317,0.0603529888],[-0.0330085765,0.0614835765],[-0.0323991912,0.0627834769],[-0.0321613061,0.0642184489],[-0.0323119887,0.0657844887],[-0.0328135134,0.0674117276],[-0.0336156361,0.0690245646],[-0.034641557,0.0705304855]],"A1107":[[-0.0196781264,0.0319114597],[-0.0205089742,0.0323295297],[-0.02172625,0.03293625],[-0.0233535508,0.0337446619],[-0.0253325218,0.0347169663],[-0.0276053384,0.0358108939],[-0.0300899683,0.0369594127],[-0.0326695279,0.0380850834],[-0.0352462515,0.0391195848],[-0.0377460842,0.0400183064],[-0.0400668268,0.0407301601],[-0.0421446679,0.0412302235],[-0.0439474602,0.0415146824],[-0.0454305979,0.0415861535],[-0.0466050886,0.0414756442],[-0.0474691779,0.0412091779],[-0.0480460817,0.0408316372],[-0.0483642993,0.0403809659],[-0.0484500241,0.0398922464],[-0.0483408049,0.0394052493],[-0.0480486146,0.0389286146],[-0.0476129827,0.0384890938],[-0.0470312161,0.0380812161],[-0.0463046984,0.0376930318],[-0.0454318752,0.0373113197],[-0.0444007171,0.036904606],[-0.0432011176,0.036449451],[-0.0418186152,0.0359230597],[-0.0402703729,0.0353131507],[-0.0385704162,0.0346287496],[-0.0367355569,0.0338611124],[-0.0348208068,0.0330408068],[-0.0328721349,0.0322015794],[-0.0309372252,0.0313661141],[-0.0291095883,0.0305912549],[-0.0274296646,0.029894109],[-0.0259501977,0.0293068643],[-0.0247004636,0.0288254636],[-0.0236934001,0.0284389557],[-0.0229135382,0.0281207604],[-0.022341535,0.0278320905],[-0.0219369262,0.0275363706],[-0.0216576849,0.0271910182],[-0.0214675182,0.0267830737],[-0.021334747,0.0262986359],[-0.0212406796,0.0257467907],[-0.0211768347,0.0251618347],[-0.0211268267,0.0245584934],[-0.0211047236,0.0239886125],[-0.0210929594,0.0234785149],[-0.021117889,0.023077889],[-0.0211600866,0.0228067532],[-0.021222692,0.0226893587],[-0.0213056371,0.0227423038],[-0.0213979529,0.0229612863],[-0.0214840433,0.0233351544],[-0.0215662652,0.0238673764],[-0.0216260642,0.024524953],[-0.021665765,0.0252874317],[-0.0216611151,0.0261105595],[-0.0216137,0.0269775889],[-0.0215111765,0.0278311765],[-0.0213490151,0.0286451262],[-0.0211207305,0.0293818416],[-0.0208308727,0.0300153171],[-0.0204879411,0.0305251634],[-0.0201107584,0.0309068695],[-0.0197342967,0.0311659634],[-0.0193943742,0.0313265964],[-0.0191481463,0.0314270352],[-0.0190633394,0.0315161172],[-0.0192226152,0.0316615041]],"A1108":[[-0.0196781264,0.0319114597],[-0.0205089742,0.0323295297],[-0.02172625,0.03293625],[-0.0233535508,0.0337446619],[-0.0253325218,0.0347169663],[-0.0276053384,0.0358108939],[-0.0300899683,0.0369594127],[-0.0326695279,0.0380850834],[-0.0352462515,0.0391195848],[-0.0377460842,0.0400183064],[-0.0400668268,0.0407301601],[-0.0421446679,0.0412302235],[-0.0439474602,0.0415146824],[-0.0454305979,0.0415861535],[-0.0466050886,0.0414756442],[-0.0474691779,0.0412091779],[-0.0480460817,0.0408316372],[-0.0483642993,0.0403809659],[-0.0484500241,0.0398922464],[-0.0483408049,0.0394052493],[-0.0480486146,0.0389286146],[-0.0476129827,0.0384890938],[-0.0470312161,0.0380812161],[-0.0463046984,0.0376930318],[-0.0454318752,0.0373113197],[-0.0444007171,0.036904606],[-0.0432011176,0.036449451],[-0.0418186152,0.0359230597],[-0.0402703729,0.0353131507],[-0.0385704162,0.0346287496],[-0.0367355569,0.0338611124],[-0.0348208068,0.0330408068],[-0.0328721349,0.0322015794],[-0.0309372252,0.0313661141],[-0.0291095883,0.0305912549],[-0.0274296646,0.029894109],[-0.0259501977,0.0293068643],[-0.0247004636,0.0288254636],[-0.0236934001,0.0284389557],[-0.0229135382,0.0281207604],[-0.022341535,0.0278320905],[-0.0219369262,0.0275363706],[-0.0216576849,0.0271910182],[-0.0214675182,0.0267830737],[-0.021334747,0.0262986359],[-0.0212406796,0.0257467907],[-0.0211768347,0.0251618347],[-0.0211268267,0.0245584934],[-0.0211047236,0.0239886125],[-0.0210929594,0.0234785149],[-0.021117889,0.023077889],[-0.0211600866,0.0228067532],[-0.021222692,0.0226893587],[-0.0213056371,0.0227423038],[-0.0213979529,0.0229612863],[-0.0214840433,0.0233351544],[-0.0215662652,0.0238673764],[-0.0216260642,0.024524953],[-0.021665765,0.0252874317],[-0.0216611151,0.0261105595],[-0.0216137,0.0269775889],[-0.0215111765,0.0278311765],[-0.0213490151,0.0286451262],[-0.0211207305,0.0293818416],[-0.0208308727,0.0300153171],[-0.0204879411,0.0305251634],[-0.0201107584,0.0309068695],[-0.0197342967,0.0311659634],[-0.0193943742,0.0313265964],[-0.0191481463,0.0314270352],[-0.0190633394,0.0315161172],[-0.0192226152,0.0316615041]],"A1109":[[-0.0196781264,0.0319114597],[-0.0205089742,0.0323295297],[-0.02172625,0.03293625],[-0.0233535508,0.0337446619],[-0.0253325218,0.0347169663],[-0.0276053384,0.0358108939],[-0.0300899683,0.0369594127],[-0.0326695279,0.0380850834],[-0.0352462515,0.0391195848],[-0.0377460842,0.0400183064],[-0.0400668268,0.0407301601],[-0.0421446679,0.0412302235],[-0.0439474602,0.0415146824],[-0.0454305979,0.0415861535],[-0.0466050886,0.0414756442],[-0.0474691779,0.0412091779],[-0.0480460817,0.0408316372],[-0.0483642993,0.0403809659],[-0.0484500241,0.0398922464],[-0.0483408049,0.0394052493],[-0.0480486146,0.0389286146],[-0.0476129827,0.0384890938],[-0.0470312161,0.0380812161],[-0.0463046984,0.0376930318],[-0.0454318752,0.0373113197],[-0.0444007171,0.036904606],[-0.0432011176,0.036449451],[-0.0418186152,0.0359230597],[-0.0402703729,0.0353131507],[-0.0385704162,0.0346287496],[-0.0367355569,0.0338611124],[-0.0348208068,0.0330408068],[-0.0328721349,0.0322015794],[-0.0309372252,0.0313661141],[-0.0291095883,0.0305912549],[-0.0274296646,0.029894109],[-0.0259501977,0.0293068643],[-0.0247004636,0.0288254636],[-0.0236934001,0.0284389557],[-0.0229135382,0.0281207604],[-0.022341535,0.0278320905],[-0.0219369262,0.0275363706],[-0.0216576849,0.0271910182],[-0.0214675182,0.0267830737],[-0.021334747,0.0262986359],[-0.0212406796,0.0257467907],[-0.0211768347,0.0251618347],[-0.0211268267,0.0245584934],[-0.0211047236,0.0239886125],[-0.0210929594,0.0234785149],[-0.021117889,0.023077889],[-0.0211600866,0.0228067532],[-0.021222692,0.0226893587],[-0.0213056371,0.0227423038],[-0.0213979529,0.0229612863],[-0.0214840433,0.0233351544],[-0.0215662652,0.0238673764],[-0.0216260642,0.024524953],[-0.021665765,0.0252874317],[-0.0216611151,0.0261105595],[-0.0216137,0.0269775889],[-0.0215111765,0.0278311765],[-0.0213490151,0.0286451262],[-0.0211207305,0.0293818416],[-0.0208308727,0.0300153171],[-0.0204879411,0.0305251634],[-0.0201107584,0.0309068695],[-0.0197342967,0.0311659634],[-0.0193943742,0.0313265964],[-0.0191481463,0.0314270352],[-0.0190633394,0.0315161172],[-0.0192226152,0.0316615041]],"A1110":[[-0.0196781264,0.0319114597],[-0.0205089742,0.0323295297],[-0.02172625,0.03293625],[-0.0233535508,0.0337446619],[-0.0253325218,0.0347169663],[-0.0276053384,0.0358108939],[-0.0300899683,0.0369594127],[-0.0326695279,0.0380850834],[-0.0352462515,0.0391195848],[-0.0377460842,0.0400183064],[-0.0400668268,0.0407301601],[-0.0421446679,0.0412302235],[-0.0439474602,0.0415146824],[-0.0454305979,0.0415861535],[-0.0466050886,0.0414756442],[-0.0474691779,0.0412091779],[-0.0480460817,0.0408316372],[-0.0483642993,0.0403809659],[-0.0484500241,0.0398922464],[-0.0483408049,0.0394052493],[-0.0480486146,0.0389286146],[-0.0476129827,0.0384890938],[-0.0470312161,0.0380812161],[-0.0463046984,0.0376930318],[-0.0454318752,0.0373113197],[-0.0444007171,0.036904606],[-0.0432011176,0.036449451],[-0.0418186152,0.0359230597],[-0.0402703729,0.0353131507],[-0.0385704162,0.0346287496],[-0.0367355569,0.0338611124],[-0.0348208068,0.0330408068],[-0.0328721349,0.0322015794],[-0.0309372252,0.0313661141],[-0.0291095883,0.0305912549],[-0.0274296646,0.029894109],[-0.0259501977,0.0293068643],[-0.0247004636,0.0288254636],[-0.0236934001,0.0284389557],[-0.0229135382,0.0281207604],[-0.022341535,0.0278320905],[-0.0219369262,0.0275363706],[-0.0216576849,0.0271910182],[-0.0214675182,0.0267830737],[-0.021334747,0.0262986359],[-0.0212406796,0.0257467907],[-0.0211768347,0.0251618347],[-0.0211268267,0.0245584934],[-0.0211047236,0.0239886125],[-0.0210929594,0.0234785149],[-0.021117889,0.023077889],[-0.0211600866,0.0228067532],[-0.021222692,0.0226893587],[-0.0213056371,0.0227423038],[-0.0213979529,0.0229612863],[-0.0214840433,0.0233351544],[-0.0215662652,0.0238673764],[-0.0216260642,0.024524953],[-0.021665765,0.0252874317],[-0.0216611151,0.0261105595],[-0.0216137,0.0269775889],[-0.0215111765,0.0278311765],[-0.0213490151,0.0286451262],[-0.0211207305,0.0293818416],[-0.0208308727,0.0300153171],[-0.0204879411,0.0305251634],[-0.0201107584,0.0309068695],[-0.0197342967,0.0311659634],[-0.0193943742,0.0313265964],[-0.0191481463,0.0314270352],[-0.0190633394,0.0315161172],[-0.0192226152,0.0316615041]],"A1111":[[-0.0196781264,0.0319114597],[-0.0205089742,0.0323295297],[-0.02172625,0.03293625],[-0.0233535508,0.0337446619],[-0.0253325218,0.0347169663],[-0.0276053384,0.0358108939],[-0.0300899683,0.0369594127],[-0.0326695279,0.0380850834],[-0.0352462515,0.0391195848],[-0.0377460842,0.0400183064],[-0.0400668268,0.0407301601],[-0.0421446679,0.0412302235],[-0.0439474602,0.0415146824],[-0.0454305979,0.0415861535],[-0.0466050886,0.0414756442],[-0.0474691779,0.0412091779],[-0.0480460817,0.0408316372],[-0.0483642993,0.0403809659],[-0.0484500241,0.0398922464],[-0.0483408049,0.0394052493],[-0.0480486146,0.0389286146],[-0.0476129827,0.0384890938],[-0.0470312161,0.0380812161],[-0.0463046984,0.0376930318],[-0.0454318752,0.0373113197],[-0.0444007171,0.036904606],[-0.0432011176,0.036449451],[-0.0418186152,0.0359230597],[-0.0402703729,0.0353131507],[-0.0385704162,0.0346287496],[-0.0367355569,0.0338611124],[-0.0348208068,0.0330408068],[-0.0328721349,0.0322015794],[-0.0309372252,0.0313661141],[-0.0291095883,0.0305912549],[-0.0274296646,0.029894109],[-0.0259501977,0.0293068643],[-0.0247004636,0.0288254636],[-0.0236934001,0.0284389557],[-0.0229135382,0.0281207604],[-0.022341535,0.0278320905],[-0.0219369262,0.0275363706],[-0.0216576849,0.0271910182],[-0.0214675182,0.0267830737],[-0.021334747,0.0262986359],[-0.0212406796,0.0257467907],[-0.0211768347,0.0251618347],[-0.0211268267,0.0245584934],[-0.0211047236,0.0239886125],[-0.0210929594,0.0234785149],[-0.021117889,0.023077889],[-0.0211600866,0.0228067532],[-0.021222692,0.0226893587],[-0.0213056371,0.0227423038],[-0.0213979529,0.0229612863],[-0.0214840433,0.0233351544],[-0.0215662652,0.0238673764],[-0.0216260642,0.024524953],[-0.021665765,0.0252874317],[-0.0216611151,0.0261105595],[-0.0216137,0.0269775889],[-0.0215111765,0.0278311765],[-0.0213490151,0.0286451262],[-0.0211207305,0.0293818416],[-0.0208308727,0.0300153171],[-0.0204879411,0.0305251634],[-0.0201107584,0.0309068695],[-0.0197342967,0.0311659634],[-0.0193943742,0.0313265964],[-0.0191481463,0.0314270352],[-0.0190633394,0.0315161172],[-0.0192226152,0.0316615041]],"A1112":[[-0.0196781264,0.0319114597],[-0.0205089742,0.0323295297],[-0.02172625,0.03293625],[-0.0233535508,0.0337446619],[-0.0253325218,0.0347169663],[-0.0276053384,0.0358108939],[-0.0300899683,0.0369594127],[-0.0326695279,0.0380850834],[-0.0352462515,0.0391195848],[-0.0377460842,0.0400183064],[-0.0400668268,0.0407301601],[-0.0421446679,0.0412302235],[-0.0439474602,0.0415146824],[-0.0454305979,0.0415861535],[-0.0466050886,0.0414756442],[-0.0474691779,0.0412091779],[-0.0480460817,0.0408316372],[-0.0483642993,0.0403809659],[-0.0484500241,0.0398922464],[-0.0483408049,0.0394052493],[-0.0480486146,0.0389286146],[-0.0476129827,0.0384890938],[-0.0470312161,0.0380812161],[-0.0463046984,0.0376930318],[-0.0454318752,0.0373113197],[-0.0444007171,0.036904606],[-0.0432011176,0.036449451],[-0.0418186152,0.0359230597],[-0.0402703729,0.0353131507],[-0.0385704162,0.0346287496],[-0.0367355569,0.0338611124],[-0.0348208068,0.0330408068],[-0.0328721349,0.0322015794],[-0.0309372252,0.0313661141],[-0.0291095883,0.0305912549],[-0.0274296646,0.029894109],[-0.0259501977,0.0293068643],[-0.0247004636,0.0288254636],[-0.0236934001,0.0284389557],[-0.0229135382,0.0281207604],[-0.022341535,0.0278320905],[-0.0219369262,0.0275363706],[-0.0216576849,0.0271910182],[-0.0214675182,0.0267830737],[-0.021334747,0.0262986359],[-0.0212406796,0.0257467907],[-0.0211768347,0.0251618347],[-0.0211268267,0.0245584934],[-0.0211047236,0.0239886125],[-0.0210929594,0.0234785149],[-0.021117889,0.023077889],[-0.0211600866,0.0228067532],[-0.021222692,0.0226893587],[-0.0213056371,0.0227423038],[-0.0213979529,0.0229612863],[-0.0214840433,0.0233351544],[-0.0215662652,0.0238673764],[-0.0216260642,0.024524953],[-0.021665765,0.0252874317],[-0.0216611151,0.0261105595],[-0.0216137,0.0269775889],[-0.0215111765,0.0278311765],[-0.0213490151,0.0286451262],[-0.0211207305,0.0293818416],[-0.0208308727,0.0300153171],[-0.0204879411,0.0305251634],[-0.0201107584,0.0309068695],[-0.0197342967,0.0311659634],[-0.0193943742,0.0313265964],[-0.0191481463,0.0314270352],[-0.0190633394,0.0315161172],[-0.0192226152,0.0316615041]],"A1113":[[-0.0196781264,0.0319114597],[-0.0205089742,0.0323295297],[-0.02172625,0.03293625],[-0.0233535508,0.0337446619],[-0.0253325218,0.0347169663],[-0.0276053384,0.0358108939],[-0.0300899683,0.0369594127],[-0.0326695279,0.0380850834],[-0.0352462515,0.0391195848],[-0.0377460842,0.0400183064],[-0.0400668268,0.0407301601],[-0.0421446679,0.0412302235],[-0.0439474602,0.0415146824],[-0.0454305979,0.0415861535],[-0.0466050886,0.0414756442],[-0.0474691779,0.0412091779],[-0.0480460817,0.0408316372],[-0.0483642993,0.0403809659],[-0.0484500241,0.0398922464],[-0.0483408049,0.0394052493],[-0.0480486146,0.0389286146],[-0.0476129827,0.0384890938],[-0.0470312161,0.0380812161],[-0.0463046984,0.0376930318],[-0.0454318752,0.0373113197],[-0.0444007171,0.036904606],[-0.0432011176,0.036449451],[-0.0418186152,0.0359230597],[-0.0402703729,0.0353131507],[-0.0385704162,0.0346287496],[-0.0367355569,0.0338611124],[-0.0348208068,0.0330408068],[-0.0328721349,0.0322015794],[-0.0309372252,0.0313661141],[-0.0291095883,0.0305912549],[-0.0274296646,0.029894109],[-0.0259501977,0.0293068643],[-0.0247004636,0.0288254636],[-0.0236934001,0.0284389557],[-0.0229135382,0.0281207604],[-0.022341535,0.0278320905],[-0.0219369262,0.0275363706],[-0.0216576849,0.0271910182],[-0.0214675182,0.0267830737],[-0.021334747,0.0262986359],[-0.0212406796,0.0257467907],[-0.0211768347,0.0251618347],[-0.0211268267,0.0245584934],[-0.0211047236,0.0239886125],[-0.0210929594,0.0234785149],[-0.021117889,0.023077889],[-0.0211600866,0.0228067532],[-0.021222692,0.0226893587],[-0.0213056371,0.0227423038],[-0.0213979529,0.0229612863],[-0.0214840433,0.0233351544],[-0.0215662652,0.0238673764],[-0.0216260642,0.024524953],[-0.021665765,0.0252874317],[-0.0216611151,0.0261105595],[-0.0216137,0.0269775889],[-0.0215111765,0.0278311765],[-0.0213490151,0.0286451262],[-0.0211207305,0.0293818416],[-0.0208308727,0.0300153171],[-0.0204879411,0.0305251634],[-0.0201107584,0.0309068695],[-0.0197342967,0.0311659634],[-0.0193943742,0.0313265964],[-0.0191481463,0.0314270352],[-0.0190633394,0.0315161172],[-0.0192226152,0.0316615041]],"A1114":[[-0.0196781264,0.0319114597],[-0.0205089742,0.0323295297],[-0.02172625,0.03293625],[-0.0233535508,0.0337446619],[-0.0253325218,0.0347169663],[-0.0276053384,0.0358108939],[-0.0300899683,0.0369594127],[-0.0326695279,0.0380850834],[-0.0352462515,0.0391195848],[-0.0377460842,0.0400183064],[-0.0400668268,0.0407301601],[-0.0421446679,0.0412302235],[-0.0439474602,0.0415146824],[-0.0454305979,0.0415861535],[-0.0466050886,0.0414756442],[-0.0474691779,0.0412091779],[-0.0480460817,0.0408316372],[-0.0483642993,0.0403809659],[-0.0484500241,0.0398922464],[-0.0483408049,0.0394052493],[-0.0480486146,0.0389286146],[-0.0476129827,0.0384890938],[-0.0470312161,0.0380812161],[-0.0463046984,0.0376930318],[-0.0454318752,0.0373113197],[-0.0444007171,0.036904606],[-0.0432011176,0.036449451],[-0.0418186152,0.0359230597],[-0.0402703729,0.0353131507],[-0.0385704162,0.0346287496],[-0.0367355569,0.0338611124],[-0.0348208068,0.0330408068],[-0.0328721349,0.0322015794],[-0.0309372252,0.0313661141],[-0.0291095883,0.0305912549],[-0.0274296646,0.029894109],[-0.0259501977,0.0293068643],[-0.0247004636,0.0288254636],[-0.0236934001,0.0284389557],[-0.0229135382,0.0281207604],[-0.022341535,0.0278320905],[-0.0219369262,0.0275363706],[-0.0216576849,0.0271910182],[-0.0214675182,0.0267830737],[-0.021334747,0.0262986359],[-0.0212406796,0.0257467907],[-0.0211768347,0.0251618347],[-0.0211268267,0.0245584934],[-0.0211047236,0.0239886125],[-0.0210929594,0.0234785149],[-0.021117889,0.023077889],[-0.0211600866,0.0228067532],[-0.021222692,0.0226893587],[-0.0213056371,0.0227423038],[-0.0213979529,0.0229612863],[-0.0214840433,0.0233351544],[-0.0215662652,0.0238673764],[-0.0216260642,0.024524953],[-0.021665765,0.0252874317],[-0.0216611151,0.0261105595],[-0.0216137,0.0269775889],[-0.0215111765,0.0278311765],[-0.0213490151,0.0286451262],[-0.0211207305,0.0293818416],[-0.0208308727,0.0300153171],[-0.0204879411,0.0305251634],[-0.0201107584,0.0309068695],[-0.0197342967,0.0311659634],[-0.0193943742,0.0313265964],[-0.0191481463,0.0314270352],[-0.0190633394,0.0315161172],[-0.0192226152,0.0316615041]],"A1115":[[-0.0196781264,0.0319114597],[-0.0205089742,0.0323295297],[-0.02172625,0.03293625],[-0.0233535508,0.0337446619],[-0.0253325218,0.0347169663],[-0.0276053384,0.0358108939],[-0.0300899683,0.0369594127],[-0.0326695279,0.0380850834],[-0.0352462515,0.0391195848],[-0.0377460842,0.0400183064],[-0.0400668268,0.0407301601],[-0.0421446679,0.0412302235],[-0.0439474602,0.0415146824],[-0.0454305979,0.0415861535],[-0.0466050886,0.0414756442],[-0.0474691779,0.0412091779],[-0.0480460817,0.0408316372],[-0.0483642993,0.0403809659],[-0.0484500241,0.0398922464],[-0.0483408049,0.0394052493],[-0.0480486146,0.0389286146],[-0.0476129827,0.0384890938],[-0.0470312161,0.0380812161],[-0.0463046984,0.0376930318],[-0.0454318752,0.0373113197],[-0.0444007171,0.036904606],[-0.0432011176,0.036449451],[-0.0418186152,0.0359230597],[-0.0402703729,0.0353131507],[-0.0385704162,0.0346287496],[-0.0367355569,0.0338611124],[-0.0348208068,0.0330408068],[-0.0328721349,0.0322015794],[-0.0309372252,0.0313661141],[-0.0291095883,0.0305912549],[-0.0274296646,0.029894109],[-0.0259501977,0.0293068643],[-0.0247004636,0.0288254636],[-0.0236934001,0.0284389557],[-0.0229135382,0.0281207604],[-0.022341535,0.0278320905],[-0.0219369262,0.0275363706],[-0.0216576849,0.0271910182],[-0.0214675182,0.0267830737],[-0.021334747,0.0262986359],[-0.0212406796,0.0257467907],[-0.0211768347,0.0251618347],[-0.0211268267,0.0245584934],[-0.0211047236,0.0239886125],[-0.0210929594,0.0234785149],[-0.021117889,0.023077889],[-0.0211600866,0.0228067532],[-0.021222692,0.0226893587],[-0.0213056371,0.0227423038],[-0.0213979529,0.0229612863],[-0.0214840433,0.0233351544],[-0.0215662652,0.0238673764],[-0.0216260642,0.024524953],[-0.021665765,0.0252874317],[-0.0216611151,0.0261105595],[-0.0216137,0.0269775889],[-0.0215111765,0.0278311765],[-0.0213490151,0.0286451262],[-0.0211207305,0.0293818416],[-0.0208308727,0.0300153171],[-0.0204879411,0.0305251634],[-0.0201107584,0.0309068695],[-0.0197342967,0.0311659634],[-0.0193943742,0.0313265964],[-0.0191481463,0.0314270352],[-0.0190633394,0.0315161172],[-0.0192226152,0.0316615041]],"A1116":[[-0.035821865,0.071839365],[-0.0370473444,0.0728355586],[-0.0382597229,0.0734518657],[-0.039401731,0.0736328025],[-0.040417054,0.0733334825],[-0.0412987169,0.0725694312],[-0.0420621077,0.0713810363],[-0.0427337695,0.0698523409],[-0.043371275,0.0680773464],[-0.0440229466,0.0661672323],[-0.0447376031,0.0642461745],[-0.0455416788,0.0624102503],[-0.0464382879,0.0607247165],[-0.0474060065,0.0592524351],[-0.048397797,0.0579906541],[-0.0493560527,0.0569235527],[-0.0502032578,0.0560346863],[-0.0508775259,0.0552743117],[-0.051322987,0.0546044155],[-0.0514892243,0.05398351],[-0.0513523665,0.0533802236],[-0.0508990581,0.0527683438],[-0.0501364846,0.0521418417],[-0.0490909571,0.0514995285],[-0.0478082102,0.050859996],[-0.0463716804,0.050278109],[-0.0448616775,0.0498081061],[-0.0433851272,0.04952727],[-0.0420673957,0.0495131099],[-0.0410029728,0.0498354728],[-0.0402739969,0.0505422112],[-0.0399157579,0.0516225436],[-0.039921428,0.0530435709],[-0.040232795,0.0547088664],[-0.0407662796,0.0564894939],[-0.0414125802,0.0582390087],[-0.0420656521,0.0598217236],[-0.0426362598,0.0610991169],[-0.0430455251,0.061970168],[-0.0432611863,0.0623733292],[-0.0432431172,0.0622591887],[-0.0430202662,0.0616456233],[-0.0426253659,0.0605817944],[-0.0421132565,0.0591507565],[-0.0415622807,0.0574726378],[-0.0410586398,0.0556836398],[-0.0406875532,0.0539446961],[-0.0405155771,0.0523937914],[-0.040588337,0.0511504799],[-0.0409074899,0.0503085614],[-0.0414272976,0.0498776547],[-0.0420792198,0.0498581483],[-0.0427617185,0.0502010042],[-0.0433544117,0.0508161974],[-0.0437615103,0.051625796],[-0.0438952835,0.0525402835],[-0.0437051226,0.0534933369],[-0.0431605075,0.0544308646],[-0.0422749302,0.0553124302],[-0.0411036951,0.0561429808],[-0.0397055853,0.0569320139],[-0.0381868091,0.0577028805],[-0.0366571629,0.0584971629],[-0.035212257,0.0593693999],[-0.0339701317,0.0603529888],[-0.0330085765,0.0614835765],[-0.0323991912,0.0627834769],[-0.0321613061,0.0642184489],[-0.0323119887,0.0657844887],[-0.0328135134,0.0674117276],[-0.0336156361,0.0690245646],[-0.034641557,0.0705304855]],"A1117":[[-0.035821865,0.071839365],[-0.0370473444,0.0728355586],[-0.0382597229,0.0734518657],[-0.039401731,0.0736328025],[-0.040417054,0.0733334825],[-0.0412987169,0.0725694312],[-0.0420621077,0.0713810363],[-0.0427337695,0.0698523409],[-0.043371275,0.0680773464],[-0.0440229466,0.0661672323],[-0.0447376031,0.0642461745],[-0.0455416788,0.0624102503],[-0.0464382879,0.0607247165],[-0.0474060065,0.0592524351],[-0.048397797,0.0579906541],[-0.0493560527,0.0569235527],[-0.0502032578,0.0560346863],[-0.0508775259,0.0552743117],[-0.051322987,0.0546044155],[-0.0514892243,0.05398351],[-0.0513523665,0.0533802236],[-0.0508990581,0.0527683438],[-0.0501364846,0.0521418417],[-0.0490909571,0.0514995285],[-0.0478082102,0.050859996],[-0.0463716804,0.050278109],[-0.0448616775,0.0498081061],[-0.0433851272,0.04952727],[-0.0420673957,0.0495131099],[-0.0410029728,0.0498354728],[-0.0402739969,0.0505422112],[-0.0399157579,0.0516225436],[-0.039921428,0.0530435709],[-0.040232795,0.0547088664],[-0.0407662796,0.0564894939],[-0.0414125802,0.0582390087],[-0.0420656521,0.0598217236],[-0.0426362598,0.0610991169],[-0.0430455251,0.061970168],[-0.0432611863,0.0623733292],[-0.0432431172,0.0622591887],[-0.0430202662,0.0616456233],[-0.0426253659,0.0605817944],[-0.0421132565,0.0591507565],[-0.0415622807,0.0574726378],[-0.0410586398,0.0556836398],[-0.0406875532,0.0539446961],[-0.0405155771,0.0523937914],[-0.040588337,0.0511504799],[-0.0409074899,0.0503085614],[-0.0414272976,0.0498776547],[-0.0420792198,0.0498581483],[-0.0427617185,0.0502010042],[-0.0433544117,0.0508161974],[-0.0437615103,0.051625796],[-0.0438952835,0.0525402835],[-0.0437051226,0.0534933369],[-0.0431605075,0.0544308646],[-0.0422749302,0.0553124302],[-0.0411036951,0.0561429808],[-0.0397055853,0.0569320139],[-0.0381868091,0.0577028805],[-0.0366571629,0.0584971629],[-0.035212257,0.0593693999],[-0.0339701317,0.0603529888],[-0.0330085765,0.0614835765],[-0.0323991912,0.0627834769],[-0.0321613061,0.0642184489],[-0.0323119887,0.0657844887],[-0.0328135134,0.0674117276],[-0.0336156361,0.0690245646],[-0.034641557,0.0705304855]],"A1118":[[-0.0196781264,0.0319114597],[-0.0205089742,0.0323295297],[-0.02172625,0.03293625],[-0.0233535508,0.0337446619],[-0.0253325218,0.0347169663],[-0.0276053384,0.0358108939],[-0.0300899683,0.0369594127],[-0.0326695279,0.0380850834],[-0.0352462515,0.0391195848],[-0.0377460842,0.0400183064],[-0.0400668268,0.0407301601],[-0.0421446679,0.0412302235],[-0.0439474602,0.0415146824],[-0.0454305979,0.0415861535],[-0.0466050886,0.0414756442],[-0.0474691779,0.0412091779],[-0.0480460817,0.0408316372],[-0.0483642993,0.0403809659],[-0.0484500241,0.0398922464],[-0.0483408049,0.0394052493],[-0.0480486146,0.0389286146],[-0.0476129827,0.0384890938],[-0.0470312161,0.0380812161],[-0.0463046984,0.0376930318],[-0.0454318752,0.0373113197],[-0.0444007171,0.036904606],[-0.0432011176,0.036449451],[-0.0418186152,0.0359230597],[-0.0402703729,0.0353131507],[-0.0385704162,0.0346287496],[-0.0367355569,0.0338611124],[-0.0348208068,0.0330408068],[-0.0328721349,0.0322015794],[-0.0309372252,0.0313661141],[-0.0291095883,0.0305912549],[-0.0274296646,0.029894109],[-0.0259501977,0.0293068643],[-0.0247004636,0.0288254636],[-0.0236934001,0.0284389557],[-0.0229135382,0.0281207604],[-0.022341535,0.0278320905],[-0.0219369262,0.0275363706],[-0.0216576849,0.0271910182],[-0.0214675182,0.0267830737],[-0.021334747,0.0262986359],[-0.0212406796,0.0257467907],[-0.0211768347,0.0251618347],[-0.0211268267,0.0245584934],[-0.0211047236,0.0239886125],[-0.0210929594,0.0234785149],[-0.021117889,0.023077889],[-0.0211600866,0.0228067532],[-0.021222692,0.0226893587],[-0.0213056371,0.0227423038],[-0.0213979529,0.0229612863],[-0.0214840433,0.0233351544],[-0.0215662652,0.0238673764],[-0.0216260642,0.024524953],[-0.021665765,0.0252874317],[-0.0216611151,0.0261105595],[-0.0216137,0.0269775889],[-0.0215111765,0.0278311765],[-0.0213490151,0.0286451262],[-0.0211207305,0.0293818416],[-0.0208308727,0.0300153171],[-0.0204879411,0.0305251634],[-0.0201107584,0.0309068695],[-0.0197342967,0.0311659634],[-0.0193943742,0.0313265964],[-0.0191481463,0.0314270352],[-0.0190633394,0.0315161172],[-0.0192226152,0.0316615041]],"A1119":[[-0.035821865,0.071839365],[-0.0370473444,0.0728355586],[-0.0382597229,0.0734518657],[-0.039401731,0.0736328025],[-0.040417054,0.0733334825],[-0.0412987169,0.0725694312],[-0.0420621077,0.0713810363],[-0.0427337695,0.0698523409],[-0.043371275,0.0680773464],[-0.0440229466,0.0661672323],[-0.0447376031,0.0642461745],[-0.0455416788,0.0624102503],[-0.0464382879,0.0607247165],[-0.0474060065,0.0592524351],[-0.048397797,0.0579906541],[-0.0493560527,0.0569235527],[-0.0502032578,0.0560346863],[-0.0508775259,0.0552743117],[-0.051322987,0.0546044155],[-0.0514892243,0.05398351],[-0.0513523665,0.0533802236],[-0.0508990581,0.0527683438],[-0.0501364846,0.0521418417],[-0.0490909571,0.0514995285],[-0.0478082102,0.050859996],[-0.0463716804,0.050278109],[-0.0448616775,0.0498081061],[-0.0433851272,0.04952727],[-0.0420673957,0.0495131099],[-0.0410029728,0.0498354728],[-0.0402739969,0.0505422112],[-0.0399157579,0.0516225436],[-0.039921428,0.0530435709],[-0.040232795,0.0547088664],[-0.0407662796,0.0564894939],[-0.0414125802,0.0582390087],[-0.0420656521,0.0598217236],[-0.0426362598,0.0610991169],[-0.0430455251,0.061970168],[-0.0432611863,0.0623733292],[-0.0432431172,0.0622591887],[-0.0430202662,0.0616456233],[-0.0426253659,0.0605817944],[-0.0421132565,0.0591507565],[-0.0415622807,0.0574726378],[-0.0410586398,0.0556836398],[-0.0406875532,0.0539446961],[-0.0405155771,0.0523937914],[-0.040588337,0.0511504799],[-0.0409074899,0.0503085614],[-0.0414272976,0.0498776547],[-0.0420792198,0.0498581483],[-0.0427617185,0.0502010042],[-0.0433544117,0.0508161974],[-0.0437615103,0.051625796],[-0.0438952835,0.0525402835],[-0.0437051226,0.0534933369],[-0.0431605075,0.0544308646],[-0.0422749302,0.0553124302],[-0.0411036951,0.0561429808],[-0.0397055853,0.0569320139],[-0.0381868091,0.0577028805],[-0.0366571629,0.0584971629],[-0.035212257,0.0593693999],[-0.0339701317,0.0603529888],[-0.0330085765,0.0614835765],[-0.0323991912,0.0627834769],[-0.0321613061,0.0642184489],[-0.0323119887,0.0657844887],[-0.0328135134,0.0674117276],[-0.0336156361,0.0690245646],[-0.034641557,0.0705304855]],"A1120":[[-0.0196781264,0.0319114597],[-0.0205089742,0.0323295297],[-0.02172625,0.03293625],[-0.0233535508,0.0337446619],[-0.0253325218,0.0347169663],[-0.0276053384,0.0358108939],[-0.0300899683,0.0369594127],[-0.0326695279,0.0380850834],[-0.0352462515,0.0391195848],[-0.0377460842,0.0400183064],[-0.0400668268,0.0407301601],[-0.0421446679,0.0412302235],[-0.0439474602,0.0415146824],[-0.0454305979,0.0415861535],[-0.0466050886,0.0414756442],[-0.0474691779,0.0412091779],[-0.0480460817,0.0408316372],[-0.0483642993,0.0403809659],[-0.0484500241,0.0398922464],[-0.0483408049,0.0394052493],[-0.0480486146,0.0389286146],[-0.0476129827,0.0384890938],[-0.0470312161,0.0380812161],[-0.0463046984,0.0376930318],[-0.0454318752,0.0373113197],[-0.0444007171,0.036904606],[-0.0432011176,0.036449451],[-0.0418186152,0.0359230597],[-0.0402703729,0.0353131507],[-0.0385704162,0.0346287496],[-0.0367355569,0.0338611124],[-0.0348208068,0.0330408068],[-0.0328721349,0.0322015794],[-0.0309372252,0.0313661141],[-0.0291095883,0.0305912549],[-0.0274296646,0.029894109],[-0.0259501977,0.0293068643],[-0.0247004636,0.0288254636],[-0.0236934001,0.0284389557],[-0.0229135382,0.0281207604],[-0.022341535,0.0278320905],[-0.0219369262,0.0275363706],[-0.0216576849,0.0271910182],[-0.0214675182,0.0267830737],[-0.021334747,0.0262986359],[-0.0212406796,0.0257467907],[-0.0211768347,0.0251618347],[-0.0211268267,0.0245584934],[-0.0211047236,0.0239886125],[-0.0210929594,0.0234785149],[-0.021117889,0.023077889],[-0.0211600866,0.0228067532],[-0.021222692,0.0226893587],[-0.0213056371,0.0227423038],[-0.0213979529,0.0229612863],[-0.0214840433,0.0233351544],[-0.0215662652,0.0238673764],[-0.0216260642,0.024524953],[-0.021665765,0.0252874317],[-0.0216611151,0.0261105595],[-0.0216137,0.0269775889],[-0.0215111765,0.0278311765],[-0.0213490151,0.0286451262],[-0.0211207305,0.0293818416],[-0.0208308727,0.0300153171],[-0.0204879411,0.0305251634],[-0.0201107584,0.0309068695],[-0.0197342967,0.0311659634],[-0.0193943742,0.0313265964],[-0.0191481463,0.0314270352],[-0.0190633394,0.0315161172],[-0.0192226152,0.0316615041]],"A1121":[[-0.035821865,0.071839365],[-0.0370473444,0.0728355586],[-0.0382597229,0.0734518657],[-0.039401731,0.0736328025],[-0.040417054,0.0733334825],[-0.0412987169,0.0725694312],[-0.0420621077,0.0713810363],[-0.0427337695,0.0698523409],[-0.043371275,0.0680773464],[-0.0440229466,0.0661672323],[-0.0447376031,0.0642461745],[-0.0455416788,0.0624102503],[-0.0464382879,0.0607247165],[-0.0474060065,0.0592524351],[-0.048397797,0.0579906541],[-0.0493560527,0.0569235527],[-0.0502032578,0.0560346863],[-0.0508775259,0.0552743117],[-0.051322987,0.0546044155],[-0.0514892243,0.05398351],[-0.0513523665,0.0533802236],[-0.0508990581,0.0527683438],[-0.0501364846,0.0521418417],[-0.0490909571,0.0514995285],[-0.0478082102,0.050859996],[-0.0463716804,0.050278109],[-0.0448616775,0.0498081061],[-0.0433851272,0.04952727],[-0.0420673957,0.0495131099],[-0.0410029728,0.0498354728],[-0.0402739969,0.0505422112],[-0.0399157579,0.0516225436],[-0.039921428,0.0530435709],[-0.040232795,0.0547088664],[-0.0407662796,0.0564894939],[-0.0414125802,0.0582390087],[-0.0420656521,0.0598217236],[-0.0426362598,0.0610991169],[-0.0430455251,0.061970168],[-0.0432611863,0.0623733292],[-0.0432431172,0.0622591887],[-0.0430202662,0.0616456233],[-0.0426253659,0.0605817944],[-0.0421132565,0.0591507565],[-0.0415622807,0.0574726378],[-0.0410586398,0.0556836398],[-0.0406875532,0.0539446961],[-0.0405155771,0.0523937914],[-0.040588337,0.0511504799],[-0.0409074899,0.0503085614],[-0.0414272976,0.0498776547],[-0.0420792198,0.0498581483],[-0.0427617185,0.0502010042],[-0.0433544117,0.0508161974],[-0.0437615103,0.051625796],[-0.0438952835,0.0525402835],[-0.0437051226,0.0534933369],[-0.0431605075,0.0544308646],[-0.0422749302,0.0553124302],[-0.0411036951,0.0561429808],[-0.0397055853,0.0569320139],[-0.0381868091,0.0577028805],[-0.0366571629,0.0584971629],[-0.035212257,0.0593693999],[-0.0339701317,0.0603529888],[-0.0330085765,0.0614835765],[-0.0323991912,0.0627834769],[-0.0321613061,0.0642184489],[-0.0323119887,0.0657844887],[-0.0328135134,0.0674117276],[-0.0336156361,0.0690245646],[-0.034641557,0.0705304855]],"A1122":[[-0.035821865,0.071839365],[-0.0370473444,0.0728355586],[-0.0382597229,0.0734518657],[-0.039401731,0.0736328025],[-0.040417054,0.0733334825],[-0.0412987169,0.0725694312],[-0.0420621077,0.0713810363],[-0.0427337695,0.0698523409],[-0.043371275,0.0680773464],[-0.0440229466,0.0661672323],[-0.0447376031,0.0642461745],[-0.0455416788,0.0624102503],[-0.0464382879,0.0607247165],[-0.0474060065,0.0592524351],[-0.048397797,0.0579906541],[-0.0493560527,0.0569235527],[-0.0502032578,0.0560346863],[-0.0508775259,0.0552743117],[-0.051322987,0.0546044155],[-0.0514892243,0.05398351],[-0.0513523665,0.0533802236],[-0.0508990581,0.0527683438],[-0.0501364846,0.0521418417],[-0.0490909571,0.0514995285],[-0.0478082102,0.050859996],[-0.0463716804,0.050278109],[-0.0448616775,0.0498081061],[-0.0433851272,0.04952727],[-0.0420673957,0.0495131099],[-0.0410029728,0.0498354728],[-0.0402739969,0.0505422112],[-0.0399157579,0.0516225436],[-0.039921428,0.0530435709],[-0.040232795,0.0547088664],[-0.0407662796,0.0564894939],[-0.0414125802,0.0582390087],[-0.0420656521,0.0598217236],[-0.0426362598,0.0610991169],[-0.0430455251,0.061970168],[-0.0432611863,0.0623733292],[-0.0432431172,0.0622591887],[-0.0430202662,0.0616456233],[-0.0426253659,0.0605817944],[-0.0421132565,0.0591507565],[-0.0415622807,0.0574726378],[-0.0410586398,0.0556836398],[-0.0406875532,0.0539446961],[-0.0405155771,0.0523937914],[-0.040588337,0.0511504799],[-0.0409074899,0.0503085614],[-0.0414272976,0.0498776547],[-0.0420792198,0.0498581483],[-0.0427617185,0.0502010042],[-0.0433544117,0.0508161974],[-0.0437615103,0.051625796],[-0.0438952835,0.0525402835],[-0.0437051226,0.0534933369],[-0.0431605075,0.0544308646],[-0.0422749302,0.0553124302],[-0.0411036951,0.0561429808],[-0.0397055853,0.0569320139],[-0.0381868091,0.0577028805],[-0.0366571629,0.0584971629],[-0.035212257,0.0593693999],[-0.0339701317,0.0603529888],[-0.0330085765,0.0614835765],[-0.0323991912,0.0627834769],[-0.0321613061,0.0642184489],[-0.0323119887,0.0657844887],[-0.0328135134,0.0674117276],[-0.0336156361,0.0690245646],[-0.034641557,0.0705304855]],"A1123":[[-0.035821865,0.071839365],[-0.0370473444,0.0728355586],[-0.0382597229,0.0734518657],[-0.039401731,0.0736328025],[-0.040417054,0.0733334825],[-0.0412987169,0.0725694312],[-0.0420621077,0.0713810363],[-0.0427337695,0.0698523409],[-0.043371275,0.0680773464],[-0.0440229466,0.0661672323],[-0.0447376031,0.0642461745],[-0.0455416788,0.0624102503],[-0.0464382879,0.0607247165],[-0.0474060065,0.0592524351],[-0.048397797,0.0579906541],[-0.0493560527,0.0569235527],[-0.0502032578,0.0560346863],[-0.0508775259,0.0552743117],[-0.051322987,0.0546044155],[-0.0514892243,0.05398351],[-0.0513523665,0.0533802236],[-0.0508990581,0.0527683438],[-0.0501364846,0.0521418417],[-0.0490909571,0.0514995285],[-0.0478082102,0.050859996],[-0.0463716804,0.050278109],[-0.0448616775,0.0498081061],[-0.0433851272,0.04952727],[-0.0420673957,0.0495131099],[-0.0410029728,0.0498354728],[-0.0402739969,0.0505422112],[-0.0399157579,0.0516225436],[-0.039921428,0.0530435709],[-0.040232795,0.0547088664],[-0.0407662796,0.0564894939],[-0.0414125802,0.0582390087],[-0.0420656521,0.0598217236],[-0.0426362598,0.0610991169],[-0.0430455251,0.061970168],[-0.0432611863,0.0623733292],[-0.0432431172,0.0622591887],[-0.0430202662,0.0616456233],[-0.0426253659,0.0605817944],[-0.0421132565,0.0591507565],[-0.0415622807,0.0574726378],[-0.0410586398,0.0556836398],[-0.0406875532,0.0539446961],[-0.0405155771,0.0523937914],[-0.040588337,0.0511504799],[-0.0409074899,0.0503085614],[-0.0414272976,0.0498776547],[-0.0420792198,0.0498581483],[-0.0427617185,0.0502010042],[-0.0433544117,0.0508161974],[-0.0437615103,0.051625796],[-0.0438952835,0.0525402835],[-0.0437051226,0.0534933369],[-0.0431605075,0.0544308646],[-0.0422749302,0.0553124302],[-0.0411036951,0.0561429808],[-0.0397055853,0.0569320139],[-0.0381868091,0.0577028805],[-0.0366571629,0.0584971629],[-0.035212257,0.0593693999],[-0.0339701317,0.0603529888],[-0.0330085765,0.0614835765],[-0.0323991912,0.0627834769],[-0.0321613061,0.0642184489],[-0.0323119887,0.0657844887],[-0.0328135134,0.0674117276],[-0.0336156361,0.0690245646],[-0.034641557,0.0705304855]],"A1124":[[-0.035821865,0.071839365],[-0.0370473444,0.0728355586],[-0.0382597229,0.0734518657],[-0.039401731,0.0736328025],[-0.040417054,0.0733334825],[-0.0412987169,0.0725694312],[-0.0420621077,0.0713810363],[-0.0427337695,0.0698523409],[-0.043371275,0.0680773464],[-0.0440229466,0.0661672323],[-0.0447376031,0.0642461745],[-0.0455416788,0.0624102503],[-0.0464382879,0.0607247165],[-0.0474060065,0.0592524351],[-0.048397797,0.0579906541],[-0.0493560527,0.0569235527],[-0.0502032578,0.0560346863],[-0.0508775259,0.0552743117],[-0.051322987,0.0546044155],[-0.0514892243,0.05398351],[-0.0513523665,0.0533802236],[-0.0508990581,0.0527683438],[-0.0501364846,0.0521418417],[-0.0490909571,0.0514995285],[-0.0478082102,0.050859996],[-0.0463716804,0.050278109],[-0.0448616775,0.0498081061],[-0.0433851272,0.04952727],[-0.0420673957,0.0495131099],[-0.0410029728,0.0498354728],[-0.0402739969,0.0505422112],[-0.0399157579,0.0516225436],[-0.039921428,0.0530435709],[-0.040232795,0.0547088664],[-0.0407662796,0.0564894939],[-0.0414125802,0.0582390087],[-0.0420656521,0.0598217236],[-0.0426362598,0.0610991169],[-0.0430455251,0.061970168],[-0.0432611863,0.0623733292],[-0.0432431172,0.0622591887],[-0.0430202662,0.0616456233],[-0.0426253659,0.0605817944],[-0.0421132565,0.0591507565],[-0.0415622807,0.0574726378],[-0.0410586398,0.0556836398],[-0.0406875532,0.0539446961],[-0.0405155771,0.0523937914],[-0.040588337,0.0511504799],[-0.0409074899,0.0503085614],[-0.0414272976,0.0498776547],[-0.0420792198,0.0498581483],[-0.0427617185,0.0502010042],[-0.0433544117,0.0508161974],[-0.0437615103,0.051625796],[-0.0438952835,0.0525402835],[-0.0437051226,0.0534933369],[-0.0431605075,0.0544308646],[-0.0422749302,0.0553124302],[-0.0411036951,0.0561429808],[-0.0397055853,0.0569320139],[-0.0381868091,0.0577028805],[-0.0366571629,0.0584971629],[-0.035212257,0.0593693999],[-0.0339701317,0.0603529888],[-0.0330085765,0.0614835765],[-0.0323991912,0.0627834769],[-0.0321613061,0.0642184489],[-0.0323119887,0.0657844887],[-0.0328135134,0.0674117276],[-0.0336156361,0.0690245646],[-0.034641557,0.0705304855]],"A1125":[[-0.035821865,0.071839365],[-0.0370473444,0.0728355586],[-0.0382597229,0.0734518657],[-0.039401731,0.0736328025],[-0.040417054,0.0733334825],[-0.0412987169,0.0725694312],[-0.0420621077,0.0713810363],[-0.0427337695,0.0698523409],[-0.043371275,0.0680773464],[-0.0440229466,0.0661672323],[-0.0447376031,0.0642461745],[-0.0455416788,0.0624102503],[-0.0464382879,0.0607247165],[-0.0474060065,0.0592524351],[-0.048397797,0.0579906541],[-0.0493560527,0.0569235527],[-0.0502032578,0.0560346863],[-0.0508775259,0.0552743117],[-0.051322987,0.0546044155],[-0.0514892243,0.05398351],[-0.0513523665,0.0533802236],[-0.0508990581,0.0527683438],[-0.0501364846,0.0521418417],[-0.0490909571,0.0514995285],[-0.0478082102,0.050859996],[-0.0463716804,0.050278109],[-0.0448616775,0.0498081061],[-0.0433851272,0.04952727],[-0.0420673957,0.0495131099],[-0.0410029728,0.0498354728],[-0.0402739969,0.0505422112],[-0.0399157579,0.0516225436],[-0.039921428,0.0530435709],[-0.040232795,0.0547088664],[-0.0407662796,0.0564894939],[-0.0414125802,0.0582390087],[-0.0420656521,0.0598217236],[-0.0426362598,0.0610991169],[-0.0430455251,0.061970168],[-0.0432611863,0.0623733292],[-0.0432431172,0.0622591887],[-0.0430202662,0.0616456233],[-0.0426253659,0.0605817944],[-0.0421132565,0.0591507565],[-0.0415622807,0.0574726378],[-0.0410586398,0.0556836398],[-0.0406875532,0.0539446961],[-0.0405155771,0.0523937914],[-0.040588337,0.0511504799],[-0.0409074899,0.0503085614],[-0.0414272976,0.0498776547],[-0.0420792198,0.0498581483],[-0.0427617185,0.0502010042],[-0.0433544117,0.0508161974],[-0.0437615103,0.051625796],[-0.0438952835,0.0525402835],[-0.0437051226,0.0534933369],[-0.0431605075,0.0544308646],[-0.0422749302,0.0553124302],[-0.0411036951,0.0561429808],[-0.0397055853,0.0569320139],[-0.0381868091,0.0577028805],[-0.0366571629,0.0584971629],[-0.035212257,0.0593693999],[-0.0339701317,0.0603529888],[-0.0330085765,0.0614835765],[-0.0323991912,0.0627834769],[-0.0321613061,0.0642184489],[-0.0323119887,0.0657844887],[-0.0328135134,0.0674117276],[-0.0336156361,0.0690245646],[-0.034641557,0.0705304855]],"A1126":[[-0.035821865,0.071839365],[-0.0370473444,0.0728355586],[-0.0382597229,0.0734518657],[-0.039401731,0.0736328025],[-0.040417054,0.0733334825],[-0.0412987169,0.0725694312],[-0.0420621077,0.0713810363],[-0.0427337695,0.0698523409],[-0.043371275,0.0680773464],[-0.0440229466,0.0661672323],[-0.0447376031,0.0642461745],[-0.0455416788,0.0624102503],[-0.0464382879,0.0607247165],[-0.0474060065,0.0592524351],[-0.048397797,0.0579906541],[-0.0493560527,0.0569235527],[-0.0502032578,0.0560346863],[-0.0508775259,0.0552743117],[-0.051322987,0.0546044155],[-0.0514892243,0.05398351],[-0.0513523665,0.0533802236],[-0.0508990581,0.0527683438],[-0.0501364846,0.0521418417],[-0.0490909571,0.0514995285],[-0.0478082102,0.050859996],[-0.0463716804,0.050278109],[-0.0448616775,0.0498081061],[-0.0433851272,0.04952727],[-0.0420673957,0.0495131099],[-0.0410029728,0.0498354728],[-0.0402739969,0.0505422112],[-0.0399157579,0.0516225436],[-0.039921428,0.0530435709],[-0.040232795,0.0547088664],[-0.0407662796,0.0564894939],[-0.0414125802,0.0582390087],[-0.0420656521,0.0598217236],[-0.0426362598,0.0610991169],[-0.0430455251,0.061970168],[-0.0432611863,0.0623733292],[-0.0432431172,0.0622591887],[-0.0430202662,0.0616456233],[-0.0426253659,0.0605817944],[-0.0421132565,0.0591507565],[-0.0415622807,0.0574726378],[-0.0410586398,0.0556836398],[-0.0406875532,0.0539446961],[-0.0405155771,0.0523937914],[-0.040588337,0.0511504799],[-0.0409074899,0.0503085614],[-0.0414272976,0.0498776547],[-0.0420792198,0.0498581483],[-0.0427617185,0.0502010042],[-0.0433544117,0.0508161974],[-0.0437615103,0.051625796],[-0.0438952835,0.0525402835],[-0.0437051226,0.0534933369],[-0.0431605075,0.0544308646],[-0.0422749302,0.0553124302],[-0.0411036951,0.0561429808],[-0.0397055853,0.0569320139],[-0.0381868091,0.0577028805],[-0.0366571629,0.0584971629],[-0.035212257,0.0593693999],[-0.0339701317,0.0603529888],[-0.0330085765,0.0614835765],[-0.0323991912,0.0627834769],[-0.0321613061,0.0642184489],[-0.0323119887,0.0657844887],[-0.0328135134,0.0674117276],[-0.0336156361,0.0690245646],[-0.034641557,0.0705304855]],"A1127":[[-0.035821865,0.071839365],[-0.0370473444,0.0728355586],[-0.0382597229,0.0734518657],[-0.039401731,0.0736328025],[-0.040417054,0.0733334825],[-0.0412987169,0.0725694312],[-0.0420621077,0.0713810363],[-0.0427337695,0.0698523409],[-0.043371275,0.0680773464],[-0.0440229466,0.0661672323],[-0.0447376031,0.0642461745],[-0.0455416788,0.0624102503],[-0.0464382879,0.0607247165],[-0.0474060065,0.0592524351],[-0.048397797,0.0579906541],[-0.0493560527,0.0569235527],[-0.0502032578,0.0560346863],[-0.0508775259,0.0552743117],[-0.051322987,0.0546044155],[-0.0514892243,0.05398351],[-0.0513523665,0.0533802236],[-0.0508990581,0.0527683438],[-0.0501364846,0.0521418417],[-0.0490909571,0.0514995285],[-0.0478082102,0.050859996],[-0.0463716804,0.050278109],[-0.0448616775,0.0498081061],[-0.0433851272,0.04952727],[-0.0420673957,0.0495131099],[-0.0410029728,0.0498354728],[-0.0402739969,0.0505422112],[-0.0399157579,0.0516225436],[-0.039921428,0.0530435709],[-0.040232795,0.0547088664],[-0.0407662796,0.0564894939],[-0.0414125802,0.0582390087],[-0.0420656521,0.0598217236],[-0.0426362598,0.0610991169],[-0.0430455251,0.061970168],[-0.0432611863,0.0623733292],[-0.0432431172,0.0622591887],[-0.0430202662,0.0616456233],[-0.0426253659,0.0605817944],[-0.0421132565,0.0591507565],[-0.0415622807,0.0574726378],[-0.0410586398,0.0556836398],[-0.0406875532,0.0539446961],[-0.0405155771,0.0523937914],[-0.040588337,0.0511504799],[-0.0409074899,0.0503085614],[-0.0414272976,0.0498776547],[-0.0420792198,0.0498581483],[-0.0427617185,0.0502010042],[-0.0433544117,0.0508161974],[-0.0437615103,0.051625796],[-0.0438952835,0.0525402835],[-0.0437051226,0.0534933369],[-0.0431605075,0.0544308646],[-0.0422749302,0.0553124302],[-0.0411036951,0.0561429808],[-0.0397055853,0.0569320139],[-0.0381868091,0.0577028805],[-0.0366571629,0.0584971629],[-0.035212257,0.0593693999],[-0.0339701317,0.0603529888],[-0.0330085765,0.0614835765],[-0.0323991912,0.0627834769],[-0.0321613061,0.0642184489],[-0.0323119887,0.0657844887],[-0.0328135134,0.0674117276],[-0.0336156361,0.0690245646],[-0.034641557,0.0705304855]],"A1128":[[-0.035821865,0.071839365],[-0.0370473444,0.0728355586],[-0.0382597229,0.0734518657],[-0.039401731,0.0736328025],[-0.040417054,0.0733334825],[-0.0412987169,0.0725694312],[-0.0420621077,0.0713810363],[-0.0427337695,0.0698523409],[-0.043371275,0.0680773464],[-0.0440229466,0.0661672323],[-0.0447376031,0.0642461745],[-0.0455416788,0.0624102503],[-0.0464382879,0.0607247165],[-0.0474060065,0.0592524351],[-0.048397797,0.0579906541],[-0.0493560527,0.0569235527],[-0.0502032578,0.0560346863],[-0.0508775259,0.0552743117],[-0.051322987,0.0546044155],[-0.0514892243,0.05398351],[-0.0513523665,0.0533802236],[-0.0508990581,0.0527683438],[-0.0501364846,0.0521418417],[-0.0490909571,0.0514995285],[-0.0478082102,0.050859996],[-0.0463716804,0.050278109],[-0.0448616775,0.0498081061],[-0.0433851272,0.04952727],[-0.0420673957,0.0495131099],[-0.0410029728,0.0498354728],[-0.0402739969,0.0505422112],[-0.0399157579,0.0516225436],[-0.039921428,0.0530435709],[-0.040232795,0.0547088664],[-0.0407662796,0.0564894939],[-0.0414125802,0.0582390087],[-0.0420656521,0.0598217236],[-0.0426362598,0.0610991169],[-0.0430455251,0.061970168],[-0.0432611863,0.0623733292],[-0.0432431172,0.0622591887],[-0.0430202662,0.0616456233],[-0.0426253659,0.0605817944],[-0.0421132565,0.0591507565],[-0.0415622807,0.0574726378],[-0.0410586398,0.0556836398],[-0.0406875532,0.0539446961],[-0.0405155771,0.0523937914],[-0.040588337,0.0511504799],[-0.0409074899,0.0503085614],[-0.0414272976,0.0498776547],[-0.0420792198,0.0498581483],[-0.0427617185,0.0502010042],[-0.0433544117,0.0508161974],[-0.0437615103,0.051625796],[-0.0438952835,0.0525402835],[-0.0437051226,0.0534933369],[-0.0431605075,0.0544308646],[-0.0422749302,0.0553124302],[-0.0411036951,0.0561429808],[-0.0397055853,0.0569320139],[-0.0381868091,0.0577028805],[-0.0366571629,0.0584971629],[-0.035212257,0.0593693999],[-0.0339701317,0.0603529888],[-0.0330085765,0.0614835765],[-0.0323991912,0.0627834769],[-0.0321613061,0.0642184489],[-0.0323119887,0.0657844887],[-0.0328135134,0.0674117276],[-0.0336156361,0.0690245646],[-0.034641557,0.0705304855]],"A1129":[[-0.035821865,0.071839365],[-0.0370473444,0.0728355586],[-0.0382597229,0.0734518657],[-0.039401731,0.0736328025],[-0.040417054,0.0733334825],[-0.0412987169,0.0725694312],[-0.0420621077,0.0713810363],[-0.0427337695,0.0698523409],[-0.043371275,0.0680773464],[-0.0440229466,0.0661672323],[-0.0447376031,0.0642461745],[-0.0455416788,0.0624102503],[-0.0464382879,0.0607247165],[-0.0474060065,0.0592524351],[-0.048397797,0.0579906541],[-0.0493560527,0.0569235527],[-0.0502032578,0.0560346863],[-0.0508775259,0.0552743117],[-0.051322987,0.0546044155],[-0.0514892243,0.05398351],[-0.0513523665,0.0533802236],[-0.0508990581,0.0527683438],[-0.0501364846,0.0521418417],[-0.0490909571,0.0514995285],[-0.0478082102,0.050859996],[-0.0463716804,0.050278109],[-0.0448616775,0.0498081061],[-0.0433851272,0.04952727],[-0.0420673957,0.0495131099],[-0.0410029728,0.0498354728],[-0.0402739969,0.0505422112],[-0.0399157579,0.0516225436],[-0.039921428,0.0530435709],[-0.040232795,0.0547088664],[-0.0407662796,0.0564894939],[-0.0414125802,0.0582390087],[-0.0420656521,0.0598217236],[-0.0426362598,0.0610991169],[-0.0430455251,0.061970168],[-0.0432611863,0.0623733292],[-0.0432431172,0.0622591887],[-0.0430202662,0.0616456233],[-0.0426253659,0.0605817944],[-0.0421132565,0.0591507565],[-0.0415622807,0.0574726378],[-0.0410586398,0.0556836398],[-0.0406875532,0.0539446961],[-0.0405155771,0.0523937914],[-0.040588337,0.0511504799],[-0.0409074899,0.0503085614],[-0.0414272976,0.0498776547],[-0.0420792198,0.0498581483],[-0.0427617185,0.0502010042],[-0.0433544117,0.0508161974],[-0.0437615103,0.051625796],[-0.0438952835,0.0525402835],[-0.0437051226,0.0534933369],[-0.0431605075,0.0544308646],[-0.0422749302,0.0553124302],[-0.0411036951,0.0561429808],[-0.0397055853,0.0569320139],[-0.0381868091,0.0577028805],[-0.0366571629,0.0584971629],[-0.035212257,0.0593693999],[-0.0339701317,0.0603529888],[-0.0330085765,0.0614835765],[-0.0323991912,0.0627834769],[-0.0321613061,0.0642184489],[-0.0323119887,0.0657844887],[-0.0328135134,0.0674117276],[-0.0336156361,0.0690245646],[-0.034641557,0.0705304855]],"A1130":[[-0.035821865,0.071839365],[-0.0370473444,0.0728355586],[-0.0382597229,0.0734518657],[-0.039401731,0.0736328025],[-0.040417054,0.0733334825],[-0.0412987169,0.0725694312],[-0.0420621077,0.0713810363],[-0.0427337695,0.0698523409],[-0.043371275,0.0680773464],[-0.0440229466,0.0661672323],[-0.0447376031,0.0642461745],[-0.0455416788,0.0624102503],[-0.0464382879,0.0607247165],[-0.0474060065,0.0592524351],[-0.048397797,0.0579906541],[-0.0493560527,0.0569235527],[-0.0502032578,0.0560346863],[-0.0508775259,0.0552743117],[-0.051322987,0.0546044155],[-0.0514892243,0.05398351],[-0.0513523665,0.0533802236],[-0.0508990581,0.0527683438],[-0.0501364846,0.0521418417],[-0.0490909571,0.0514995285],[-0.0478082102,0.050859996],[-0.0463716804,0.050278109],[-0.0448616775,0.0498081061],[-0.0433851272,0.04952727],[-0.0420673957,0.0495131099],[-0.0410029728,0.0498354728],[-0.0402739969,0.0505422112],[-0.0399157579,0.0516225436],[-0.039921428,0.0530435709],[-0.040232795,0.0547088664],[-0.0407662796,0.0564894939],[-0.0414125802,0.0582390087],[-0.0420656521,0.0598217236],[-0.0426362598,0.0610991169],[-0.0430455251,0.061970168],[-0.0432611863,0.0623733292],[-0.0432431172,0.0622591887],[-0.0430202662,0.0616456233],[-0.0426253659,0.0605817944],[-0.0421132565,0.0591507565],[-0.0415622807,0.0574726378],[-0.0410586398,0.0556836398],[-0.0406875532,0.0539446961],[-0.0405155771,0.0523937914],[-0.040588337,0.0511504799],[-0.0409074899,0.0503085614],[-0.0414272976,0.0498776547],[-0.0420792198,0.0498581483],[-0.0427617185,0.0502010042],[-0.0433544117,0.0508161974],[-0.0437615103,0.051625796],[-0.0438952835,0.0525402835],[-0.0437051226,0.0534933369],[-0.0431605075,0.0544308646],[-0.0422749302,0.0553124302],[-0.0411036951,0.0561429808],[-0.0397055853,0.0569320139],[-0.0381868091,0.0577028805],[-0.0366571629,0.0584971629],[-0.035212257,0.0593693999],[-0.0339701317,0.0603529888],[-0.0330085765,0.0614835765],[-0.0323991912,0.0627834769],[-0.0321613061,0.0642184489],[-0.0323119887,0.0657844887],[-0.0328135134,0.0674117276],[-0.0336156361,0.0690245646],[-0.034641557,0.0705304855]],"A1131":[[-0.035821865,0.071839365],[-0.0370473444,0.0728355586],[-0.0382597229,0.0734518657],[-0.039401731,0.0736328025],[-0.040417054,0.0733334825],[-0.0412987169,0.0725694312],[-0.0420621077,0.0713810363],[-0.0427337695,0.0698523409],[-0.043371275,0.0680773464],[-0.0440229466,0.0661672323],[-0.0447376031,0.0642461745],[-0.0455416788,0.0624102503],[-0.0464382879,0.0607247165],[-0.0474060065,0.0592524351],[-0.048397797,0.0579906541],[-0.0493560527,0.0569235527],[-0.0502032578,0.0560346863],[-0.0508775259,0.0552743117],[-0.051322987,0.0546044155],[-0.0514892243,0.05398351],[-0.0513523665,0.0533802236],[-0.0508990581,0.0527683438],[-0.0501364846,0.0521418417],[-0.0490909571,0.0514995285],[-0.0478082102,0.050859996],[-0.0463716804,0.050278109],[-0.0448616775,0.0498081061],[-0.0433851272,0.04952727],[-0.0420673957,0.0495131099],[-0.0410029728,0.0498354728],[-0.0402739969,0.0505422112],[-0.0399157579,0.0516225436],[-0.039921428,0.0530435709],[-0.040232795,0.0547088664],[-0.0407662796,0.0564894939],[-0.0414125802,0.0582390087],[-0.0420656521,0.0598217236],[-0.0426362598,0.0610991169],[-0.0430455251,0.061970168],[-0.0432611863,0.0623733292],[-0.0432431172,0.0622591887],[-0.0430202662,0.0616456233],[-0.0426253659,0.0605817944],[-0.0421132565,0.0591507565],[-0.0415622807,0.0574726378],[-0.0410586398,0.0556836398],[-0.0406875532,0.0539446961],[-0.0405155771,0.0523937914],[-0.040588337,0.0511504799],[-0.0409074899,0.0503085614],[-0.0414272976,0.0498776547],[-0.0420792198,0.0498581483],[-0.0427617185,0.0502010042],[-0.0433544117,0.0508161974],[-0.0437615103,0.051625796],[-0.0438952835,0.0525402835],[-0.0437051226,0.0534933369],[-0.0431605075,0.0544308646],[-0.0422749302,0.0553124302],[-0.0411036951,0.0561429808],[-0.0397055853,0.0569320139],[-0.0381868091,0.0577028805],[-0.0366571629,0.0584971629],[-0.035212257,0.0593693999],[-0.0339701317,0.0603529888],[-0.0330085765,0.0614835765],[-0.0323991912,0.0627834769],[-0.0321613061,0.0642184489],[-0.0323119887,0.0657844887],[-0.0328135134,0.0674117276],[-0.0336156361,0.0690245646],[-0.034641557,0.0705304855]],"A1132":[[-0.035821865,0.071839365],[-0.0370473444,0.0728355586],[-0.0382597229,0.0734518657],[-0.039401731,0.0736328025],[-0.040417054,0.0733334825],[-0.0412987169,0.0725694312],[-0.0420621077,0.0713810363],[-0.0427337695,0.0698523409],[-0.043371275,0.0680773464],[-0.0440229466,0.0661672323],[-0.0447376031,0.0642461745],[-0.0455416788,0.0624102503],[-0.0464382879,0.0607247165],[-0.0474060065,0.0592524351],[-0.048397797,0.0579906541],[-0.0493560527,0.0569235527],[-0.0502032578,0.0560346863],[-0.0508775259,0.0552743117],[-0.051322987,0.0546044155],[-0.0514892243,0.05398351],[-0.0513523665,0.0533802236],[-0.0508990581,0.0527683438],[-0.0501364846,0.0521418417],[-0.0490909571,0.0514995285],[-0.0478082102,0.050859996],[-0.0463716804,0.050278109],[-0.0448616775,0.0498081061],[-0.0433851272,0.04952727],[-0.0420673957,0.0495131099],[-0.0410029728,0.0498354728],[-0.0402739969,0.0505422112],[-0.0399157579,0.0516225436],[-0.039921428,0.0530435709],[-0.040232795,0.0547088664],[-0.0407662796,0.0564894939],[-0.0414125802,0.0582390087],[-0.0420656521,0.0598217236],[-0.0426362598,0.0610991169],[-0.0430455251,0.061970168],[-0.0432611863,0.0623733292],[-0.0432431172,0.0622591887],[-0.0430202662,0.0616456233],[-0.0426253659,0.0605817944],[-0.0421132565,0.0591507565],[-0.0415622807,0.0574726378],[-0.0410586398,0.0556836398],[-0.0406875532,0.0539446961],[-0.0405155771,0.0523937914],[-0.040588337,0.0511504799],[-0.0409074899,0.0503085614],[-0.0414272976,0.0498776547],[-0.0420792198,0.0498581483],[-0.0427617185,0.0502010042],[-0.0433544117,0.0508161974],[-0.0437615103,0.051625796],[-0.0438952835,0.0525402835],[-0.0437051226,0.0534933369],[-0.0431605075,0.0544308646],[-0.0422749302,0.0553124302],[-0.0411036951,0.0561429808],[-0.0397055853,0.0569320139],[-0.0381868091,0.0577028805],[-0.0366571629,0.0584971629],[-0.035212257,0.0593693999],[-0.0339701317,0.0603529888],[-0.0330085765,0.0614835765],[-0.0323991912,0.0627834769],[-0.0321613061,0.0642184489],[-0.0323119887,0.0657844887],[-0.0328135134,0.0674117276],[-0.0336156361,0.0690245646],[-0.034641557,0.0705304855]],"A1133":[[-0.035821865,0.071839365],[-0.0370473444,0.0728355586],[-0.0382597229,0.0734518657],[-0.039401731,0.0736328025],[-0.040417054,0.0733334825],[-0.0412987169,0.0725694312],[-0.0420621077,0.0713810363],[-0.0427337695,0.0698523409],[-0.043371275,0.0680773464],[-0.0440229466,0.0661672323],[-0.0447376031,0.0642461745],[-0.0455416788,0.0624102503],[-0.0464382879,0.0607247165],[-0.0474060065,0.0592524351],[-0.048397797,0.0579906541],[-0.0493560527,0.0569235527],[-0.0502032578,0.0560346863],[-0.0508775259,0.0552743117],[-0.051322987,0.0546044155],[-0.0514892243,0.05398351],[-0.0513523665,0.0533802236],[-0.0508990581,0.0527683438],[-0.0501364846,0.0521418417],[-0.0490909571,0.0514995285],[-0.0478082102,0.050859996],[-0.0463716804,0.050278109],[-0.0448616775,0.0498081061],[-0.0433851272,0.04952727],[-0.0420673957,0.0495131099],[-0.0410029728,0.0498354728],[-0.0402739969,0.0505422112],[-0.0399157579,0.0516225436],[-0.039921428,0.0530435709],[-0.040232795,0.0547088664],[-0.0407662796,0.0564894939],[-0.0414125802,0.0582390087],[-0.0420656521,0.0598217236],[-0.0426362598,0.0610991169],[-0.0430455251,0.061970168],[-0.0432611863,0.0623733292],[-0.0432431172,0.0622591887],[-0.0430202662,0.0616456233],[-0.0426253659,0.0605817944],[-0.0421132565,0.0591507565],[-0.0415622807,0.0574726378],[-0.0410586398,0.0556836398],[-0.0406875532,0.0539446961],[-0.0405155771,0.0523937914],[-0.040588337,0.0511504799],[-0.0409074899,0.0503085614],[-0.0414272976,0.0498776547],[-0.0420792198,0.0498581483],[-0.0427617185,0.0502010042],[-0.0433544117,0.0508161974],[-0.0437615103,0.051625796],[-0.0438952835,0.0525402835],[-0.0437051226,0.0534933369],[-0.0431605075,0.0544308646],[-0.0422749302,0.0553124302],[-0.0411036951,0.0561429808],[-0.0397055853,0.0569320139],[-0.0381868091,0.0577028805],[-0.0366571629,0.0584971629],[-0.035212257,0.0593693999],[-0.0339701317,0.0603529888],[-0.0330085765,0.0614835765],[-0.0323991912,0.0627834769],[-0.0321613061,0.0642184489],[-0.0323119887,0.0657844887],[-0.0328135134,0.0674117276],[-0.0336156361,0.0690245646],[-0.034641557,0.0705304855]],"A1134":[[-0.035821865,0.071839365],[-0.0370473444,0.0728355586],[-0.0382597229,0.0734518657],[-0.039401731,0.0736328025],[-0.040417054,0.0733334825],[-0.0412987169,0.0725694312],[-0.0420621077,0.0713810363],[-0.0427337695,0.0698523409],[-0.043371275,0.0680773464],[-0.0440229466,0.0661672323],[-0.0447376031,0.0642461745],[-0.0455416788,0.0624102503],[-0.0464382879,0.0607247165],[-0.0474060065,0.0592524351],[-0.048397797,0.0579906541],[-0.0493560527,0.0569235527],[-0.0502032578,0.0560346863],[-0.0508775259,0.0552743117],[-0.051322987,0.0546044155],[-0.0514892243,0.05398351],[-0.0513523665,0.0533802236],[-0.0508990581,0.0527683438],[-0.0501364846,0.0521418417],[-0.0490909571,0.0514995285],[-0.0478082102,0.050859996],[-0.0463716804,0.050278109],[-0.0448616775,0.0498081061],[-0.0433851272,0.04952727],[-0.0420673957,0.0495131099],[-0.0410029728,0.0498354728],[-0.0402739969,0.0505422112],[-0.0399157579,0.0516225436],[-0.039921428,0.0530435709],[-0.040232795,0.0547088664],[-0.0407662796,0.0564894939],[-0.0414125802,0.0582390087],[-0.0420656521,0.0598217236],[-0.0426362598,0.0610991169],[-0.0430455251,0.061970168],[-0.0432611863,0.0623733292],[-0.0432431172,0.0622591887],[-0.0430202662,0.0616456233],[-0.0426253659,0.0605817944],[-0.0421132565,0.0591507565],[-0.0415622807,0.0574726378],[-0.0410586398,0.0556836398],[-0.0406875532,0.0539446961],[-0.0405155771,0.0523937914],[-0.040588337,0.0511504799],[-0.0409074899,0.0503085614],[-0.0414272976,0.0498776547],[-0.0420792198,0.0498581483],[-0.0427617185,0.0502010042],[-0.0433544117,0.0508161974],[-0.0437615103,0.051625796],[-0.0438952835,0.0525402835],[-0.0437051226,0.0534933369],[-0.0431605075,0.0544308646],[-0.0422749302,0.0553124302],[-0.0411036951,0.0561429808],[-0.0397055853,0.0569320139],[-0.0381868091,0.0577028805],[-0.0366571629,0.0584971629],[-0.035212257,0.0593693999],[-0.0339701317,0.0603529888],[-0.0330085765,0.0614835765],[-0.0323991912,0.0627834769],[-0.0321613061,0.0642184489],[-0.0323119887,0.0657844887],[-0.0328135134,0.0674117276],[-0.0336156361,0.0690245646],[-0.034641557,0.0705304855]],"A1135":[[-0.035821865,0.071839365],[-0.0370473444,0.0728355586],[-0.0382597229,0.0734518657],[-0.039401731,0.0736328025],[-0.040417054,0.0733334825],[-0.0412987169,0.0725694312],[-0.0420621077,0.0713810363],[-0.0427337695,0.0698523409],[-0.043371275,0.0680773464],[-0.0440229466,0.0661672323],[-0.0447376031,0.0642461745],[-0.0455416788,0.0624102503],[-0.0464382879,0.0607247165],[-0.0474060065,0.0592524351],[-0.048397797,0.0579906541],[-0.0493560527,0.0569235527],[-0.0502032578,0.0560346863],[-0.0508775259,0.0552743117],[-0.051322987,0.0546044155],[-0.0514892243,0.05398351],[-0.0513523665,0.0533802236],[-0.0508990581,0.0527683438],[-0.0501364846,0.0521418417],[-0.0490909571,0.0514995285],[-0.0478082102,0.050859996],[-0.0463716804,0.050278109],[-0.0448616775,0.0498081061],[-0.0433851272,0.04952727],[-0.0420673957,0.0495131099],[-0.0410029728,0.0498354728],[-0.0402739969,0.0505422112],[-0.0399157579,0.0516225436],[-0.039921428,0.0530435709],[-0.040232795,0.0547088664],[-0.0407662796,0.0564894939],[-0.0414125802,0.0582390087],[-0.0420656521,0.0598217236],[-0.0426362598,0.0610991169],[-0.0430455251,0.061970168],[-0.0432611863,0.0623733292],[-0.0432431172,0.0622591887],[-0.0430202662,0.0616456233],[-0.0426253659,0.0605817944],[-0.0421132565,0.0591507565],[-0.0415622807,0.0574726378],[-0.0410586398,0.0556836398],[-0.0406875532,0.0539446961],[-0.0405155771,0.0523937914],[-0.040588337,0.0511504799],[-0.0409074899,0.0503085614],[-0.0414272976,0.0498776547],[-0.0420792198,0.0498581483],[-0.0427617185,0.0502010042],[-0.0433544117,0.0508161974],[-0.0437615103,0.051625796],[-0.0438952835,0.0525402835],[-0.0437051226,0.0534933369],[-0.0431605075,0.0544308646],[-0.0422749302,0.0553124302],[-0.0411036951,0.0561429808],[-0.0397055853,0.0569320139],[-0.0381868091,0.0577028805],[-0.0366571629,0.0584971629],[-0.035212257,0.0593693999],[-0.0339701317,0.0603529888],[-0.0330085765,0.0614835765],[-0.0323991912,0.0627834769],[-0.0321613061,0.0642184489],[-0.0323119887,0.0657844887],[-0.0328135134,0.0674117276],[-0.0336156361,0.0690245646],[-0.034641557,0.0705304855]],"A1136":[[-0.035821865,0.071839365],[-0.0370473444,0.0728355586],[-0.0382597229,0.0734518657],[-0.039401731,0.0736328025],[-0.040417054,0.0733334825],[-0.0412987169,0.0725694312],[-0.0420621077,0.0713810363],[-0.0427337695,0.0698523409],[-0.043371275,0.0680773464],[-0.0440229466,0.0661672323],[-0.0447376031,0.0642461745],[-0.0455416788,0.0624102503],[-0.0464382879,0.0607247165],[-0.0474060065,0.0592524351],[-0.048397797,0.0579906541],[-0.0493560527,0.0569235527],[-0.0502032578,0.0560346863],[-0.0508775259,0.0552743117],[-0.051322987,0.0546044155],[-0.0514892243,0.05398351],[-0.0513523665,0.0533802236],[-0.0508990581,0.0527683438],[-0.0501364846,0.0521418417],[-0.0490909571,0.0514995285],[-0.0478082102,0.050859996],[-0.0463716804,0.050278109],[-0.0448616775,0.0498081061],[-0.0433851272,0.04952727],[-0.0420673957,0.0495131099],[-0.0410029728,0.0498354728],[-0.0402739969,0.0505422112],[-0.0399157579,0.0516225436],[-0.039921428,0.0530435709],[-0.040232795,0.0547088664],[-0.0407662796,0.0564894939],[-0.0414125802,0.0582390087],[-0.0420656521,0.0598217236],[-0.0426362598,0.0610991169],[-0.0430455251,0.061970168],[-0.0432611863,0.0623733292],[-0.0432431172,0.0622591887],[-0.0430202662,0.0616456233],[-0.0426253659,0.0605817944],[-0.0421132565,0.0591507565],[-0.0415622807,0.0574726378],[-0.0410586398,0.0556836398],[-0.0406875532,0.0539446961],[-0.0405155771,0.0523937914],[-0.040588337,0.0511504799],[-0.0409074899,0.0503085614],[-0.0414272976,0.0498776547],[-0.0420792198,0.0498581483],[-0.0427617185,0.0502010042],[-0.0433544117,0.0508161974],[-0.0437615103,0.051625796],[-0.0438952835,0.0525402835],[-0.0437051226,0.0534933369],[-0.0431605075,0.0544308646],[-0.0422749302,0.0553124302],[-0.0411036951,0.0561429808],[-0.0397055853,0.0569320139],[-0.0381868091,0.0577028805],[-0.0366571629,0.0584971629],[-0.035212257,0.0593693999],[-0.0339701317,0.0603529888],[-0.0330085765,0.0614835765],[-0.0323991912,0.0627834769],[-0.0321613061,0.0642184489],[-0.0323119887,0.0657844887],[-0.0328135134,0.0674117276],[-0.0336156361,0.0690245646],[-0.034641557,0.0705304855]],"A1137":[[-0.035821865,0.071839365],[-0.0370473444,0.0728355586],[-0.0382597229,0.0734518657],[-0.039401731,0.0736328025],[-0.040417054,0.0733334825],[-0.0412987169,0.0725694312],[-0.0420621077,0.0713810363],[-0.0427337695,0.0698523409],[-0.043371275,0.0680773464],[-0.0440229466,0.0661672323],[-0.0447376031,0.0642461745],[-0.0455416788,0.0624102503],[-0.0464382879,0.0607247165],[-0.0474060065,0.0592524351],[-0.048397797,0.0579906541],[-0.0493560527,0.0569235527],[-0.0502032578,0.0560346863],[-0.0508775259,0.0552743117],[-0.051322987,0.0546044155],[-0.0514892243,0.05398351],[-0.0513523665,0.0533802236],[-0.0508990581,0.0527683438],[-0.0501364846,0.0521418417],[-0.0490909571,0.0514995285],[-0.0478082102,0.050859996],[-0.0463716804,0.050278109],[-0.0448616775,0.0498081061],[-0.0433851272,0.04952727],[-0.0420673957,0.0495131099],[-0.0410029728,0.0498354728],[-0.0402739969,0.0505422112],[-0.0399157579,0.0516225436],[-0.039921428,0.0530435709],[-0.040232795,0.0547088664],[-0.0407662796,0.0564894939],[-0.0414125802,0.0582390087],[-0.0420656521,0.0598217236],[-0.0426362598,0.0610991169],[-0.0430455251,0.061970168],[-0.0432611863,0.0623733292],[-0.0432431172,0.0622591887],[-0.0430202662,0.0616456233],[-0.0426253659,0.0605817944],[-0.0421132565,0.0591507565],[-0.0415622807,0.0574726378],[-0.0410586398,0.0556836398],[-0.0406875532,0.0539446961],[-0.0405155771,0.0523937914],[-0.040588337,0.0511504799],[-0.0409074899,0.0503085614],[-0.0414272976,0.0498776547],[-0.0420792198,0.0498581483],[-0.0427617185,0.0502010042],[-0.0433544117,0.0508161974],[-0.0437615103,0.051625796],[-0.0438952835,0.0525402835],[-0.0437051226,0.0534933369],[-0.0431605075,0.0544308646],[-0.0422749302,0.0553124302],[-0.0411036951,0.0561429808],[-0.0397055853,0.0569320139],[-0.0381868091,0.0577028805],[-0.0366571629,0.0584971629],[-0.035212257,0.0593693999],[-0.0339701317,0.0603529888],[-0.0330085765,0.0614835765],[-0.0323991912,0.0627834769],[-0.0321613061,0.0642184489],[-0.0323119887,0.0657844887],[-0.0328135134,0.0674117276],[-0.0336156361,0.0690245646],[-0.034641557,0.0705304855]],"A1138":[[-0.0196781264,0.0319114597],[-0.0205089742,0.0323295297],[-0.02172625,0.03293625],[-0.0233535508,0.0337446619],[-0.0253325218,0.0347169663],[-0.0276053384,0.0358108939],[-0.0300899683,0.0369594127],[-0.0326695279,0.0380850834],[-0.0352462515,0.0391195848],[-0.0377460842,0.0400183064],[-0.0400668268,0.0407301601],[-0.0421446679,0.0412302235],[-0.0439474602,0.0415146824],[-0.0454305979,0.0415861535],[-0.0466050886,0.0414756442],[-0.0474691779,0.0412091779],[-0.0480460817,0.0408316372],[-0.0483642993,0.0403809659],[-0.0484500241,0.0398922464],[-0.0483408049,0.0394052493],[-0.0480486146,0.0389286146],[-0.0476129827,0.0384890938],[-0.0470312161,0.0380812161],[-0.0463046984,0.0376930318],[-0.0454318752,0.0373113197],[-0.0444007171,0.036904606],[-0.0432011176,0.036449451],[-0.0418186152,0.0359230597],[-0.0402703729,0.0353131507],[-0.0385704162,0.0346287496],[-0.0367355569,0.0338611124],[-0.0348208068,0.0330408068],[-0.0328721349,0.0322015794],[-0.0309372252,0.0313661141],[-0.0291095883,0.0305912549],[-0.0274296646,0.029894109],[-0.0259501977,0.0293068643],[-0.0247004636,0.0288254636],[-0.0236934001,0.0284389557],[-0.0229135382,0.0281207604],[-0.022341535,0.0278320905],[-0.0219369262,0.0275363706],[-0.0216576849,0.0271910182],[-0.0214675182,0.0267830737],[-0.021334747,0.0262986359],[-0.0212406796,0.0257467907],[-0.0211768347,0.0251618347],[-0.0211268267,0.0245584934],[-0.0211047236,0.0239886125],[-0.0210929594,0.0234785149],[-0.021117889,0.023077889],[-0.0211600866,0.0228067532],[-0.021222692,0.0226893587],[-0.0213056371,0.0227423038],[-0.0213979529,0.0229612863],[-0.0214840433,0.0233351544],[-0.0215662652,0.0238673764],[-0.0216260642,0.024524953],[-0.021665765,0.0252874317],[-0.0216611151,0.0261105595],[-0.0216137,0.0269775889],[-0.0215111765,0.0278311765],[-0.0213490151,0.0286451262],[-0.0211207305,0.0293818416],[-0.0208308727,0.0300153171],[-0.0204879411,0.0305251634],[-0.0201107584,0.0309068695],[-0.0197342967,0.0311659634],[-0.0193943742,0.0313265964],[-0.0191481463,0.0314270352],[-0.0190633394,0.0315161172],[-0.0192226152,0.0316615041]],"A1139":[[-0.0196781264,0.0319114597],[-0.0205089742,0.0323295297],[-0.02172625,0.03293625],[-0.0233535508,0.0337446619],[-0.0253325218,0.0347169663],[-0.0276053384,0.0358108939],[-0.0300899683,0.0369594127],[-0.0326695279,0.0380850834],[-0.0352462515,0.0391195848],[-0.0377460842,0.0400183064],[-0.0400668268,0.0407301601],[-0.0421446679,0.0412302235],[-0.0439474602,0.0415146824],[-0.0454305979,0.0415861535],[-0.0466050886,0.0414756442],[-0.0474691779,0.0412091779],[-0.0480460817,0.0408316372],[-0.0483642993,0.0403809659],[-0.0484500241,0.0398922464],[-0.0483408049,0.0394052493],[-0.0480486146,0.0389286146],[-0.0476129827,0.0384890938],[-0.0470312161,0.0380812161],[-0.0463046984,0.0376930318],[-0.0454318752,0.0373113197],[-0.0444007171,0.036904606],[-0.0432011176,0.036449451],[-0.0418186152,0.0359230597],[-0.0402703729,0.0353131507],[-0.0385704162,0.0346287496],[-0.0367355569,0.0338611124],[-0.0348208068,0.0330408068],[-0.0328721349,0.0322015794],[-0.0309372252,0.0313661141],[-0.0291095883,0.0305912549],[-0.0274296646,0.029894109],[-0.0259501977,0.0293068643],[-0.0247004636,0.0288254636],[-0.0236934001,0.0284389557],[-0.0229135382,0.0281207604],[-0.022341535,0.0278320905],[-0.0219369262,0.0275363706],[-0.0216576849,0.0271910182],[-0.0214675182,0.0267830737],[-0.021334747,0.0262986359],[-0.0212406796,0.0257467907],[-0.0211768347,0.0251618347],[-0.0211268267,0.0245584934],[-0.0211047236,0.0239886125],[-0.0210929594,0.0234785149],[-0.021117889,0.023077889],[-0.0211600866,0.0228067532],[-0.021222692,0.0226893587],[-0.0213056371,0.0227423038],[-0.0213979529,0.0229612863],[-0.0214840433,0.0233351544],[-0.0215662652,0.0238673764],[-0.0216260642,0.024524953],[-0.021665765,0.0252874317],[-0.0216611151,0.0261105595],[-0.0216137,0.0269775889],[-0.0215111765,0.0278311765],[-0.0213490151,0.0286451262],[-0.0211207305,0.0293818416],[-0.0208308727,0.0300153171],[-0.0204879411,0.0305251634],[-0.0201107584,0.0309068695],[-0.0197342967,0.0311659634],[-0.0193943742,0.0313265964],[-0.0191481463,0.0314270352],[-0.0190633394,0.0315161172],[-0.0192226152,0.0316615041]],"A1140":[[-0.035821865,0.071839365],[-0.0370473444,0.0728355586],[-0.0382597229,0.0734518657],[-0.039401731,0.0736328025],[-0.040417054,0.0733334825],[-0.0412987169,0.0725694312],[-0.0420621077,0.0713810363],[-0.0427337695,0.0698523409],[-0.043371275,0.0680773464],[-0.0440229466,0.0661672323],[-0.0447376031,0.0642461745],[-0.0455416788,0.0624102503],[-0.0464382879,0.0607247165],[-0.0474060065,0.0592524351],[-0.048397797,0.0579906541],[-0.0493560527,0.0569235527],[-0.0502032578,0.0560346863],[-0.0508775259,0.0552743117],[-0.051322987,0.0546044155],[-0.0514892243,0.05398351],[-0.0513523665,0.0533802236],[-0.0508990581,0.0527683438],[-0.0501364846,0.0521418417],[-0.0490909571,0.0514995285],[-0.0478082102,0.050859996],[-0.0463716804,0.050278109],[-0.0448616775,0.0498081061],[-0.0433851272,0.04952727],[-0.0420673957,0.0495131099],[-0.0410029728,0.0498354728],[-0.0402739969,0.0505422112],[-0.0399157579,0.0516225436],[-0.039921428,0.0530435709],[-0.040232795,0.0547088664],[-0.0407662796,0.0564894939],[-0.0414125802,0.0582390087],[-0.0420656521,0.0598217236],[-0.0426362598,0.0610991169],[-0.0430455251,0.061970168],[-0.0432611863,0.0623733292],[-0.0432431172,0.0622591887],[-0.0430202662,0.0616456233],[-0.0426253659,0.0605817944],[-0.0421132565,0.0591507565],[-0.0415622807,0.0574726378],[-0.0410586398,0.0556836398],[-0.0406875532,0.0539446961],[-0.0405155771,0.0523937914],[-0.040588337,0.0511504799],[-0.0409074899,0.0503085614],[-0.0414272976,0.0498776547],[-0.0420792198,0.0498581483],[-0.0427617185,0.0502010042],[-0.0433544117,0.0508161974],[-0.0437615103,0.051625796],[-0.0438952835,0.0525402835],[-0.0437051226,0.0534933369],[-0.0431605075,0.0544308646],[-0.0422749302,0.0553124302],[-0.0411036951,0.0561429808],[-0.0397055853,0.0569320139],[-0.0381868091,0.0577028805],[-0.0366571629,0.0584971629],[-0.035212257,0.0593693999],[-0.0339701317,0.0603529888],[-0.0330085765,0.0614835765],[-0.0323991912,0.0627834769],[-0.0321613061,0.0642184489],[-0.0323119887,0.0657844887],[-0.0328135134,0.0674117276],[-0.0336156361,0.0690245646],[-0.034641557,0.0705304855]],"A1141":[[-0.0196781264,0.0319114597],[-0.0205089742,0.0323295297],[-0.02172625,0.03293625],[-0.0233535508,0.0337446619],[-0.0253325218,0.0347169663],[-0.0276053384,0.0358108939],[-0.0300899683,0.0369594127],[-0.0326695279,0.0380850834],[-0.0352462515,0.0391195848],[-0.0377460842,0.0400183064],[-0.0400668268,0.0407301601],[-0.0421446679,0.0412302235],[-0.0439474602,0.0415146824],[-0.0454305979,0.0415861535],[-0.0466050886,0.0414756442],[-0.0474691779,0.0412091779],[-0.0480460817,0.0408316372],[-0.0483642993,0.0403809659],[-0.0484500241,0.0398922464],[-0.0483408049,0.0394052493],[-0.0480486146,0.0389286146],[-0.0476129827,0.0384890938],[-0.0470312161,0.0380812161],[-0.0463046984,0.0376930318],[-0.0454318752,0.0373113197],[-0.0444007171,0.036904606],[-0.0432011176,0.036449451],[-0.0418186152,0.0359230597],[-0.0402703729,0.0353131507],[-0.0385704162,0.0346287496],[-0.0367355569,0.0338611124],[-0.0348208068,0.0330408068],[-0.0328721349,0.0322015794],[-0.0309372252,0.0313661141],[-0.0291095883,0.0305912549],[-0.0274296646,0.029894109],[-0.0259501977,0.0293068643],[-0.0247004636,0.0288254636],[-0.0236934001,0.0284389557],[-0.0229135382,0.0281207604],[-0.022341535,0.0278320905],[-0.0219369262,0.0275363706],[-0.0216576849,0.0271910182],[-0.0214675182,0.0267830737],[-0.021334747,0.0262986359],[-0.0212406796,0.0257467907],[-0.0211768347,0.0251618347],[-0.0211268267,0.0245584934],[-0.0211047236,0.0239886125],[-0.0210929594,0.0234785149],[-0.021117889,0.023077889],[-0.0211600866,0.0228067532],[-0.021222692,0.0226893587],[-0.0213056371,0.0227423038],[-0.0213979529,0.0229612863],[-0.0214840433,0.0233351544],[-0.0215662652,0.0238673764],[-0.0216260642,0.024524953],[-0.021665765,0.0252874317],[-0.0216611151,0.0261105595],[-0.0216137,0.0269775889],[-0.0215111765,0.0278311765],[-0.0213490151,0.0286451262],[-0.0211207305,0.0293818416],[-0.0208308727,0.0300153171],[-0.0204879411,0.0305251634],[-0.0201107584,0.0309068695],[-0.0197342967,0.0311659634],[-0.0193943742,0.0313265964],[-0.0191481463,0.0314270352],[-0.0190633394,0.0315161172],[-0.0192226152,0.0316615041]],"B1021":[[-0.035821865,0.071839365],[-0.0370473444,0.0728355586],[-0.0382597229,0.0734518657],[-0.039401731,0.0736328025],[-0.040417054,0.0733334825],[-0.0412987169,0.0725694312],[-0.0420621077,0.0713810363],[-0.0427337695,0.0698523409],[-0.043371275,0.0680773464],[-0.0440229466,0.0661672323],[-0.0447376031,0.0642461745],[-0.0455416788,0.0624102503],[-0.0464382879,0.0607247165],[-0.0474060065,0.0592524351],[-0.048397797,0.0579906541],[-0.0493560527,0.0569235527],[-0.0502032578,0.0560346863],[-0.0508775259,0.0552743117],[-0.051322987,0.0546044155],[-0.0514892243,0.05398351],[-0.0513523665,0.0533802236],[-0.0508990581,0.0527683438],[-0.0501364846,0.0521418417],[-0.0490909571,0.0514995285],[-0.0478082102,0.050859996],[-0.0463716804,0.050278109],[-0.0448616775,0.0498081061],[-0.0433851272,0.04952727],[-0.0420673957,0.0495131099],[-0.0410029728,0.0498354728],[-0.0402739969,0.0505422112],[-0.0399157579,0.0516225436],[-0.039921428,0.0530435709],[-0.040232795,0.0547088664],[-0.0407662796,0.0564894939],[-0.0414125802,0.0582390087],[-0.0420656521,0.0598217236],[-0.0426362598,0.0610991169],[-0.0430455251,0.061970168],[-0.0432611863,0.0623733292],[-0.0432431172,0.0622591887],[-0.0430202662,0.0616456233],[-0.0426253659,0.0605817944],[-0.0421132565,0.0591507565],[-0.0415622807,0.0574726378],[-0.0410586398,0.0556836398],[-0.0406875532,0.0539446961],[-0.0405155771,0.0523937914],[-0.040588337,0.0511504799],[-0.0409074899,0.0503085614],[-0.0414272976,0.0498776547],[-0.0420792198,0.0498581483],[-0.0427617185,0.0502010042],[-0.0433544117,0.0508161974],[-0.0437615103,0.051625796],[-0.0438952835,0.0525402835],[-0.0437051226,0.0534933369],[-0.0431605075,0.0544308646],[-0.0422749302,0.0553124302],[-0.0411036951,0.0561429808],[-0.0397055853,0.0569320139],[-0.0381868091,0.0577028805],[-0.0366571629,0.0584971629],[-0.035212257,0.0593693999],[-0.0339701317,0.0603529888],[-0.0330085765,0.0614835765],[-0.0323991912,0.0627834769],[-0.0321613061,0.0642184489],[-0.0323119887,0.0657844887],[-0.0328135134,0.0674117276],[-0.0336156361,0.0690245646],[-0.034641557,0.0705304855]],"B1022":[[-0.035821865,0.071839365],[-0.0370473444,0.0728355586],[-0.0382597229,0.0734518657],[-0.039401731,0.0736328025],[-0.040417054,0.0733334825],[-0.0412987169,0.0725694312],[-0.0420621077,0.0713810363],[-0.0427337695,0.0698523409],[-0.043371275,0.0680773464],[-0.0440229466,0.0661672323],[-0.0447376031,0.0642461745],[-0.0455416788,0.0624102503],[-0.0464382879,0.0607247165],[-0.0474060065,0.0592524351],[-0.048397797,0.0579906541],[-0.0493560527,0.0569235527],[-0.0502032578,0.0560346863],[-0.0508775259,0.0552743117],[-0.051322987,0.0546044155],[-0.0514892243,0.05398351],[-0.0513523665,0.0533802236],[-0.0508990581,0.0527683438],[-0.0501364846,0.0521418417],[-0.0490909571,0.0514995285],[-0.0478082102,0.050859996],[-0.0463716804,0.050278109],[-0.0448616775,0.0498081061],[-0.0433851272,0.04952727],[-0.0420673957,0.0495131099],[-0.0410029728,0.0498354728],[-0.0402739969,0.0505422112],[-0.0399157579,0.0516225436],[-0.039921428,0.0530435709],[-0.040232795,0.0547088664],[-0.0407662796,0.0564894939],[-0.0414125802,0.0582390087],[-0.0420656521,0.0598217236],[-0.0426362598,0.0610991169],[-0.0430455251,0.061970168],[-0.0432611863,0.0623733292],[-0.0432431172,0.0622591887],[-0.0430202662,0.0616456233],[-0.0426253659,0.0605817944],[-0.0421132565,0.0591507565],[-0.0415622807,0.0574726378],[-0.0410586398,0.0556836398],[-0.0406875532,0.0539446961],[-0.0405155771,0.0523937914],[-0.040588337,0.0511504799],[-0.0409074899,0.0503085614],[-0.0414272976,0.0498776547],[-0.0420792198,0.0498581483],[-0.0427617185,0.0502010042],[-0.0433544117,0.0508161974],[-0.0437615103,0.051625796],[-0.0438952835,0.0525402835],[-0.0437051226,0.0534933369],[-0.0431605075,0.0544308646],[-0.0422749302,0.0553124302],[-0.0411036951,0.0561429808],[-0.0397055853,0.0569320139],[-0.0381868091,0.0577028805],[-0.0366571629,0.0584971629],[-0.035212257,0.0593693999],[-0.0339701317,0.0603529888],[-0.0330085765,0.0614835765],[-0.0323991912,0.0627834769],[-0.0321613061,0.0642184489],[-0.0323119887,0.0657844887],[-0.0328135134,0.0674117276],[-0.0336156361,0.0690245646],[-0.034641557,0.0705304855]],"B1023":[[-0.035821865,0.071839365],[-0.0370473444,0.0728355586],[-0.0382597229,0.0734518657],[-0.039401731,0.0736328025],[-0.040417054,0.0733334825],[-0.0412987169,0.0725694312],[-0.0420621077,0.0713810363],[-0.0427337695,0.0698523409],[-0.043371275,0.0680773464],[-0.0440229466,0.0661672323],[-0.0447376031,0.0642461745],[-0.0455416788,0.0624102503],[-0.0464382879,0.0607247165],[-0.0474060065,0.0592524351],[-0.048397797,0.0579906541],[-0.0493560527,0.0569235527],[-0.0502032578,0.0560346863],[-0.0508775259,0.0552743117],[-0.051322987,0.0546044155],[-0.0514892243,0.05398351],[-0.0513523665,0.0533802236],[-0.0508990581,0.0527683438],[-0.0501364846,0.0521418417],[-0.0490909571,0.0514995285],[-0.0478082102,0.050859996],[-0.0463716804,0.050278109],[-0.0448616775,0.0498081061],[-0.0433851272,0.04952727],[-0.0420673957,0.0495131099],[-0.0410029728,0.0498354728],[-0.0402739969,0.0505422112],[-0.0399157579,0.0516225436],[-0.039921428,0.0530435709],[-0.040232795,0.0547088664],[-0.0407662796,0.0564894939],[-0.0414125802,0.0582390087],[-0.0420656521,0.0598217236],[-0.0426362598,0.0610991169],[-0.0430455251,0.061970168],[-0.0432611863,0.0623733292],[-0.0432431172,0.0622591887],[-0.0430202662,0.0616456233],[-0.0426253659,0.0605817944],[-0.0421132565,0.0591507565],[-0.0415622807,0.0574726378],[-0.0410586398,0.0556836398],[-0.0406875532,0.0539446961],[-0.0405155771,0.0523937914],[-0.040588337,0.0511504799],[-0.0409074899,0.0503085614],[-0.0414272976,0.0498776547],[-0.0420792198,0.0498581483],[-0.0427617185,0.0502010042],[-0.0433544117,0.0508161974],[-0.0437615103,0.051625796],[-0.0438952835,0.0525402835],[-0.0437051226,0.0534933369],[-0.0431605075,0.0544308646],[-0.0422749302,0.0553124302],[-0.0411036951,0.0561429808],[-0.0397055853,0.0569320139],[-0.0381868091,0.0577028805],[-0.0366571629,0.0584971629],[-0.035212257,0.0593693999],[-0.0339701317,0.0603529888],[-0.0330085765,0.0614835765],[-0.0323991912,0.0627834769],[-0.0321613061,0.0642184489],[-0.0323119887,0.0657844887],[-0.0328135134,0.0674117276],[-0.0336156361,0.0690245646],[-0.034641557,0.0705304855]],"B1201":[[-0.035821865,0.071839365],[-0.0370473444,0.0728355586],[-0.0382597229,0.0734518657],[-0.039401731,0.0736328025],[-0.040417054,0.0733334825],[-0.0412987169,0.0725694312],[-0.0420621077,0.0713810363],[-0.0427337695,0.0698523409],[-0.043371275,0.0680773464],[-0.0440229466,0.0661672323],[-0.0447376031,0.0642461745],[-0.0455416788,0.0624102503],[-0.0464382879,0.0607247165],[-0.0474060065,0.0592524351],[-0.048397797,0.0579906541],[-0.0493560527,0.0569235527],[-0.0502032578,0.0560346863],[-0.0508775259,0.0552743117],[-0.051322987,0.0546044155],[-0.0514892243,0.05398351],[-0.0513523665,0.0533802236],[-0.0508990581,0.0527683438],[-0.0501364846,0.0521418417],[-0.0490909571,0.0514995285],[-0.0478082102,0.050859996],[-0.0463716804,0.050278109],[-0.0448616775,0.0498081061],[-0.0433851272,0.04952727],[-0.0420673957,0.0495131099],[-0.0410029728,0.0498354728],[-0.0402739969,0.0505422112],[-0.0399157579,0.0516225436],[-0.039921428,0.0530435709],[-0.040232795,0.0547088664],[-0.0407662796,0.0564894939],[-0.0414125802,0.0582390087],[-0.0420656521,0.0598217236],[-0.0426362598,0.0610991169],[-0.0430455251,0.061970168],[-0.0432611863,0.0623733292],[-0.0432431172,0.0622591887],[-0.0430202662,0.0616456233],[-0.0426253659,0.0605817944],[-0.0421132565,0.0591507565],[-0.0415622807,0.0574726378],[-0.0410586398,0.0556836398],[-0.0406875532,0.0539446961],[-0.0405155771,0.0523937914],[-0.040588337,0.0511504799],[-0.0409074899,0.0503085614],[-0.0414272976,0.0498776547],[-0.0420792198,0.0498581483],[-0.0427617185,0.0502010042],[-0.0433544117,0.0508161974],[-0.0437615103,0.051625796],[-0.0438952835,0.0525402835],[-0.0437051226,0.0534933369],[-0.0431605075,0.0544308646],[-0.0422749302,0.0553124302],[-0.0411036951,0.0561429808],[-0.0397055853,0.0569320139],[-0.0381868091,0.0577028805],[-0.0366571629,0.0584971629],[-0.035212257,0.0593693999],[-0.0339701317,0.0603529888],[-0.0330085765,0.0614835765],[-0.0323991912,0.0627834769],[-0.0321613061,0.0642184489],[-0.0323119887,0.0657844887],[-0.0328135134,0.0674117276],[-0.0336156361,0.0690245646],[-0.034641557,0.0705304855]],"B1202":[[-0.035821865,0.071839365],[-0.0370473444,0.0728355586],[-0.0382597229,0.0734518657],[-0.039401731,0.0736328025],[-0.040417054,0.0733334825],[-0.0412987169,0.0725694312],[-0.0420621077,0.0713810363],[-0.0427337695,0.0698523409],[-0.043371275,0.0680773464],[-0.0440229466,0.0661672323],[-0.0447376031,0.0642461745],[-0.0455416788,0.0624102503],[-0.0464382879,0.0607247165],[-0.0474060065,0.0592524351],[-0.048397797,0.0579906541],[-0.0493560527,0.0569235527],[-0.0502032578,0.0560346863],[-0.0508775259,0.0552743117],[-0.051322987,0.0546044155],[-0.0514892243,0.05398351],[-0.0513523665,0.0533802236],[-0.0508990581,0.0527683438],[-0.0501364846,0.0521418417],[-0.0490909571,0.0514995285],[-0.0478082102,0.050859996],[-0.0463716804,0.050278109],[-0.0448616775,0.0498081061],[-0.0433851272,0.04952727],[-0.0420673957,0.0495131099],[-0.0410029728,0.0498354728],[-0.0402739969,0.0505422112],[-0.0399157579,0.0516225436],[-0.039921428,0.0530435709],[-0.040232795,0.0547088664],[-0.0407662796,0.0564894939],[-0.0414125802,0.0582390087],[-0.0420656521,0.0598217236],[-0.0426362598,0.0610991169],[-0.0430455251,0.061970168],[-0.0432611863,0.0623733292],[-0.0432431172,0.0622591887],[-0.0430202662,0.0616456233],[-0.0426253659,0.0605817944],[-0.0421132565,0.0591507565],[-0.0415622807,0.0574726378],[-0.0410586398,0.0556836398],[-0.0406875532,0.0539446961],[-0.0405155771,0.0523937914],[-0.040588337,0.0511504799],[-0.0409074899,0.0503085614],[-0.0414272976,0.0498776547],[-0.0420792198,0.0498581483],[-0.0427617185,0.0502010042],[-0.0433544117,0.0508161974],[-0.0437615103,0.051625796],[-0.0438952835,0.0525402835],[-0.0437051226,0.0534933369],[-0.0431605075,0.0544308646],[-0.0422749302,0.0553124302],[-0.0411036951,0.0561429808],[-0.0397055853,0.0569320139],[-0.0381868091,0.0577028805],[-0.0366571629,0.0584971629],[-0.035212257,0.0593693999],[-0.0339701317,0.0603529888],[-0.0330085765,0.0614835765],[-0.0323991912,0.0627834769],[-0.0321613061,0.0642184489],[-0.0323119887,0.0657844887],[-0.0328135134,0.0674117276],[-0.0336156361,0.0690245646],[-0.034641557,0.0705304855]],"B1203":[[-0.035821865,0.071839365],[-0.0370473444,0.0728355586],[-0.0382597229,0.0734518657],[-0.039401731,0.0736328025],[-0.040417054,0.0733334825],[-0.0412987169,0.0725694312],[-0.0420621077,0.0713810363],[-0.0427337695,0.0698523409],[-0.043371275,0.0680773464],[-0.0440229466,0.0661672323],[-0.0447376031,0.0642461745],[-0.0455416788,0.0624102503],[-0.0464382879,0.0607247165],[-0.0474060065,0.0592524351],[-0.048397797,0.0579906541],[-0.0493560527,0.0569235527],[-0.0502032578,0.0560346863],[-0.0508775259,0.0552743117],[-0.051322987,0.0546044155],[-0.0514892243,0.05398351],[-0.0513523665,0.0533802236],[-0.0508990581,0.0527683438],[-0.0501364846,0.0521418417],[-0.0490909571,0.0514995285],[-0.0478082102,0.050859996],[-0.0463716804,0.050278109],[-0.0448616775,0.0498081061],[-0.0433851272,0.04952727],[-0.0420673957,0.0495131099],[-0.0410029728,0.0498354728],[-0.0402739969,0.0505422112],[-0.0399157579,0.0516225436],[-0.039921428,0.0530435709],[-0.040232795,0.0547088664],[-0.0407662796,0.0564894939],[-0.0414125802,0.0582390087],[-0.0420656521,0.0598217236],[-0.0426362598,0.0610991169],[-0.0430455251,0.061970168],[-0.0432611863,0.0623733292],[-0.0432431172,0.0622591887],[-0.0430202662,0.0616456233],[-0.0426253659,0.0605817944],[-0.0421132565,0.0591507565],[-0.0415622807,0.0574726378],[-0.0410586398,0.0556836398],[-0.0406875532,0.0539446961],[-0.0405155771,0.0523937914],[-0.040588337,0.0511504799],[-0.0409074899,0.0503085614],[-0.0414272976,0.0498776547],[-0.0420792198,0.0498581483],[-0.0427617185,0.0502010042],[-0.0433544117,0.0508161974],[-0.0437615103,0.051625796],[-0.0438952835,0.0525402835],[-0.0437051226,0.0534933369],[-0.0431605075,0.0544308646],[-0.0422749302,0.0553124302],[-0.0411036951,0.0561429808],[-0.0397055853,0.0569320139],[-0.0381868091,0.0577028805],[-0.0366571629,0.0584971629],[-0.035212257,0.0593693999],[-0.0339701317,0.0603529888],[-0.0330085765,0.0614835765],[-0.0323991912,0.0627834769],[-0.0321613061,0.0642184489],[-0.0323119887,0.0657844887],[-0.0328135134,0.0674117276],[-0.0336156361,0.0690245646],[-0.034641557,0.0705304855]],"B1204":[[-0.035821865,0.071839365],[-0.0370473444,0.0728355586],[-0.0382597229,0.0734518657],[-0.039401731,0.0736328025],[-0.040417054,0.0733334825],[-0.0412987169,0.0725694312],[-0.0420621077,0.0713810363],[-0.0427337695,0.0698523409],[-0.043371275,0.0680773464],[-0.0440229466,0.0661672323],[-0.0447376031,0.0642461745],[-0.0455416788,0.0624102503],[-0.0464382879,0.0607247165],[-0.0474060065,0.0592524351],[-0.048397797,0.0579906541],[-0.0493560527,0.0569235527],[-0.0502032578,0.0560346863],[-0.0508775259,0.0552743117],[-0.051322987,0.0546044155],[-0.0514892243,0.05398351],[-0.0513523665,0.0533802236],[-0.0508990581,0.0527683438],[-0.0501364846,0.0521418417],[-0.0490909571,0.0514995285],[-0.0478082102,0.050859996],[-0.0463716804,0.050278109],[-0.0448616775,0.0498081061],[-0.0433851272,0.04952727],[-0.0420673957,0.0495131099],[-0.0410029728,0.0498354728],[-0.0402739969,0.0505422112],[-0.0399157579,0.0516225436],[-0.039921428,0.0530435709],[-0.040232795,0.0547088664],[-0.0407662796,0.0564894939],[-0.0414125802,0.0582390087],[-0.0420656521,0.0598217236],[-0.0426362598,0.0610991169],[-0.0430455251,0.061970168],[-0.0432611863,0.0623733292],[-0.0432431172,0.0622591887],[-0.0430202662,0.0616456233],[-0.0426253659,0.0605817944],[-0.0421132565,0.0591507565],[-0.0415622807,0.0574726378],[-0.0410586398,0.0556836398],[-0.0406875532,0.0539446961],[-0.0405155771,0.0523937914],[-0.040588337,0.0511504799],[-0.0409074899,0.0503085614],[-0.0414272976,0.0498776547],[-0.0420792198,0.0498581483],[-0.0427617185,0.0502010042],[-0.0433544117,0.0508161974],[-0.0437615103,0.051625796],[-0.0438952835,0.0525402835],[-0.0437051226,0.0534933369],[-0.0431605075,0.0544308646],[-0.0422749302,0.0553124302],[-0.0411036951,0.0561429808],[-0.0397055853,0.0569320139],[-0.0381868091,0.0577028805],[-0.0366571629,0.0584971629],[-0.035212257,0.0593693999],[-0.0339701317,0.0603529888],[-0.0330085765,0.0614835765],[-0.0323991912,0.0627834769],[-0.0321613061,0.0642184489],[-0.0323119887,0.0657844887],[-0.0328135134,0.0674117276],[-0.0336156361,0.0690245646],[-0.034641557,0.0705304855]],"B1205":[[-0.035821865,0.071839365],[-0.0370473444,0.0728355586],[-0.0382597229,0.0734518657],[-0.039401731,0.0736328025],[-0.040417054,0.0733334825],[-0.0412987169,0.0725694312],[-0.0420621077,0.0713810363],[-0.0427337695,0.0698523409],[-0.043371275,0.0680773464],[-0.0440229466,0.0661672323],[-0.0447376031,0.0642461745],[-0.0455416788,0.0624102503],[-0.0464382879,0.0607247165],[-0.0474060065,0.0592524351],[-0.048397797,0.0579906541],[-0.0493560527,0.0569235527],[-0.0502032578,0.0560346863],[-0.0508775259,0.0552743117],[-0.051322987,0.0546044155],[-0.0514892243,0.05398351],[-0.0513523665,0.0533802236],[-0.0508990581,0.0527683438],[-0.0501364846,0.0521418417],[-0.0490909571,0.0514995285],[-0.0478082102,0.050859996],[-0.0463716804,0.050278109],[-0.0448616775,0.0498081061],[-0.0433851272,0.04952727],[-0.0420673957,0.0495131099],[-0.0410029728,0.0498354728],[-0.0402739969,0.0505422112],[-0.0399157579,0.0516225436],[-0.039921428,0.0530435709],[-0.040232795,0.0547088664],[-0.0407662796,0.0564894939],[-0.0414125802,0.0582390087],[-0.0420656521,0.0598217236],[-0.0426362598,0.0610991169],[-0.0430455251,0.061970168],[-0.0432611863,0.0623733292],[-0.0432431172,0.0622591887],[-0.0430202662,0.0616456233],[-0.0426253659,0.0605817944],[-0.0421132565,0.0591507565],[-0.0415622807,0.0574726378],[-0.0410586398,0.0556836398],[-0.0406875532,0.0539446961],[-0.0405155771,0.0523937914],[-0.040588337,0.0511504799],[-0.0409074899,0.0503085614],[-0.0414272976,0.0498776547],[-0.0420792198,0.0498581483],[-0.0427617185,0.0502010042],[-0.0433544117,0.0508161974],[-0.0437615103,0.051625796],[-0.0438952835,0.0525402835],[-0.0437051226,0.0534933369],[-0.0431605075,0.0544308646],[-0.0422749302,0.0553124302],[-0.0411036951,0.0561429808],[-0.0397055853,0.0569320139],[-0.0381868091,0.0577028805],[-0.0366571629,0.0584971629],[-0.035212257,0.0593693999],[-0.0339701317,0.0603529888],[-0.0330085765,0.0614835765],[-0.0323991912,0.0627834769],[-0.0321613061,0.0642184489],[-0.0323119887,0.0657844887],[-0.0328135134,0.0674117276],[-0.0336156361,0.0690245646],[-0.034641557,0.0705304855]],"B1206":[[-0.035821865,0.071839365],[-0.0370473444,0.0728355586],[-0.0382597229,0.0734518657],[-0.039401731,0.0736328025],[-0.040417054,0.0733334825],[-0.0412987169,0.0725694312],[-0.0420621077,0.0713810363],[-0.0427337695,0.0698523409],[-0.043371275,0.0680773464],[-0.0440229466,0.0661672323],[-0.0447376031,0.0642461745],[-0.0455416788,0.0624102503],[-0.0464382879,0.0607247165],[-0.0474060065,0.0592524351],[-0.048397797,0.0579906541],[-0.0493560527,0.0569235527],[-0.0502032578,0.0560346863],[-0.0508775259,0.0552743117],[-0.051322987,0.0546044155],[-0.0514892243,0.05398351],[-0.0513523665,0.0533802236],[-0.0508990581,0.0527683438],[-0.0501364846,0.0521418417],[-0.0490909571,0.0514995285],[-0.0478082102,0.050859996],[-0.0463716804,0.050278109],[-0.0448616775,0.0498081061],[-0.0433851272,0.04952727],[-0.0420673957,0.0495131099],[-0.0410029728,0.0498354728],[-0.0402739969,0.0505422112],[-0.0399157579,0.0516225436],[-0.039921428,0.0530435709],[-0.040232795,0.0547088664],[-0.0407662796,0.0564894939],[-0.0414125802,0.0582390087],[-0.0420656521,0.0598217236],[-0.0426362598,0.0610991169],[-0.0430455251,0.061970168],[-0.0432611863,0.0623733292],[-0.0432431172,0.0622591887],[-0.0430202662,0.0616456233],[-0.0426253659,0.0605817944],[-0.0421132565,0.0591507565],[-0.0415622807,0.0574726378],[-0.0410586398,0.0556836398],[-0.0406875532,0.0539446961],[-0.0405155771,0.0523937914],[-0.040588337,0.0511504799],[-0.0409074899,0.0503085614],[-0.0414272976,0.0498776547],[-0.0420792198,0.0498581483],[-0.0427617185,0.0502010042],[-0.0433544117,0.0508161974],[-0.0437615103,0.051625796],[-0.0438952835,0.0525402835],[-0.0437051226,0.0534933369],[-0.0431605075,0.0544308646],[-0.0422749302,0.0553124302],[-0.0411036951,0.0561429808],[-0.0397055853,0.0569320139],[-0.0381868091,0.0577028805],[-0.0366571629,0.0584971629],[-0.035212257,0.0593693999],[-0.0339701317,0.0603529888],[-0.0330085765,0.0614835765],[-0.0323991912,0.0627834769],[-0.0321613061,0.0642184489],[-0.0323119887,0.0657844887],[-0.0328135134,0.0674117276],[-0.0336156361,0.0690245646],[-0.034641557,0.0705304855]],"B1207":[[-0.035821865,0.071839365],[-0.0370473444,0.0728355586],[-0.0382597229,0.0734518657],[-0.039401731,0.0736328025],[-0.040417054,0.0733334825],[-0.0412987169,0.0725694312],[-0.0420621077,0.0713810363],[-0.0427337695,0.0698523409],[-0.043371275,0.0680773464],[-0.0440229466,0.0661672323],[-0.0447376031,0.0642461745],[-0.0455416788,0.0624102503],[-0.0464382879,0.0607247165],[-0.0474060065,0.0592524351],[-0.048397797,0.0579906541],[-0.0493560527,0.0569235527],[-0.0502032578,0.0560346863],[-0.0508775259,0.0552743117],[-0.051322987,0.0546044155],[-0.0514892243,0.05398351],[-0.0513523665,0.0533802236],[-0.0508990581,0.0527683438],[-0.0501364846,0.0521418417],[-0.0490909571,0.0514995285],[-0.0478082102,0.050859996],[-0.0463716804,0.050278109],[-0.0448616775,0.0498081061],[-0.0433851272,0.04952727],[-0.0420673957,0.0495131099],[-0.0410029728,0.0498354728],[-0.0402739969,0.0505422112],[-0.0399157579,0.0516225436],[-0.039921428,0.0530435709],[-0.040232795,0.0547088664],[-0.0407662796,0.0564894939],[-0.0414125802,0.0582390087],[-0.0420656521,0.0598217236],[-0.0426362598,0.0610991169],[-0.0430455251,0.061970168],[-0.0432611863,0.0623733292],[-0.0432431172,0.0622591887],[-0.0430202662,0.0616456233],[-0.0426253659,0.0605817944],[-0.0421132565,0.0591507565],[-0.0415622807,0.0574726378],[-0.0410586398,0.0556836398],[-0.0406875532,0.0539446961],[-0.0405155771,0.0523937914],[-0.040588337,0.0511504799],[-0.0409074899,0.0503085614],[-0.0414272976,0.0498776547],[-0.0420792198,0.0498581483],[-0.0427617185,0.0502010042],[-0.0433544117,0.0508161974],[-0.0437615103,0.051625796],[-0.0438952835,0.0525402835],[-0.0437051226,0.0534933369],[-0.0431605075,0.0544308646],[-0.0422749302,0.0553124302],[-0.0411036951,0.0561429808],[-0.0397055853,0.0569320139],[-0.0381868091,0.0577028805],[-0.0366571629,0.0584971629],[-0.035212257,0.0593693999],[-0.0339701317,0.0603529888],[-0.0330085765,0.0614835765],[-0.0323991912,0.0627834769],[-0.0321613061,0.0642184489],[-0.0323119887,0.0657844887],[-0.0328135134,0.0674117276],[-0.0336156361,0.0690245646],[-0.034641557,0.0705304855]],"B1208":[[-0.035821865,0.071839365],[-0.0370473444,0.0728355586],[-0.0382597229,0.0734518657],[-0.039401731,0.0736328025],[-0.040417054,0.0733334825],[-0.0412987169,0.0725694312],[-0.0420621077,0.0713810363],[-0.0427337695,0.0698523409],[-0.043371275,0.0680773464],[-0.0440229466,0.0661672323],[-0.0447376031,0.0642461745],[-0.0455416788,0.0624102503],[-0.0464382879,0.0607247165],[-0.0474060065,0.0592524351],[-0.048397797,0.0579906541],[-0.0493560527,0.0569235527],[-0.0502032578,0.0560346863],[-0.0508775259,0.0552743117],[-0.051322987,0.0546044155],[-0.0514892243,0.05398351],[-0.0513523665,0.0533802236],[-0.0508990581,0.0527683438],[-0.0501364846,0.0521418417],[-0.0490909571,0.0514995285],[-0.0478082102,0.050859996],[-0.0463716804,0.050278109],[-0.0448616775,0.0498081061],[-0.0433851272,0.04952727],[-0.0420673957,0.0495131099],[-0.0410029728,0.0498354728],[-0.0402739969,0.0505422112],[-0.0399157579,0.0516225436],[-0.039921428,0.0530435709],[-0.040232795,0.0547088664],[-0.0407662796,0.0564894939],[-0.0414125802,0.0582390087],[-0.0420656521,0.0598217236],[-0.0426362598,0.0610991169],[-0.0430455251,0.061970168],[-0.0432611863,0.0623733292],[-0.0432431172,0.0622591887],[-0.0430202662,0.0616456233],[-0.0426253659,0.0605817944],[-0.0421132565,0.0591507565],[-0.0415622807,0.0574726378],[-0.0410586398,0.0556836398],[-0.0406875532,0.0539446961],[-0.0405155771,0.0523937914],[-0.040588337,0.0511504799],[-0.0409074899,0.0503085614],[-0.0414272976,0.0498776547],[-0.0420792198,0.0498581483],[-0.0427617185,0.0502010042],[-0.0433544117,0.0508161974],[-0.0437615103,0.051625796],[-0.0438952835,0.0525402835],[-0.0437051226,0.0534933369],[-0.0431605075,0.0544308646],[-0.0422749302,0.0553124302],[-0.0411036951,0.0561429808],[-0.0397055853,0.0569320139],[-0.0381868091,0.0577028805],[-0.0366571629,0.0584971629],[-0.035212257,0.0593693999],[-0.0339701317,0.0603529888],[-0.0330085765,0.0614835765],[-0.0323991912,0.0627834769],[-0.0321613061,0.0642184489],[-0.0323119887,0.0657844887],[-0.0328135134,0.0674117276],[-0.0336156361,0.0690245646],[-0.034641557,0.0705304855]],"B1209":[[-0.0196781264,0.0319114597],[-0.0205089742,0.0323295297],[-0.02172625,0.03293625],[-0.0233535508,0.0337446619],[-0.0253325218,0.0347169663],[-0.0276053384,0.0358108939],[-0.0300899683,0.0369594127],[-0.0326695279,0.0380850834],[-0.0352462515,0.0391195848],[-0.0377460842,0.0400183064],[-0.0400668268,0.0407301601],[-0.0421446679,0.0412302235],[-0.0439474602,0.0415146824],[-0.0454305979,0.0415861535],[-0.0466050886,0.0414756442],[-0.0474691779,0.0412091779],[-0.0480460817,0.0408316372],[-0.0483642993,0.0403809659],[-0.0484500241,0.0398922464],[-0.0483408049,0.0394052493],[-0.0480486146,0.0389286146],[-0.0476129827,0.0384890938],[-0.0470312161,0.0380812161],[-0.0463046984,0.0376930318],[-0.0454318752,0.0373113197],[-0.0444007171,0.036904606],[-0.0432011176,0.036449451],[-0.0418186152,0.0359230597],[-0.0402703729,0.0353131507],[-0.0385704162,0.0346287496],[-0.0367355569,0.0338611124],[-0.0348208068,0.0330408068],[-0.0328721349,0.0322015794],[-0.0309372252,0.0313661141],[-0.0291095883,0.0305912549],[-0.0274296646,0.029894109],[-0.0259501977,0.0293068643],[-0.0247004636,0.0288254636],[-0.0236934001,0.0284389557],[-0.0229135382,0.0281207604],[-0.022341535,0.0278320905],[-0.0219369262,0.0275363706],[-0.0216576849,0.0271910182],[-0.0214675182,0.0267830737],[-0.021334747,0.0262986359],[-0.0212406796,0.0257467907],[-0.0211768347,0.0251618347],[-0.0211268267,0.0245584934],[-0.0211047236,0.0239886125],[-0.0210929594,0.0234785149],[-0.021117889,0.023077889],[-0.0211600866,0.0228067532],[-0.021222692,0.0226893587],[-0.0213056371,0.0227423038],[-0.0213979529,0.0229612863],[-0.0214840433,0.0233351544],[-0.0215662652,0.0238673764],[-0.0216260642,0.024524953],[-0.021665765,0.0252874317],[-0.0216611151,0.0261105595],[-0.0216137,0.0269775889],[-0.0215111765,0.0278311765],[-0.0213490151,0.0286451262],[-0.0211207305,0.0293818416],[-0.0208308727,0.0300153171],[-0.0204879411,0.0305251634],[-0.0201107584,0.0309068695],[-0.0197342967,0.0311659634],[-0.0193943742,0.0313265964],[-0.0191481463,0.0314270352],[-0.0190633394,0.0315161172],[-0.0192226152,0.0316615041]],"B1210":[[-0.035821865,0.071839365],[-0.0370473444,0.0728355586],[-0.0382597229,0.0734518657],[-0.039401731,0.0736328025],[-0.040417054,0.0733334825],[-0.0412987169,0.0725694312],[-0.0420621077,0.0713810363],[-0.0427337695,0.0698523409],[-0.043371275,0.0680773464],[-0.0440229466,0.0661672323],[-0.0447376031,0.0642461745],[-0.0455416788,0.0624102503],[-0.0464382879,0.0607247165],[-0.0474060065,0.0592524351],[-0.048397797,0.0579906541],[-0.0493560527,0.0569235527],[-0.0502032578,0.0560346863],[-0.0508775259,0.0552743117],[-0.051322987,0.0546044155],[-0.0514892243,0.05398351],[-0.0513523665,0.0533802236],[-0.0508990581,0.0527683438],[-0.0501364846,0.0521418417],[-0.0490909571,0.0514995285],[-0.0478082102,0.050859996],[-0.0463716804,0.050278109],[-0.0448616775,0.0498081061],[-0.0433851272,0.04952727],[-0.0420673957,0.0495131099],[-0.0410029728,0.0498354728],[-0.0402739969,0.0505422112],[-0.0399157579,0.0516225436],[-0.039921428,0.0530435709],[-0.040232795,0.0547088664],[-0.0407662796,0.0564894939],[-0.0414125802,0.0582390087],[-0.0420656521,0.0598217236],[-0.0426362598,0.0610991169],[-0.0430455251,0.061970168],[-0.0432611863,0.0623733292],[-0.0432431172,0.0622591887],[-0.0430202662,0.0616456233],[-0.0426253659,0.0605817944],[-0.0421132565,0.0591507565],[-0.0415622807,0.0574726378],[-0.0410586398,0.0556836398],[-0.0406875532,0.0539446961],[-0.0405155771,0.0523937914],[-0.040588337,0.0511504799],[-0.0409074899,0.0503085614],[-0.0414272976,0.0498776547],[-0.0420792198,0.0498581483],[-0.0427617185,0.0502010042],[-0.0433544117,0.0508161974],[-0.0437615103,0.051625796],[-0.0438952835,0.0525402835],[-0.0437051226,0.0534933369],[-0.0431605075,0.0544308646],[-0.0422749302,0.0553124302],[-0.0411036951,0.0561429808],[-0.0397055853,0.0569320139],[-0.0381868091,0.0577028805],[-0.0366571629,0.0584971629],[-0.035212257,0.0593693999],[-0.0339701317,0.0603529888],[-0.0330085765,0.0614835765],[-0.0323991912,0.0627834769],[-0.0321613061,0.0642184489],[-0.0323119887,0.0657844887],[-0.0328135134,0.0674117276],[-0.0336156361,0.0690245646],[-0.034641557,0.0705304855]],"B1211":[[-0.035821865,0.071839365],[-0.0370473444,0.0728355586],[-0.0382597229,0.0734518657],[-0.039401731,0.0736328025],[-0.040417054,0.0733334825],[-0.0412987169,0.0725694312],[-0.0420621077,0.0713810363],[-0.0427337695,0.0698523409],[-0.043371275,0.0680773464],[-0.0440229466,0.0661672323],[-0.0447376031,0.0642461745],[-0.0455416788,0.0624102503],[-0.0464382879,0.0607247165],[-0.0474060065,0.0592524351],[-0.048397797,0.0579906541],[-0.0493560527,0.0569235527],[-0.0502032578,0.0560346863],[-0.0508775259,0.0552743117],[-0.051322987,0.0546044155],[-0.0514892243,0.05398351],[-0.0513523665,0.0533802236],[-0.0508990581,0.0527683438],[-0.0501364846,0.0521418417],[-0.0490909571,0.0514995285],[-0.0478082102,0.050859996],[-0.0463716804,0.050278109],[-0.0448616775,0.0498081061],[-0.0433851272,0.04952727],[-0.0420673957,0.0495131099],[-0.0410029728,0.0498354728],[-0.0402739969,0.0505422112],[-0.0399157579,0.0516225436],[-0.039921428,0.0530435709],[-0.040232795,0.0547088664],[-0.0407662796,0.0564894939],[-0.0414125802,0.0582390087],[-0.0420656521,0.0598217236],[-0.0426362598,0.0610991169],[-0.0430455251,0.061970168],[-0.0432611863,0.0623733292],[-0.0432431172,0.0622591887],[-0.0430202662,0.0616456233],[-0.0426253659,0.0605817944],[-0.0421132565,0.0591507565],[-0.0415622807,0.0574726378],[-0.0410586398,0.0556836398],[-0.0406875532,0.0539446961],[-0.0405155771,0.0523937914],[-0.040588337,0.0511504799],[-0.0409074899,0.0503085614],[-0.0414272976,0.0498776547],[-0.0420792198,0.0498581483],[-0.0427617185,0.0502010042],[-0.0433544117,0.0508161974],[-0.0437615103,0.051625796],[-0.0438952835,0.0525402835],[-0.0437051226,0.0534933369],[-0.0431605075,0.0544308646],[-0.0422749302,0.0553124302],[-0.0411036951,0.0561429808],[-0.0397055853,0.0569320139],[-0.0381868091,0.0577028805],[-0.0366571629,0.0584971629],[-0.035212257,0.0593693999],[-0.0339701317,0.0603529888],[-0.0330085765,0.0614835765],[-0.0323991912,0.0627834769],[-0.0321613061,0.0642184489],[-0.0323119887,0.0657844887],[-0.0328135134,0.0674117276],[-0.0336156361,0.0690245646],[-0.034641557,0.0705304855]],"B1212":[[-0.0196781264,0.0319114597],[-0.0205089742,0.0323295297],[-0.02172625,0.03293625],[-0.0233535508,0.0337446619],[-0.0253325218,0.0347169663],[-0.0276053384,0.0358108939],[-0.0300899683,0.0369594127],[-0.0326695279,0.0380850834],[-0.0352462515,0.0391195848],[-0.0377460842,0.0400183064],[-0.0400668268,0.0407301601],[-0.0421446679,0.0412302235],[-0.0439474602,0.0415146824],[-0.0454305979,0.0415861535],[-0.0466050886,0.0414756442],[-0.0474691779,0.0412091779],[-0.0480460817,0.0408316372],[-0.0483642993,0.0403809659],[-0.0484500241,0.0398922464],[-0.0483408049,0.0394052493],[-0.0480486146,0.0389286146],[-0.0476129827,0.0384890938],[-0.0470312161,0.0380812161],[-0.0463046984,0.0376930318],[-0.0454318752,0.0373113197],[-0.0444007171,0.036904606],[-0.0432011176,0.036449451],[-0.0418186152,0.0359230597],[-0.0402703729,0.0353131507],[-0.0385704162,0.0346287496],[-0.0367355569,0.0338611124],[-0.0348208068,0.0330408068],[-0.0328721349,0.0322015794],[-0.0309372252,0.0313661141],[-0.0291095883,0.0305912549],[-0.0274296646,0.029894109],[-0.0259501977,0.0293068643],[-0.0247004636,0.0288254636],[-0.0236934001,0.0284389557],[-0.0229135382,0.0281207604],[-0.022341535,0.0278320905],[-0.0219369262,0.0275363706],[-0.0216576849,0.0271910182],[-0.0214675182,0.0267830737],[-0.021334747,0.0262986359],[-0.0212406796,0.0257467907],[-0.0211768347,0.0251618347],[-0.0211268267,0.0245584934],[-0.0211047236,0.0239886125],[-0.0210929594,0.0234785149],[-0.021117889,0.023077889],[-0.0211600866,0.0228067532],[-0.021222692,0.0226893587],[-0.0213056371,0.0227423038],[-0.0213979529,0.0229612863],[-0.0214840433,0.0233351544],[-0.0215662652,0.0238673764],[-0.0216260642,0.024524953],[-0.021665765,0.0252874317],[-0.0216611151,0.0261105595],[-0.0216137,0.0269775889],[-0.0215111765,0.0278311765],[-0.0213490151,0.0286451262],[-0.0211207305,0.0293818416],[-0.0208308727,0.0300153171],[-0.0204879411,0.0305251634],[-0.0201107584,0.0309068695],[-0.0197342967,0.0311659634],[-0.0193943742,0.0313265964],[-0.0191481463,0.0314270352],[-0.0190633394,0.0315161172],[-0.0192226152,0.0316615041]],"B1213":[[-0.0196781264,0.0319114597],[-0.0205089742,0.0323295297],[-0.02172625,0.03293625],[-0.0233535508,0.0337446619],[-0.0253325218,0.0347169663],[-0.0276053384,0.0358108939],[-0.0300899683,0.0369594127],[-0.0326695279,0.0380850834],[-0.0352462515,0.0391195848],[-0.0377460842,0.0400183064],[-0.0400668268,0.0407301601],[-0.0421446679,0.0412302235],[-0.0439474602,0.0415146824],[-0.0454305979,0.0415861535],[-0.0466050886,0.0414756442],[-0.0474691779,0.0412091779],[-0.0480460817,0.0408316372],[-0.0483642993,0.0403809659],[-0.0484500241,0.0398922464],[-0.0483408049,0.0394052493],[-0.0480486146,0.0389286146],[-0.0476129827,0.0384890938],[-0.0470312161,0.0380812161],[-0.0463046984,0.0376930318],[-0.0454318752,0.0373113197],[-0.0444007171,0.036904606],[-0.0432011176,0.036449451],[-0.0418186152,0.0359230597],[-0.0402703729,0.0353131507],[-0.0385704162,0.0346287496],[-0.0367355569,0.0338611124],[-0.0348208068,0.0330408068],[-0.0328721349,0.0322015794],[-0.0309372252,0.0313661141],[-0.0291095883,0.0305912549],[-0.0274296646,0.029894109],[-0.0259501977,0.0293068643],[-0.0247004636,0.0288254636],[-0.0236934001,0.0284389557],[-0.0229135382,0.0281207604],[-0.022341535,0.0278320905],[-0.0219369262,0.0275363706],[-0.0216576849,0.0271910182],[-0.0214675182,0.0267830737],[-0.021334747,0.0262986359],[-0.0212406796,0.0257467907],[-0.0211768347,0.0251618347],[-0.0211268267,0.0245584934],[-0.0211047236,0.0239886125],[-0.0210929594,0.0234785149],[-0.021117889,0.023077889],[-0.0211600866,0.0228067532],[-0.021222692,0.0226893587],[-0.0213056371,0.0227423038],[-0.0213979529,0.0229612863],[-0.0214840433,0.0233351544],[-0.0215662652,0.0238673764],[-0.0216260642,0.024524953],[-0.021665765,0.0252874317],[-0.0216611151,0.0261105595],[-0.0216137,0.0269775889],[-0.0215111765,0.0278311765],[-0.0213490151,0.0286451262],[-0.0211207305,0.0293818416],[-0.0208308727,0.0300153171],[-0.0204879411,0.0305251634],[-0.0201107584,0.0309068695],[-0.0197342967,0.0311659634],[-0.0193943742,0.0313265964],[-0.0191481463,0.0314270352],[-0.0190633394,0.0315161172],[-0.0192226152,0.0316615041]],"B1214":[[-0.0196781264,0.0319114597],[-0.0205089742,0.0323295297],[-0.02172625,0.03293625],[-0.0233535508,0.0337446619],[-0.0253325218,0.0347169663],[-0.0276053384,0.0358108939],[-0.0300899683,0.0369594127],[-0.0326695279,0.0380850834],[-0.0352462515,0.0391195848],[-0.0377460842,0.0400183064],[-0.0400668268,0.0407301601],[-0.0421446679,0.0412302235],[-0.0439474602,0.0415146824],[-0.0454305979,0.0415861535],[-0.0466050886,0.0414756442],[-0.0474691779,0.0412091779],[-0.0480460817,0.0408316372],[-0.0483642993,0.0403809659],[-0.0484500241,0.0398922464],[-0.0483408049,0.0394052493],[-0.0480486146,0.0389286146],[-0.0476129827,0.0384890938],[-0.0470312161,0.0380812161],[-0.0463046984,0.0376930318],[-0.0454318752,0.0373113197],[-0.0444007171,0.036904606],[-0.0432011176,0.036449451],[-0.0418186152,0.0359230597],[-0.0402703729,0.0353131507],[-0.0385704162,0.0346287496],[-0.0367355569,0.0338611124],[-0.0348208068,0.0330408068],[-0.0328721349,0.0322015794],[-0.0309372252,0.0313661141],[-0.0291095883,0.0305912549],[-0.0274296646,0.029894109],[-0.0259501977,0.0293068643],[-0.0247004636,0.0288254636],[-0.0236934001,0.0284389557],[-0.0229135382,0.0281207604],[-0.022341535,0.0278320905],[-0.0219369262,0.0275363706],[-0.0216576849,0.0271910182],[-0.0214675182,0.0267830737],[-0.021334747,0.0262986359],[-0.0212406796,0.0257467907],[-0.0211768347,0.0251618347],[-0.0211268267,0.0245584934],[-0.0211047236,0.0239886125],[-0.0210929594,0.0234785149],[-0.021117889,0.023077889],[-0.0211600866,0.0228067532],[-0.021222692,0.0226893587],[-0.0213056371,0.0227423038],[-0.0213979529,0.0229612863],[-0.0214840433,0.0233351544],[-0.0215662652,0.0238673764],[-0.0216260642,0.024524953],[-0.021665765,0.0252874317],[-0.0216611151,0.0261105595],[-0.0216137,0.0269775889],[-0.0215111765,0.0278311765],[-0.0213490151,0.0286451262],[-0.0211207305,0.0293818416],[-0.0208308727,0.0300153171],[-0.0204879411,0.0305251634],[-0.0201107584,0.0309068695],[-0.0197342967,0.0311659634],[-0.0193943742,0.0313265964],[-0.0191481463,0.0314270352],[-0.0190633394,0.0315161172],[-0.0192226152,0.0316615041]],"B1215":[[-0.035821865,0.071839365],[-0.0370473444,0.0728355586],[-0.0382597229,0.0734518657],[-0.039401731,0.0736328025],[-0.040417054,0.0733334825],[-0.0412987169,0.0725694312],[-0.0420621077,0.0713810363],[-0.0427337695,0.0698523409],[-0.043371275,0.0680773464],[-0.0440229466,0.0661672323],[-0.0447376031,0.0642461745],[-0.0455416788,0.0624102503],[-0.0464382879,0.0607247165],[-0.0474060065,0.0592524351],[-0.048397797,0.0579906541],[-0.0493560527,0.0569235527],[-0.0502032578,0.0560346863],[-0.0508775259,0.0552743117],[-0.051322987,0.0546044155],[-0.0514892243,0.05398351],[-0.0513523665,0.0533802236],[-0.0508990581,0.0527683438],[-0.0501364846,0.0521418417],[-0.0490909571,0.0514995285],[-0.0478082102,0.050859996],[-0.0463716804,0.050278109],[-0.0448616775,0.0498081061],[-0.0433851272,0.04952727],[-0.0420673957,0.0495131099],[-0.0410029728,0.0498354728],[-0.0402739969,0.0505422112],[-0.0399157579,0.0516225436],[-0.039921428,0.0530435709],[-0.040232795,0.0547088664],[-0.0407662796,0.0564894939],[-0.0414125802,0.0582390087],[-0.0420656521,0.0598217236],[-0.0426362598,0.0610991169],[-0.0430455251,0.061970168],[-0.0432611863,0.0623733292],[-0.0432431172,0.0622591887],[-0.0430202662,0.0616456233],[-0.0426253659,0.0605817944],[-0.0421132565,0.0591507565],[-0.0415622807,0.0574726378],[-0.0410586398,0.0556836398],[-0.0406875532,0.0539446961],[-0.0405155771,0.0523937914],[-0.040588337,0.0511504799],[-0.0409074899,0.0503085614],[-0.0414272976,0.0498776547],[-0.0420792198,0.0498581483],[-0.0427617185,0.0502010042],[-0.0433544117,0.0508161974],[-0.0437615103,0.051625796],[-0.0438952835,0.0525402835],[-0.0437051226,0.0534933369],[-0.0431605075,0.0544308646],[-0.0422749302,0.0553124302],[-0.0411036951,0.0561429808],[-0.0397055853,0.0569320139],[-0.0381868091,0.0577028805],[-0.0366571629,0.0584971629],[-0.035212257,0.0593693999],[-0.0339701317,0.0603529888],[-0.0330085765,0.0614835765],[-0.0323991912,0.0627834769],[-0.0321613061,0.0642184489],[-0.0323119887,0.0657844887],[-0.0328135134,0.0674117276],[-0.0336156361,0.0690245646],[-0.034641557,0.0705304855]],"B1216":[[-0.0196781264,0.0319114597],[-0.0205089742,0.0323295297],[-0.02172625,0.03293625],[-0.0233535508,0.0337446619],[-0.0253325218,0.0347169663],[-0.0276053384,0.0358108939],[-0.0300899683,0.0369594127],[-0.0326695279,0.0380850834],[-0.0352462515,0.0391195848],[-0.0377460842,0.0400183064],[-0.0400668268,0.0407301601],[-0.0421446679,0.0412302235],[-0.0439474602,0.0415146824],[-0.0454305979,0.0415861535],[-0.0466050886,0.0414756442],[-0.0474691779,0.0412091779],[-0.0480460817,0.0408316372],[-0.0483642993,0.0403809659],[-0.0484500241,0.0398922464],[-0.0483408049,0.0394052493],[-0.0480486146,0.0389286146],[-0.0476129827,0.0384890938],[-0.0470312161,0.0380812161],[-0.0463046984,0.0376930318],[-0.0454318752,0.0373113197],[-0.0444007171,0.036904606],[-0.0432011176,0.036449451],[-0.0418186152,0.0359230597],[-0.0402703729,0.0353131507],[-0.0385704162,0.0346287496],[-0.0367355569,0.0338611124],[-0.0348208068,0.0330408068],[-0.0328721349,0.0322015794],[-0.0309372252,0.0313661141],[-0.0291095883,0.0305912549],[-0.0274296646,0.029894109],[-0.0259501977,0.0293068643],[-0.0247004636,0.0288254636],[-0.0236934001,0.0284389557],[-0.0229135382,0.0281207604],[-0.022341535,0.0278320905],[-0.0219369262,0.0275363706],[-0.0216576849,0.0271910182],[-0.0214675182,0.0267830737],[-0.021334747,0.0262986359],[-0.0212406796,0.0257467907],[-0.0211768347,0.0251618347],[-0.0211268267,0.0245584934],[-0.0211047236,0.0239886125],[-0.0210929594,0.0234785149],[-0.021117889,0.023077889],[-0.0211600866,0.0228067532],[-0.021222692,0.0226893587],[-0.0213056371,0.0227423038],[-0.0213979529,0.0229612863],[-0.0214840433,0.0233351544],[-0.0215662652,0.0238673764],[-0.0216260642,0.024524953],[-0.021665765,0.0252874317],[-0.0216611151,0.0261105595],[-0.0216137,0.0269775889],[-0.0215111765,0.0278311765],[-0.0213490151,0.0286451262],[-0.0211207305,0.0293818416],[-0.0208308727,0.0300153171],[-0.0204879411,0.0305251634],[-0.0201107584,0.0309068695],[-0.0197342967,0.0311659634],[-0.0193943742,0.0313265964],[-0.0191481463,0.0314270352],[-0.0190633394,0.0315161172],[-0.0192226152,0.0316615041]],"B1217":[[-0.0196781264,0.0319114597],[-0.0205089742,0.0323295297],[-0.02172625,0.03293625],[-0.0233535508,0.0337446619],[-0.0253325218,0.0347169663],[-0.0276053384,0.0358108939],[-0.0300899683,0.0369594127],[-0.0326695279,0.0380850834],[-0.0352462515,0.0391195848],[-0.0377460842,0.0400183064],[-0.0400668268,0.0407301601],[-0.0421446679,0.0412302235],[-0.0439474602,0.0415146824],[-0.0454305979,0.0415861535],[-0.0466050886,0.0414756442],[-0.0474691779,0.0412091779],[-0.0480460817,0.0408316372],[-0.0483642993,0.0403809659],[-0.0484500241,0.0398922464],[-0.0483408049,0.0394052493],[-0.0480486146,0.0389286146],[-0.0476129827,0.0384890938],[-0.0470312161,0.0380812161],[-0.0463046984,0.0376930318],[-0.0454318752,0.0373113197],[-0.0444007171,0.036904606],[-0.0432011176,0.036449451],[-0.0418186152,0.0359230597],[-0.0402703729,0.0353131507],[-0.0385704162,0.0346287496],[-0.0367355569,0.0338611124],[-0.0348208068,0.0330408068],[-0.0328721349,0.0322015794],[-0.0309372252,0.0313661141],[-0.0291095883,0.0305912549],[-0.0274296646,0.029894109],[-0.0259501977,0.0293068643],[-0.0247004636,0.0288254636],[-0.0236934001,0.0284389557],[-0.0229135382,0.0281207604],[-0.022341535,0.0278320905],[-0.0219369262,0.0275363706],[-0.0216576849,0.0271910182],[-0.0214675182,0.0267830737],[-0.021334747,0.0262986359],[-0.0212406796,0.0257467907],[-0.0211768347,0.0251618347],[-0.0211268267,0.0245584934],[-0.0211047236,0.0239886125],[-0.0210929594,0.0234785149],[-0.021117889,0.023077889],[-0.0211600866,0.0228067532],[-0.021222692,0.0226893587],[-0.0213056371,0.0227423038],[-0.0213979529,0.0229612863],[-0.0214840433,0.0233351544],[-0.0215662652,0.0238673764],[-0.0216260642,0.024524953],[-0.021665765,0.0252874317],[-0.0216611151,0.0261105595],[-0.0216137,0.0269775889],[-0.0215111765,0.0278311765],[-0.0213490151,0.0286451262],[-0.0211207305,0.0293818416],[-0.0208308727,0.0300153171],[-0.0204879411,0.0305251634],[-0.0201107584,0.0309068695],[-0.0197342967,0.0311659634],[-0.0193943742,0.0313265964],[-0.0191481463,0.0314270352],[-0.0190633394,0.0315161172],[-0.0192226152,0.0316615041]],"B1218":[[-0.0196781264,0.0319114597],[-0.0205089742,0.0323295297],[-0.02172625,0.03293625],[-0.0233535508,0.0337446619],[-0.0253325218,0.0347169663],[-0.0276053384,0.0358108939],[-0.0300899683,0.0369594127],[-0.0326695279,0.0380850834],[-0.0352462515,0.0391195848],[-0.0377460842,0.0400183064],[-0.0400668268,0.0407301601],[-0.0421446679,0.0412302235],[-0.0439474602,0.0415146824],[-0.0454305979,0.0415861535],[-0.0466050886,0.0414756442],[-0.0474691779,0.0412091779],[-0.0480460817,0.0408316372],[-0.0483642993,0.0403809659],[-0.0484500241,0.0398922464],[-0.0483408049,0.0394052493],[-0.0480486146,0.0389286146],[-0.0476129827,0.0384890938],[-0.0470312161,0.0380812161],[-0.0463046984,0.0376930318],[-0.0454318752,0.0373113197],[-0.0444007171,0.036904606],[-0.0432011176,0.036449451],[-0.0418186152,0.0359230597],[-0.0402703729,0.0353131507],[-0.0385704162,0.0346287496],[-0.0367355569,0.0338611124],[-0.0348208068,0.0330408068],[-0.0328721349,0.0322015794],[-0.0309372252,0.0313661141],[-0.0291095883,0.0305912549],[-0.0274296646,0.029894109],[-0.0259501977,0.0293068643],[-0.0247004636,0.0288254636],[-0.0236934001,0.0284389557],[-0.0229135382,0.0281207604],[-0.022341535,0.0278320905],[-0.0219369262,0.0275363706],[-0.0216576849,0.0271910182],[-0.0214675182,0.0267830737],[-0.021334747,0.0262986359],[-0.0212406796,0.0257467907],[-0.0211768347,0.0251618347],[-0.0211268267,0.0245584934],[-0.0211047236,0.0239886125],[-0.0210929594,0.0234785149],[-0.021117889,0.023077889],[-0.0211600866,0.0228067532],[-0.021222692,0.0226893587],[-0.0213056371,0.0227423038],[-0.0213979529,0.0229612863],[-0.0214840433,0.0233351544],[-0.0215662652,0.0238673764],[-0.0216260642,0.024524953],[-0.021665765,0.0252874317],[-0.0216611151,0.0261105595],[-0.0216137,0.0269775889],[-0.0215111765,0.0278311765],[-0.0213490151,0.0286451262],[-0.0211207305,0.0293818416],[-0.0208308727,0.0300153171],[-0.0204879411,0.0305251634],[-0.0201107584,0.0309068695],[-0.0197342967,0.0311659634],[-0.0193943742,0.0313265964],[-0.0191481463,0.0314270352],[-0.0190633394,0.0315161172],[-0.0192226152,0.0316615041]],"B1219":[[-0.0196781264,0.0319114597],[-0.0205089742,0.0323295297],[-0.02172625,0.03293625],[-0.0233535508,0.0337446619],[-0.0253325218,0.0347169663],[-0.0276053384,0.0358108939],[-0.0300899683,0.0369594127],[-0.0326695279,0.0380850834],[-0.0352462515,0.0391195848],[-0.0377460842,0.0400183064],[-0.0400668268,0.0407301601],[-0.0421446679,0.0412302235],[-0.0439474602,0.0415146824],[-0.0454305979,0.0415861535],[-0.0466050886,0.0414756442],[-0.0474691779,0.0412091779],[-0.0480460817,0.0408316372],[-0.0483642993,0.0403809659],[-0.0484500241,0.0398922464],[-0.0483408049,0.0394052493],[-0.0480486146,0.0389286146],[-0.0476129827,0.0384890938],[-0.0470312161,0.0380812161],[-0.0463046984,0.0376930318],[-0.0454318752,0.0373113197],[-0.0444007171,0.036904606],[-0.0432011176,0.036449451],[-0.0418186152,0.0359230597],[-0.0402703729,0.0353131507],[-0.0385704162,0.0346287496],[-0.0367355569,0.0338611124],[-0.0348208068,0.0330408068],[-0.0328721349,0.0322015794],[-0.0309372252,0.0313661141],[-0.0291095883,0.0305912549],[-0.0274296646,0.029894109],[-0.0259501977,0.0293068643],[-0.0247004636,0.0288254636],[-0.0236934001,0.0284389557],[-0.0229135382,0.0281207604],[-0.022341535,0.0278320905],[-0.0219369262,0.0275363706],[-0.0216576849,0.0271910182],[-0.0214675182,0.0267830737],[-0.021334747,0.0262986359],[-0.0212406796,0.0257467907],[-0.0211768347,0.0251618347],[-0.0211268267,0.0245584934],[-0.0211047236,0.0239886125],[-0.0210929594,0.0234785149],[-0.021117889,0.023077889],[-0.0211600866,0.0228067532],[-0.021222692,0.0226893587],[-0.0213056371,0.0227423038],[-0.0213979529,0.0229612863],[-0.0214840433,0.0233351544],[-0.0215662652,0.0238673764],[-0.0216260642,0.024524953],[-0.021665765,0.0252874317],[-0.0216611151,0.0261105595],[-0.0216137,0.0269775889],[-0.0215111765,0.0278311765],[-0.0213490151,0.0286451262],[-0.0211207305,0.0293818416],[-0.0208308727,0.0300153171],[-0.0204879411,0.0305251634],[-0.0201107584,0.0309068695],[-0.0197342967,0.0311659634],[-0.0193943742,0.0313265964],[-0.0191481463,0.0314270352],[-0.0190633394,0.0315161172],[-0.0192226152,0.0316615041]],"B1220":[[-0.0196781264,0.0319114597],[-0.0205089742,0.0323295297],[-0.02172625,0.03293625],[-0.0233535508,0.0337446619],[-0.0253325218,0.0347169663],[-0.0276053384,0.0358108939],[-0.0300899683,0.0369594127],[-0.0326695279,0.0380850834],[-0.0352462515,0.0391195848],[-0.0377460842,0.0400183064],[-0.0400668268,0.0407301601],[-0.0421446679,0.0412302235],[-0.0439474602,0.0415146824],[-0.0454305979,0.0415861535],[-0.0466050886,0.0414756442],[-0.0474691779,0.0412091779],[-0.0480460817,0.0408316372],[-0.0483642993,0.0403809659],[-0.0484500241,0.0398922464],[-0.0483408049,0.0394052493],[-0.0480486146,0.0389286146],[-0.0476129827,0.0384890938],[-0.0470312161,0.0380812161],[-0.0463046984,0.0376930318],[-0.0454318752,0.0373113197],[-0.0444007171,0.036904606],[-0.0432011176,0.036449451],[-0.0418186152,0.0359230597],[-0.0402703729,0.0353131507],[-0.0385704162,0.0346287496],[-0.0367355569,0.0338611124],[-0.0348208068,0.0330408068],[-0.0328721349,0.0322015794],[-0.0309372252,0.0313661141],[-0.0291095883,0.0305912549],[-0.0274296646,0.029894109],[-0.0259501977,0.0293068643],[-0.0247004636,0.0288254636],[-0.0236934001,0.0284389557],[-0.0229135382,0.0281207604],[-0.022341535,0.0278320905],[-0.0219369262,0.0275363706],[-0.0216576849,0.0271910182],[-0.0214675182,0.0267830737],[-0.021334747,0.0262986359],[-0.0212406796,0.0257467907],[-0.0211768347,0.0251618347],[-0.0211268267,0.0245584934],[-0.0211047236,0.0239886125],[-0.0210929594,0.0234785149],[-0.021117889,0.023077889],[-0.0211600866,0.0228067532],[-0.021222692,0.0226893587],[-0.0213056371,0.0227423038],[-0.0213979529,0.0229612863],[-0.0214840433,0.0233351544],[-0.0215662652,0.0238673764],[-0.0216260642,0.024524953],[-0.021665765,0.0252874317],[-0.0216611151,0.0261105595],[-0.0216137,0.0269775889],[-0.0215111765,0.0278311765],[-0.0213490151,0.0286451262],[-0.0211207305,0.0293818416],[-0.0208308727,0.0300153171],[-0.0204879411,0.0305251634],[-0.0201107584,0.0309068695],[-0.0197342967,0.0311659634],[-0.0193943742,0.0313265964],[-0.0191481463,0.0314270352],[-0.0190633394,0.0315161172],[-0.0192226152,0.0316615041]],"B1221":[[-0.035821865,0.071839365],[-0.0370473444,0.0728355586],[-0.0382597229,0.0734518657],[-0.039401731,0.0736328025],[-0.040417054,0.0733334825],[-0.0412987169,0.0725694312],[-0.0420621077,0.0713810363],[-0.0427337695,0.0698523409],[-0.043371275,0.0680773464],[-0.0440229466,0.0661672323],[-0.0447376031,0.0642461745],[-0.0455416788,0.0624102503],[-0.0464382879,0.0607247165],[-0.0474060065,0.0592524351],[-0.048397797,0.0579906541],[-0.0493560527,0.0569235527],[-0.0502032578,0.0560346863],[-0.0508775259,0.0552743117],[-0.051322987,0.0546044155],[-0.0514892243,0.05398351],[-0.0513523665,0.0533802236],[-0.0508990581,0.0527683438],[-0.0501364846,0.0521418417],[-0.0490909571,0.0514995285],[-0.0478082102,0.050859996],[-0.0463716804,0.050278109],[-0.0448616775,0.0498081061],[-0.0433851272,0.04952727],[-0.0420673957,0.0495131099],[-0.0410029728,0.0498354728],[-0.0402739969,0.0505422112],[-0.0399157579,0.0516225436],[-0.039921428,0.0530435709],[-0.040232795,0.0547088664],[-0.0407662796,0.0564894939],[-0.0414125802,0.0582390087],[-0.0420656521,0.0598217236],[-0.0426362598,0.0610991169],[-0.0430455251,0.061970168],[-0.0432611863,0.0623733292],[-0.0432431172,0.0622591887],[-0.0430202662,0.0616456233],[-0.0426253659,0.0605817944],[-0.0421132565,0.0591507565],[-0.0415622807,0.0574726378],[-0.0410586398,0.0556836398],[-0.0406875532,0.0539446961],[-0.0405155771,0.0523937914],[-0.040588337,0.0511504799],[-0.0409074899,0.0503085614],[-0.0414272976,0.0498776547],[-0.0420792198,0.0498581483],[-0.0427617185,0.0502010042],[-0.0433544117,0.0508161974],[-0.0437615103,0.051625796],[-0.0438952835,0.0525402835],[-0.0437051226,0.0534933369],[-0.0431605075,0.0544308646],[-0.0422749302,0.0553124302],[-0.0411036951,0.0561429808],[-0.0397055853,0.0569320139],[-0.0381868091,0.0577028805],[-0.0366571629,0.0584971629],[-0.035212257,0.0593693999],[-0.0339701317,0.0603529888],[-0.0330085765,0.0614835765],[-0.0323991912,0.0627834769],[-0.0321613061,0.0642184489],[-0.0323119887,0.0657844887],[-0.0328135134,0.0674117276],[-0.0336156361,0.0690245646],[-0.034641557,0.0705304855]],"B1222":[[-0.035821865,0.071839365],[-0.0370473444,0.0728355586],[-0.0382597229,0.0734518657],[-0.039401731,0.0736328025],[-0.040417054,0.0733334825],[-0.0412987169,0.0725694312],[-0.0420621077,0.0713810363],[-0.0427337695,0.0698523409],[-0.043371275,0.0680773464],[-0.0440229466,0.0661672323],[-0.0447376031,0.0642461745],[-0.0455416788,0.0624102503],[-0.0464382879,0.0607247165],[-0.0474060065,0.0592524351],[-0.048397797,0.0579906541],[-0.0493560527,0.0569235527],[-0.0502032578,0.0560346863],[-0.0508775259,0.0552743117],[-0.051322987,0.0546044155],[-0.0514892243,0.05398351],[-0.0513523665,0.0533802236],[-0.0508990581,0.0527683438],[-0.0501364846,0.0521418417],[-0.0490909571,0.0514995285],[-0.0478082102,0.050859996],[-0.0463716804,0.050278109],[-0.0448616775,0.0498081061],[-0.0433851272,0.04952727],[-0.0420673957,0.0495131099],[-0.0410029728,0.0498354728],[-0.0402739969,0.0505422112],[-0.0399157579,0.0516225436],[-0.039921428,0.0530435709],[-0.040232795,0.0547088664],[-0.0407662796,0.0564894939],[-0.0414125802,0.0582390087],[-0.0420656521,0.0598217236],[-0.0426362598,0.0610991169],[-0.0430455251,0.061970168],[-0.0432611863,0.0623733292],[-0.0432431172,0.0622591887],[-0.0430202662,0.0616456233],[-0.0426253659,0.0605817944],[-0.0421132565,0.0591507565],[-0.0415622807,0.0574726378],[-0.0410586398,0.0556836398],[-0.0406875532,0.0539446961],[-0.0405155771,0.0523937914],[-0.040588337,0.0511504799],[-0.0409074899,0.0503085614],[-0.0414272976,0.0498776547],[-0.0420792198,0.0498581483],[-0.0427617185,0.0502010042],[-0.0433544117,0.0508161974],[-0.0437615103,0.051625796],[-0.0438952835,0.0525402835],[-0.0437051226,0.0534933369],[-0.0431605075,0.0544308646],[-0.0422749302,0.0553124302],[-0.0411036951,0.0561429808],[-0.0397055853,0.0569320139],[-0.0381868091,0.0577028805],[-0.0366571629,0.0584971629],[-0.035212257,0.0593693999],[-0.0339701317,0.0603529888],[-0.0330085765,0.0614835765],[-0.0323991912,0.0627834769],[-0.0321613061,0.0642184489],[-0.0323119887,0.0657844887],[-0.0328135134,0.0674117276],[-0.0336156361,0.0690245646],[-0.034641557,0.0705304855]],"B1223":[[-0.035821865,0.071839365],[-0.0370473444,0.0728355586],[-0.0382597229,0.0734518657],[-0.039401731,0.0736328025],[-0.040417054,0.0733334825],[-0.0412987169,0.0725694312],[-0.0420621077,0.0713810363],[-0.0427337695,0.0698523409],[-0.043371275,0.0680773464],[-0.0440229466,0.0661672323],[-0.0447376031,0.0642461745],[-0.0455416788,0.0624102503],[-0.0464382879,0.0607247165],[-0.0474060065,0.0592524351],[-0.048397797,0.0579906541],[-0.0493560527,0.0569235527],[-0.0502032578,0.0560346863],[-0.0508775259,0.0552743117],[-0.051322987,0.0546044155],[-0.0514892243,0.05398351],[-0.0513523665,0.0533802236],[-0.0508990581,0.0527683438],[-0.0501364846,0.0521418417],[-0.0490909571,0.0514995285],[-0.0478082102,0.050859996],[-0.0463716804,0.050278109],[-0.0448616775,0.0498081061],[-0.0433851272,0.04952727],[-0.0420673957,0.0495131099],[-0.0410029728,0.0498354728],[-0.0402739969,0.0505422112],[-0.0399157579,0.0516225436],[-0.039921428,0.0530435709],[-0.040232795,0.0547088664],[-0.0407662796,0.0564894939],[-0.0414125802,0.0582390087],[-0.0420656521,0.0598217236],[-0.0426362598,0.0610991169],[-0.0430455251,0.061970168],[-0.0432611863,0.0623733292],[-0.0432431172,0.0622591887],[-0.0430202662,0.0616456233],[-0.0426253659,0.0605817944],[-0.0421132565,0.0591507565],[-0.0415622807,0.0574726378],[-0.0410586398,0.0556836398],[-0.0406875532,0.0539446961],[-0.0405155771,0.0523937914],[-0.040588337,0.0511504799],[-0.0409074899,0.0503085614],[-0.0414272976,0.0498776547],[-0.0420792198,0.0498581483],[-0.0427617185,0.0502010042],[-0.0433544117,0.0508161974],[-0.0437615103,0.051625796],[-0.0438952835,0.0525402835],[-0.0437051226,0.0534933369],[-0.0431605075,0.0544308646],[-0.0422749302,0.0553124302],[-0.0411036951,0.0561429808],[-0.0397055853,0.0569320139],[-0.0381868091,0.0577028805],[-0.0366571629,0.0584971629],[-0.035212257,0.0593693999],[-0.0339701317,0.0603529888],[-0.0330085765,0.0614835765],[-0.0323991912,0.0627834769],[-0.0321613061,0.0642184489],[-0.0323119887,0.0657844887],[-0.0328135134,0.0674117276],[-0.0336156361,0.0690245646],[-0.034641557,0.0705304855]],"B1224":[[-0.035821865,0.071839365],[-0.0370473444,0.0728355586],[-0.0382597229,0.0734518657],[-0.039401731,0.0736328025],[-0.040417054,0.0733334825],[-0.0412987169,0.0725694312],[-0.0420621077,0.0713810363],[-0.0427337695,0.0698523409],[-0.043371275,0.0680773464],[-0.0440229466,0.0661672323],[-0.0447376031,0.0642461745],[-0.0455416788,0.0624102503],[-0.0464382879,0.0607247165],[-0.0474060065,0.0592524351],[-0.048397797,0.0579906541],[-0.0493560527,0.0569235527],[-0.0502032578,0.0560346863],[-0.0508775259,0.0552743117],[-0.051322987,0.0546044155],[-0.0514892243,0.05398351],[-0.0513523665,0.0533802236],[-0.0508990581,0.0527683438],[-0.0501364846,0.0521418417],[-0.0490909571,0.0514995285],[-0.0478082102,0.050859996],[-0.0463716804,0.050278109],[-0.0448616775,0.0498081061],[-0.0433851272,0.04952727],[-0.0420673957,0.0495131099],[-0.0410029728,0.0498354728],[-0.0402739969,0.0505422112],[-0.0399157579,0.0516225436],[-0.039921428,0.0530435709],[-0.040232795,0.0547088664],[-0.0407662796,0.0564894939],[-0.0414125802,0.0582390087],[-0.0420656521,0.0598217236],[-0.0426362598,0.0610991169],[-0.0430455251,0.061970168],[-0.0432611863,0.0623733292],[-0.0432431172,0.0622591887],[-0.0430202662,0.0616456233],[-0.0426253659,0.0605817944],[-0.0421132565,0.0591507565],[-0.0415622807,0.0574726378],[-0.0410586398,0.0556836398],[-0.0406875532,0.0539446961],[-0.0405155771,0.0523937914],[-0.040588337,0.0511504799],[-0.0409074899,0.0503085614],[-0.0414272976,0.0498776547],[-0.0420792198,0.0498581483],[-0.0427617185,0.0502010042],[-0.0433544117,0.0508161974],[-0.0437615103,0.051625796],[-0.0438952835,0.0525402835],[-0.0437051226,0.0534933369],[-0.0431605075,0.0544308646],[-0.0422749302,0.0553124302],[-0.0411036951,0.0561429808],[-0.0397055853,0.0569320139],[-0.0381868091,0.0577028805],[-0.0366571629,0.0584971629],[-0.035212257,0.0593693999],[-0.0339701317,0.0603529888],[-0.0330085765,0.0614835765],[-0.0323991912,0.0627834769],[-0.0321613061,0.0642184489],[-0.0323119887,0.0657844887],[-0.0328135134,0.0674117276],[-0.0336156361,0.0690245646],[-0.034641557,0.0705304855]],"B1225":[[-0.035821865,0.071839365],[-0.0370473444,0.0728355586],[-0.0382597229,0.0734518657],[-0.039401731,0.0736328025],[-0.040417054,0.0733334825],[-0.0412987169,0.0725694312],[-0.0420621077,0.0713810363],[-0.0427337695,0.0698523409],[-0.043371275,0.0680773464],[-0.0440229466,0.0661672323],[-0.0447376031,0.0642461745],[-0.0455416788,0.0624102503],[-0.0464382879,0.0607247165],[-0.0474060065,0.0592524351],[-0.048397797,0.0579906541],[-0.0493560527,0.0569235527],[-0.0502032578,0.0560346863],[-0.0508775259,0.0552743117],[-0.051322987,0.0546044155],[-0.0514892243,0.05398351],[-0.0513523665,0.0533802236],[-0.0508990581,0.0527683438],[-0.0501364846,0.0521418417],[-0.0490909571,0.0514995285],[-0.0478082102,0.050859996],[-0.0463716804,0.050278109],[-0.0448616775,0.0498081061],[-0.0433851272,0.04952727],[-0.0420673957,0.0495131099],[-0.0410029728,0.0498354728],[-0.0402739969,0.0505422112],[-0.0399157579,0.0516225436],[-0.039921428,0.0530435709],[-0.040232795,0.0547088664],[-0.0407662796,0.0564894939],[-0.0414125802,0.0582390087],[-0.0420656521,0.0598217236],[-0.0426362598,0.0610991169],[-0.0430455251,0.061970168],[-0.0432611863,0.0623733292],[-0.0432431172,0.0622591887],[-0.0430202662,0.0616456233],[-0.0426253659,0.0605817944],[-0.0421132565,0.0591507565],[-0.0415622807,0.0574726378],[-0.0410586398,0.0556836398],[-0.0406875532,0.0539446961],[-0.0405155771,0.0523937914],[-0.040588337,0.0511504799],[-0.0409074899,0.0503085614],[-0.0414272976,0.0498776547],[-0.0420792198,0.0498581483],[-0.0427617185,0.0502010042],[-0.0433544117,0.0508161974],[-0.0437615103,0.051625796],[-0.0438952835,0.0525402835],[-0.0437051226,0.0534933369],[-0.0431605075,0.0544308646],[-0.0422749302,0.0553124302],[-0.0411036951,0.0561429808],[-0.0397055853,0.0569320139],[-0.0381868091,0.0577028805],[-0.0366571629,0.0584971629],[-0.035212257,0.0593693999],[-0.0339701317,0.0603529888],[-0.0330085765,0.0614835765],[-0.0323991912,0.0627834769],[-0.0321613061,0.0642184489],[-0.0323119887,0.0657844887],[-0.0328135134,0.0674117276],[-0.0336156361,0.0690245646],[-0.034641557,0.0705304855]],"B1226":[[-0.035821865,0.071839365],[-0.0370473444,0.0728355586],[-0.0382597229,0.0734518657],[-0.039401731,0.0736328025],[-0.040417054,0.0733334825],[-0.0412987169,0.0725694312],[-0.0420621077,0.0713810363],[-0.0427337695,0.0698523409],[-0.043371275,0.0680773464],[-0.0440229466,0.0661672323],[-0.0447376031,0.0642461745],[-0.0455416788,0.0624102503],[-0.0464382879,0.0607247165],[-0.0474060065,0.0592524351],[-0.048397797,0.0579906541],[-0.0493560527,0.0569235527],[-0.0502032578,0.0560346863],[-0.0508775259,0.0552743117],[-0.051322987,0.0546044155],[-0.0514892243,0.05398351],[-0.0513523665,0.0533802236],[-0.0508990581,0.0527683438],[-0.0501364846,0.0521418417],[-0.0490909571,0.0514995285],[-0.0478082102,0.050859996],[-0.0463716804,0.050278109],[-0.0448616775,0.0498081061],[-0.0433851272,0.04952727],[-0.0420673957,0.0495131099],[-0.0410029728,0.0498354728],[-0.0402739969,0.0505422112],[-0.0399157579,0.0516225436],[-0.039921428,0.0530435709],[-0.040232795,0.0547088664],[-0.0407662796,0.0564894939],[-0.0414125802,0.0582390087],[-0.0420656521,0.0598217236],[-0.0426362598,0.0610991169],[-0.0430455251,0.061970168],[-0.0432611863,0.0623733292],[-0.0432431172,0.0622591887],[-0.0430202662,0.0616456233],[-0.0426253659,0.0605817944],[-0.0421132565,0.0591507565],[-0.0415622807,0.0574726378],[-0.0410586398,0.0556836398],[-0.0406875532,0.0539446961],[-0.0405155771,0.0523937914],[-0.040588337,0.0511504799],[-0.0409074899,0.0503085614],[-0.0414272976,0.0498776547],[-0.0420792198,0.0498581483],[-0.0427617185,0.0502010042],[-0.0433544117,0.0508161974],[-0.0437615103,0.051625796],[-0.0438952835,0.0525402835],[-0.0437051226,0.0534933369],[-0.0431605075,0.0544308646],[-0.0422749302,0.0553124302],[-0.0411036951,0.0561429808],[-0.0397055853,0.0569320139],[-0.0381868091,0.0577028805],[-0.0366571629,0.0584971629],[-0.035212257,0.0593693999],[-0.0339701317,0.0603529888],[-0.0330085765,0.0614835765],[-0.0323991912,0.0627834769],[-0.0321613061,0.0642184489],[-0.0323119887,0.0657844887],[-0.0328135134,0.0674117276],[-0.0336156361,0.0690245646],[-0.034641557,0.0705304855]],"B1227":[[-0.0196781264,0.0319114597],[-0.0205089742,0.0323295297],[-0.02172625,0.03293625],[-0.0233535508,0.0337446619],[-0.0253325218,0.0347169663],[-0.0276053384,0.0358108939],[-0.0300899683,0.0369594127],[-0.0326695279,0.0380850834],[-0.0352462515,0.0391195848],[-0.0377460842,0.0400183064],[-0.0400668268,0.0407301601],[-0.0421446679,0.0412302235],[-0.0439474602,0.0415146824],[-0.0454305979,0.0415861535],[-0.0466050886,0.0414756442],[-0.0474691779,0.0412091779],[-0.0480460817,0.0408316372],[-0.0483642993,0.0403809659],[-0.0484500241,0.0398922464],[-0.0483408049,0.0394052493],[-0.0480486146,0.0389286146],[-0.0476129827,0.0384890938],[-0.0470312161,0.0380812161],[-0.0463046984,0.0376930318],[-0.0454318752,0.0373113197],[-0.0444007171,0.036904606],[-0.0432011176,0.036449451],[-0.0418186152,0.0359230597],[-0.0402703729,0.0353131507],[-0.0385704162,0.0346287496],[-0.0367355569,0.0338611124],[-0.0348208068,0.0330408068],[-0.0328721349,0.0322015794],[-0.0309372252,0.0313661141],[-0.0291095883,0.0305912549],[-0.0274296646,0.029894109],[-0.0259501977,0.0293068643],[-0.0247004636,0.0288254636],[-0.0236934001,0.0284389557],[-0.0229135382,0.0281207604],[-0.022341535,0.0278320905],[-0.0219369262,0.0275363706],[-0.0216576849,0.0271910182],[-0.0214675182,0.0267830737],[-0.021334747,0.0262986359],[-0.0212406796,0.0257467907],[-0.0211768347,0.0251618347],[-0.0211268267,0.0245584934],[-0.0211047236,0.0239886125],[-0.0210929594,0.0234785149],[-0.021117889,0.023077889],[-0.0211600866,0.0228067532],[-0.021222692,0.0226893587],[-0.0213056371,0.0227423038],[-0.0213979529,0.0229612863],[-0.0214840433,0.0233351544],[-0.0215662652,0.0238673764],[-0.0216260642,0.024524953],[-0.021665765,0.0252874317],[-0.0216611151,0.0261105595],[-0.0216137,0.0269775889],[-0.0215111765,0.0278311765],[-0.0213490151,0.0286451262],[-0.0211207305,0.0293818416],[-0.0208308727,0.0300153171],[-0.0204879411,0.0305251634],[-0.0201107584,0.0309068695],[-0.0197342967,0.0311659634],[-0.0193943742,0.0313265964],[-0.0191481463,0.0314270352],[-0.0190633394,0.0315161172],[-0.0192226152,0.0316615041]],"B1228":[[-0.0196781264,0.0319114597],[-0.0205089742,0.0323295297],[-0.02172625,0.03293625],[-0.0233535508,0.0337446619],[-0.0253325218,0.0347169663],[-0.0276053384,0.0358108939],[-0.0300899683,0.0369594127],[-0.0326695279,0.0380850834],[-0.0352462515,0.0391195848],[-0.0377460842,0.0400183064],[-0.0400668268,0.0407301601],[-0.0421446679,0.0412302235],[-0.0439474602,0.0415146824],[-0.0454305979,0.0415861535],[-0.0466050886,0.0414756442],[-0.0474691779,0.0412091779],[-0.0480460817,0.0408316372],[-0.0483642993,0.0403809659],[-0.0484500241,0.0398922464],[-0.0483408049,0.0394052493],[-0.0480486146,0.0389286146],[-0.0476129827,0.0384890938],[-0.0470312161,0.0380812161],[-0.0463046984,0.0376930318],[-0.0454318752,0.0373113197],[-0.0444007171,0.036904606],[-0.0432011176,0.036449451],[-0.0418186152,0.0359230597],[-0.0402703729,0.0353131507],[-0.0385704162,0.0346287496],[-0.0367355569,0.0338611124],[-0.0348208068,0.0330408068],[-0.0328721349,0.0322015794],[-0.0309372252,0.0313661141],[-0.0291095883,0.0305912549],[-0.0274296646,0.029894109],[-0.0259501977,0.0293068643],[-0.0247004636,0.0288254636],[-0.0236934001,0.0284389557],[-0.0229135382,0.0281207604],[-0.022341535,0.0278320905],[-0.0219369262,0.0275363706],[-0.0216576849,0.0271910182],[-0.0214675182,0.0267830737],[-0.021334747,0.0262986359],[-0.0212406796,0.0257467907],[-0.0211768347,0.0251618347],[-0.0211268267,0.0245584934],[-0.0211047236,0.0239886125],[-0.0210929594,0.0234785149],[-0.021117889,0.023077889],[-0.0211600866,0.0228067532],[-0.021222692,0.0226893587],[-0.0213056371,0.0227423038],[-0.0213979529,0.0229612863],[-0.0214840433,0.0233351544],[-0.0215662652,0.0238673764],[-0.0216260642,0.024524953],[-0.021665765,0.0252874317],[-0.0216611151,0.0261105595],[-0.0216137,0.0269775889],[-0.0215111765,0.0278311765],[-0.0213490151,0.0286451262],[-0.0211207305,0.0293818416],[-0.0208308727,0.0300153171],[-0.0204879411,0.0305251634],[-0.0201107584,0.0309068695],[-0.0197342967,0.0311659634],[-0.0193943742,0.0313265964],[-0.0191481463,0.0314270352],[-0.0190633394,0.0315161172],[-0.0192226152,0.0316615041]],"B1229":[[-0.0196781264,0.0319114597],[-0.0205089742,0.0323295297],[-0.02172625,0.03293625],[-0.0233535508,0.0337446619],[-0.0253325218,0.0347169663],[-0.0276053384,0.0358108939],[-0.0300899683,0.0369594127],[-0.0326695279,0.0380850834],[-0.0352462515,0.0391195848],[-0.0377460842,0.0400183064],[-0.0400668268,0.0407301601],[-0.0421446679,0.0412302235],[-0.0439474602,0.0415146824],[-0.0454305979,0.0415861535],[-0.0466050886,0.0414756442],[-0.0474691779,0.0412091779],[-0.0480460817,0.0408316372],[-0.0483642993,0.0403809659],[-0.0484500241,0.0398922464],[-0.0483408049,0.0394052493],[-0.0480486146,0.0389286146],[-0.0476129827,0.0384890938],[-0.0470312161,0.0380812161],[-0.0463046984,0.0376930318],[-0.0454318752,0.0373113197],[-0.0444007171,0.036904606],[-0.0432011176,0.036449451],[-0.0418186152,0.0359230597],[-0.0402703729,0.0353131507],[-0.0385704162,0.0346287496],[-0.0367355569,0.0338611124],[-0.0348208068,0.0330408068],[-0.0328721349,0.0322015794],[-0.0309372252,0.0313661141],[-0.0291095883,0.0305912549],[-0.0274296646,0.029894109],[-0.0259501977,0.0293068643],[-0.0247004636,0.0288254636],[-0.0236934001,0.0284389557],[-0.0229135382,0.0281207604],[-0.022341535,0.0278320905],[-0.0219369262,0.0275363706],[-0.0216576849,0.0271910182],[-0.0214675182,0.0267830737],[-0.021334747,0.0262986359],[-0.0212406796,0.0257467907],[-0.0211768347,0.0251618347],[-0.0211268267,0.0245584934],[-0.0211047236,0.0239886125],[-0.0210929594,0.0234785149],[-0.021117889,0.023077889],[-0.0211600866,0.0228067532],[-0.021222692,0.0226893587],[-0.0213056371,0.0227423038],[-0.0213979529,0.0229612863],[-0.0214840433,0.0233351544],[-0.0215662652,0.0238673764],[-0.0216260642,0.024524953],[-0.021665765,0.0252874317],[-0.0216611151,0.0261105595],[-0.0216137,0.0269775889],[-0.0215111765,0.0278311765],[-0.0213490151,0.0286451262],[-0.0211207305,0.0293818416],[-0.0208308727,0.0300153171],[-0.0204879411,0.0305251634],[-0.0201107584,0.0309068695],[-0.0197342967,0.0311659634],[-0.0193943742,0.0313265964],[-0.0191481463,0.0314270352],[-0.0190633394,0.0315161172],[-0.0192226152,0.0316615041]],"B1230":[[-0.0196781264,0.0319114597],[-0.0205089742,0.0323295297],[-0.02172625,0.03293625],[-0.0233535508,0.0337446619],[-0.0253325218,0.0347169663],[-0.0276053384,0.0358108939],[-0.0300899683,0.0369594127],[-0.0326695279,0.0380850834],[-0.0352462515,0.0391195848],[-0.0377460842,0.0400183064],[-0.0400668268,0.0407301601],[-0.0421446679,0.0412302235],[-0.0439474602,0.0415146824],[-0.0454305979,0.0415861535],[-0.0466050886,0.0414756442],[-0.0474691779,0.0412091779],[-0.0480460817,0.0408316372],[-0.0483642993,0.0403809659],[-0.0484500241,0.0398922464],[-0.0483408049,0.0394052493],[-0.0480486146,0.0389286146],[-0.0476129827,0.0384890938],[-0.0470312161,0.0380812161],[-0.0463046984,0.0376930318],[-0.0454318752,0.0373113197],[-0.0444007171,0.036904606],[-0.0432011176,0.036449451],[-0.0418186152,0.0359230597],[-0.0402703729,0.0353131507],[-0.0385704162,0.0346287496],[-0.0367355569,0.0338611124],[-0.0348208068,0.0330408068],[-0.0328721349,0.0322015794],[-0.0309372252,0.0313661141],[-0.0291095883,0.0305912549],[-0.0274296646,0.029894109],[-0.0259501977,0.0293068643],[-0.0247004636,0.0288254636],[-0.0236934001,0.0284389557],[-0.0229135382,0.0281207604],[-0.022341535,0.0278320905],[-0.0219369262,0.0275363706],[-0.0216576849,0.0271910182],[-0.0214675182,0.0267830737],[-0.021334747,0.0262986359],[-0.0212406796,0.0257467907],[-0.0211768347,0.0251618347],[-0.0211268267,0.0245584934],[-0.0211047236,0.0239886125],[-0.0210929594,0.0234785149],[-0.021117889,0.023077889],[-0.0211600866,0.0228067532],[-0.021222692,0.0226893587],[-0.0213056371,0.0227423038],[-0.0213979529,0.0229612863],[-0.0214840433,0.0233351544],[-0.0215662652,0.0238673764],[-0.0216260642,0.024524953],[-0.021665765,0.0252874317],[-0.0216611151,0.0261105595],[-0.0216137,0.0269775889],[-0.0215111765,0.0278311765],[-0.0213490151,0.0286451262],[-0.0211207305,0.0293818416],[-0.0208308727,0.0300153171],[-0.0204879411,0.0305251634],[-0.0201107584,0.0309068695],[-0.0197342967,0.0311659634],[-0.0193943742,0.0313265964],[-0.0191481463,0.0314270352],[-0.0190633394,0.0315161172],[-0.0192226152,0.0316615041]],"B1231":[[-0.0196781264,0.0319114597],[-0.0205089742,0.0323295297],[-0.02172625,0.03293625],[-0.0233535508,0.0337446619],[-0.0253325218,0.0347169663],[-0.0276053384,0.0358108939],[-0.0300899683,0.0369594127],[-0.0326695279,0.0380850834],[-0.0352462515,0.0391195848],[-0.0377460842,0.0400183064],[-0.0400668268,0.0407301601],[-0.0421446679,0.0412302235],[-0.0439474602,0.0415146824],[-0.0454305979,0.0415861535],[-0.0466050886,0.0414756442],[-0.0474691779,0.0412091779],[-0.0480460817,0.0408316372],[-0.0483642993,0.0403809659],[-0.0484500241,0.0398922464],[-0.0483408049,0.0394052493],[-0.0480486146,0.0389286146],[-0.0476129827,0.0384890938],[-0.0470312161,0.0380812161],[-0.0463046984,0.0376930318],[-0.0454318752,0.0373113197],[-0.0444007171,0.036904606],[-0.0432011176,0.036449451],[-0.0418186152,0.0359230597],[-0.0402703729,0.0353131507],[-0.0385704162,0.0346287496],[-0.0367355569,0.0338611124],[-0.0348208068,0.0330408068],[-0.0328721349,0.0322015794],[-0.0309372252,0.0313661141],[-0.0291095883,0.0305912549],[-0.0274296646,0.029894109],[-0.0259501977,0.0293068643],[-0.0247004636,0.0288254636],[-0.0236934001,0.0284389557],[-0.0229135382,0.0281207604],[-0.022341535,0.0278320905],[-0.0219369262,0.0275363706],[-0.0216576849,0.0271910182],[-0.0214675182,0.0267830737],[-0.021334747,0.0262986359],[-0.0212406796,0.0257467907],[-0.0211768347,0.0251618347],[-0.0211268267,0.0245584934],[-0.0211047236,0.0239886125],[-0.0210929594,0.0234785149],[-0.021117889,0.023077889],[-0.0211600866,0.0228067532],[-0.021222692,0.0226893587],[-0.0213056371,0.0227423038],[-0.0213979529,0.0229612863],[-0.0214840433,0.0233351544],[-0.0215662652,0.0238673764],[-0.0216260642,0.024524953],[-0.021665765,0.0252874317],[-0.0216611151,0.0261105595],[-0.0216137,0.0269775889],[-0.0215111765,0.0278311765],[-0.0213490151,0.0286451262],[-0.0211207305,0.0293818416],[-0.0208308727,0.0300153171],[-0.0204879411,0.0305251634],[-0.0201107584,0.0309068695],[-0.0197342967,0.0311659634],[-0.0193943742,0.0313265964],[-0.0191481463,0.0314270352],[-0.0190633394,0.0315161172],[-0.0192226152,0.0316615041]],"B1232":[[-0.0196781264,0.0319114597],[-0.0205089742,0.0323295297],[-0.02172625,0.03293625],[-0.0233535508,0.0337446619],[-0.0253325218,0.0347169663],[-0.0276053384,0.0358108939],[-0.0300899683,0.0369594127],[-0.0326695279,0.0380850834],[-0.0352462515,0.0391195848],[-0.0377460842,0.0400183064],[-0.0400668268,0.0407301601],[-0.0421446679,0.0412302235],[-0.0439474602,0.0415146824],[-0.0454305979,0.0415861535],[-0.0466050886,0.0414756442],[-0.0474691779,0.0412091779],[-0.0480460817,0.0408316372],[-0.0483642993,0.0403809659],[-0.0484500241,0.0398922464],[-0.0483408049,0.0394052493],[-0.0480486146,0.0389286146],[-0.0476129827,0.0384890938],[-0.0470312161,0.0380812161],[-0.0463046984,0.0376930318],[-0.0454318752,0.0373113197],[-0.0444007171,0.036904606],[-0.0432011176,0.036449451],[-0.0418186152,0.0359230597],[-0.0402703729,0.0353131507],[-0.0385704162,0.0346287496],[-0.0367355569,0.0338611124],[-0.0348208068,0.0330408068],[-0.0328721349,0.0322015794],[-0.0309372252,0.0313661141],[-0.0291095883,0.0305912549],[-0.0274296646,0.029894109],[-0.0259501977,0.0293068643],[-0.0247004636,0.0288254636],[-0.0236934001,0.0284389557],[-0.0229135382,0.0281207604],[-0.022341535,0.0278320905],[-0.0219369262,0.0275363706],[-0.0216576849,0.0271910182],[-0.0214675182,0.0267830737],[-0.021334747,0.0262986359],[-0.0212406796,0.0257467907],[-0.0211768347,0.0251618347],[-0.0211268267,0.0245584934],[-0.0211047236,0.0239886125],[-0.0210929594,0.0234785149],[-0.021117889,0.023077889],[-0.0211600866,0.0228067532],[-0.021222692,0.0226893587],[-0.0213056371,0.0227423038],[-0.0213979529,0.0229612863],[-0.0214840433,0.0233351544],[-0.0215662652,0.0238673764],[-0.0216260642,0.024524953],[-0.021665765,0.0252874317],[-0.0216611151,0.0261105595],[-0.0216137,0.0269775889],[-0.0215111765,0.0278311765],[-0.0213490151,0.0286451262],[-0.0211207305,0.0293818416],[-0.0208308727,0.0300153171],[-0.0204879411,0.0305251634],[-0.0201107584,0.0309068695],[-0.0197342967,0.0311659634],[-0.0193943742,0.0313265964],[-0.0191481463,0.0314270352],[-0.0190633394,0.0315161172],[-0.0192226152,0.0316615041]],"B1233":[[-0.0196781264,0.0319114597],[-0.0205089742,0.0323295297],[-0.02172625,0.03293625],[-0.0233535508,0.0337446619],[-0.0253325218,0.0347169663],[-0.0276053384,0.0358108939],[-0.0300899683,0.0369594127],[-0.0326695279,0.0380850834],[-0.0352462515,0.0391195848],[-0.0377460842,0.0400183064],[-0.0400668268,0.0407301601],[-0.0421446679,0.0412302235],[-0.0439474602,0.0415146824],[-0.0454305979,0.0415861535],[-0.0466050886,0.0414756442],[-0.0474691779,0.0412091779],[-0.0480460817,0.0408316372],[-0.0483642993,0.0403809659],[-0.0484500241,0.0398922464],[-0.0483408049,0.0394052493],[-0.0480486146,0.0389286146],[-0.0476129827,0.0384890938],[-0.0470312161,0.0380812161],[-0.0463046984,0.0376930318],[-0.0454318752,0.0373113197],[-0.0444007171,0.036904606],[-0.0432011176,0.036449451],[-0.0418186152,0.0359230597],[-0.0402703729,0.0353131507],[-0.0385704162,0.0346287496],[-0.0367355569,0.0338611124],[-0.0348208068,0.0330408068],[-0.0328721349,0.0322015794],[-0.0309372252,0.0313661141],[-0.0291095883,0.0305912549],[-0.0274296646,0.029894109],[-0.0259501977,0.0293068643],[-0.0247004636,0.0288254636],[-0.0236934001,0.0284389557],[-0.0229135382,0.0281207604],[-0.022341535,0.0278320905],[-0.0219369262,0.0275363706],[-0.0216576849,0.0271910182],[-0.0214675182,0.0267830737],[-0.021334747,0.0262986359],[-0.0212406796,0.0257467907],[-0.0211768347,0.0251618347],[-0.0211268267,0.0245584934],[-0.0211047236,0.0239886125],[-0.0210929594,0.0234785149],[-0.021117889,0.023077889],[-0.0211600866,0.0228067532],[-0.021222692,0.0226893587],[-0.0213056371,0.0227423038],[-0.0213979529,0.0229612863],[-0.0214840433,0.0233351544],[-0.0215662652,0.0238673764],[-0.0216260642,0.024524953],[-0.021665765,0.0252874317],[-0.0216611151,0.0261105595],[-0.0216137,0.0269775889],[-0.0215111765,0.0278311765],[-0.0213490151,0.0286451262],[-0.0211207305,0.0293818416],[-0.0208308727,0.0300153171],[-0.0204879411,0.0305251634],[-0.0201107584,0.0309068695],[-0.0197342967,0.0311659634],[-0.0193943742,0.0313265964],[-0.0191481463,0.0314270352],[-0.0190633394,0.0315161172],[-0.0192226152,0.0316615041]],"B1234":[[-0.035821865,0.071839365],[-0.0370473444,0.0728355586],[-0.0382597229,0.0734518657],[-0.039401731,0.0736328025],[-0.040417054,0.0733334825],[-0.0412987169,0.0725694312],[-0.0420621077,0.0713810363],[-0.0427337695,0.0698523409],[-0.043371275,0.0680773464],[-0.0440229466,0.0661672323],[-0.0447376031,0.0642461745],[-0.0455416788,0.0624102503],[-0.0464382879,0.0607247165],[-0.0474060065,0.0592524351],[-0.048397797,0.0579906541],[-0.0493560527,0.0569235527],[-0.0502032578,0.0560346863],[-0.0508775259,0.0552743117],[-0.051322987,0.0546044155],[-0.0514892243,0.05398351],[-0.0513523665,0.0533802236],[-0.0508990581,0.0527683438],[-0.0501364846,0.0521418417],[-0.0490909571,0.0514995285],[-0.0478082102,0.050859996],[-0.0463716804,0.050278109],[-0.0448616775,0.0498081061],[-0.0433851272,0.04952727],[-0.0420673957,0.0495131099],[-0.0410029728,0.0498354728],[-0.0402739969,0.0505422112],[-0.0399157579,0.0516225436],[-0.039921428,0.0530435709],[-0.040232795,0.0547088664],[-0.0407662796,0.0564894939],[-0.0414125802,0.0582390087],[-0.0420656521,0.0598217236],[-0.0426362598,0.0610991169],[-0.0430455251,0.061970168],[-0.0432611863,0.0623733292],[-0.0432431172,0.0622591887],[-0.0430202662,0.0616456233],[-0.0426253659,0.0605817944],[-0.0421132565,0.0591507565],[-0.0415622807,0.0574726378],[-0.0410586398,0.0556836398],[-0.0406875532,0.0539446961],[-0.0405155771,0.0523937914],[-0.040588337,0.0511504799],[-0.0409074899,0.0503085614],[-0.0414272976,0.0498776547],[-0.0420792198,0.0498581483],[-0.0427617185,0.0502010042],[-0.0433544117,0.0508161974],[-0.0437615103,0.051625796],[-0.0438952835,0.0525402835],[-0.0437051226,0.0534933369],[-0.0431605075,0.0544308646],[-0.0422749302,0.0553124302],[-0.0411036951,0.0561429808],[-0.0397055853,0.0569320139],[-0.0381868091,0.0577028805],[-0.0366571629,0.0584971629],[-0.035212257,0.0593693999],[-0.0339701317,0.0603529888],[-0.0330085765,0.0614835765],[-0.0323991912,0.0627834769],[-0.0321613061,0.0642184489],[-0.0323119887,0.0657844887],[-0.0328135134,0.0674117276],[-0.0336156361,0.0690245646],[-0.034641557,0.0705304855]],"B1235":[[-0.0196781264,0.0319114597],[-0.0205089742,0.0323295297],[-0.02172625,0.03293625],[-0.0233535508,0.0337446619],[-0.0253325218,0.0347169663],[-0.0276053384,0.0358108939],[-0.0300899683,0.0369594127],[-0.0326695279,0.0380850834],[-0.0352462515,0.0391195848],[-0.0377460842,0.0400183064],[-0.0400668268,0.0407301601],[-0.0421446679,0.0412302235],[-0.0439474602,0.0415146824],[-0.0454305979,0.0415861535],[-0.0466050886,0.0414756442],[-0.0474691779,0.0412091779],[-0.0480460817,0.0408316372],[-0.0483642993,0.0403809659],[-0.0484500241,0.0398922464],[-0.0483408049,0.0394052493],[-0.0480486146,0.0389286146],[-0.0476129827,0.0384890938],[-0.0470312161,0.0380812161],[-0.0463046984,0.0376930318],[-0.0454318752,0.0373113197],[-0.0444007171,0.036904606],[-0.0432011176,0.036449451],[-0.0418186152,0.0359230597],[-0.0402703729,0.0353131507],[-0.0385704162,0.0346287496],[-0.0367355569,0.0338611124],[-0.0348208068,0.0330408068],[-0.0328721349,0.0322015794],[-0.0309372252,0.0313661141],[-0.0291095883,0.0305912549],[-0.0274296646,0.029894109],[-0.0259501977,0.0293068643],[-0.0247004636,0.0288254636],[-0.0236934001,0.0284389557],[-0.0229135382,0.0281207604],[-0.022341535,0.0278320905],[-0.0219369262,0.0275363706],[-0.0216576849,0.0271910182],[-0.0214675182,0.0267830737],[-0.021334747,0.0262986359],[-0.0212406796,0.0257467907],[-0.0211768347,0.0251618347],[-0.0211268267,0.0245584934],[-0.0211047236,0.0239886125],[-0.0210929594,0.0234785149],[-0.021117889,0.023077889],[-0.0211600866,0.0228067532],[-0.021222692,0.0226893587],[-0.0213056371,0.0227423038],[-0.0213979529,0.0229612863],[-0.0214840433,0.0233351544],[-0.0215662652,0.0238673764],[-0.0216260642,0.024524953],[-0.021665765,0.0252874317],[-0.0216611151,0.0261105595],[-0.0216137,0.0269775889],[-0.0215111765,0.0278311765],[-0.0213490151,0.0286451262],[-0.0211207305,0.0293818416],[-0.0208308727,0.0300153171],[-0.0204879411,0.0305251634],[-0.0201107584,0.0309068695],[-0.0197342967,0.0311659634],[-0.0193943742,0.0313265964],[-0.0191481463,0.0314270352],[-0.0190633394,0.0315161172],[-0.0192226152,0.0316615041]],"B1236":[[-0.035821865,0.071839365],[-0.0370473444,0.0728355586],[-0.0382597229,0.0734518657],[-0.039401731,0.0736328025],[-0.040417054,0.0733334825],[-0.0412987169,0.0725694312],[-0.0420621077,0.0713810363],[-0.0427337695,0.0698523409],[-0.043371275,0.0680773464],[-0.0440229466,0.0661672323],[-0.0447376031,0.0642461745],[-0.0455416788,0.0624102503],[-0.0464382879,0.0607247165],[-0.0474060065,0.0592524351],[-0.048397797,0.0579906541],[-0.0493560527,0.0569235527],[-0.0502032578,0.0560346863],[-0.0508775259,0.0552743117],[-0.051322987,0.0546044155],[-0.0514892243,0.05398351],[-0.0513523665,0.0533802236],[-0.0508990581,0.0527683438],[-0.0501364846,0.0521418417],[-0.0490909571,0.0514995285],[-0.0478082102,0.050859996],[-0.0463716804,0.050278109],[-0.0448616775,0.0498081061],[-0.0433851272,0.04952727],[-0.0420673957,0.0495131099],[-0.0410029728,0.0498354728],[-0.0402739969,0.0505422112],[-0.0399157579,0.0516225436],[-0.039921428,0.0530435709],[-0.040232795,0.0547088664],[-0.0407662796,0.0564894939],[-0.0414125802,0.0582390087],[-0.0420656521,0.0598217236],[-0.0426362598,0.0610991169],[-0.0430455251,0.061970168],[-0.0432611863,0.0623733292],[-0.0432431172,0.0622591887],[-0.0430202662,0.0616456233],[-0.0426253659,0.0605817944],[-0.0421132565,0.0591507565],[-0.0415622807,0.0574726378],[-0.0410586398,0.0556836398],[-0.0406875532,0.0539446961],[-0.0405155771,0.0523937914],[-0.040588337,0.0511504799],[-0.0409074899,0.0503085614],[-0.0414272976,0.0498776547],[-0.0420792198,0.0498581483],[-0.0427617185,0.0502010042],[-0.0433544117,0.0508161974],[-0.0437615103,0.051625796],[-0.0438952835,0.0525402835],[-0.0437051226,0.0534933369],[-0.0431605075,0.0544308646],[-0.0422749302,0.0553124302],[-0.0411036951,0.0561429808],[-0.0397055853,0.0569320139],[-0.0381868091,0.0577028805],[-0.0366571629,0.0584971629],[-0.035212257,0.0593693999],[-0.0339701317,0.0603529888],[-0.0330085765,0.0614835765],[-0.0323991912,0.0627834769],[-0.0321613061,0.0642184489],[-0.0323119887,0.0657844887],[-0.0328135134,0.0674117276],[-0.0336156361,0.0690245646],[-0.034641557,0.0705304855]],"B1237":[[-0.035821865,0.071839365],[-0.0370473444,0.0728355586],[-0.0382597229,0.0734518657],[-0.039401731,0.0736328025],[-0.040417054,0.0733334825],[-0.0412987169,0.0725694312],[-0.0420621077,0.0713810363],[-0.0427337695,0.0698523409],[-0.043371275,0.0680773464],[-0.0440229466,0.0661672323],[-0.0447376031,0.0642461745],[-0.0455416788,0.0624102503],[-0.0464382879,0.0607247165],[-0.0474060065,0.0592524351],[-0.048397797,0.0579906541],[-0.0493560527,0.0569235527],[-0.0502032578,0.0560346863],[-0.0508775259,0.0552743117],[-0.051322987,0.0546044155],[-0.0514892243,0.05398351],[-0.0513523665,0.0533802236],[-0.0508990581,0.0527683438],[-0.0501364846,0.0521418417],[-0.0490909571,0.0514995285],[-0.0478082102,0.050859996],[-0.0463716804,0.050278109],[-0.0448616775,0.0498081061],[-0.0433851272,0.04952727],[-0.0420673957,0.0495131099],[-0.0410029728,0.0498354728],[-0.0402739969,0.0505422112],[-0.0399157579,0.0516225436],[-0.039921428,0.0530435709],[-0.040232795,0.0547088664],[-0.0407662796,0.0564894939],[-0.0414125802,0.0582390087],[-0.0420656521,0.0598217236],[-0.0426362598,0.0610991169],[-0.0430455251,0.061970168],[-0.0432611863,0.0623733292],[-0.0432431172,0.0622591887],[-0.0430202662,0.0616456233],[-0.0426253659,0.0605817944],[-0.0421132565,0.0591507565],[-0.0415622807,0.0574726378],[-0.0410586398,0.0556836398],[-0.0406875532,0.0539446961],[-0.0405155771,0.0523937914],[-0.040588337,0.0511504799],[-0.0409074899,0.0503085614],[-0.0414272976,0.0498776547],[-0.0420792198,0.0498581483],[-0.0427617185,0.0502010042],[-0.0433544117,0.0508161974],[-0.0437615103,0.051625796],[-0.0438952835,0.0525402835],[-0.0437051226,0.0534933369],[-0.0431605075,0.0544308646],[-0.0422749302,0.0553124302],[-0.0411036951,0.0561429808],[-0.0397055853,0.0569320139],[-0.0381868091,0.0577028805],[-0.0366571629,0.0584971629],[-0.035212257,0.0593693999],[-0.0339701317,0.0603529888],[-0.0330085765,0.0614835765],[-0.0323991912,0.0627834769],[-0.0321613061,0.0642184489],[-0.0323119887,0.0657844887],[-0.0328135134,0.0674117276],[-0.0336156361,0.0690245646],[-0.034641557,0.0705304855]],"B1238":[[-0.035821865,0.071839365],[-0.0370473444,0.0728355586],[-0.0382597229,0.0734518657],[-0.039401731,0.0736328025],[-0.040417054,0.0733334825],[-0.0412987169,0.0725694312],[-0.0420621077,0.0713810363],[-0.0427337695,0.0698523409],[-0.043371275,0.0680773464],[-0.0440229466,0.0661672323],[-0.0447376031,0.0642461745],[-0.0455416788,0.0624102503],[-0.0464382879,0.0607247165],[-0.0474060065,0.0592524351],[-0.048397797,0.0579906541],[-0.0493560527,0.0569235527],[-0.0502032578,0.0560346863],[-0.0508775259,0.0552743117],[-0.051322987,0.0546044155],[-0.0514892243,0.05398351],[-0.0513523665,0.0533802236],[-0.0508990581,0.0527683438],[-0.0501364846,0.0521418417],[-0.0490909571,0.0514995285],[-0.0478082102,0.050859996],[-0.0463716804,0.050278109],[-0.0448616775,0.0498081061],[-0.0433851272,0.04952727],[-0.0420673957,0.0495131099],[-0.0410029728,0.0498354728],[-0.0402739969,0.0505422112],[-0.0399157579,0.0516225436],[-0.039921428,0.0530435709],[-0.040232795,0.0547088664],[-0.0407662796,0.0564894939],[-0.0414125802,0.0582390087],[-0.0420656521,0.0598217236],[-0.0426362598,0.0610991169],[-0.0430455251,0.061970168],[-0.0432611863,0.0623733292],[-0.0432431172,0.0622591887],[-0.0430202662,0.0616456233],[-0.0426253659,0.0605817944],[-0.0421132565,0.0591507565],[-0.0415622807,0.0574726378],[-0.0410586398,0.0556836398],[-0.0406875532,0.0539446961],[-0.0405155771,0.0523937914],[-0.040588337,0.0511504799],[-0.0409074899,0.0503085614],[-0.0414272976,0.0498776547],[-0.0420792198,0.0498581483],[-0.0427617185,0.0502010042],[-0.0433544117,0.0508161974],[-0.0437615103,0.051625796],[-0.0438952835,0.0525402835],[-0.0437051226,0.0534933369],[-0.0431605075,0.0544308646],[-0.0422749302,0.0553124302],[-0.0411036951,0.0561429808],[-0.0397055853,0.0569320139],[-0.0381868091,0.0577028805],[-0.0366571629,0.0584971629],[-0.035212257,0.0593693999],[-0.0339701317,0.0603529888],[-0.0330085765,0.0614835765],[-0.0323991912,0.0627834769],[-0.0321613061,0.0642184489],[-0.0323119887,0.0657844887],[-0.0328135134,0.0674117276],[-0.0336156361,0.0690245646],[-0.034641557,0.0705304855]],"B1239":[[-0.035821865,0.071839365],[-0.0370473444,0.0728355586],[-0.0382597229,0.0734518657],[-0.039401731,0.0736328025],[-0.040417054,0.0733334825],[-0.0412987169,0.0725694312],[-0.0420621077,0.0713810363],[-0.0427337695,0.0698523409],[-0.043371275,0.0680773464],[-0.0440229466,0.0661672323],[-0.0447376031,0.0642461745],[-0.0455416788,0.0624102503],[-0.0464382879,0.0607247165],[-0.0474060065,0.0592524351],[-0.048397797,0.0579906541],[-0.0493560527,0.0569235527],[-0.0502032578,0.0560346863],[-0.0508775259,0.0552743117],[-0.051322987,0.0546044155],[-0.0514892243,0.05398351],[-0.0513523665,0.0533802236],[-0.0508990581,0.0527683438],[-0.0501364846,0.0521418417],[-0.0490909571,0.0514995285],[-0.0478082102,0.050859996],[-0.0463716804,0.050278109],[-0.0448616775,0.0498081061],[-0.0433851272,0.04952727],[-0.0420673957,0.0495131099],[-0.0410029728,0.0498354728],[-0.0402739969,0.0505422112],[-0.0399157579,0.0516225436],[-0.039921428,0.0530435709],[-0.040232795,0.0547088664],[-0.0407662796,0.0564894939],[-0.0414125802,0.0582390087],[-0.0420656521,0.0598217236],[-0.0426362598,0.0610991169],[-0.0430455251,0.061970168],[-0.0432611863,0.0623733292],[-0.0432431172,0.0622591887],[-0.0430202662,0.0616456233],[-0.0426253659,0.0605817944],[-0.0421132565,0.0591507565],[-0.0415622807,0.0574726378],[-0.0410586398,0.0556836398],[-0.0406875532,0.0539446961],[-0.0405155771,0.0523937914],[-0.040588337,0.0511504799],[-0.0409074899,0.0503085614],[-0.0414272976,0.0498776547],[-0.0420792198,0.0498581483],[-0.0427617185,0.0502010042],[-0.0433544117,0.0508161974],[-0.0437615103,0.051625796],[-0.0438952835,0.0525402835],[-0.0437051226,0.0534933369],[-0.0431605075,0.0544308646],[-0.0422749302,0.0553124302],[-0.0411036951,0.0561429808],[-0.0397055853,0.0569320139],[-0.0381868091,0.0577028805],[-0.0366571629,0.0584971629],[-0.035212257,0.0593693999],[-0.0339701317,0.0603529888],[-0.0330085765,0.0614835765],[-0.0323991912,0.0627834769],[-0.0321613061,0.0642184489],[-0.0323119887,0.0657844887],[-0.0328135134,0.0674117276],[-0.0336156361,0.0690245646],[-0.034641557,0.0705304855]],"B1240":[[-0.0196781264,0.0319114597],[-0.0205089742,0.0323295297],[-0.02172625,0.03293625],[-0.0233535508,0.0337446619],[-0.0253325218,0.0347169663],[-0.0276053384,0.0358108939],[-0.0300899683,0.0369594127],[-0.0326695279,0.0380850834],[-0.0352462515,0.0391195848],[-0.0377460842,0.0400183064],[-0.0400668268,0.0407301601],[-0.0421446679,0.0412302235],[-0.0439474602,0.0415146824],[-0.0454305979,0.0415861535],[-0.0466050886,0.0414756442],[-0.0474691779,0.0412091779],[-0.0480460817,0.0408316372],[-0.0483642993,0.0403809659],[-0.0484500241,0.0398922464],[-0.0483408049,0.0394052493],[-0.0480486146,0.0389286146],[-0.0476129827,0.0384890938],[-0.0470312161,0.0380812161],[-0.0463046984,0.0376930318],[-0.0454318752,0.0373113197],[-0.0444007171,0.036904606],[-0.0432011176,0.036449451],[-0.0418186152,0.0359230597],[-0.0402703729,0.0353131507],[-0.0385704162,0.0346287496],[-0.0367355569,0.0338611124],[-0.0348208068,0.0330408068],[-0.0328721349,0.0322015794],[-0.0309372252,0.0313661141],[-0.0291095883,0.0305912549],[-0.0274296646,0.029894109],[-0.0259501977,0.0293068643],[-0.0247004636,0.0288254636],[-0.0236934001,0.0284389557],[-0.0229135382,0.0281207604],[-0.022341535,0.0278320905],[-0.0219369262,0.0275363706],[-0.0216576849,0.0271910182],[-0.0214675182,0.0267830737],[-0.021334747,0.0262986359],[-0.0212406796,0.0257467907],[-0.0211768347,0.0251618347],[-0.0211268267,0.0245584934],[-0.0211047236,0.0239886125],[-0.0210929594,0.0234785149],[-0.021117889,0.023077889],[-0.0211600866,0.0228067532],[-0.021222692,0.0226893587],[-0.0213056371,0.0227423038],[-0.0213979529,0.0229612863],[-0.0214840433,0.0233351544],[-0.0215662652,0.0238673764],[-0.0216260642,0.024524953],[-0.021665765,0.0252874317],[-0.0216611151,0.0261105595],[-0.0216137,0.0269775889],[-0.0215111765,0.0278311765],[-0.0213490151,0.0286451262],[-0.0211207305,0.0293818416],[-0.0208308727,0.0300153171],[-0.0204879411,0.0305251634],[-0.0201107584,0.0309068695],[-0.0197342967,0.0311659634],[-0.0193943742,0.0313265964],[-0.0191481463,0.0314270352],[-0.0190633394,0.0315161172],[-0.0192226152,0.0316615041]],"K1401":[[-0.0196781264,0.0319114597],[-0.0205089742,0.0323295297],[-0.02172625,0.03293625],[-0.0233535508,0.0337446619],[-0.0253325218,0.0347169663],[-0.0276053384,0.0358108939],[-0.0300899683,0.0369594127],[-0.0326695279,0.0380850834],[-0.0352462515,0.0391195848],[-0.0377460842,0.0400183064],[-0.0400668268,0.0407301601],[-0.0421446679,0.0412302235],[-0.0439474602,0.0415146824],[-0.0454305979,0.0415861535],[-0.0466050886,0.0414756442],[-0.0474691779,0.0412091779],[-0.0480460817,0.0408316372],[-0.0483642993,0.0403809659],[-0.0484500241,0.0398922464],[-0.0483408049,0.0394052493],[-0.0480486146,0.0389286146],[-0.0476129827,0.0384890938],[-0.0470312161,0.0380812161],[-0.0463046984,0.0376930318],[-0.0454318752,0.0373113197],[-0.0444007171,0.036904606],[-0.0432011176,0.036449451],[-0.0418186152,0.0359230597],[-0.0402703729,0.0353131507],[-0.0385704162,0.0346287496],[-0.0367355569,0.0338611124],[-0.0348208068,0.0330408068],[-0.0328721349,0.0322015794],[-0.0309372252,0.0313661141],[-0.0291095883,0.0305912549],[-0.0274296646,0.029894109],[-0.0259501977,0.0293068643],[-0.0247004636,0.0288254636],[-0.0236934001,0.0284389557],[-0.0229135382,0.0281207604],[-0.022341535,0.0278320905],[-0.0219369262,0.0275363706],[-0.0216576849,0.0271910182],[-0.0214675182,0.0267830737],[-0.021334747,0.0262986359],[-0.0212406796,0.0257467907],[-0.0211768347,0.0251618347],[-0.0211268267,0.0245584934],[-0.0211047236,0.0239886125],[-0.0210929594,0.0234785149],[-0.021117889,0.023077889],[-0.0211600866,0.0228067532],[-0.021222692,0.0226893587],[-0.0213056371,0.0227423038],[-0.0213979529,0.0229612863],[-0.0214840433,0.0233351544],[-0.0215662652,0.0238673764],[-0.0216260642,0.024524953],[-0.021665765,0.0252874317],[-0.0216611151,0.0261105595],[-0.0216137,0.0269775889],[-0.0215111765,0.0278311765],[-0.0213490151,0.0286451262],[-0.0211207305,0.0293818416],[-0.0208308727,0.0300153171],[-0.0204879411,0.0305251634],[-0.0201107584,0.0309068695],[-0.0197342967,0.0311659634],[-0.0193943742,0.0313265964],[-0.0191481463,0.0314270352],[-0.0190633394,0.0315161172],[-0.0192226152,0.0316615041]],"L1501":[[-0.035821865,0.071839365],[-0.0370473444,0.0728355586],[-0.0382597229,0.0734518657],[-0.039401731,0.0736328025],[-0.040417054,0.0733334825],[-0.0412987169,0.0725694312],[-0.0420621077,0.0713810363],[-0.0427337695,0.0698523409],[-0.043371275,0.0680773464],[-0.0440229466,0.0661672323],[-0.0447376031,0.0642461745],[-0.0455416788,0.0624102503],[-0.0464382879,0.0607247165],[-0.0474060065,0.0592524351],[-0.048397797,0.0579906541],[-0.0493560527,0.0569235527],[-0.0502032578,0.0560346863],[-0.0508775259,0.0552743117],[-0.051322987,0.0546044155],[-0.0514892243,0.05398351],[-0.0513523665,0.0533802236],[-0.0508990581,0.0527683438],[-0.0501364846,0.0521418417],[-0.0490909571,0.0514995285],[-0.0478082102,0.050859996],[-0.0463716804,0.050278109],[-0.0448616775,0.0498081061],[-0.0433851272,0.04952727],[-0.0420673957,0.0495131099],[-0.0410029728,0.0498354728],[-0.0402739969,0.0505422112],[-0.0399157579,0.0516225436],[-0.039921428,0.0530435709],[-0.040232795,0.0547088664],[-0.0407662796,0.0564894939],[-0.0414125802,0.0582390087],[-0.0420656521,0.0598217236],[-0.0426362598,0.0610991169],[-0.0430455251,0.061970168],[-0.0432611863,0.0623733292],[-0.0432431172,0.0622591887],[-0.0430202662,0.0616456233],[-0.0426253659,0.0605817944],[-0.0421132565,0.0591507565],[-0.0415622807,0.0574726378],[-0.0410586398,0.0556836398],[-0.0406875532,0.0539446961],[-0.0405155771,0.0523937914],[-0.040588337,0.0511504799],[-0.0409074899,0.0503085614],[-0.0414272976,0.0498776547],[-0.0420792198,0.0498581483],[-0.0427617185,0.0502010042],[-0.0433544117,0.0508161974],[-0.0437615103,0.051625796],[-0.0438952835,0.0525402835],[-0.0437051226,0.0534933369],[-0.0431605075,0.0544308646],[-0.0422749302,0.0553124302],[-0.0411036951,0.0561429808],[-0.0397055853,0.0569320139],[-0.0381868091,0.0577028805],[-0.0366571629,0.0584971629],[-0.035212257,0.0593693999],[-0.0339701317,0.0603529888],[-0.0330085765,0.0614835765],[-0.0323991912,0.0627834769],[-0.0321613061,0.0642184489],[-0.0323119887,0.0657844887],[-0.0328135134,0.0674117276],[-0.0336156361,0.0690245646],[-0.034641557,0.0705304855]],"L1502":[[-0.035821865,0.071839365],[-0.0370473444,0.0728355586],[-0.0382597229,0.0734518657],[-0.039401731,0.0736328025],[-0.040417054,0.0733334825],[-0.0412987169,0.0725694312],[-0.0420621077,0.0713810363],[-0.0427337695,0.0698523409],[-0.043371275,0.0680773464],[-0.0440229466,0.0661672323],[-0.0447376031,0.0642461745],[-0.0455416788,0.0624102503],[-0.0464382879,0.0607247165],[-0.0474060065,0.0592524351],[-0.048397797,0.0579906541],[-0.0493560527,0.0569235527],[-0.0502032578,0.0560346863],[-0.0508775259,0.0552743117],[-0.051322987,0.0546044155],[-0.0514892243,0.05398351],[-0.0513523665,0.0533802236],[-0.0508990581,0.0527683438],[-0.0501364846,0.0521418417],[-0.0490909571,0.0514995285],[-0.0478082102,0.050859996],[-0.0463716804,0.050278109],[-0.0448616775,0.0498081061],[-0.0433851272,0.04952727],[-0.0420673957,0.0495131099],[-0.0410029728,0.0498354728],[-0.0402739969,0.0505422112],[-0.0399157579,0.0516225436],[-0.039921428,0.0530435709],[-0.040232795,0.0547088664],[-0.0407662796,0.0564894939],[-0.0414125802,0.0582390087],[-0.0420656521,0.0598217236],[-0.0426362598,0.0610991169],[-0.0430455251,0.061970168],[-0.0432611863,0.0623733292],[-0.0432431172,0.0622591887],[-0.0430202662,0.0616456233],[-0.0426253659,0.0605817944],[-0.0421132565,0.0591507565],[-0.0415622807,0.0574726378],[-0.0410586398,0.0556836398],[-0.0406875532,0.0539446961],[-0.0405155771,0.0523937914],[-0.040588337,0.0511504799],[-0.0409074899,0.0503085614],[-0.0414272976,0.0498776547],[-0.0420792198,0.0498581483],[-0.0427617185,0.0502010042],[-0.0433544117,0.0508161974],[-0.0437615103,0.051625796],[-0.0438952835,0.0525402835],[-0.0437051226,0.0534933369],[-0.0431605075,0.0544308646],[-0.0422749302,0.0553124302],[-0.0411036951,0.0561429808],[-0.0397055853,0.0569320139],[-0.0381868091,0.0577028805],[-0.0366571629,0.0584971629],[-0.035212257,0.0593693999],[-0.0339701317,0.0603529888],[-0.0330085765,0.0614835765],[-0.0323991912,0.0627834769],[-0.0321613061,0.0642184489],[-0.0323119887,0.0657844887],[-0.0328135134,0.0674117276],[-0.0336156361,0.0690245646],[-0.034641557,0.0705304855]],"L1503":[[-0.035821865,0.071839365],[-0.0370473444,0.0728355586],[-0.0382597229,0.0734518657],[-0.039401731,0.0736328025],[-0.040417054,0.0733334825],[-0.0412987169,0.0725694312],[-0.0420621077,0.0713810363],[-0.0427337695,0.0698523409],[-0.043371275,0.0680773464],[-0.0440229466,0.0661672323],[-0.0447376031,0.0642461745],[-0.0455416788,0.0624102503],[-0.0464382879,0.0607247165],[-0.0474060065,0.0592524351],[-0.048397797,0.0579906541],[-0.0493560527,0.0569235527],[-0.0502032578,0.0560346863],[-0.0508775259,0.0552743117],[-0.051322987,0.0546044155],[-0.0514892243,0.05398351],[-0.0513523665,0.0533802236],[-0.0508990581,0.0527683438],[-0.0501364846,0.0521418417],[-0.0490909571,0.0514995285],[-0.0478082102,0.050859996],[-0.0463716804,0.050278109],[-0.0448616775,0.0498081061],[-0.0433851272,0.04952727],[-0.0420673957,0.0495131099],[-0.0410029728,0.0498354728],[-0.0402739969,0.0505422112],[-0.0399157579,0.0516225436],[-0.039921428,0.0530435709],[-0.040232795,0.0547088664],[-0.0407662796,0.0564894939],[-0.0414125802,0.0582390087],[-0.0420656521,0.0598217236],[-0.0426362598,0.0610991169],[-0.0430455251,0.061970168],[-0.0432611863,0.0623733292],[-0.0432431172,0.0622591887],[-0.0430202662,0.0616456233],[-0.0426253659,0.0605817944],[-0.0421132565,0.0591507565],[-0.0415622807,0.0574726378],[-0.0410586398,0.0556836398],[-0.0406875532,0.0539446961],[-0.0405155771,0.0523937914],[-0.040588337,0.0511504799],[-0.0409074899,0.0503085614],[-0.0414272976,0.0498776547],[-0.0420792198,0.0498581483],[-0.0427617185,0.0502010042],[-0.0433544117,0.0508161974],[-0.0437615103,0.051625796],[-0.0438952835,0.0525402835],[-0.0437051226,0.0534933369],[-0.0431605075,0.0544308646],[-0.0422749302,0.0553124302],[-0.0411036951,0.0561429808],[-0.0397055853,0.0569320139],[-0.0381868091,0.0577028805],[-0.0366571629,0.0584971629],[-0.035212257,0.0593693999],[-0.0339701317,0.0603529888],[-0.0330085765,0.0614835765],[-0.0323991912,0.0627834769],[-0.0321613061,0.0642184489],[-0.0323119887,0.0657844887],[-0.0328135134,0.0674117276],[-0.0336156361,0.0690245646],[-0.034641557,0.0705304855]],"M1501":[[-0.035821865,0.071839365],[-0.0370473444,0.0728355586],[-0.0382597229,0.0734518657],[-0.039401731,0.0736328025],[-0.040417054,0.0733334825],[-0.0412987169,0.0725694312],[-0.0420621077,0.0713810363],[-0.0427337695,0.0698523409],[-0.043371275,0.0680773464],[-0.0440229466,0.0661672323],[-0.0447376031,0.0642461745],[-0.0455416788,0.0624102503],[-0.0464382879,0.0607247165],[-0.0474060065,0.0592524351],[-0.048397797,0.0579906541],[-0.0493560527,0.0569235527],[-0.0502032578,0.0560346863],[-0.0508775259,0.0552743117],[-0.051322987,0.0546044155],[-0.0514892243,0.05398351],[-0.0513523665,0.0533802236],[-0.0508990581,0.0527683438],[-0.0501364846,0.0521418417],[-0.0490909571,0.0514995285],[-0.0478082102,0.050859996],[-0.0463716804,0.050278109],[-0.0448616775,0.0498081061],[-0.0433851272,0.04952727],[-0.0420673957,0.0495131099],[-0.0410029728,0.0498354728],[-0.0402739969,0.0505422112],[-0.0399157579,0.0516225436],[-0.039921428,0.0530435709],[-0.040232795,0.0547088664],[-0.0407662796,0.0564894939],[-0.0414125802,0.0582390087],[-0.0420656521,0.0598217236],[-0.0426362598,0.0610991169],[-0.0430455251,0.061970168],[-0.0432611863,0.0623733292],[-0.0432431172,0.0622591887],[-0.0430202662,0.0616456233],[-0.0426253659,0.0605817944],[-0.0421132565,0.0591507565],[-0.0415622807,0.0574726378],[-0.0410586398,0.0556836398],[-0.0406875532,0.0539446961],[-0.0405155771,0.0523937914],[-0.040588337,0.0511504799],[-0.0409074899,0.0503085614],[-0.0414272976,0.0498776547],[-0.0420792198,0.0498581483],[-0.0427617185,0.0502010042],[-0.0433544117,0.0508161974],[-0.0437615103,0.051625796],[-0.0438952835,0.0525402835],[-0.0437051226,0.0534933369],[-0.0431605075,0.0544308646],[-0.0422749302,0.0553124302],[-0.0411036951,0.0561429808],[-0.0397055853,0.0569320139],[-0.0381868091,0.0577028805],[-0.0366571629,0.0584971629],[-0.035212257,0.0593693999],[-0.0339701317,0.0603529888],[-0.0330085765,0.0614835765],[-0.0323991912,0.0627834769],[-0.0321613061,0.0642184489],[-0.0323119887,0.0657844887],[-0.0328135134,0.0674117276],[-0.0336156361,0.0690245646],[-0.034641557,0.0705304855]]}}
```

In [3]:

```
data_df = pd.DataFrame.from_dict(data)
data_df
```

Out[3]:

|  | chl\_id | loc\_res\_Mg | bin | scan\_angles | scan\_amp\_C2 | scan\_amp\_C7 | ci |
| --- | --- | --- | --- | --- | --- | --- | --- |
| A1011 | A11 | 2.83069 | 0 | [0.0, 5.0, 10.0, 15.0, 20.0, 25.0, 30.0, 35.0,... | [0.0129, 0.01332, 0.01395, 0.01476, 0.0157, 0.... | [0.05098, 0.05026, 0.04912, 0.04752, 0.04548, ... | [[-0.035821865, 0.071839365], [-0.0370473444, ... |
| A1012 | A12 | 2.81667 | 0 | [0.0, 5.0, 10.0, 15.0, 20.0, 25.0, 30.0, 35.0,... | [-0.02389, -0.02806, -0.03202, -0.03567, -0.03... | [0.03589, 0.03423, 0.03175, 0.02853, 0.02474, ... | [[-0.035821865, 0.071839365], [-0.0370473444, ... |
| A1013 | A13 | 2.84799 | 0 | [0.0, 5.0, 10.0, 15.0, 20.0, 25.0, 30.0, 35.0,... | [0.02966, 0.03034, 0.03056, 0.03032, 0.02962, ... | [0.03642, 0.03842, 0.03937, 0.03916, 0.03778, ... | [[-0.035821865, 0.071839365], [-0.0370473444, ... |
| A1101 | A01 | 2.94216 | 1 | [0.0, 5.0, 10.0, 15.0, 20.0, 25.0, 30.0, 35.0,... | [-0.00236, -0.00218, -0.00194, -0.00164, -0.00... | [0.01043, 0.01117, 0.01175, 0.01214, 0.01235, ... | [[-0.0196781264, 0.0319114597], [-0.0205089742... |
| A1102 | A02 | 2.91775 | 1 | [0.0, 5.0, 10.0, 15.0, 20.0, 25.0, 30.0, 35.0,... | [0.03146, 0.03127, 0.03077, 0.02996, 0.02889, ... | [-0.0059, -0.00679, -0.00769, -0.0086, -0.0094... | [[-0.0196781264, 0.0319114597], [-0.0205089742... |
| ... | ... | ... | ... | ... | ... | ... | ... |
| K1401 | K01 | 3.04320 | 1 | [0.0, 5.0, 10.0, 15.0, 20.0, 25.0, 30.0, 35.0,... | [0.00455, 0.00405, 0.00353, 0.00298, 0.00239, ... | [0.0029, 0.00365, 0.00442, 0.00518, 0.0059, 0.... | [[-0.0196781264, 0.0319114597], [-0.0205089742... |
| L1501 | L01 | 2.82427 | 0 | [0.0, 5.0, 10.0, 15.0, 20.0, 25.0, 30.0, 35.0,... | [0.0068, 0.00865, 0.01057, 0.01255, 0.01453, 0... | [0.04567, 0.04813, 0.05026, 0.05201, 0.05328, ... | [[-0.035821865, 0.071839365], [-0.0370473444, ... |
| L1502 | L02 | 2.81980 | 0 | [0.0, 5.0, 10.0, 15.0, 20.0, 25.0, 30.0, 35.0,... | [0.04098, 0.04115, 0.04072, 0.03968, 0.03807, ... | [0.02533, 0.0244, 0.02325, 0.02192, 0.02045, 0... | [[-0.035821865, 0.071839365], [-0.0370473444, ... |
| L1503 | L03 | 2.82468 | 0 | [0.0, 5.0, 10.0, 15.0, 20.0, 25.0, 30.0, 35.0,... | [0.02561, 0.02386, 0.02189, 0.01974, 0.01748, ... | [0.00184, 0.003, 0.00433, 0.00582, 0.00742, 0.... | [[-0.035821865, 0.071839365], [-0.0370473444, ... |
| M1501 | M01 | 2.84425 | 0 | [0.0, 5.0, 10.0, 15.0, 20.0, 25.0, 30.0, 35.0,... | [-0.01432, -0.01465, -0.01506, -0.01558, -0.01... | [0.01094, 0.0092, 0.00742, 0.00559, 0.00376, 0... | [[-0.035821865, 0.071839365], [-0.0370473444, ... |

92 rows × 7 columns

#### Local Resolution and Binning¶

The ESP map local resolution at the central Mg of each chlorophyll is shown in the graph below. The colors indicate the binning groups used.

In [4]:

```
data_df.sort_values(by = ['loc_res_Mg'], inplace = True)
res_Mg = np.array(data_df['loc_res_Mg'].to_list())
bin0 = [res for res in res_Mg if res < 2.91]
bin1 = [res for res in res_Mg if res > 2.91]
x0 = np.arange(len(bin0))
x1 = np.arange(len(bin1)) + len(bin0)

fig, ax = plt.subplots(figsize = (6, 4))
ax.plot(x0, bin0, c = 'tab:blue', ls = 'none', marker = 'o', ms = 2, label = "bin: 0")
ax.plot(x1, bin1, c = 'tab:orange', ls = 'none', marker = 'o', ms = 2, label = "bin: 1")
ax.set_xlabel('chlorophyll index')
ax.set_ylabel("Resolution ($\AA$)")
ax.set_title('Resolution Binning', fontweight="bold")
ax.legend(loc = 'lower right')
```

Out[4]:

```
<matplotlib.legend.Legend at 0x7f1d69342c18>
```

#### Cone Scans for all Chlorophyll¶

The cone scans for each chlorophyll compared to the chlorophyll's corresponding null distribution is shown below.

In [5]:

```
data_df.sort_index(inplace = True)

fig, axs = plt.subplots(23, 4, figsize = (13, 65), sharex = True, sharey = True)
plt.setp(fig, tight_layout = True)

all_chl_ids = data_df.index.to_list()
for chl_id, ax in zip(all_chl_ids, axs.flat):
    # Data
    row = data_df.loc[chl_id]
    scan_angles = np.array(row['scan_angles'])
    scan_amp_C2 = np.array(row['scan_amp_C2'])
    scan_amp_C7 = np.array(row['scan_amp_C7'])
    ci = np.array(row['ci'])
    chl_site = row['chl_id'][0] + str(int(row['chl_id'][1:]))
    res_Mg = str(row['loc_res_Mg'])

    # Plotting
    l_c2 = ax.plot(scan_angles, scan_amp_C2*10)
    ci_fill = ax.fill_between(scan_angles, *ci.T*10)
    h_l = ax.axhline()

    ## Making things pretty
    plt.setp(l_c2, ls = '-', color = "tab:blue")
    plt.setp(ci_fill, color = 'tab:orange', alpha = 0.2)
    plt.setp(h_l, color = 'black', ls = '--')
    plt.setp(ax, xlim = [0, 355])
    plt.setp(ax, ylabel = 'ESP', xlabel = "Scan Angle ($^\circ$)")
    subplot_title = f"{chl_site} | {res_Mg[:4]} $\AA$"
    ax.set_title(subplot_title)
    ax.label_outer()

# custom shared legend
ax_label = fig.add_subplot(111) # Label Subplot
ax_label.axis('off')
fill = mpatches.Patch(color = 'tab:orange',
                      alpha = 0.2,
                      label = 'Null Distribution')
line = mlines.Line2D([], [], color = 'tab:blue',
                     label = 'C2 Cone Scan')
ax_label.legend(handles = [line, fill],
                prop = {"size":15},
                ncol = 2,
                loc = 'upper center',
                bbox_to_anchor = (0.5, 1.015))
```

Out[5]:

```
<matplotlib.legend.Legend at 0x7f1d68f94e48>
```
